# Anomalous effect of non-alternant hydrocarbons on carbocation and carbanion electronic configurations†
†Electronic supplementary information (ESI) available. See DOI: 10.1039/c7sc01047h


**DOI:** 10.1039/c7sc01047h

**Published:** 2017-05-04

**Authors:** Logan J. Fischer, Andrew S. Dutton, Arthur H. Winter

**Affiliations:** a Department of Chemistry , Iowa State University , 1608 Gilman Hall , Ames , IA 50010 , USA . Email: winter@iastate.edu

## Abstract

Carbocations are widely viewed to be closed-shell singlet electrophiles. Here, computations show that azulenyl-substituted carbocations have triplet ground states. This triplet ground state for azulenyl carbocations stands in striking contrast to the isomeric naphthenyl carbocation, which is a normal closed-shell singlet with a large singlet–triplet gap. Furthermore, substitution of the azulenyl carbocation can substantially alter the energy gap between the different electronic configurations and can manipulate the ground state towards either the singlet or the triplet state depending on the nature and location of the substituent. A detailed investigation into the origin of this spin state reversal, including NICS calculations, structural effects, substitution patterns, orbital analysis, and detailed linear free-energy relationships allowed us to distill a set of principles that caused these azulenyl carbocations to have such low-lying diradical states. The fundamental origin of this effect mostly centers on singlet state destabilization from increasing antiaromatic character, in combination with a smaller, but important, Baird triplet state aromatic stabilization. We find that azulene is not unique, as extension of these principles allowed us to generate simple rules to predict an entire class of analogous non-alternant carbocation and carbanion structures with low-energy or ground state diradical states, including a purely hydrocarbon triplet cation with a large singlet–triplet gap of 8 kcal mol^–1^. Although these ions have innocuous-looking Lewis structures, these triplet diradical ions are likely to have substantially different reactivity and properties than typical closed-shell singlet ions.

## Abstract 2

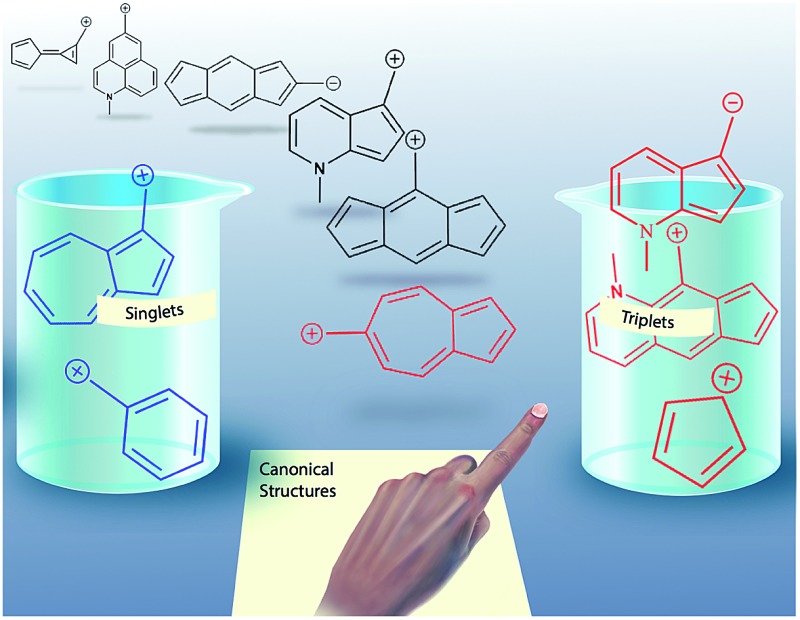
Simple rules based on canonical structures allow for the prediction of a new class of triplet carbocation and carbanion species.

## Introduction

The textbook view of the electronic structure of a carbocation is that of an approximately sp^2^ hybridized carbon with an empty p orbital.^1^ This simplistic picture stands in sharp contrast to the related nitrenes,^2^–^6^ carbenes,^7^–^10^ nitrenium ions,^11^–^14^ oxenium ions,^15^–^19^ and other reactive intermediates that possess one or more lone pairs of electrons that can be distributed between two orbitals, allowing (at least) two closed-shell singlet configurations, an open-shell singlet configuration, and a triplet configuration, each of which potentially offers up a unique landscape of reactivity and properties. Since carbocations do not possess a lone pair, and given early theoretical investigations by Schleyer suggested that simple carbocations had large energy gaps to open-shell states,^20^ the simple view of the electrophilic closed-shell singlet carbocation has largely remained unchallenged.

Exceptions exist, however. Antiaromatic carbocations with low-energy diradical states,^21^ and in some cases high-spin triplet states, have been found. The most famous of these is the antiaromatic cyclopentadienyl cation,^22^ a textbook example of an antiaromatic ring which has an observable EPR spectrum. The cyclopentadienyl cation and its derivatives are unusual in having a triplet ground state, as other formally 4*n*-antiaromatic cations undergo Jahn–Teller structural distortions that stabilize the singlet state below the triplet. Recent computational and experimental investigations of a *meta*-donor-substituted benzylic cation have suggested that a triplet configuration is nearly degenerate with the closed-shell singlet.^23^,^24^ We recently elaborated upon this discovery by showing that this ion was just one member of a class of donor-unconjugated carbocations, which have low-energy or diradical ground states.^25^

Cyclic, conjugated hydrocarbons, like azulene and naphthalene, can be classified into one of two categories: alternant or non-alternant hydrocarbons. Coulson and Rushbrooke classified cyclic, conjugated hydrocarbons in this fashion, using the well-known starring method for determining if a cyclic, conjugated hydrocarbon belongs to the alternant or non-alternant category.^26^

Here, we demonstrate that carbocations conjugated to the non-alternant hydrocarbon azulene can have low-energy or ground state triplets depending on the point of attachment. A detailed investigation into the origin of this spin state reversal, including NICS calculations, structural effects, substitution patterns, detailed linear free-energy relationships (LFER), and orbital analyses, allowed us to distill a set of principles that explains why these azulenyl carbocations have low-lying diradical states. These principles allowed for the prediction of a set of analogous structures containing not only carbocations, but also carbanions that exhibit low-lying or ground state triplets using simple canonical structure ideas. These examples point to not only a new and potentially broad class of carbocations that defy the textbook paradigm that carbocations are electrophilic species with closed-shell singlet ground states, but also to a new class of high-spin carbanions that may have application to high-spin materials.

## Computational methods

In order to investigate the electronic states of the carbocations of interest in this study, singlet–triplet gaps (Δ*G*_ST_) were computed using DFT (B3LYP^27^–^29^), CBS (CBS-QB3 30,31), and CASPT2 32,33 methods. Using the standard convention, Δ*G*_ST_ refers to the gas-phase Gibbs free energy difference between the lowest energy singlet state and the lowest energy triplet state. A value of Δ*G*_ST_ that is positive indicates a triplet ground state, whereas a value of Δ*G*_ST_ that is negative indicates a singlet ground state.

A number of studies have shown that the B3LYP functional performs well when compared to experimental or multireference computational methods, such as CASPT2, for computing Δ*G*_ST_ of related hypovalent species, such as nitrenium ions, oxenium ions, and carbenes.^23^,^34^–^38^

Triplet species are formed by a narrowing of the frontier orbitals, and it is always a concern in these cases that the singlet structures have a multireference state. In these cases, the inability of the B3LYP and CBS-QB3 methods to account for open-shell character needs to be explicitly addressed. In this study, an unrestricted broken-symmetry approach to computing the singlet states was used for B3LYP calculations, in combination with CASPT2 benchmarking. Unfortunately, this approach using DFT very frequently suffers from considerable spin contamination when there is also a low-energy triplet state, which is indicated by *S*^2^ values greater than zero. In cases where broken-symmetry DFT calculations were performed, eqn (1) was used to titrate out contamination from a low-energy triplet state and determine a spin-purified energy of the singlet state.^39^–^42^
1
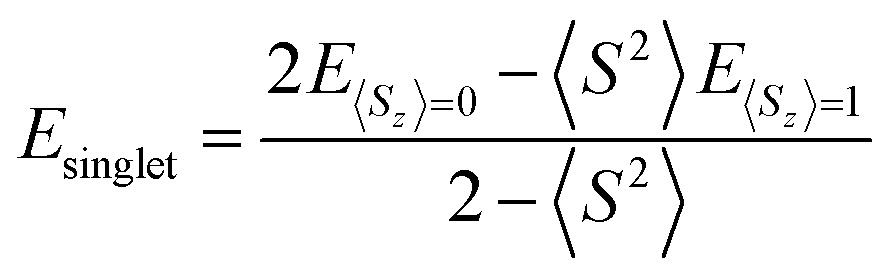
where *E*_singlet_ is the corrected singlet energy, *E*_*S*_*z*_=0_ is the broken-symmetry energy, *S*^2^ is the expectation value of the total-spin operator for the broken-symmetry calculation (anywhere from about zero to one), and *E*_*S*_*z*_=1_ is the energy of the triplet state at the singlet geometry.

In addition to trends, we are also interested in absolute quantitative accuracy. In order to evaluate the effectiveness of the computational methods for obtaining the Δ*G*_ST_ values detailed here, benchmarking studies against a small set of experimentally known Δ*G*_ST_ of related hypovalent molecules were conducted. As can be seen in Table 1, reasonable accuracy is seen between our chosen methods and the experimental data. Calculation of the Δ*G*_ST_ using DFT has a root-mean square deviation (RMSD) error of 2.9 kcal mol^–1^ and a maximum deviation from the experimental value of 6.2 kcal mol^–1^. CBS-QB3 gives a RMSD of 1.3 kcal mol^–1^ and a maximum deviation from the experimental value by 2.3 kcal mol^–1^. Generally, one obtains accurate results for CASPT2 calculations when an appropriate active space and a flexible basis set are used. With a RMSD error of 2.8 kcal mol^–1^ and a maximum error of 4.6 kcal mol^–1^ using CASPT2, the CASPT2 and B3LYP results are comparable. The CBS-QB3 method appears to provide the best quantitative accuracy, as would be expected, but the CASPT2 method was employed to provide added confidence in the single-reference methods because of its ability to handle non-dynamical correlation.

**Table 1:** Experimental Δ*G*_ST_ values compared to computed Δ*G*_ST_

Compound	Δ*G*_ST_			
	Exp.	B3LYP^*a*^	CBS^*b*^	CASPT2^*c*^
Methylene	9.2 43	7.5	8.3	12.4 (5, 6)
Amidogen	30.1 44	29.0	28.8	32.4 (5, 6)
Difluoromethylene	–57.0 43	–51.6	–56.1	–54.1 (10, 10)
Phenylcarbene	2.3 45	2.4	4.6	6.9 (8, 8)
Phenyloxenium	–19.8 46	–13.6	–18.6	–18.6 (10, 12)
Benzyl cation	–44.5 47	–38.9	–43.7	–43.7 (6, 7)
RMSD error		2.4	1.3	2.5
Maximum error		6.2	2.3	4.6

^*a*^UB3LYP/6-31+G(d,p).

^*b*^CBS-QB3.

^*c*^CASPT2/ANO-L-VTZP//CBSB7.

**Fig. 1:**
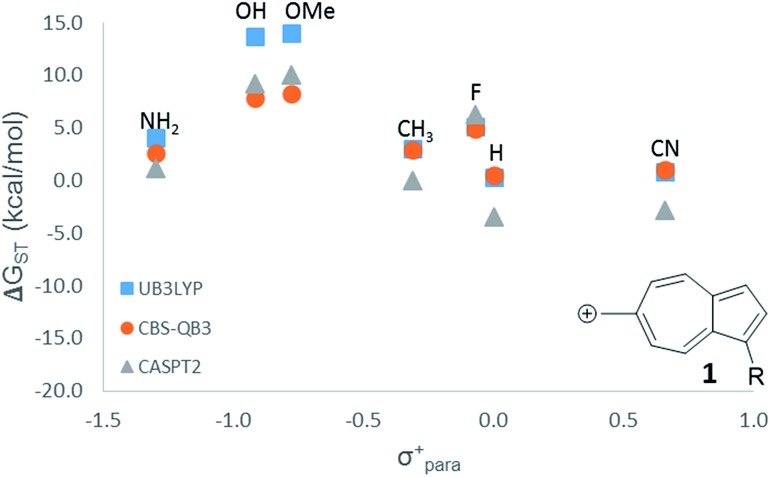
LFER comparing the three methods used in this study. (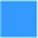) UB3LYP/6-31+G(d,p) (corrected singlet) (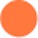) CBS-QB3 (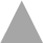) CASPT2/ANO-L-VTZP//CBSB7.

For the numerous Hammett-like linear free-energy relationships and the testing of hypothesized low-lying or ground state triplet structures described later, we elected to use the more economical B3LYP level of theory. To ensure that this method is sufficiently accurate to detect the trends, we utilized all three computational methods (CASPT2, CBS-QB3, and B3LYP) on one LFER plot, which all three methods gave nearly identical results (Fig. 1).

Complete basis set (CBS-QB3), Hartree–Fock (HF), and density functional theory (DFT) computations were performed using the Gaussian 09 software suite.^48^ DFT computations were performed employing the B3LYP functional, along with the 6-31+G(d,p) polarized double-ζ basis set. Hartree–Fock computations were performed to determine nuclear independent chemical shift (NICS) values, using the GIAO method^49^ and the 6-31+G(d,p) basis set. In all cases, optimized geometries were found to have zero imaginary frequencies and corrections for the zero-point vibrational energy and thermal energy were added unscaled. CASPT2 single-point energy calculations were performed on the CBS-QB3 geometries using the Molcas 8 software suite^50^–^52^ and the ANO-L-VTZP basis set,^53^,^54^ which is of polarized valence triple zeta quality. Orbitals chosen to define the active space for CASPT2 calculations for experimentally known compounds are depicted in the ESI† and were visualized using IboView.^55^,^56^ Active orbitals for other structures were selected so as to include all π orbitals. Values for the thermal correction to Gibbs free energy were taken from the CBSB7 frequency calculation within the CBS-QB3 calculation and added to the CASPT2 energies to obtain Gibbs free energy. All calculations were performed in the gas-phase at the default temperature (298.15 K). When multiple rotamers were possible, the lowest energy one from each spin state was used to calculate the Δ*G*_ST_ value.

## Results and discussion

### Azulenyl carbocations can have triplet ground states

**Fig. 2:**
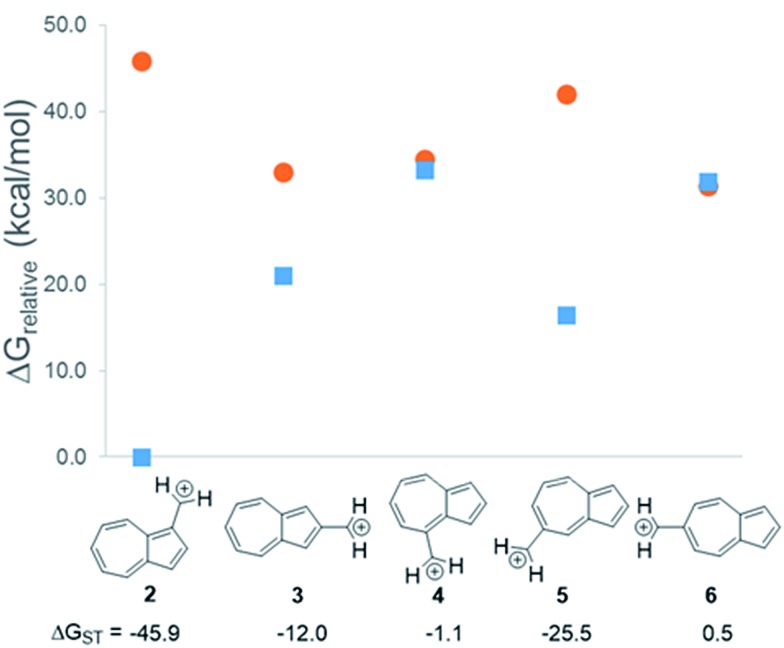
Effect on singlet and triplet carbocation energies with substitution of azulene. Δ*G*_ST_ in kcal mol^–1^ ((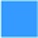) singlet, (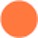) triplet) CBS-QB3.

In changing the position of substitution of the non-alternant hydrocarbon azulene in ions **2–6**, a striking change in the Δ*G*_ST_ is observed (Fig. 2). Calculations using CBS-QB3 show the substitution at the 1-position of azulene (**2**) gives the most singlet favored Δ*G*_ST_ of –45.9 kcal mol^–1^, in line with the experimental Δ*G*_ST_ of benzyl cation, obtained by photoelectron spectroscopy, of –44.5 kcal mol^–1^.^47^ In contrast to the singlet favored **2** is **6** with a triplet favored Δ*G*_ST_ of +0.5 kcal mol^–1^. It is astonishing that the purely hydrocarbon “benzylic” cation **6** is computed to have essentially degenerate singlet and triplet states. By comparison, the isomeric aromatic hydrocarbon, naphthalene, which, like azulene, is an aromatic molecule with 10 π electrons and differs structurally only by the ring-attachment point, has two possible cation isomers. Both of these isomers are computed to be singlet ground state species by –34.7 and –28.1 kcal mol^–1^ – that is, be “normal” closed-shell singlet carbocations with large energy gaps to a diradical excited state.

What is the origin of this inversion of ground spin states for azulenyl cation? Fortunately, these structures are isomers and their absolute energies can be directly compared to determine the relative stabilities of the singlet and triplet states. As seen in Fig. 2, the difference in the Δ*G*_ST_ between **2** and **6** comes from a destabilization of the singlet state, along with a stabilization of the triplet state for **6** compared to **2**. Note that ions **3**, **4**, and **6** have nearly identical stabilized triplet energies compared to **2**, but only **6** is computed to have a triplet ground state due to a more destabilized singlet state. Ion **4** has a more destabilized singlet state than **6**, but is computed to narrowly have a singlet ground state due to the triplet state being slightly higher in energy than in **6**.

### Changes in triplet state energies cannot be explained by differences in exchange energies

To identify the origin of these relative energy differences, the triplet state energies were examined. The most obvious explanation for the differences in the triplet energies between **2** and **6** could be potentially explained by varying magnitudes of triplet-favoring electron exchange. As described by Borden and Davidson,^57^ the orbitals of the unpaired electrons of a diradical can be described in two fashions: disjoint or non-disjoint. If the orbital amplitudes of the SOMOs occupy the same atoms, the orbitals are classified as non-disjoint and the unpaired electrons will have large exchange integrals, favoring the triplet configuration. This preference of the electrons to be in a spin unpaired state can be attributed to the reduction of electron–electron repulsion gained by the unpaired spins. If the orbital amplitudes of the SOMOs occupy different atoms, the orbitals are said to be disjoint. Since the electrons placed in disjoint orbitals do not occupy the same physical space, there is no exchange energy to be gained by adopting a triplet *versus* singlet configuration.

**Fig. 3:**
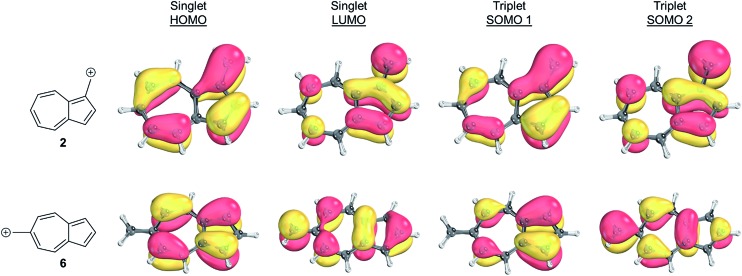
Orbital representations^61^,^62^ of the HOMO, LUMO, SOMO 1, and SOMO 2 of structures **2** and **6**. Threshold of 80.00 was used.

Inspecting the SOMOs of **2** and **6** (Fig. 3), the most singlet and triplet favored structures respectively, rules out this facile explanation that exchange energy accounts for the differences in triplet stabilization. In these cases, it can be seen that the SOMOs of both structures can be classified as non-disjoint as there is sharing of SOMO amplitudes on the same carbons. In fact, **2** has more shared SOMO amplitudes than **6**, contrary to the computed difference in triplet energies, indicating an alternate explanation is required.

### Triplet stabilization and singlet destabilization of 6 can be explained by antiaromaticity/Baird aromaticity arguments

Aromaticity/antiaromaticity is a useful construct to explain the special stability or instability of cyclic, π-conjugated structures. Ideas of aromaticity/antiaromaticity to explain the stability of cyclic, conjugated systems having a closed-shell singlet configuration was first suggested by Erich Hückel^58^ and later formulated as the 4*n* + 2 rule. In 1971, Baird discovered that structures that were hypothesized to be destabilized due to being antiaromatic in a closed-shell singlet configuration by Hückel's 4*n* + 2 equation, could be described as gaining aromatic stabilization when adopting a triplet configuration.^59^ The equation 4*n* can be used to predict if a system will exhibit aromatic stability while adopting a triplet configuration.

While there is wide agreement about the existence of aromaticity/antiaromaticity as a general principle, there is similarly wide disagreement on how to apply these terms to specific molecules and how to quantify the effects of aromaticity/antiaromaticity. Individual indices, such as bond length alternations, magnetic measurements (*e.g.* chemical shifts, magnetic susceptibility exultations, and nucleus independent chemical shifts (NICS)), molecular stability compared to acyclic systems, HOMO–LUMO separation, and reactivity, have all been applied as tests for aromaticity/antiaromaticity, but no single measurement has acquired consensus as a definitive indicator for all ring systems. Consequently, we examine aromaticity/antiaromaticity by looking at a combination of these measurements.

**Fig. 4:**
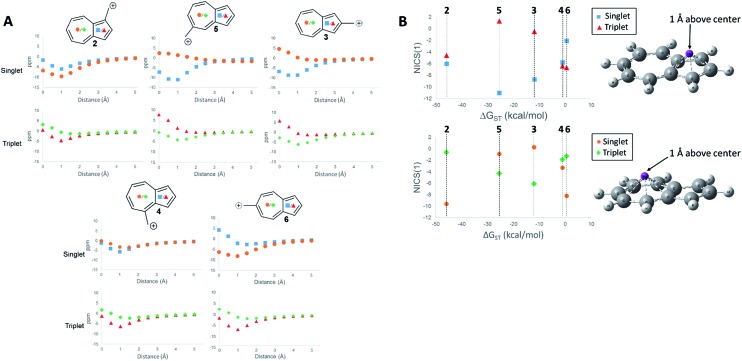
NICS values of the center of the 5-membered and 7-membered rings for structures **2–6** on the singlet and triplet surfaces. (A) NICS value *versus* distance above plane of ring. (B) NICS(1) value of **2–6** plotted against the Δ*G*_ST_ ((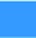) 5-membered ring singlet, (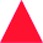) 5-membered ring triplet, (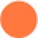) 7-membered ring singlet, (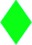) 7-membered ring triplet) GIAO-HF/6-31+G(d,p)//CBSB7.

First, we examined the NICS values for the ions,^60^,^61^ which calculates the NMR isotropic shielding tensor of a dummy atom placed at the center of the ring being examined. A positive NICS value suggests that the ring structure of interest is antiaromatic, while negative values suggest aromaticity. Schleyer, *et al.*, probed the aromaticity/antiaromaticity of the parent azulene structure by calculating the NICS values from the center of both rings, a precedent that will be followed in this investigation.^60^ Additionally, a method proposed by Stanger, plotting the NICS value starting from the center of the ring plane out to a distance of 5 Å, was utilized.^62^ When discussing specific NICS values, we use the value obtained when the dummy atom is 1 Å from the center of the ring, the NICS(1) value, which attempts to isolate the effects of the pi current from the sigma current effects. These plots are shown in Fig. 4.

The NICS values of the 5-membered and 7-membered rings of structures **2–6** on the singlet and triplet surfaces are plotted against the respective Δ*G*_ST_ (Fig. 4A). Plots of the NICS(1) against the Δ*G*_ST_ yield correlations between the aromaticity and the Δ*G*_ST_ (Fig. 4B). The NICS(1) values of the 5-membered ring in Fig. 4B, with the exception of ion **2** (discussed below), shows as the ring increases aromatic character in the singlet state, indicated by a decreasing NICS value, the triplet state shows an increasing NICS value due to larger antiaromatic character. This correlation in the change in aromaticity/antiaromaticity gives credibility to the notion that increasing destabilization due to antiaromaticity on the singlet state leads to a stabilization of the triplet state due to triplet state aromaticity. The inverse is also true. For instance, ions **4** and **6** have the highest-energy singlet states, and also have the smallest singlet state NICS(1) values in the 5-membered ring. These ions also have the most negative triplet NICS(1) values for the 5-membered ring. The trend is similar (although diminished in magnitude) in the 7 membered ring. With the exception of **2** mentioned earlier, ions **4** and **6** have the most negative NICS(1) value in the triplet state and the most positive NICS(1) of the singlet state.

The above-mentioned discrepancy of ion **2** not displaying the expected NICS(1) values that would suggest a large stabilization of the singlet state gained by singlet aromaticity and triplet destabilization due to triplet antiaromaticity shows the limits of the NICS method. While the ions **3–6** appear to have stabilities mostly driven by the aromaticity/antiaromaticity of the five-membered ring, here, the stability appears to be mostly driven by the seven membered ring, with a highly stabilized singlet state and a destabilized triplet state. Indeed, this ion has the most negative NICS(1) value for the singlet state of all the ions and the most positive value of the NICS(1) for the triplet state of all the ions.

**Fig. 5:**
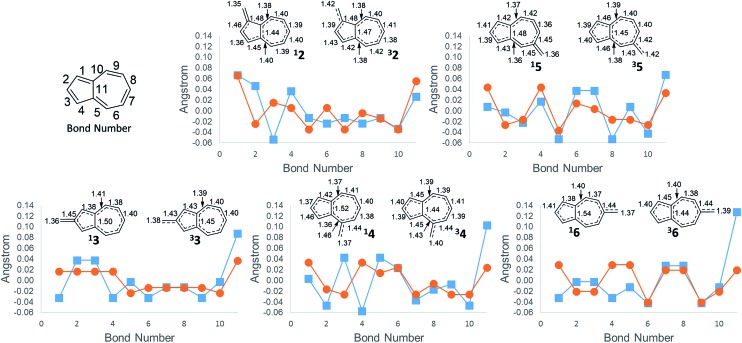
Difference from average bond length ((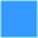) singlet, (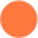) triplet). Bond lengths for singlet state (left structure) and triplet state (right structure). Arranged from most singlet favoured to least singlet favoured (CBS-QB3 geometries).

An additional way of identifying aromaticity of a system is by inspection of bond length alternation around the cycle. Highly bond-alternating structures (like singlet cyclobutadiene) suggest antiaromatic character, whereas bond length equalization (like benzene) suggest aromaticity. As shown in Fig. 5, as the Δ*G*_ST_ swings towards the triplet state, the bond alternation in the singlet state starts to increase, suggesting an increase in singlet antiaromaticity. Concomitantly, the bond alternation in the triplet state decreases until a more equal alternation pattern is achieved in structure **6**, suggesting that the triplet state achieves more aromatic character. These data are consistent with the NICS values that suggest that the destabilization of the singlet state is driven by antiaromaticity, coinciding with stabilization of the triplet driven by increased aromaticity.

The bond length of bond 11 (Fig. 5)—the bond shared by the two rings of azulene—also appears to be a diagnostic indicator of the aromaticity/antiaromaticity of the system. As the absolute energy of the state increases by differing substitution patterns, the length of this bridge bond increases for the singlet state. This increasing bond length indicates a change from a totally conjugated bicyclic system to, in effect, the conjugation occurring in a monocycle spanning the outermost ring of the bicyclic system. For example, in **2**, this bond length on the singlet surface is 1.44 Å, about the typical length of an aromatic C–C bond. For **6**, this bond length is 1.54 Å, the length of a typical C–C single bond, suggesting that conjugation across this bond is minimal. The triplet bridge bond length changes very little compared to the singlet length, implying that the triplet state is less sensitive to changes in aromaticity than the singlet state. This insensitivity of the triplet state seems to be born out in the absolute energies for ions **2–6**, which show drastically altered singlet energies, but more minimally altered triplet energies.

### Substitution effects and linear free-energy relationships

**Fig. 6:**
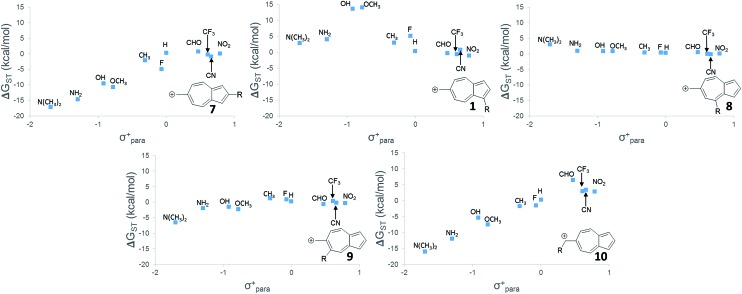
Linear free-energy relationships of structures **1**, **7–10**. UB3LYP/6-31+G(d,p).

While **6** is fundamentally interesting as a purely hydrocarbon ground state triplet ion, we wondered how the singlet–triplet splittings of these carbocations would be effected by ring substitutions. Appending substituents to structure **6** were carried out to create linear free-energy relationship (LFER) plots of the Δ*G*_ST_*vs.* the *σ*^+^ Hammett substituent parameter,^63^ shown in Fig. 6.

The LFER plots in Fig. 6 reveal obvious trends. Varying substituents of structure **1** leads to ions with the largest Δ*G*_ST_ in favor of the triplet state (by 14.0 kcal mol^–1^, with the substitution of the methoxy group). The dominant trend from the LFER plots are that any time a donor can be attached in a way that can directly stabilize the cation through a resonance structure, the singlet state is stabilized. However, increasing the strength of the withdrawing group does little to further enhance the Δ*G*_ST_ in favor of the triplet. Additionally, substituent effects are minimal when the substituent is not directly conjugated to the cation center, or require a canonical structure featuring a double bond at bond 11 (Fig. 5) to achieve conjugation to the carbenium center. In these cases, such as in ions **1** and **9**, the slope of the plot is nearly flat. When the substituent is in direct conjugation with the carbenium center, such as in ions **7**, **9** and **10**, the slope is large and positive.

On a technical note, for the methoxy and hydroxy substituted ions **1**, the Δ*G*_ST_ appears to be outliers from the other data points in the plot, showing very large singlet–triplet splittings in favor of the triplet. Closer analysis shows that these two extreme values are artifacts from the UB3LYP singlet optimization, which is highly spin contaminated, leading to poor singlet geometries that are effectively triplet diradical-like geometries. Calculations of the singlet state by CASSCF show that the singlet states are predominantly single reference, justifying the use of a restricted approach. The restricted optimization leads to Δ*G*_ST_ in line with the CASPT2 and CBS values in the plot, ∼+8 kcal mol^–1^ in favor of the triplet state. Note that these large Δ*G*_ST_ values are outside the anticipated error of the three methods: these computations predict these ions to have triplet ground states.

### Substituent effects are non-additive

**Fig. 7:**
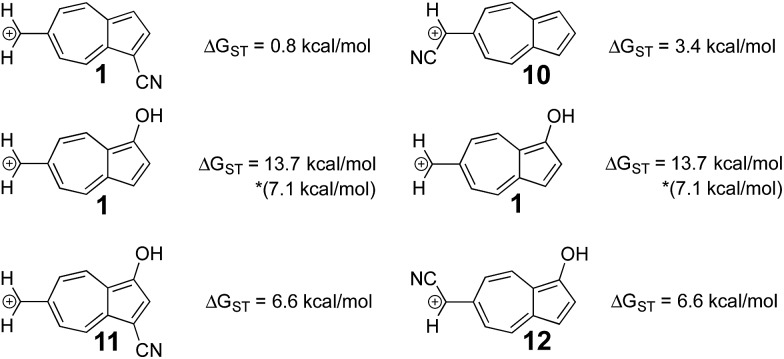
Using the optimal substitution pattern from the LFERs to produce a captodative effect. (UB3LYP/6-31+G(d,p)) *Δ*G*_ST_ using the uncorrected singlet energy.

In a previously published work by our lab,^25^ it was investigated if adding multiple electron donating substituents that favor the triplet state to create a polysubstituted ion would lead to an even more favorable triplet state. Here, a more favorable triplet state was not observed for combinations of substituents, and instead favored the triplet to the same degree as the monosubstituted ion. From the LFER plots in Fig. 6, we noticed that in some substitution locations on azulene, donor substituents (like OH) favored the triplet state compared to the unsubstituted ion, while in other positions, electron withdrawing substituents favored the triplet state over the unsubstituted ion. We considered the possibility that a captodative effect might have an additive effect. Thus, we computed the Δ*G*_ST_ values in new systems in which both the electron withdrawing and electron donating group on structure **6** that individually gave the most triplet favored ground state were appended together. By adding both of these substituents to structure **6**, captodative structures were created to give ions **11** and **12**, shown in Fig. 7. These cases demonstrate that the substituent effects are non-additive, as they generate triplet ions with singlet–triplet splittings that are no larger than the mono-substituted derivatives.

### Hückel/Baird aromaticity rules lead to predictive general principles using simple canonical structures

**Fig. 8:**
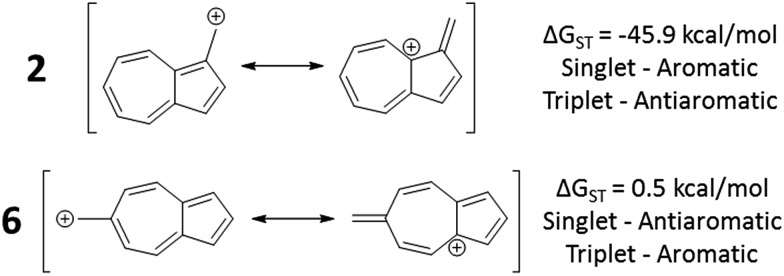
Canonical structures of **2** (top), having tropylium cation-like canonical structure and **6** (bottom), having cyclopentadienyl cation-like canonical structure.

To test the correlation of singlet and triplet state aromaticity and antiaromaticity to the Δ*G*_ST_, structures **2** (the most singlet favored of the core azulenyl carbocations) and **6** (the triplet favored azulene-carbocation) were analyzed. If all of the canonical resonance structures are drawn out for each structure, a pattern can be seen. With the canonical resonance structures of **2**, the majority feature rings with Hückel singlet state aromaticity (*e.g.* tropylium ion-like rings) and triplet state antiaromaticity, aligning with the computationally predicted singlet ground state. The canonical resonance structures of **6**, on the other hand, exhibit antiaromaticity on the singlet surface (*e.g.* cyclopentadienyl-like rings), but aromaticity on the triplet surface, predicting the computationally favored triplet state. Fig. 8 depicts example canonical structures for structures **2** and **6** that illustrate this point.

### A simple canonical structure model predicts additional carbocations with low-energy diradical states

**Fig. 9:**
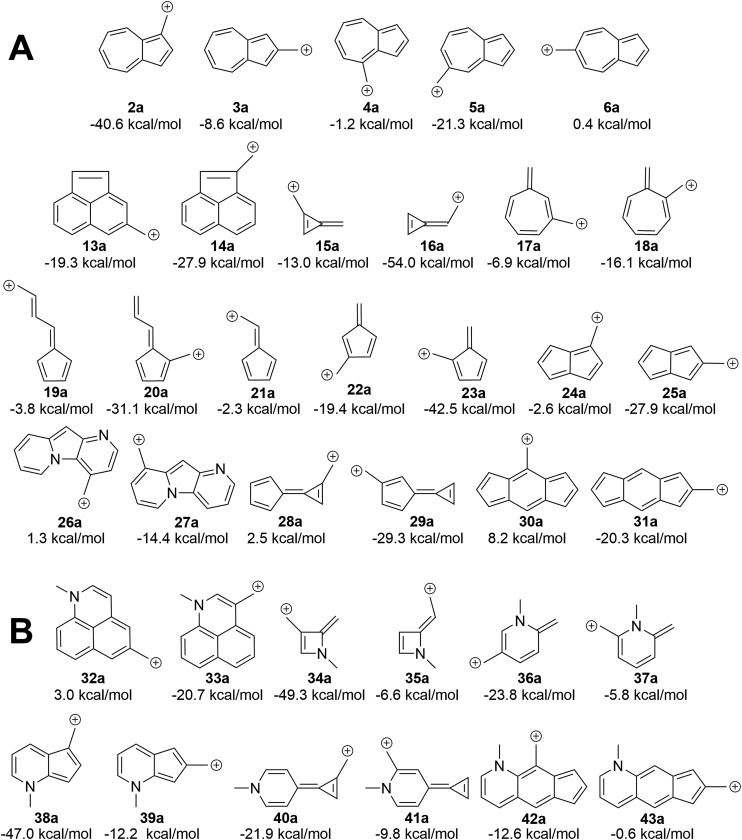
(A) Δ*G*_ST_ of additional ions. Ions with formally Hückel antiaromatic/Baird aromatic canonical structures have low-lying diradical states. (B) Manipulation of the Δ*G*_ST_ through the introduction of a 3° amine leads to a change in the favored spin state. UB3LYP/6-31+G(d,p).

We asked the question if azulene was special or just a member of a class of ions featuring low-energy diradical states. Since we found that the switch to a triplet ground state ion occurred due to increased antiaromaticity in the singlet state and increased aromaticity in the triplet state, structures of potentially triplet favored molecules could be more readily predicted and subsequently tested (Fig. 9A). To identify novel structures, we sought ions possessing canonical structures that satisfied the Hückel antiaromatic/Baird aromatic 4*n* equation.

Multi-ringed systems were also utilized to increase the size of the system that would be affected by the changes in aromaticity, but also staying within an atom count reasonable for our theoretical method. As can be seen in Fig. 9A, we were able to propose structures that have computed triplet ground state configurations. Structures **26a** and **28a** are predicted to have triplet ground states by 1.2 and 2.5 kcal mol^–1^ respectively. Intriguingly, structure **30a** has a computed Δ*G*_ST_ of +8.2 kcal mol^–1^. This represents the largest Δ*G*_ST_ for an ion of this type. Although this canonical structure method cannot guarantee that a proposed ion will have a triplet ground state, it successfully predicts whether an ion will have a low-lying (and potentially ground state) diradical state.

### Addition of two electrons to the π system predictably changes the aromaticity rules

Using a more general understanding of how Hückel's and Baird's rules of aromaticity govern the ground state of ions, we questioned how these aromaticity rules could be further used to manipulate structures to obtain a given ground state multiplicity. We asked whether we could take a structure that exhibited a singlet ground state and manipulate it in such a way to then favor a triplet ground state. We hypothesized that adding two additional electrons to a π system should change the favorability from the singlet state to the triplet state.

An easy way of adding two additional π electrons to the system is to introduce heteroatoms bearing a lone pair (in this study, a methylamino group) within the cyclic system of a singlet favoring structure. The same canonical structure method discussed previously was utilized to select the optimal site for introduction of the amino group, yielding the structures in Fig. 9B. By introducing the amino group into the system at predetermined positions, a structure that showed a singlet ground state prior to insertion, exhibited a low-lying diradical state with the added heteroatom. A similar outcome was also seen for structures that displayed a low-lying or ground state triplet, such that addition of the amino group transitioned the structure to favor the singlet state. The most favorable triplet produced by this addition of an amino group can be seen when the amino group is introduced within **13a**, having a singlet favored configuration by 19.3 kcal mol^–1^, yielding **32a** with the triplet state being favored by 3.0 kcal mol^–1^. Other structures of note are **16a**, singlet favored by 54.0 kcal mol^–1^, to **35a**, now only singlet favored by 6.6 kcal mol^–1^, and **31a** transitioning from singlet favored by 20.3 kcal mol^–1^, to only 0.6 kcal mol^–1^ with introduction of the amino group to yield structure **43a**.

### Replacing the carbocation with a carbanion similarly changes the aromaticity rules

**Fig. 10:**
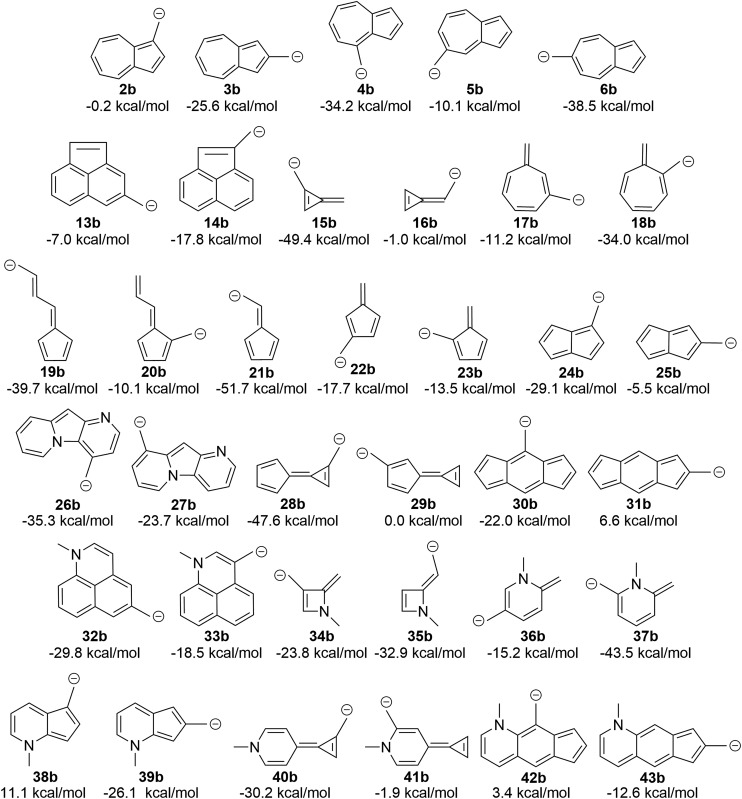
Transition of the carbocation to a carbanion inverts the favored state, making singlet-favored molecules adopt a low-lying diradical state and *vice versa* (UB3LYP/6-31+G(d,p)).

The attentive reader might also wonder if these effects could be reversed. That is, could one make the benzyl anions and reverse the connectivity rules described above (since one is adding two electrons to the system) and identify anions with low-energy triplet states? This appears to be the case (Fig. 10). Indeed, changing from the carbocation to the carbanion manipulates the aromaticity in such a fashion as to change the preferred electronic state. It is intriguing to observe that if one arranges the azulenyl cations in order from most to least singlet favored (**2a**, **5a**, **3a**, **4a**, **6a**), the anions are then listed in the exact order of least singlet favored to most singlet favored. Structures **42b**, **31b**, and **38b** show notable favorability of the diradical state by 3.4 kcal mol^–1^, 6.6 kcal mol^–1^, and 11.1 kcal mol^–1^, respectively. The ability to further manipulate the π system to select the aromaticity rules that govern the molecule further opens the possibilities to discovering more molecules that exhibit a triplet ground state.

## Conclusions

Certain azulenyl cations are predicted to have triplet ground states, a surprise considering that normal benzylic cations have singlet ground states with very large energy gaps to an excited triplet state. By substituting electron donating or withdrawing groups on the azulene, the triplet configuration could be favored even further, although substituent effects appear to be non-additive. Aromaticity/antiaromaticity arguments of the singlet and triplet state explain the remarkable ground triplet state for these ions. By employing Hückel's 4*n* + 2 equation for singlet state aromaticity and Baird's 4*n* for triplet state aromaticity, the Δ*G*_ST_ of structures **2–6** can be explained such that the canonical structures of structure **2** follow Hückel's equation for singlet state aromaticity, leading to a lower energy singlet state, while structure **6** follows Baird's equation for triplet aromaticity, leading to a lower energy triplet state. The ability to apply these rules allowed for the prediction of other structures that computationally favor the triplet state over the singlet state. Structures **26a** and **28a** are predicted to have triplet ground states by 1.2 and 2.5 kcal mol^–1^ respectively, but structure **30a**, with a Δ*G*_ST_ of 8.2 kcal mol^–1^, is, to our knowledge, the most triplet favored hydrocarbon carbocation of this type.

Further utilization of the aromaticity rules lead to the introduction of a heteroatom in order to add two π electrons and change singlet favored structures into low-lying diradical or ground state triplet systems. Conversion of the carbocation to a carbanion as another pathway to introduction of additional π electrons also yielded conversion of singlet favoring structures into structures that exhibit low-lying diradical or triplet states, such as **42b**, **31b**, and **38b**, which favor a triplet ground state by 3.4, 6.6, and 11.1 kcal mol^–1^ respectively. Interestingly, upon broader examination of all of the structures presented here, each can be classified as a non-alternant hydrocarbon (with the exception of the heteroatom containing structures, as they are not hydrocarbons). This suggests that other non-alternant hydrocarbons will display similar properties as those discussed here. Surely, these structures are just examples of a broad class of ions that exhibit low-lying or triplet diradical ground states, inviting further theoretical and experimental investigations.

Our special interest in identifying carbocations that have low-lying diradical forms comes from their application to identifying new structures that can be used as photoremovable protecting groups (PPGs)—that is, structures that undergo solvolysis to form a carbenium ion pair upon exposure to light. The core idea is that carbocations with nearly degenerate closed-shell and diradical states have a nearby productive conical intersection that can channel the excited state to the ion pair. We used this principle to identify BODIPY-derived photocages, which have applications as PPGs cleaved using green light.^64^ To this end, we note that azulene is a remarkable structure in that it is an exceedingly compact chromophore that absorbs red light, making minimally-perturbing PPGs based on this group of potential interest. More generally, this study strikes another blow to the textbook paradigm of the carbocation being a closed-shell singlet structure. Typically, the cyclopentadienyl cation is taught as the exception to this rule, but the exceptions continue to mount.

## Supplementary Material

Supplementary information
