# Supplementary material for: Anomalous effect of non-alternant hydrocarbons on carbocation and carbanion electronic configurations
Source: Chem Sci. 2017 May 4;8(6):4231–41. doi: 10.1039/c7sc01047h (PMC5902794; doi:10.1039/c7sc01047h)
Supplement: Supplementary file 1 [file SC-008-C7SC01047H-s001.pdf]

Electronic Supplementary Material (ESI) for Chemical Science.
This journal is © The Royal Society of Chemistry 2017

Supplementary Information

Anomalous Effect of Non-Alternant Hydrocarbons on Carbocation and Carbanion Electronic Configurations

Logan J. Fischer, Andrew S. Dutton, Arthur H. Winter*

*Iowa State University, Department of Chemistry, 1608 Gilman Hall, Ames, Iowa, 50011, United States of America.
E-mail: winter@iastate.edu

Contents

S2	Experimental vs. Theoretical Values Plot
S3	Nucleus Independent Chemical Shift values of Compounds **2-6**
S4	Linear Free Energy Relationships of Compounds **1**, **7-10**
S5-6	Values used to create Linear Free Energy Relationships of Compounds **1**, **7-10**
S7	Linear Free Energy Relationships of Compound **4**
S8-319	Cartesian Coordinates, Energies, and CASPT2 Occupation for Structures and CASPT2 Active Space Orbitals for Experimental Compounds

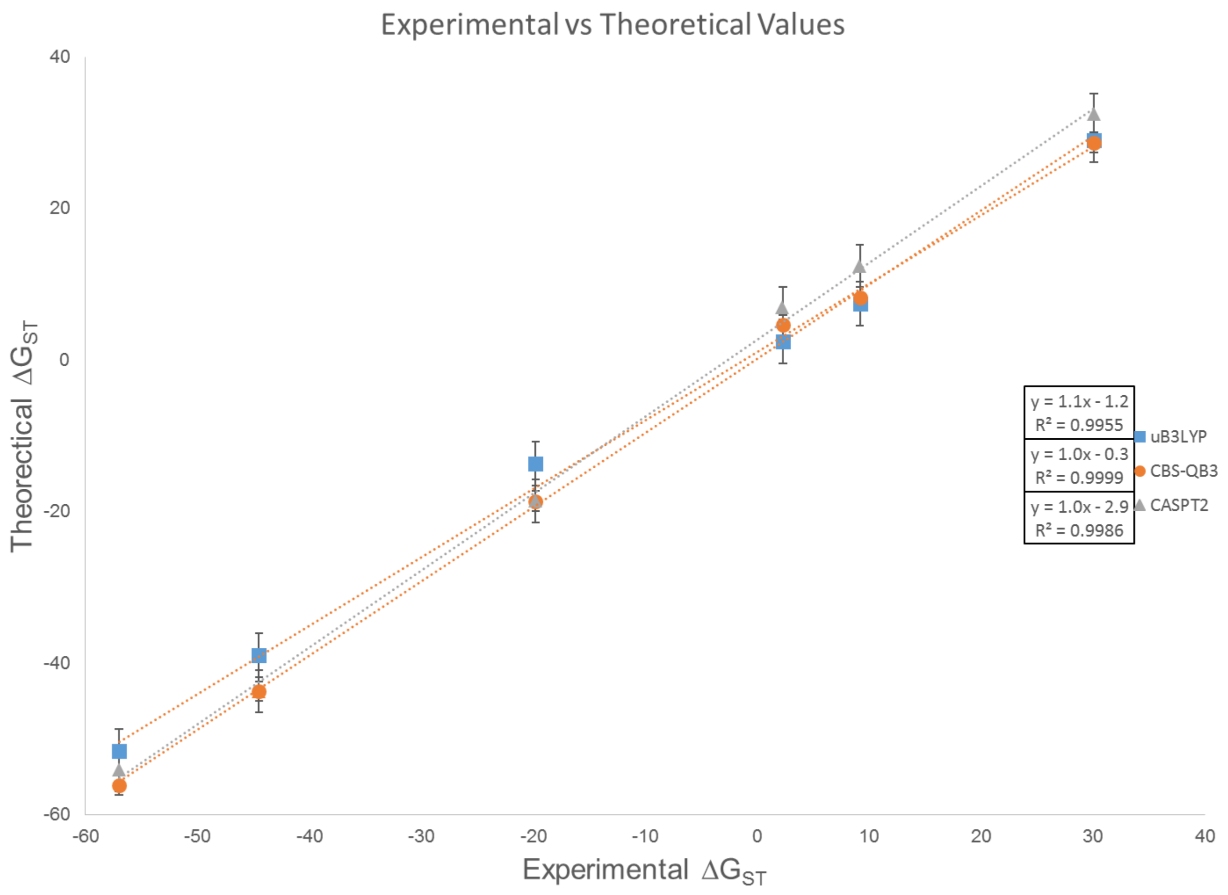

Figure S1. Singlet-triplet gap experimental values plotted against theoretical values. ■ UB3LYP/6-31+G(d,p), ● CBS-QB3, ▲ CASPT2/ANO-L-VTZP//CBSB7

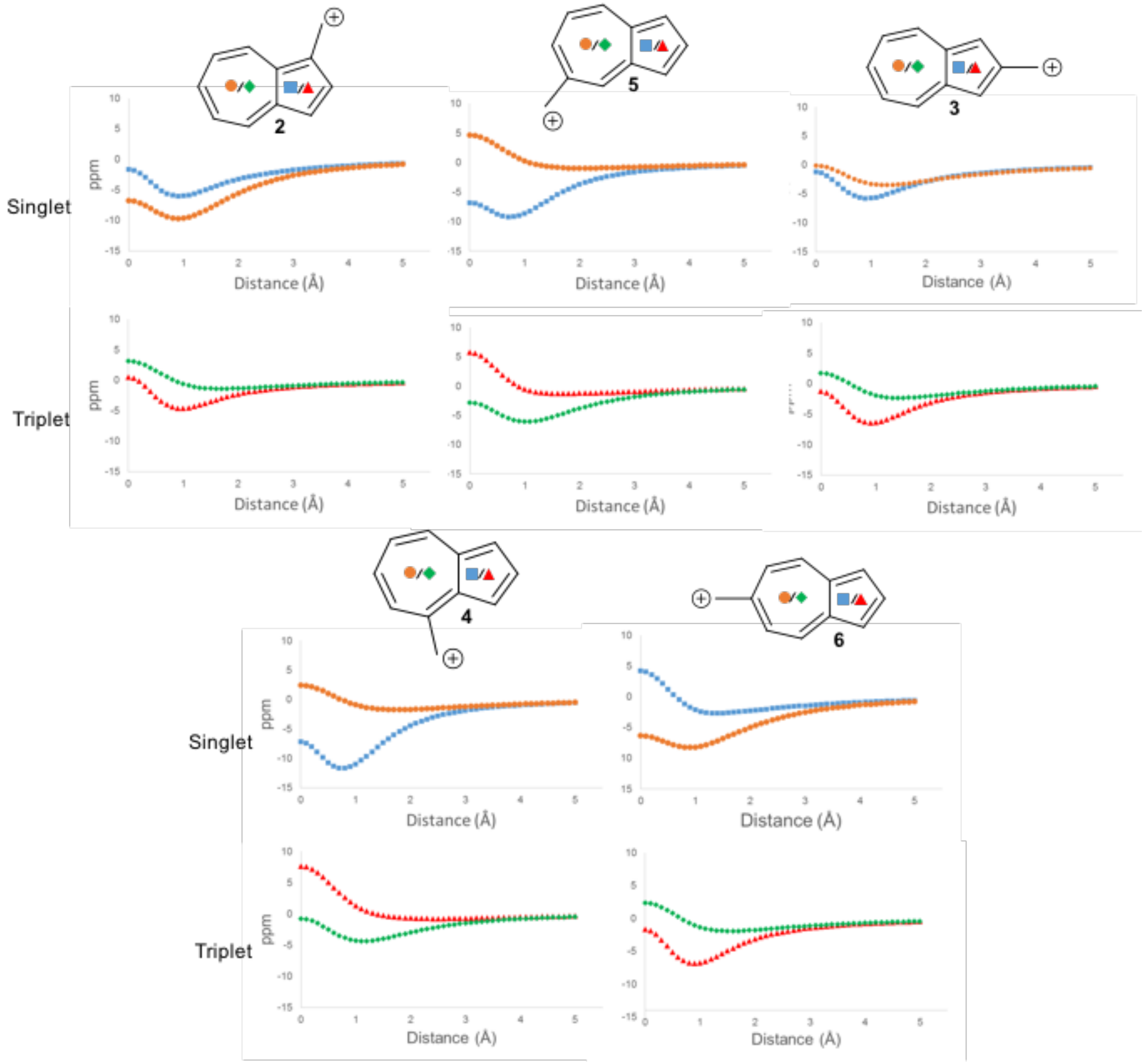

Figure S2. NICS values from 0 Å to 5 Å, taken every 0.1 Å.

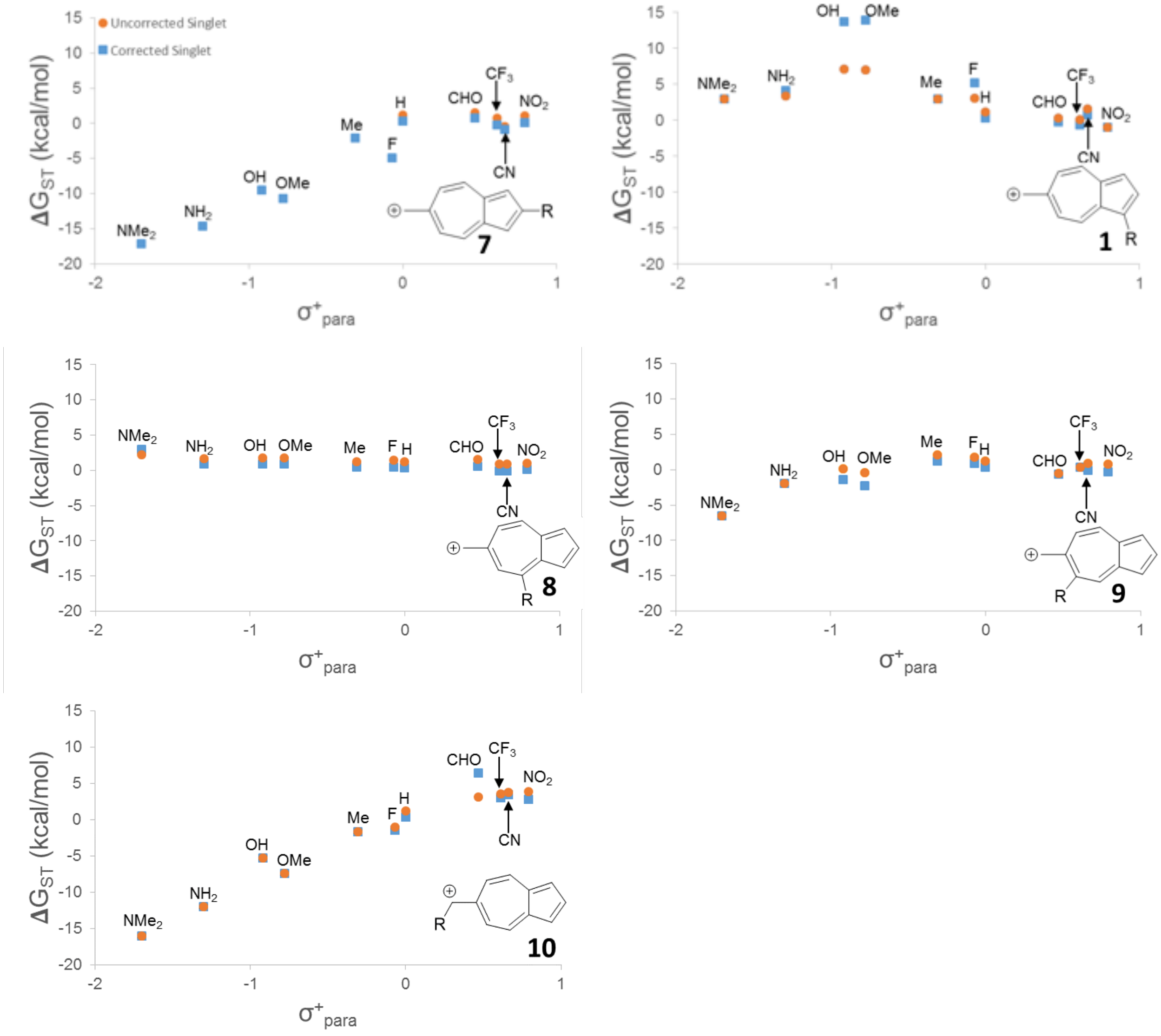

Figure S3. Liner free energy relationships of the singlet-triplet gap of compound **1**, **7**-**10** using the spin-contamination corrected and non-spin-contamination corrected singlet energies.

Table 1. Computed ΔG_{ST} (kcal/mol) and Singlet Spin Contamination from Substituted Azulenyl Cation Analogues

		ΔG_{ST}		$\langle S^2 \rangle$
Compound	R	Uncorrected Singlet	Corrected Singlet	Uncorrected Singlet
**7**	$N(CH_3)_2$	-17.2	-17.2	0.0000
	NH_2	-14.7	-14.7	0.0000
	OH	-9.5	-9.5	0.0000
	OCH_3	-10.7	-10.7	0.0000
	CH_3	-2.1	-2.1	0.0000
	H	1.2	0.3	0.3084
	CHO	1.5	0.7	0.3651
	CF_3	0.8	-0.3	0.3085
	CN	-0.5	-0.9	0.1060
	NO_2	1.0	0.1	0.3586
**1**	$N(CH_3)_2$	3.0	3.0	0.7073
	NH_2	3.4	4.1	0.7987
	OH	7.1	13.7	0.9941
	OCH_3	7.0	14.0	1.0158
	CH_3	2.9	3.0	0.6373
	H	1.2	0.3	0.3084
	CHO	0.3	-0.3	0.1646
	CF_3	0.1	-0.6	0.1773
	CN	1.6	0.8	0.3625
	NO_2	-1.0	-1.0	0.0000
**8**	$N(CH_3)_2$	2.2	3.0	0.7970
	NH_2	1.6	1.0	0.7194
	OH	1.8	0.9	0.6055
	OCH_3	1.8	0.9	0.6081
	CH_3	1.3	0.5	0.2608
	H	1.2	0.3	0.3084

	CHO	1.5	0.6	0.3337
	CF_3	0.9	-0.1	0.2923
	CN	0.9	-0.1	0.3030
	NO_2	1.11	0.1	0.3185
**9**	$N(CH_3)_2$	-6.5	-6.5	0.0000
	NH_2	-1.9	-1.9	0.0000
	OH	0.1	-1.4	0.5408
	OCH_3	-0.4	-2.2	0.5463
	CH_3	2.1	1.3	0.4751
	H	1.2	0.3	0.3084
	CHO	-0.5	-0.6	0.0098
	CF_3	0.3	0.4	0.2986
	CN	0.9	-0.1	0.3847
	NO_2	0.7	-0.3	0.2401
**10**	$N(CH_3)_2$	-16.0	-16.0	0.0000
	NH_2	-12.0	-12.0	0.0000
	OH	-5.3	-5.3	0.0000
	OCH_3	-7.5	-7.5	0.0000
	CH_3	-1.7	-1.7	0.0000
	H	1.2	0.3	0.3084
	CHO	3.1	6.4	1.0441
	CF_3	3.6	3.0	0.5661
	CN	3.8	3.5	0.5625
	NO_2	3.9	2.8	0.5015

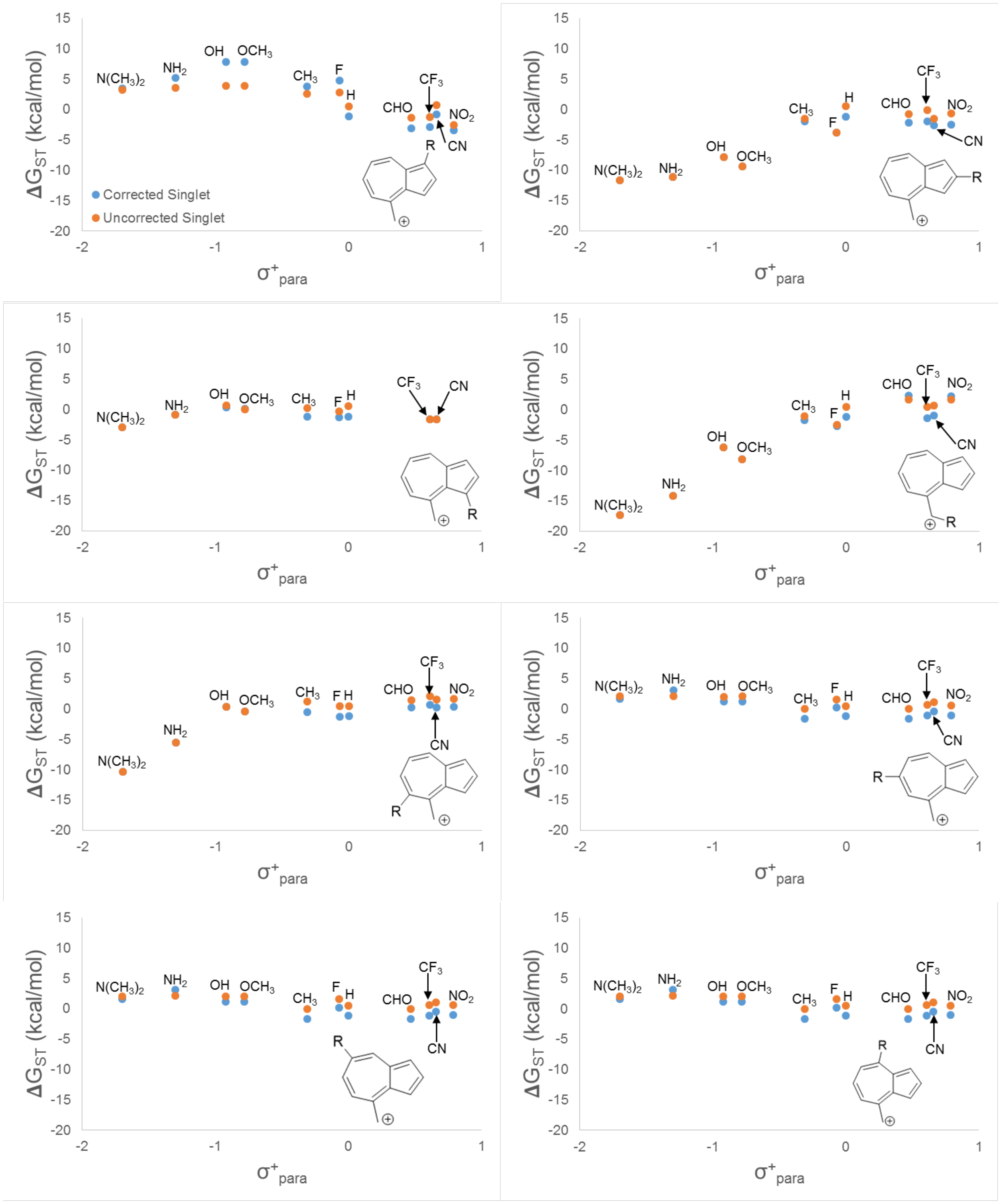

Figure S4. Liner free energy relationships of the singlet-triplet gap of compound **4** using the spin contamination corrected and non-spin-contamination-corrected singlet energies.

Computation Structure Geometries and Energies
*All energies are in absolute units (A.U., Hartrees). Orbital depictions are of the specified active space for CASPT2 calculations. Additional CASPT2 calculations were performed using an all π active space.

Methylene UB3LYP Singlet

Atomic Number	Atomic Type	Coordinates (Angstroms)		
		X	Y	Z
6	C	0.000000	0.000000	0.148631
1	H	0.000000	0.923133	-0.445892
1	H	0.000000	-0.923133	-0.445892

Uncorrected Sum of Electronic and Thermal Free Energies = -39.149928
Corrected Sum of Electronic and Thermal Free Energies = -39.147732

Methylene CBS-QB3 Singlet

Atomic Number	Atomic Type	Coordinates (Angstroms)		
		X	Y	Z
6	C	0.000000	0.000000	0.177302
1	H	0.000000	0.860324	-0.531905
1	H	0.000000	-0.860324	-0.531905

CBS-QB3 Free Energy = -39.075022
Thermal Correction to Gibbs Free Energy = -0.001215
CASPT2 Energy = -39.041155

Occupation				
**A1**	**A2**	**B2**	**B1**	**Coefficient**
2 2 0		2	0	0.973576
2 0 0		2	2	-0.213733
0 2 2		2	0	-0.061546

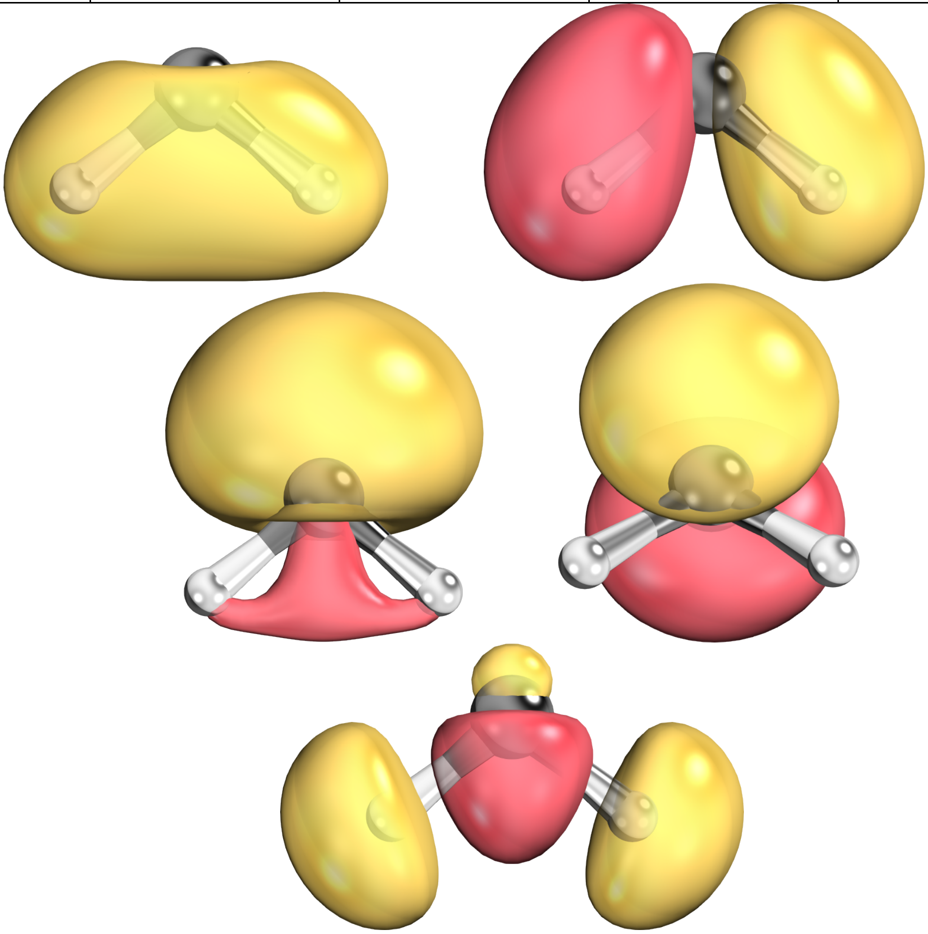

Methylene UB3LYP Triplet

Atomic Number	Atomic Type	Coordinates (Angstroms)		
		X	Y	Z
6	C	0.000000	0.000000	0.104021
1	H	0.000000	0.998623	-0.312064
1	H	0.000000	-0.998623	-0.312064

Sum of Electronic and Thermal Free Energies = -39.159601

Methylene CBS-QB3 Triplet

Atomic Number	Atomic Type	Coordinates (Angstroms)		
		X	Y	Z
6	C	0.000000	0.000000	0.103907
1	H	0.000000	0.996713	-0.311721
1	H	0.000000	-0.996713	-0.311721

CBS-QB3 Free Energy = -39.088193
Thermal Correction to Gibbs Free Energy = -0.001197
CASPT2 Energy = -39.060932

Occupation				
**A1**	**A2**	**B2**	**B1**	**Coefficient**
2 u 2		0	u	0.002998
2 u 0		2	u	0.993867
u u u		2	d	-0.061546

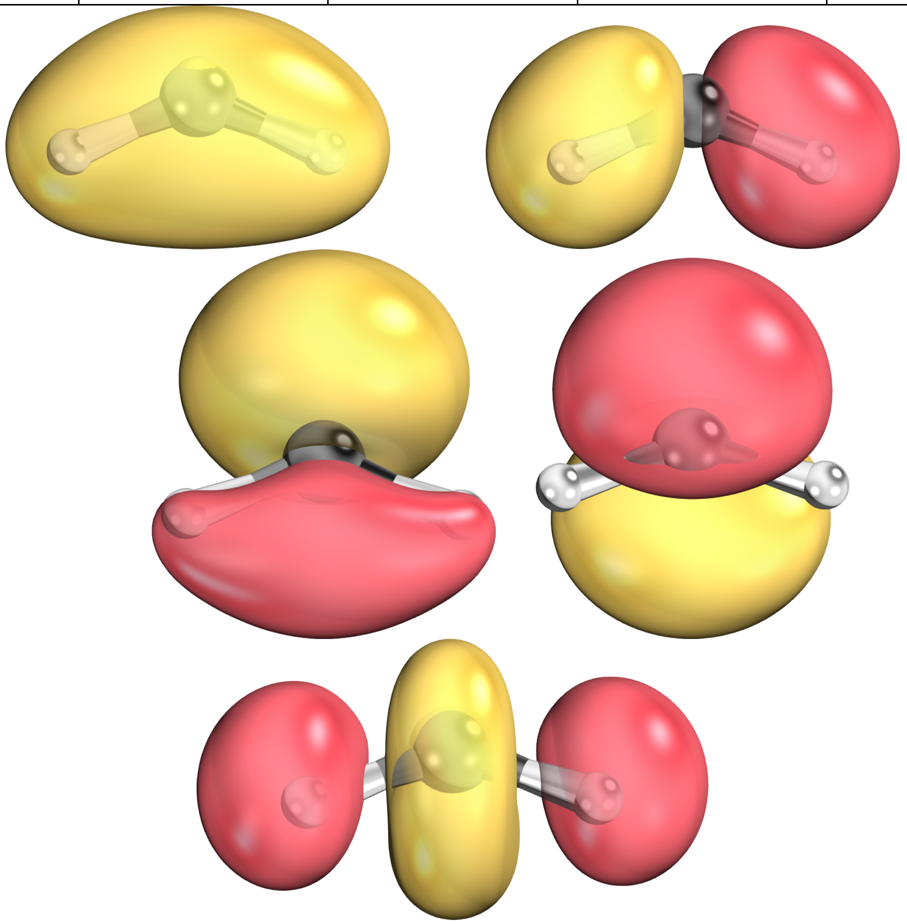

Amidogen UB3LYP Singlet

Atomic Number	Atomic Type	Coordinates (Angstroms)		
		X	Y	Z
7	N	0.000000	0.000000	0.079126
1	H	0.000000	0.979913	-0.276942
1	H	0.000000	-0.979913	-0.276942

Uncorrected Sum of Electronic and Thermal Free Energies = -55.446278
Corrected Sum of Electronic and Thermal Free Energies = -55.424926

Amidogen CBS-QB3 Singlet

Atomic Number	Atomic Type	Coordinates (Angstroms)		
		X	Y	Z
7	N	0.000000	0.000000	0.139699
1	H	0.000000	0.849254	-0.488946
1	H	0.000000	-0.849254	-0.488946

CBS-QB3 Free Energy = -55.352799
Thermal Correction to Gibbs Free Energy = -0.000047
CASPT2 Energy = -55.320396

**Occupation**				
**A1**	**A2**	**B2**	**B1**	**Coefficient**
2 2 0		2	0	0.951118
2 0 0		2	2	0.041630
0 2 2		2	0	0.003517

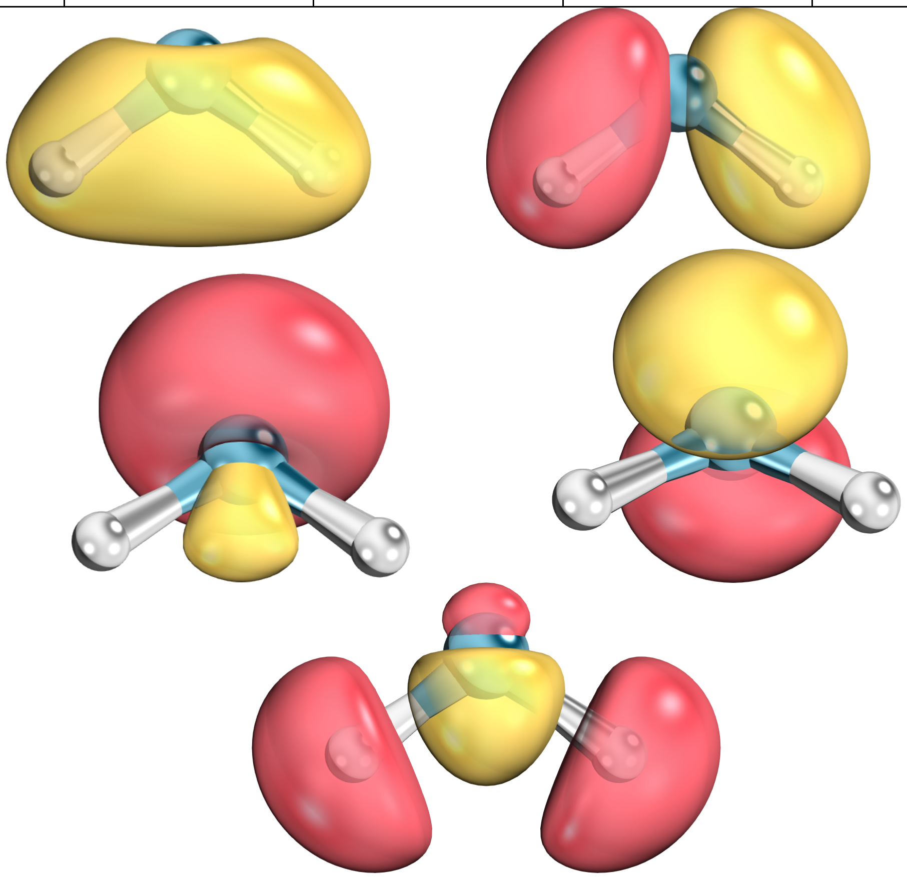

Amidogen UB3LYP Triplet

Atomic Number	Atomic Type	Coordinates (Angstroms)		
		X	Y	Z
7	N	0.000000	0.000000	0.050942
1	H	0.000000	1.013481	-0.178298
1	H	0.000000	-1.013481	-0.178298

Sum of Electronic and Thermal Free Energies = -55.471152

Amidogen CBS-QB3 Triplet

Atomic Number	Atomic Type	Coordinates (Angstroms)		
		X	Y	Z
7	N	0.000000	0.000000	0.050992
1	H	0.000000	1.012139	-0.178472
1	H	0.000000	-1.012139	-0.178472

CBS-QB3 Free Energy = -55.398640
Thermal Correction to Gibbs Free Energy = -0.001263
CASPT2 Energy = -55.372976

Occupation				
A1	A2	B2	B1	Coefficient
2 u 2		0	u	0.003060
2 u 0		2	u	0.985090
u d u		2	u	0.004570
u u u		2	d	0.004030

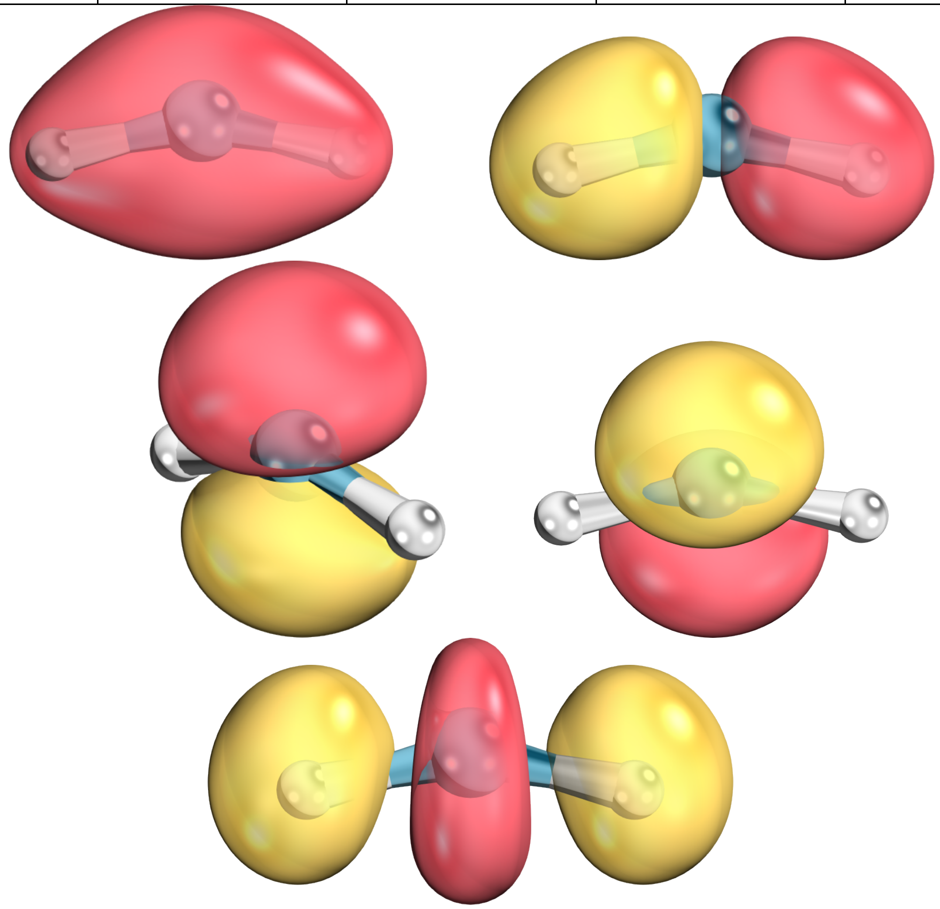

Difluoromethylene UB3LYP Singlet

Atomic Number	Atomic Type	Coordinates (Angstroms)		
		X	Y	Z
6	C	0.000000	0.000000	0.605686
9	F	0.000000	1.038096	-0.201895
9	F	0.000000	-1.038096	-0.201895

Uncorrected Sum of Electronic and Thermal Free Energies = -237.727998
Corrected Sum of Electronic and Thermal Free Energies = -237.727998

Difluorormethylene CBS-QB3 Singlet

Atomic Number	Atomic Type	Coordinates (Angstroms)		
		X	Y	Z
6	C	0.000000	0.000000	0.600408
9	F	0.000000	1.033467	-0.200136
9	F	0.000000	-1.033467	-0.200136

CBS-QB3 Free Energy = -237.500551
Thermal Correction to Gibbs Free Energy = -0.016551
CASPT2 Energy = -237.402675

Occupation				
**A1**	**A2**	**B2**	**B1**	**Coefficient**
2 2 0 0	2	2 0	2 0 0	-0.964919
2 2 0 0	2	u d	u d 0	0.003174
2 0 0 2	2	2 0	2 0 0	0.058492
u 2 u 0	2	d d	2 0 0	-0.057712
2 2 u 0	u	d 0	2 d 0	0.002994
2 0 0 0	2	2 0	2 2 0	0.142144

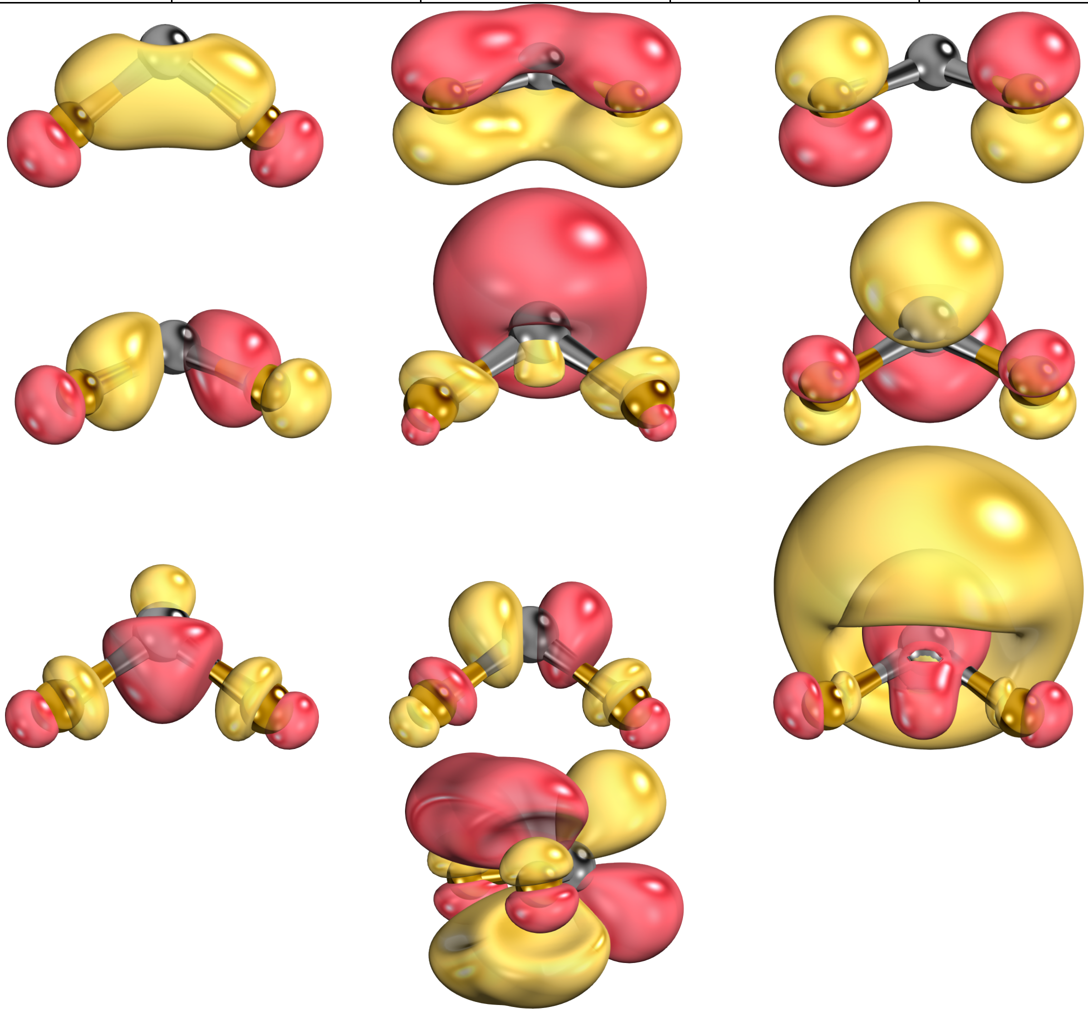

Difluorormethylene UB3LYP Triplet

Atomic Number	Atomic Type	Coordinates (Angstroms)		
		X	Y	Z
6	C	0.000000	0.000000	0.501805
9	F	0.000000	1.146508	-0.167268
9	F	0.000000	-1.146508	-0.167268

Sum of Electronic and Thermal Free Energies = -237.645801

Difluorormethylene CBS-QB3 Triplet

Atomic Number	Atomic Type	Coordinates (Angstroms)		
		X	Y	Z
6	C	0.000000	0.000000	0.497674
9	F	0.000000	1.142042	-0.165891
9	F	0.000000	-1.142042	-0.165891

CBS-QB3 Free Energy = -237.411175
Thermal Correction to Gibbs Free Energy = -0.017795
CASPT2 Energy = -237.315261

Occupation				
**A1**	**A2**	**B2**	**B1**	**Coefficient**
2 u 0 0	2	2 0	2 u 0	0.982692

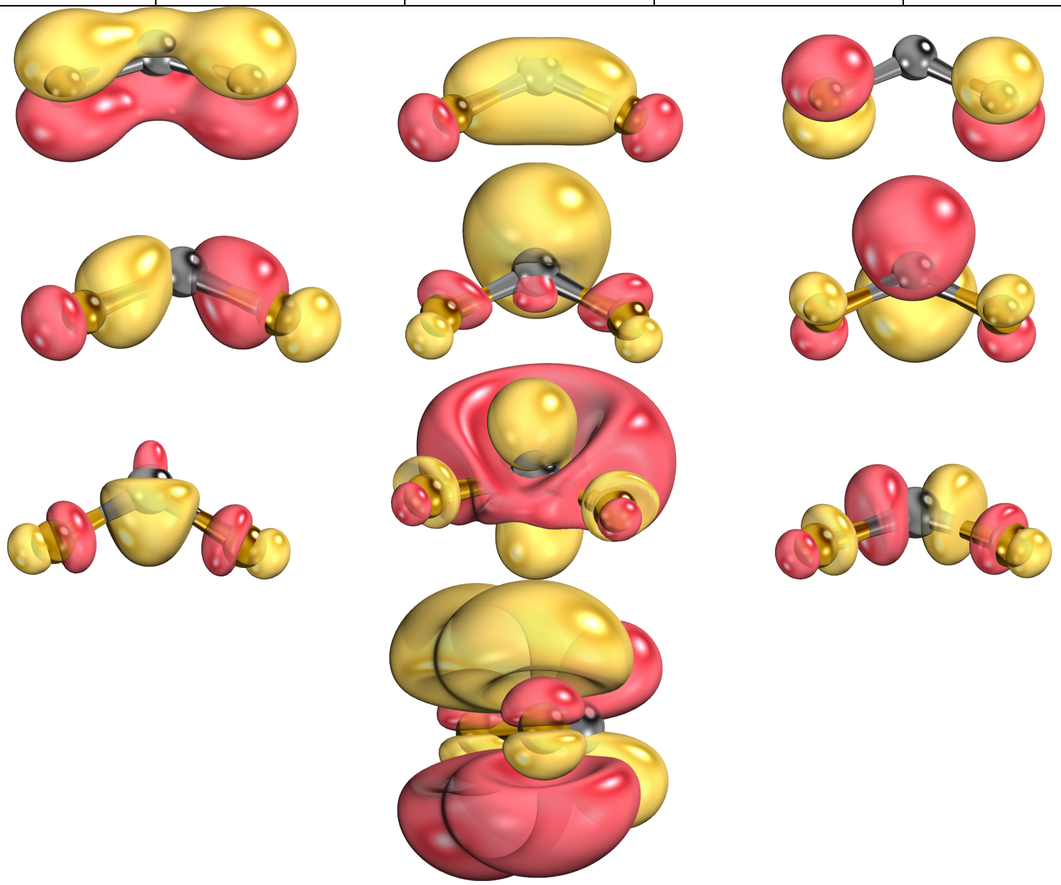

Phenylcarbene UB3LYP Singlet

Atomic Number	Atomic Type	Coordinates (Angstroms)		
		X	Y	Z
6	C	1.025597	1.247885	-0.000078
6	C	1.769454	0.059740	-0.000056
6	C	1.125274	-1.184745	-0.000014
6	C	-0.264162	-1.242303	0.000010
6	C	-1.048006	-0.056711	0.000045
6	C	-0.364059	1.191868	-0.000016
1	H	1.535686	2.206821	-0.000121
1	H	2.855057	0.105108	-0.000080
1	H	1.711817	-2.098649	-0.000003
1	H	-0.786622	-2.193863	0.000022
1	H	-0.947950	2.108449	-0.000015
6	C	-2.474925	-0.171556	0.000111
1	H	-2.983023	0.807076	0.000185

Uncorrected Sum of Electronic and Thermal Free Energies = -270.173758
Corrected Sum of Electronic and Thermal Free Energies = -270.175503

Phenylcarbene CBS-QB3 Singlet

Atomic Number	Atomic Type	Coordinates (Angstroms)		
		X	Y	Z
6	C	1.108313	-1.194352	0.000002
6	C	1.760684	0.040621	0.000005
6	C	1.038146	1.238899	0.000000
6	C	-0.346565	1.198172	-0.000002
6	C	-1.046114	-0.038905	0.000001
6	C	-0.278430	-1.231071	-0.000002
1	H	1.685032	-2.111803	0.000001
1	H	2.845260	0.072658	0.000004
1	H	1.563717	2.186927	-0.000002
1	H	-0.922233	2.117967	-0.000005
1	H	-0.823917	-2.167446	-0.000005
6	C	-2.478451	-0.172762	-0.000003
1	H	-2.893359	0.858084	-0.000003

CBS-QB3 Free Energy = -269.725588
Thermal Correction to Gibbs Free Energy = 0.072948
CASPT2 Energy = -269.607499

Occupation		
**A1**	**A2**	**Coefficient**
2	2 2 2 0 0 0 0	-0.924766
2	2 2 0 0 2 0 0	0.136808
2	2 u d u d 0 0	-0.095964
2	u 2 d 0 u 0 d	0.096123
2	2 0 2 2 0 0 0	0.133299
2	2 0 2 u 0 d 0	-0.060991
2	u d 2 u 0 0 d	-0.086700
0	2 2 2 2 0 0 0	0.064056
0	2 2 2 u 0 d 0	0.115937
0	2 2 2 0 0 2 0	0.101217
2	2 u u d d 0 0	-0.124184

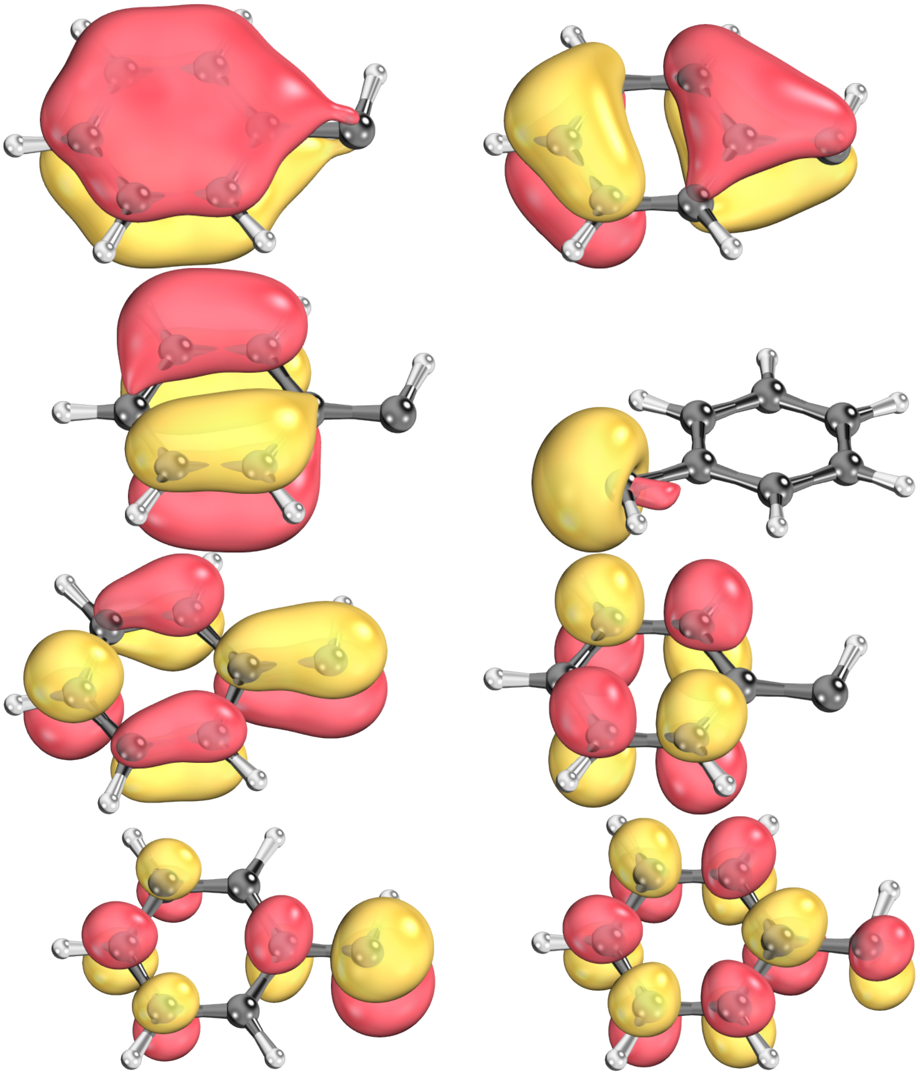

Phenylcarbene UB3LYP Triplet

Atomic Number	Atomic Type	Coordinates (Angstroms)		
		X	Y	Z
6	C	1.137153	-1.171441	0.000002
6	C	1.780083	0.076276	0.000002
6	C	1.012152	1.250755	0.000001
6	C	-0.374673	1.190199	-0.000001
6	C	-1.052533	-0.072404	-0.000001
6	C	-0.248034	-1.253875	0.000000
1	H	1.727552	-2.083422	0.000003
1	H	2.864255	0.132768	0.000004
1	H	1.504497	2.219393	0.000001
1	H	-0.961676	2.103894	-0.000002
1	H	-0.742115	-2.220413	0.000000
6	C	-2.444462	-0.141704	-0.000003
1	H	-3.250628	0.580940	-0.000004

Sum of Electronic and Thermal Free Energies = -270.179373

Phenylcarbene CBS-QB3 Triplet

Atomic Number	Atomic Type	Coordinates (Angstroms)		
		X	Y	Z
6	C	-1.133367	-1.168439	-0.000002
6	C	-1.774668	0.075551	-0.000002
6	C	-1.009454	1.247069	-0.000001
6	C	0.372573	1.187806	0.000001
6	C	1.050194	-0.071684	0.000001
6	C	0.247096	-1.250648	0.000000
1	H	-1.723094	-2.078554	-0.000002
1	H	-2.856828	0.131517	-0.000003
1	H	-1.502049	2.213410	-0.000001
1	H	0.958360	2.099915	0.000002
1	H	0.740904	-2.215104	0.000001
6	C	2.437318	-0.141075	0.000003
1	H	3.244551	0.577337	0.000005

CBS-QB3 Free Energy = -269.732846
Thermal Correction to Gibbs Free Energy = 0.071521
CASPT2 Energy = -269.617144

**Occupation**		
**A1**	**A2**	**Coefficient**
u	2 2 2 u 0 0 0	-0.919622
u	2 d 2 u 0 u 0	0.082883
u	2 2 u d u 0 0	-0.085375
u	2 2 u u d 0 0	-0.055665
u	2 2 u 0 u d 0	0.051700
u	2 u 2 d 0 u 0	0.140127
u	2 u 2 u 0 d 0	0.099961
u	u 2 2 d 0 0 u	-0.051146
u	2 2 0 u 2 0 0	-0.137842
u	2 d u u u d 0	0.056899
u	2 u d u d u 0	0.056096
u	u 2 d u d 0 u	-0.052730
u	u 2 d u u 0 d	-0.069180
u	2 0 2 u 0 2 0	-0.112532
u	u d 2 u 0 u d	0.058358
u	2 u u u d d 0	0.074713

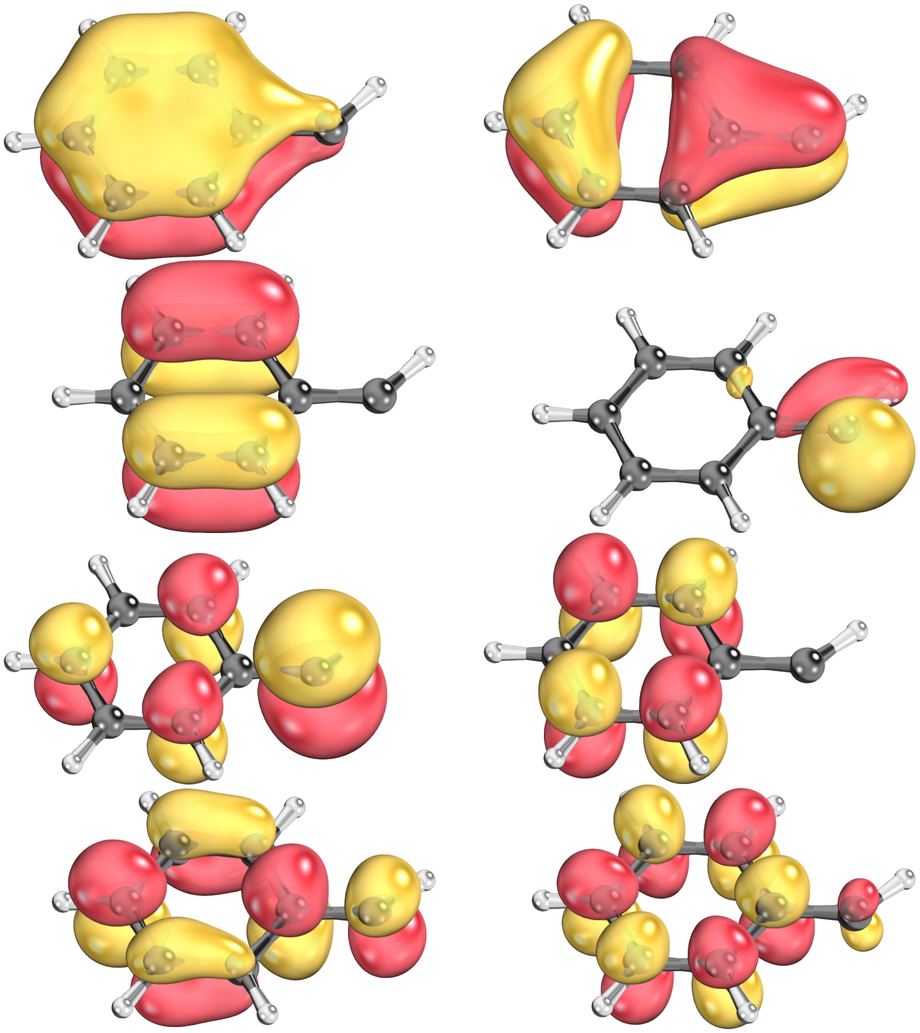

Phenyloxenium UB3LYP Singlet

Atomic Number	Atomic Type	Coordinates (Angstroms)		
		X	Y	Z
6	C	0.000000	1.259543	-1.079057
6	C	0.000000	0.000000	-1.747216
6	C	0.000000	-1.259543	-1.079057
6	C	0.000000	-1.286214	0.285383
6	C	0.000000	0.000000	1.048620
6	C	0.000000	1.286214	0.285383
1	H	0.000000	2.172684	-1.665454
1	H	0.000000	0.000000	-2.835885
1	H	0.000000	-2.172684	-1.665454
1	H	0.000000	-2.203389	0.868153
1	H	0.000000	2.203389	0.868153
8	O	0.000000	0.000000	2.268269

Uncorrected Sum of Electronic and Thermal Free Energies = -306.470408
Corrected Sum of Electronic and Thermal Free Energies = -306.470408

Phenyloxenium CBS-QB3 Singlet

Atomic Number	Atomic Type	Coordinates (Angstroms)		
		X	Y	Z
6	C	1.076296	1.256858	0.000000
6	C	1.742307	0.000000	0.000000
6	C	1.076296	-1.256858	0.000000
6	C	-0.283506	-1.283168	0.000000
6	C	-1.049826	0.000000	0.000000
6	C	-0.283506	1.283168	0.000000
1	H	1.662498	2.167649	0.000000
1	H	2.829118	0.000000	0.000000
1	H	1.662498	-2.167649	0.000000
1	H	-0.865392	-2.198543	0.000000
1	H	-0.865392	2.198543	0.000000
8	O	-2.261461	0.000000	0.000000

CBS-QB3 Free Energy = -306.011908
Thermal Correction to Gibbs Free Energy = 0.062578
CASPT2 Energy = -305.884346

Occupation				
**A1**	**A2**	**B2**	**B1**	**Coefficient**
	2 2	2 2 2	0 2 0 0 0	0.050863
	2 0	2 2 2	2 2 0 0 0	-0.927066
	2 0	2 2 2	2 0 2 0 0	0.193356
	2 0	2 2 2	0 2 2 0 0	0.054065
	2 0	2 2 2	2 0 u d 0	0.081052
	2 0	2 2 2	u d u d 0	0.096955
	u d	2 2 2	u 2 d 0 0	0.087523
	0 2	2 2 2	2 2 0 0 0	0.104410
	u u	2 2 2	d 2 d 0 0	-0.089813
	u u	2 2 2	d 2 0 d 0	0.059716
	u u	2 2 2	d 2 0 0 d	-0.057788
	0 0	2 2 2	2 2 2 0 0	0.076470
	0 0	2 2 2	2 2 u d 0	-0.089529
	0 0	2 2 2	2 2 0 2 0	0.056802

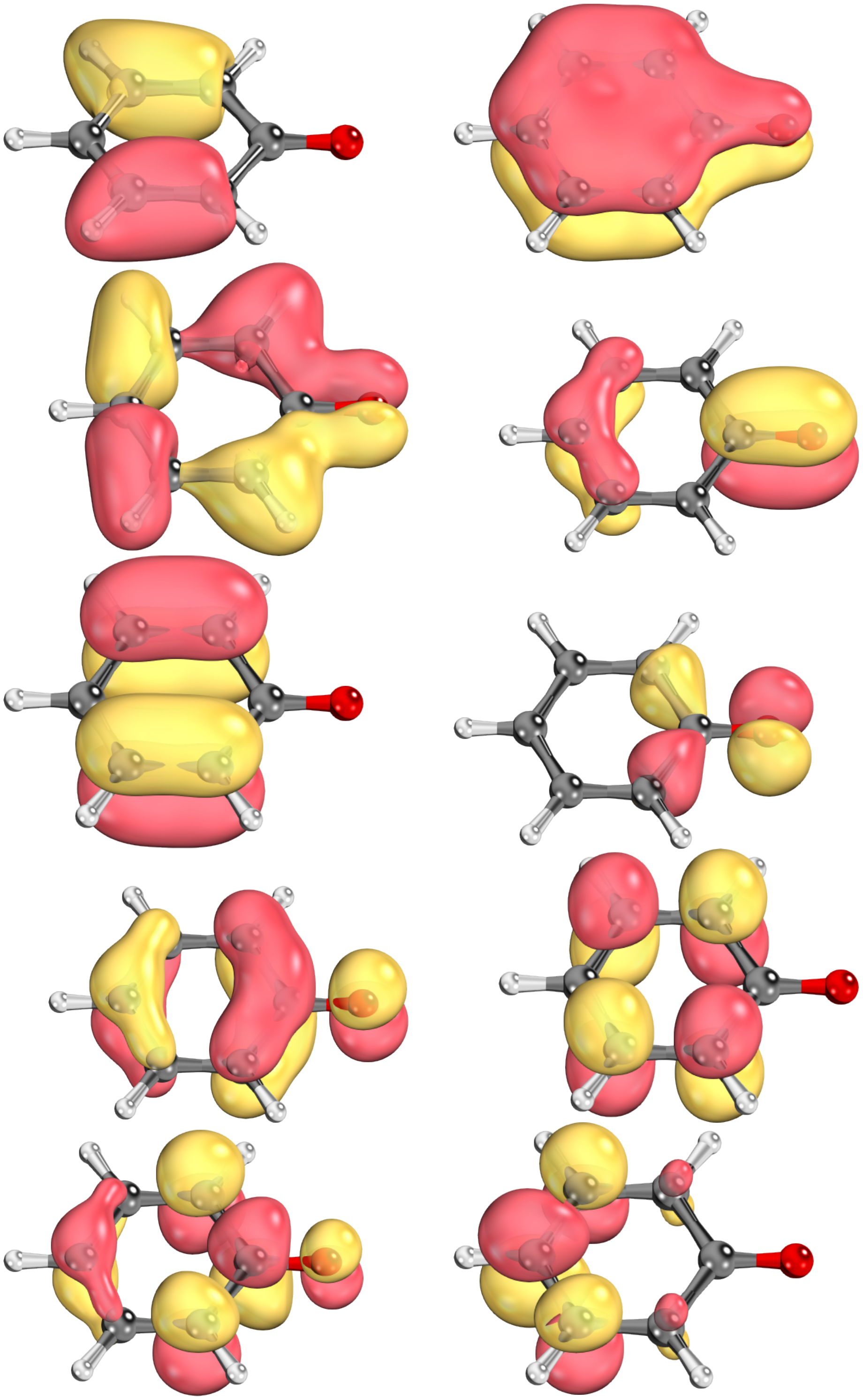

Phenyloxenium UB3LYP Triplet

Atomic Number	Atomic Type	Coordinates (Angstroms)		
		X	Y	Z
6	C	0.000000	1.233545	-1.071516
6	C	0.000000	0.000000	-1.772303
6	C	0.000000	-1.233545	-1.071516
6	C	0.000000	-1.270989	0.298165
6	C	0.000000	0.000000	1.011198
6	C	0.000000	1.270989	0.298165
1	H	0.000000	2.168683	-1.624567
1	H	0.000000	0.000000	-2.857636
1	H	0.000000	-2.168683	-1.624567
1	H	0.000000	-2.186404	0.878097
1	H	0.000000	2.186404	0.878097
8	O	0.000000	0.000000	2.274678

Sum of Electronic and Thermal Free Energies = -306.448697

Phenyloxenium CBS-QB3 Triplet

Atomic Number	Atomic Type	Coordinates (Angstroms)		
		X	Y	Z
6	C	0.000000	1.230122	-1.068767
6	C	0.000000	0.000000	-1.767873
6	C	0.000000	-1.230122	-1.068767
6	C	0.000000	-1.268039	0.296199
6	C	0.000000	0.000000	1.013082
6	C	0.000000	1.268039	0.296199
1	H	0.000000	2.163656	-1.620785
1	H	0.000000	0.000000	-2.851144
1	H	0.000000	-2.163656	-1.620785
1	H	0.000000	-2.181498	0.875449
1	H	0.000000	2.181498	0.875449
8	O	0.000000	0.000000	2.267672

CBS-QB3 Free Energy = -305.982307
Thermal Correction to Gibbs Free Energy = 0.060342
CASPT2 Energy = -305.852540

Occupation				
**A1**	**A2**	**B2**	**B1**	**Coefficient**
	2 2	2 u 2	2 0 u 0 0	0.051328
	2 0	2 2 u	u 2 2 0 0	-0.058704
	2 0	2 u 2	2 2 u 0 0	0.935521
	2 0	2 u 2	2 d u u 0	-0.055503
	2 0	2 u 2	2 u d u 0	-0.111407
	2 0	2 u 2	2 u u d 0	-0.072836
	2 0	2 u 2	2 0 u 2 0	0.061022
	u d	2 u 2	2 u 2 0 0	-0.076658
	0 2	2 u 2	2 2 u 0 0	-0.124482
	u u	2 d 2	2 2 u 0 0	-0.052877
	u u	2 u 2	2 2 d 0 0	0.115728
	u u	2 u 2	2 d u d 0	-0.071705

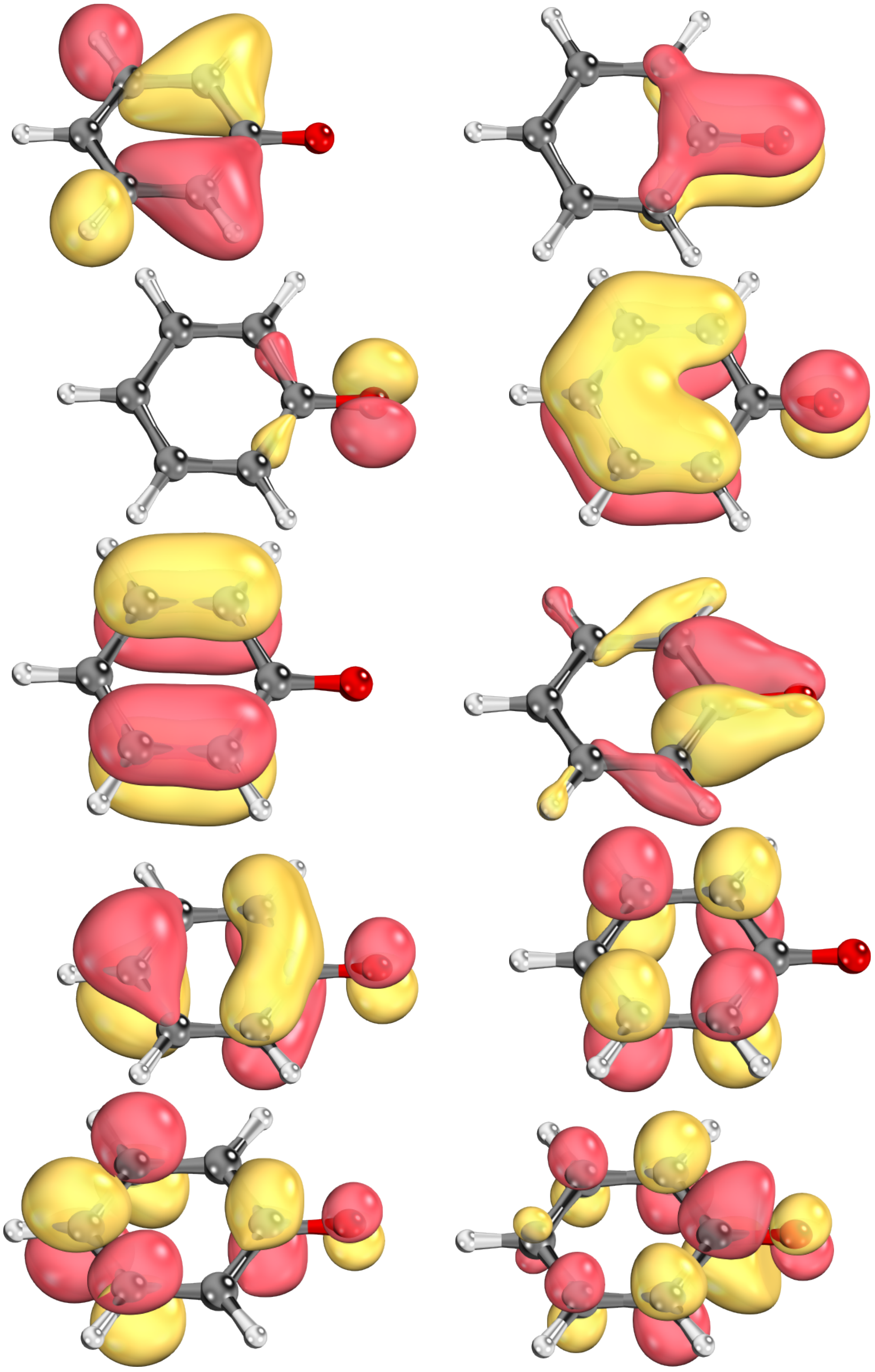

Benzylium UB3LYP Singlet

Atomic Number	Atomic Type	Coordinates (Angstroms)		
		X	Y	Z
6	C	1.122357	1.235886	-0.000056
6	C	1.804027	-0.000208	-0.000063
6	C	1.122102	-1.236134	-0.000024
6	C	-0.254595	-1.246582	0.000024
6	C	-0.984115	0.000325	0.000032
6	C	-0.254357	1.246770	-0.000010
1	H	1.684977	2.163046	-0.000087
1	H	2.890901	0.000042	-0.000100
1	H	1.684156	-2.163623	-0.000032
1	H	-0.807001	-2.181634	0.000056
1	H	-0.806254	2.182124	-0.000003
6	C	-2.355441	0.000020	0.000082
1	H	-2.923672	0.927120	0.000119
1	H	-2.922973	-0.927540	0.000141

Sum of Electronic and Thermal Free Energies = -270.589274

Benzylium Singlet CBS-QB3

Atomic Number	Atomic Type	Coordinates (Angstroms)		
		X	Y	Z
6	C	-1.118968	-1.233142	-0.000057
6	C	-1.798694	0.000037	-0.000065
6	C	-1.118935	1.233181	-0.000025
6	C	0.253521	1.244023	0.000026
6	C	0.981402	-0.000055	0.000032
6	C	0.253487	-1.244073	-0.000007
1	H	-1.680872	-2.158351	-0.000089
1	H	-2.883661	0.000020	-0.000104
1	H	-1.680765	2.158435	-0.000033
1	H	0.805108	2.177172	0.000058
1	H	0.804981	-2.177278	0.000001
6	C	2.348891	0.000011	0.000080
1	H	2.915560	-0.925820	0.000113
1	H	2.915421	0.925925	0.000137

CBS-QB3 Free Energy = -270.104264
Thermal Correction to Gibbs Free Energy = 0.087653
CASPT2 Energy = -270.036803

Occupation				
**A1**	**A2**	**B2**	**B1**	**Coefficient**
	2 0		2 2 0 0 0	0.936992
	2 0		2 0 2 0 0	-0.173130
	2 0		2 0 0 2 0	-0.064455
	u d		2 u d 0 0	0.060666
	0 2		2 2 0 0 0	-0.119597
	u u		2 d d 0 0	0.054356
	u u		2 d 0 d 0	0.069050
	2 0		u d u d 0	-0.090459
	2 0		u d 0 u d	0.063350
	u d		u 2 d 0 0	0.067359
	u u		d 2 d 0 0	-0.069588
	u u		d 2 0 0 d	-0.085002
	0 0		2 2 2 0 0	-0.063605
	0 0		2 2 u 0 d	-0.063130

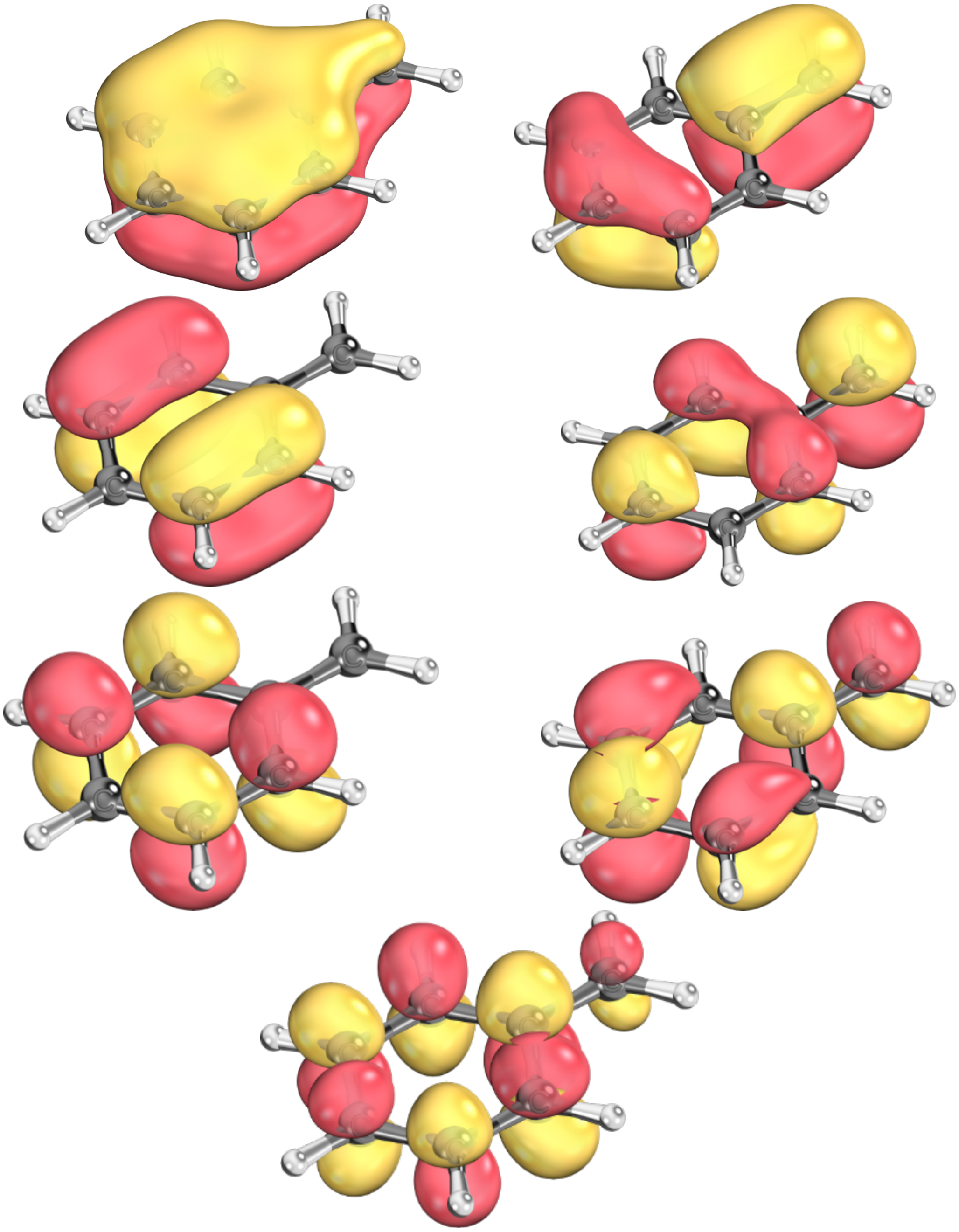

Benzylium Triplet UB3LYP

Atomic Number	Atomic Type	Coordinates (Angstroms)		
		X	Y	Z
6	C	1.170029	1.193277	-0.000061
6	C	1.889815	-0.000312	-0.000072
6	C	1.168509	-1.193901	-0.000028
6	C	-0.274954	-1.204850	0.000022
6	C	-1.036543	0.000204	0.000041
6	C	-0.273842	1.205177	-0.000007
1	H	1.690858	2.147050	-0.000092
1	H	2.973402	-0.001459	-0.000110
1	H	1.689185	-2.147747	-0.000030
1	H	-0.784990	-2.163944	0.000045
1	H	-0.783326	2.164518	-0.000005
6	C	-2.440633	0.000526	0.000098
1	H	-2.999805	0.929309	0.000108
1	H	-2.999605	-0.928458	0.000133

Sum of Electronic and Thermal Free Energies = -270.589274

Benzylium Triplet CBS-QB3

Atomic Number	Atomic Type	Coordinates (Angstroms)		
		X	Y	Z
6	C	-1.166802	-1.190199	-0.000060
6	C	-1.885065	0.000138	-0.000072
6	C	-1.166219	1.190451	-0.000029
6	C	0.274034	1.202062	0.000024
6	C	1.034323	-0.000073	0.000040
6	C	0.273513	-1.202171	-0.000006
1	H	-1.686838	-2.142178	-0.000091
1	H	-2.966493	0.000535	-0.000112
1	H	-1.686149	2.142496	-0.000034
1	H	0.782679	2.159635	0.000052
1	H	0.781930	-2.159863	-0.000003
6	C	2.434590	-0.000229	0.000096
1	H	2.992277	-0.927476	0.000106
1	H	2.992344	0.926981	0.000130

CBS-QB3 Free Energy = -270.069551
Thermal Correction to Gibbs Free Energy = 0.081947
CASPT2 Energy = -269.961460

Occupation				
A1	A2	B2	B1	Coefficient
	2 u		u 2 0 0 0	0.064873
	u 2		2 0 u 0 0	0.050518
	2 u		u d u 0 0	0.108503
	u 2		0 2 u 0 0	0.061907
	u 0		2 2 u 0 0	-0.942529
	u 0		2 d u 0 u	0.070921
	0 u		2 d u u 0	-0.066344
	u 0		d 2 u u 0	-0.096131
	u 0		2 u d u 0	-0.128398
	u 0		2 u u d 0	-0.073257
	u 0		u 2 u d 0	-0.062627
	u 0		2 0 u 2 0	-0.092708
	u 0		u d u u d	0.057319

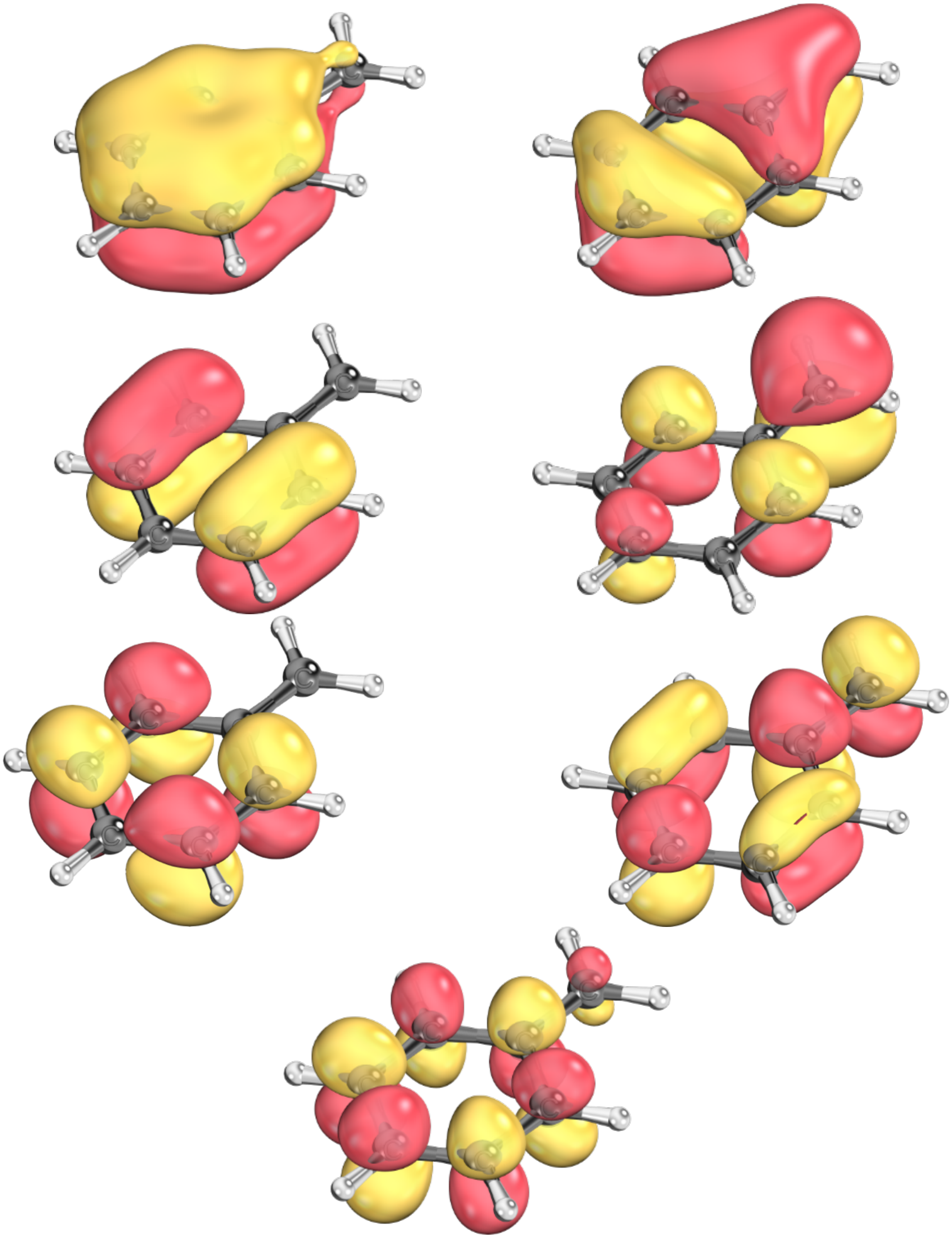

1-Naphthalenylmethylium CBS-QB3 Singlet

Atomic Number	Atomic Type	Coordinates (Angstroms)		
		X	Y	Z
6	C	2.749475	-0.296491	0.000042
6	C	1.768265	-1.262361	0.000057
6	C	0.398975	-0.889915	0.000026
6	C	0.029842	0.495601	-0.000016
6	C	1.048439	1.449427	-0.000049
6	C	2.383902	1.058757	-0.000012
1	H	-0.300129	-2.918555	0.000042
1	H	3.795565	-0.575194	0.000064
1	H	2.027487	-2.314675	0.000083
6	C	-0.604762	-1.876475	0.000010
6	C	-1.403840	0.821052	-0.000047
1	H	0.819928	2.507199	-0.000122
1	H	3.155813	1.819303	-0.000030
6	C	-2.363289	-0.254432	-0.000090
6	C	-1.973563	-1.570308	-0.000048
1	H	-3.416573	0.002212	-0.000164
1	H	-2.707332	-2.365811	-0.000061
6	C	-1.892646	2.092509	0.000091
1	H	-2.961581	2.271636	0.000100
1	H	-1.257968	2.969706	0.000304

CBS-QB3 Free Energy = -423.520419

1-Naphthalenylmethylium CBS-QB3 Triplet

Atomic Number	Atomic Type	Coordinates (Angstroms)		
		X	Y	Z
6	C	-2.745449	-0.296157	0.000006
6	C	-1.728689	-1.278037	0.000015
6	C	-0.363312	-0.929380	0.000009
6	C	-0.007859	0.454778	0.000011
6	C	-1.055072	1.417418	-0.000001
6	C	-2.408461	1.044435	-0.000009
1	H	0.364413	-2.972194	-0.000001
1	H	-3.783945	-0.604171	0.000008
1	H	-2.004560	-2.326867	0.000026
6	C	0.644000	-1.925921	-0.000002
6	C	1.378616	0.828875	0.000005
1	H	-0.816947	2.472621	-0.000010
1	H	-3.175460	1.808191	-0.000024
6	C	2.353051	-0.213971	-0.000011
6	C	1.994125	-1.548946	-0.000018
1	H	3.400682	0.060849	-0.000015
1	H	2.763582	-2.311394	-0.000031
6	C	1.813230	2.186650	-0.000002
1	H	2.872130	2.408371	-0.000241
1	H	1.135023	3.026126	0.000271

CBS-QB3 Free Energy = -423.465061

2-Naphthalenylmethylium CBS-QB3 Singlet

Atomic Number	Atomic Type	Coordinates (Angstroms)		
		X	Y	Z
6	C	2.860747	0.398489	0.000032
6	C	1.791434	1.282177	-0.000160
6	C	0.476660	0.801159	-0.000122
6	C	0.268997	-0.632145	0.000122
6	C	1.397678	-1.513456	0.000316
6	C	2.668215	-1.003353	0.000271
1	H	-0.477123	2.751556	-0.000496
1	H	3.871450	0.790614	0.000000
1	H	1.970743	2.350557	-0.000341
6	C	-0.657208	1.683046	-0.000317
6	C	-1.026024	-1.118108	0.000160
1	H	1.227460	-2.583878	0.000497
1	H	3.527061	-1.662327	0.000417
6	C	-2.155522	-0.231500	-0.000037
6	C	-1.922804	1.199588	-0.000278
1	H	-2.776646	1.866005	-0.000425
1	H	-1.202424	-2.188825	0.000343
6	C	-3.413721	-0.760121	0.000004
1	H	-4.293956	-0.126145	-0.000137
1	H	-3.577275	-1.832209	0.000191

CBS-QB3 Free Energy = -423.519329

2-Naphthalenylmethylium CBS-QB3 Triplet

Atomic Number	Atomic Type	Coordinates (Angstroms)		
		X	Y	Z
6	C	-2.924050	0.372221	-0.000035
6	C	-1.844008	1.255682	-0.000061
6	C	-0.506012	0.776412	-0.000032
6	C	-0.257529	-0.637829	0.000026
6	C	-1.364170	-1.502748	0.000051
6	C	-2.682126	-0.997087	0.000022
1	H	0.419532	2.724419	-0.000107
1	H	-3.937873	0.750916	-0.000058
1	H	-2.017794	2.326355	-0.000107
6	C	0.589515	1.653183	-0.000061
6	C	1.072581	-1.113317	0.000055
1	H	-1.206477	-2.575343	0.000096
1	H	-3.512621	-1.693191	0.000044
6	C	2.188640	-0.221551	0.000027
6	C	1.904268	1.168235	-0.000035
1	H	2.726523	1.874788	-0.000060
1	H	1.250643	-2.182683	0.000101
6	C	3.519754	-0.673637	0.000069
1	H	4.343572	0.027661	0.000385
1	H	3.753323	-1.730302	-0.000445

CBS-QB3 Free Energy = -423.474489

Compound 1-$N(CH_3)_2$ Singlet

Atomic Number	Atomic Type	Coordinates (Angstroms)		
		X	Y	Z
6	C	3.007101	-0.562168	-0.015580
6	C	1.865709	-1.451522	-0.229532
6	C	0.524603	-1.206013	-0.234030
6	C	2.899104	0.867239	0.094948
6	C	-0.173597	0.032062	-0.035633
6	C	1.782355	1.683441	0.086290
6	C	0.424258	1.323747	0.030449
1	H	2.147879	-2.484277	-0.417433
1	H	-0.083906	-2.076122	-0.446563
1	H	3.850975	1.385469	0.184204
1	H	1.977977	2.751079	0.147896
6	C	-1.608738	0.256889	-0.031383
6	C	-1.845034	1.710689	-0.036191
6	C	-0.643546	2.330601	0.032590
1	H	-0.471598	3.397762	0.096303
1	H	-2.813486	2.188045	-0.013028
6	C	4.269052	-1.110761	0.043378
1	H	4.427440	-2.179867	-0.048924
1	H	5.147776	-0.491538	0.186608
7	N	-2.603243	-0.632002	0.021685
6	C	-4.002770	-0.239137	-0.217696
1	H	-4.056177	0.637898	-0.858706
1	H	-4.505905	-1.065698	-0.723917
1	H	-4.512935	-0.044140	0.731942
6	C	-2.437871	-2.038130	0.417556
1	H	-2.348480	-2.685311	-0.461606
1	H	-1.571980	-2.154810	1.065610
1	H	-3.328633	-2.336108	0.974827

Uncorrected Sum of Electronic and Thermal Free Energies = -558.094885
Corrected Sum of Electronic and Thermal Free Energies = -558.094890

Compound 1-$N(CH_3)_2$ Triplet

Atomic Number	Atomic Type	Coordinates (Angstroms)		
		X	Y	Z
6	C	2.994695	-0.571283	-0.033681
6	C	1.865912	-1.446178	-0.194575
6	C	0.498060	-1.186755	-0.178322
6	C	2.917964	0.861004	0.112508
6	C	-0.171660	0.029351	-0.011102
6	C	1.810505	1.686497	0.123035
6	C	0.446771	1.355040	0.051933
1	H	2.127658	-2.490201	-0.344880
1	H	-0.111852	-2.067929	-0.333775
1	H	3.878594	1.360890	0.201721
1	H	2.018881	2.751555	0.197407
6	C	-1.619857	0.263367	-0.022446
6	C	-1.825535	1.685738	-0.046192
6	C	-0.602182	2.319800	0.022112
1	H	-0.450080	3.391421	0.061244
1	H	-2.786435	2.179817	-0.052860
6	C	4.258215	-1.155512	-0.039743
1	H	4.385200	-2.226119	-0.158503
1	H	5.156060	-0.558032	0.075399
7	N	-2.621097	-0.634947	0.001666
6	C	-4.003996	-0.209260	-0.262830
1	H	-4.023524	0.582717	-1.010760
1	H	-4.559431	-1.063661	-0.652694
1	H	-4.488389	0.137009	0.657765
6	C	-2.474085	-2.030890	0.429807
1	H	-2.334306	-2.698038	-0.428416
1	H	-1.643951	-2.133934	1.127219
1	H	-3.389590	-2.326374	0.946431

Sum of Electronic and Thermal Free Energies = -558.099551

Compound 1-NH_2 UB3LYP Singlet

Atomic Number	Atomic Type	Coordinates (Angstroms)		
		X	Y	Z
6	C	-2.463898	-0.224419	0.000038
6	C	-1.567218	-1.374616	-0.000595
6	C	-0.203078	-1.432064	-0.000524
6	C	-2.039729	1.146536	0.000049
6	C	0.735161	-0.359372	-0.000199
6	C	-0.767811	1.701345	-0.000025
6	C	0.468694	1.042600	-0.000015
1	H	-2.078225	-2.333553	-0.000970
1	H	0.210855	-2.438263	-0.000868
1	H	-2.854157	1.866962	0.000103
1	H	-0.725639	2.787601	0.000036
6	C	2.167254	-0.488555	-0.000017
6	C	2.767925	0.841287	0.000040
6	C	1.756854	1.751017	-0.000113
1	H	1.857148	2.829004	-0.000292
1	H	3.832150	1.039654	-0.000343
6	C	-3.828874	-0.460701	0.000556
1	H	-4.228755	-1.468985	0.000592
1	H	-4.543144	0.355315	0.000930
7	N	2.867639	-1.622186	0.000540
1	H	3.879221	-1.609458	0.000655
1	H	2.425390	-2.531325	0.001205

Uncorrected Sum of Electronic and Thermal Free Energies = -479.526851
Corrected Sum of Electronic and Thermal Free Energies = -479.525725

Compound 1-NH_2 CBS-QB3 Singlet

Atomic Number	Atomic Type	Coordinates (Angstroms)		
		X	Y	Z
6	C	-2.471300	-0.220897	0.000061
6	C	-1.574121	-1.388176	-0.000149
6	C	-0.230761	-1.451386	-0.000190
6	C	-2.017392	1.149032	-0.000053
6	C	0.726092	-0.366283	-0.000057
6	C	-0.753316	1.693735	-0.000124
6	C	0.474317	1.006826	-0.000060
1	H	-2.098299	-2.337876	-0.000293
1	H	0.185342	-2.455223	-0.000371
1	H	-2.825345	1.875521	-0.000056
1	H	-0.696841	2.776722	-0.000200
6	C	2.145035	-0.498423	0.000034
6	C	2.778279	0.849896	0.000102
6	C	1.786848	1.742848	-0.000072
1	H	1.871740	2.820013	-0.000110
1	H	3.843988	1.027807	0.000213
6	C	-3.820474	-0.407957	0.000313
1	H	-4.254383	-1.400177	0.000410
1	H	-4.508110	0.427941	0.000420
7	N	2.849981	-1.610914	0.000118
1	H	3.860926	-1.591076	0.000193
1	H	2.411878	-2.522543	0.000135

CBS-QB3 Free Energy = -478.763143
Thermal Correction to Gibbs Free Energy = 0.144397
CASPT2 Energy = -478.589640

Occupation		
A1	A2	Coefficient
	2 2 2 2 2 2 2 0 0 0 0 0 0	0.869893
	2 2 2 2 2 2 0 2 0 0 0 0 0	-0.163632
	2 2 2 2 2 2 0 0 2 0 0 0 0	-0.084453
	2 2 2 2 2 u d u d 0 0 0 0	-0.103309
	2 2 2 u 2 d 2 0 0 0 0 0 0	0.065715
	2 2 2 u 2 d 0 2 0 0 0 0 0	-0.073056
	2 2 2 u 2 d u 0 0 d 0 0 0	0.052267
	2 2 u 2 2 d u d 0 0 0 0 0	-0.069386
	2 u 2 2 2 d u 0 0 0 d 0 0	-0.061647
	2 2 2 2 0 2 0 0 2 0 0 0 0	-0.066059
	2 2 u 2 d 2 u 0 d 0 0 0 0	0.071289

	2 2 u 2 d 2 0 02 0 0 0	0.050298
	2 2 2 0 2 2 0 2 0 0 0 0	-0.052133

Compound 1-NH_2 UB3LYP Triplet

Atomic Number	Atomic Type	Coordinates (Angstroms)		
		X	Y	Z
6	C	-2.455058	-0.237025	0.000033
6	C	-2.053334	1.149878	0.000158
6	C	-0.790275	1.713961	-0.000034
6	C	-1.562340	-1.365096	-0.000509
6	C	0.459587	1.075462	-0.000091
6	C	-0.172633	-1.412326	-0.000407
6	C	0.735756	-0.354424	-0.000013
1	H	-2.879199	1.856035	0.000355
1	H	-0.758660	2.801074	-0.000083
1	H	-2.057159	-2.332455	-0.000914
6	C	1.722715	1.751423	-0.000230
1	H	1.839447	2.828246	-0.000344
6	C	2.754759	0.831054	-0.000007
6	C	2.188738	-0.479533	0.000198
1	H	3.814639	1.051843	-0.000074
1	H	0.241313	-2.418761	-0.000704
6	C	-3.820140	-0.511631	0.000381
1	H	-4.555958	0.285226	0.000854
1	H	-4.191507	-1.530709	0.000213
7	N	2.881701	-1.623543	0.000432
1	H	3.892918	-1.616512	0.000232
1	H	2.435614	-2.529640	0.000557

Sum of Electronic and Thermal Free Energies = -479.532267

Compound 1-NH_2 CBS-QB3 Triplet

Atomic Number	Atomic Type	Coordinates (Angstroms)		
		X	Y	Z
6	C	-2.448589	-0.237064	0.000043
6	C	-2.048518	1.147582	0.000110
6	C	-0.789779	1.710623	0.000030
6	C	-1.557764	-1.363052	-0.000204
6	C	0.457260	1.073726	-0.000027
6	C	-0.172096	-1.409680	-0.000202
6	C	0.733438	-0.354058	-0.000036
1	H	-2.874278	1.851033	0.000214
1	H	-0.758335	2.795995	0.000025
1	H	-2.052921	-2.328152	-0.000374
6	C	1.717665	1.748157	-0.000085
1	H	1.834146	2.822902	-0.000118
6	C	2.747509	0.830574	-0.000038
6	C	2.184248	-0.479235	0.000018
1	H	3.805196	1.051869	-0.000033
1	H	0.242066	-2.414316	-0.000341
6	C	-3.809236	-0.511112	0.000199
1	H	-4.543391	0.284494	0.000411
1	H	-4.179029	-1.528573	0.000122
7	N	2.875491	-1.619749	0.000135
1	H	3.885043	-1.612314	0.000070
1	H	2.428235	-2.523462	0.000234

CBS-QB3 Free Energy = -478.767241
Thermal Correction to Gibbs Free Energy = 0.142634
CASPT2 Energy = -478.589729

Occupation		
A1	A2	Coefficient
	2 2 2 2 2 u u 0 0 0 0 0	-0.875603
	2 2 2 2 u 2 0 u 0 0 0 0	0.055340
	2 u 2 2 2 2 0 0 u 0 0 0	-0.056462
	2 2 2 2 u d u u 0 0 0 0	-0.152476
	2 2 2 u 2 d u 0 u 0 0 0	-0.088117
	2 u 2 2 2 d u 0 0 u 0 0	0.060900
	2 2 2 2 u u u d 0 0 0 0	0.106633
	2 2 2 u 2 u d 0 u 0 0 0	0.066524
	2 2 2 u 2 u u 0 d 0 0 0	0.076943
	2 2 2 2 0 u u 2 0 0 0 0	0.096771
	2 2 2 u d u u u d 0 0 0	0.058514

	2 u 2 2 d u u u 0 d 0 0	-0.052565
	2 2 2 0 2 u u 0 2 0 0 0	0.060831
	2 2 0 2 2 u u 0 0 0 0 2	0.050299

Compound 1-OH UB3LYP Singlet

Atomic Number	Atomic Type	Coordinates (Angstroms)		
		X	Y	Z
6	C	2.438361	-0.226292	-0.000036
6	C	1.549104	-1.375855	-0.000073
6	C	0.164310	-1.433855	-0.000057
6	C	2.035749	1.168057	0.000020
6	C	-0.730921	-0.361750	-0.000026
6	C	0.773151	1.733363	0.000053
6	C	-0.469808	1.083701	0.000040
1	H	2.057669	-2.336085	-0.000105
1	H	-0.278890	-2.426331	-0.000069
1	H	2.864377	1.870699	0.000034
1	H	0.737983	2.820257	0.000073
6	C	-2.164789	-0.498447	-0.000006
6	C	-2.764662	0.782348	0.000101
6	C	-1.738257	1.733672	0.000010
1	H	-1.881149	2.807432	0.000043
1	H	-3.828381	0.986602	0.000168
6	C	3.786271	-0.494538	-0.000044
1	H	4.163256	-1.512122	-0.000053
1	H	4.523444	0.301908	0.000022
8	O	-2.740018	-1.691606	-0.000007
1	H	-3.709224	-1.641930	0.000055

Uncorrected Sum of Electronic and Thermal Free Energies = -499.380900
Corrected Sum of Electronic and Thermal Free Energies = -499.370407

Compound 1-OH CBS-QB3 Singlet

Atomic Number	Atomic Type	Coordinates (Angstroms)		
		X	Y	Z
6	C	2.442805	-0.214660	-0.000036
6	C	1.572200	-1.393844	-0.000058
6	C	0.228370	-1.466831	-0.000043
6	C	1.998237	1.147400	0.000005
6	C	-0.729030	-0.387977	-0.000001
6	C	0.723046	1.699711	0.000035
6	C	-0.487433	1.013706	0.000033
1	H	2.108426	-2.336241	-0.000091
1	H	-0.207132	-2.460538	-0.000066
1	H	2.804535	1.875629	0.000016
1	H	0.669916	2.782967	0.000062
6	C	-2.114256	-0.523467	0.000014
6	C	-2.774588	0.814473	0.000056
6	C	-1.802214	1.732319	0.000064
1	H	-1.903741	2.807400	0.000089
1	H	-3.843963	0.976263	0.000073
6	C	3.806276	-0.373096	-0.000054
1	H	4.259944	-1.356925	-0.000083
1	H	4.476405	0.477062	-0.000036
8	O	-2.731239	-1.682284	-0.000008
1	H	-3.694942	-1.593736	0.000007

CBS-QB3 Free Energy = -498.608899
Thermal Correction to Gibbs Free Energy = 0.131725
CASPT2 Energy = -498.424333

Occupation		
A1	A2	Coefficient
	2 2 2 2 2 2 2 0 0 0 0 0 0	0.874045
	2 2 2 2 2 2 0 2 0 0 0 0 0	-0.196185
	2 2 2 2 2 2 0 0 2 0 0 0 0	-0.056987
	2 2 2 2 u d u d 0 0 0 0	-0.106624
	2 2 2 u 2 d 0 u d 0 0 0	-0.065362
	2 u 2 2 2 d u d 0 0 0 0	-0.078916
	2 u 2 2 2 d u 0 0 d 0 0	0.076330
	u 2 2 2 2 d u 0 0 0 d 0	-0.060277
	2 2 2 2 0 2 0 2 0 0 0 0	-0.066225
	2 2 2 2 0 2 0 u d 0 0 0	-0.070222
	2 2 2 2 0 2 0 0 2 0 0 0	-0.063090
	2 u 2 2 d 2 0 u d 0 0 0	0.052803

	2 2 2 0 2 2 0 2 0 0 0 0	-0.050279
	2 2 2 0 2 2 0 0 2 0 0 0	-0.051070
	2 2 0 2 2 2 0 0 0 0 0 2	-0.053811

Compound 1-OH UB3LYP Triplet

Atomic Number	Atomic Type	Coordinates (Angstroms)		
		X	Y	Z
6	C	2.443047	-0.221751	-0.000027
6	C	1.562814	-1.361045	-0.000070
6	C	0.174658	-1.421484	-0.000062
6	C	2.028863	1.161746	0.000028
6	C	-0.727298	-0.357022	-0.000027
6	C	0.762496	1.722076	0.000056
6	C	-0.477710	1.066912	0.000036
1	H	2.068467	-2.322975	-0.000101
1	H	-0.262998	-2.416719	-0.000080
1	H	2.850028	1.873690	0.000046
1	H	0.724397	2.808923	0.000075
6	C	-2.171656	-0.499945	-0.000013
6	C	-2.779311	0.778987	0.000114
6	C	-1.761142	1.723067	-0.000012
1	H	-1.899004	2.797630	0.000015
1	H	-3.843431	0.978949	0.000203
6	C	3.812672	-0.478378	-0.000033
1	H	4.197017	-1.492674	-0.000067
1	H	4.538863	0.327344	0.000011
8	O	-2.734205	-1.697061	-0.000012
1	H	-3.704294	-1.656651	0.000051

Sum of Electronic and Thermal Energies = -499.392167

Compound 1-OH CBS-QB3 Triplet

Atomic Number	Atomic Type	Coordinates (Angstroms)		
		X	Y	Z
6	C	2.436358	-0.221719	-0.000032
6	C	1.557956	-1.358872	-0.000063
6	C	0.173854	-1.418546	-0.000053
6	C	2.024112	1.159575	0.000017
6	C	-0.724983	-0.356265	-0.000013
6	C	0.761924	1.718894	0.000047
6	C	-0.475223	1.065344	0.000036
1	H	2.063520	-2.318697	-0.000095
1	H	-0.264937	-2.411383	-0.000073
1	H	2.845261	1.868661	0.000030
1	H	0.724018	2.803977	0.000071
6	C	-2.167064	-0.500325	0.000004
6	C	-2.771734	0.777940	0.000078
6	C	-1.756321	1.719654	0.000029
1	H	-1.893904	2.792056	0.000052
1	H	-3.833868	0.977756	0.000124
6	C	3.801635	-0.478165	-0.000047
1	H	4.183786	-1.491034	-0.000080
1	H	4.525988	0.326394	-0.000022
8	O	-2.727338	-1.693389	-0.000009
1	H	-3.694253	-1.645708	0.000025

CBS-QB3 Free Energy = -498.621447
Thermal Correction to Gibbs Free Energy = 0.130746
CASPT2 Energy = -498.437990

Occupation		
A1	A2	Coefficient
	2 2 2 2 2 u u 0 0 0 0 0	0.886310
	2 2 2 2 u d u u 0 0 0 0	0.078751
	2 2 2 u 2 d u 0 u 0 0 0	-0.096861
	2 u 2 2 2 d u 0 0 0 u 0	-0.050876
	2 2 2 2 u u d u 0 0 0 0	-0.131927
	2 2 2 2 u u u d 0 0 0 0	-0.094754
	2 2 2 u 2 u u 0 d 0 0 0	0.071665
	2 2 2 2 0 u u 2 0 0 0 0	-0.094071
	2 2 2 0 2 u u 0 2 0 0 0	-0.074360

Compound 1-OCH_3 UB3LYP Singlet

Atomic Number	Atomic Type	Coordinates (Angstroms)		
		X	Y	Z
6	C	2.783841	-0.487332	-0.000057
6	C	1.733658	-1.490253	-0.000074
6	C	0.355237	-1.339954	-0.000048
6	C	2.588205	0.954733	-0.000007
6	C	-0.373137	-0.148758	0.000002
6	C	1.424842	1.698849	0.000034
6	C	0.097662	1.237301	0.000039
1	H	2.093400	-2.515550	-0.000114
1	H	-0.230452	-2.255588	-0.000069
1	H	3.511520	1.527540	-0.000002
1	H	1.549043	2.779193	0.000068
6	C	-1.817841	-0.072684	0.000026
6	C	-2.218081	1.291780	0.000073
6	C	-1.060966	2.071532	0.000086
1	H	-1.037757	3.154633	0.000121
1	H	-3.233827	1.662137	0.000097
6	C	4.077721	-0.945237	-0.000093
1	H	4.304489	-2.006354	-0.000132
1	H	4.921464	-0.262838	-0.000084
8	O	-2.551514	-1.160342	0.000001
6	C	-3.998566	-1.048832	0.000023
1	H	-4.328789	-0.526898	-0.901452
1	H	-4.363669	-2.073455	0.000001
1	H	-4.328765	-0.526946	0.901535

Uncorrected Sum of Electronic and Thermal Free Energies = -538.665147
Corrected Sum of Electronic and Thermal Free Energies = -538.654043

Compound 1-OCH_3 CBS-QB3 Singlet

Atomic Number	Atomic Type	Coordinates (Angstroms)		
		X	Y	Z
6	C	2.792110	-0.471343	-0.000058
6	C	1.748271	-1.504930	-0.000076
6	C	0.409149	-1.376504	-0.000051
6	C	2.548727	0.943532	-0.000009
6	C	-0.374961	-0.163959	0.000001
6	C	1.371344	1.674234	0.000032
6	C	0.068897	1.171720	0.000036
1	H	2.136053	-2.517765	-0.000115
1	H	-0.170848	-2.293158	-0.000072
1	H	3.453237	1.545639	-0.000003
1	H	1.476605	2.753699	0.000066
6	C	-1.780073	-0.096960	0.000025
6	C	-2.233402	1.327286	0.000077
6	C	-1.130926	2.081394	0.000086
1	H	-1.068204	3.159817	0.000120
1	H	-3.259868	1.660144	0.000104
6	C	4.112131	-0.835173	-0.000089
1	H	4.411199	-1.876221	-0.000127
1	H	4.903305	-0.096533	-0.000077
8	O	-2.527843	-1.157648	0.000002
6	C	-3.981029	-1.062925	0.000025
1	H	-4.316514	-0.550438	-0.901427
1	H	-4.327155	-2.091740	0.000000
1	H	-4.316489	-0.550491	0.901518

CBS-QB3 Free Energy = -537.828453
Thermal Correction to Gibbs Free Energy = 0.157178
CASPT2 Energy = -537.637998

Occupation		
**A1**	**A2**	**Coefficient**
	2 2 2 2 2 2 2 0 0 0 0 0 0 0	-0.866920
	2 2 2 2 2 2 0 2 0 0 0 0 0 0	0.188418
	2 2 2 2 2 2 0 0 2 0 0 0 0 0	0.072088
	2 2 2 2 2 u d u d 0 0 0 0 0	0.116090
	2 2 2 u 2 2 d 2 0 0 0 0 0 0	-0.052932
	2 2 2 u 2 2 d 0 2 0 0 0 0 0	0.061124
	2 2 2 u 2 2 d 0 u d 0 0 0 0	0.051850
	2 2 2 u 2 2 d u 0 0 0 d 0 0	-0.055276
	2 2 u 2 2 2 d u d 0 0 0 0 0	-0.073731

	2 2 u 2 2 2 d u 0 0 d 0 0 0	0.056522
	2 u 2 2 2 2 d u 0 0 0 d 0 0	0.069073
	2 2 2 2 2 0 2 0 2 0 0 0 0 0	0.053683
	2 2 2 2 2 0 2 0 u d 0 0 0 0	-0.052636
	2 2 2 2 2 0 2 0 0 2 0 0 0 0	0.065710
	2 2 u 2 2 d 2 u 0 d 0 0 0 0	0.061614
	2 2 u 2 2 d 2 0 u d 0 0 0 0	-0.050521
	2 2 u 2 2 d 2 0 0 2 0 0 0 0	0.054150
	2 2 2 2 0 2 2 0 0 0 0 0 0 2	0.060482
	2 2 2 0 2 2 2 0 2 0 0 0 0 0	0.051804
	0 2 2 2 2 2 2 2 0 0 0 0 2 0	0.057111

Compound 1-OCH_3 UB3LYP Triplet

Atomic Number	Atomic Type	Coordinates (Angstroms)		
		X	Y	Z
6	C	-2.785455	-0.481171	0.000051
6	C	-1.744522	-1.475601	0.000085
6	C	-0.363011	-1.327162	0.000068
6	C	-2.583206	0.948279	-0.000012
6	C	0.371592	-0.141578	0.000014
6	C	-1.414754	1.690969	-0.000052
6	C	-0.089795	1.229362	-0.000042
1	H	-2.100515	-2.502387	0.000125
1	H	0.218626	-2.245644	0.000092
1	H	-3.501443	1.529702	-0.000024
1	H	-1.539232	2.771405	-0.000082
6	C	1.824980	-0.069209	-0.000010
6	C	2.229140	1.292813	-0.000098
6	C	1.078362	2.069707	-0.000040
1	H	1.051553	3.152798	-0.000067
1	H	3.245302	1.661454	-0.000154
6	C	-4.101600	-0.939266	0.000078
1	H	-4.330284	-1.999515	0.000123
1	H	-4.939545	-0.250545	0.000054
8	O	2.547315	-1.164844	0.000006
6	C	3.996081	-1.065750	-0.000038
1	H	4.329213	-0.546588	0.901632
1	H	4.351762	-2.093635	-0.000019
1	H	4.329161	-0.546639	-0.901758

Sum of Electronic and Thermal Free Energies = -538.676331

Compound 1-OCH_3 CBS-QB3 Triplet

Atomic Number	Atomic Type	Coordinates (Angstroms)		
		X	Y	Z
6	C	2.777411	-0.481863	-0.000054
6	C	1.737644	-1.473723	-0.000080
6	C	0.360455	-1.323996	-0.000059
6	C	2.577762	0.945468	0.000003
6	C	-0.370018	-0.140218	-0.000007
6	C	1.413945	1.687542	0.000044
6	C	0.091902	1.228101	0.000040
1	H	2.093345	-2.498598	-0.000120
1	H	-0.223315	-2.239045	-0.000081
1	H	3.496171	1.523080	0.000013
1	H	1.539251	2.766099	0.000076
6	C	-1.820815	-0.067963	0.000017
6	C	-2.222086	1.292574	0.000085
6	C	-1.073845	2.066895	0.000061
1	H	-1.046942	3.147795	0.000092
1	H	-3.235814	1.661868	0.000126
6	C	4.088629	-0.940068	-0.000085
1	H	4.314590	-1.998749	-0.000127
1	H	4.925187	-0.252943	-0.000067
8	O	-2.539313	-1.160828	-0.000003
6	C	-3.987808	-1.065371	0.000032
1	H	-4.322538	-0.547759	-0.899841
1	H	-4.341991	-2.091585	0.000009
1	H	-4.322498	-0.547815	0.899952

CBS-QB3 Free Energy = -537.841563
Thermal Correction to Gibbs Free Energy = 0.156352
CASPT2 Energy = -537.653129

Occupation		
A1	A2	Coefficient
	2 2 2 2 2 2 u u 0 0 0 0 0 0	-0.882478
	2 2 2 2 2 u 2 0 u 0 0 0 0 0	-0.062713
	2 2 2 2 2 u d u u 0 0 0 0 0	0.100876
	2 2 2 u 2 2 d u 0 u 0 0 0 0	0.092207
	2 2 2 2 2 u u d u 0 0 0 0 0	-0.102985
	2 2 2 2 2 u u u d 0 0 0 0 0	-0.097992
	2 2 2 u 2 2 u u 0 d 0 0 0 0	-0.072035
	2 2 u 2 2 2 u d 0 u 0 0 0 0	0.056414
	2 2 2 2 2 u 0 2 u 0 0 0 0 0	0.051470

	2 2 2 2 2 0 u u 2 0 0 0 0 0	0.095085
	2 2 u 2 2 d u u u 0 d 0 0 0	-0.051643
	2 2 2 2 0 2 u u 0 0 0 0 0 2	0.052026
	2 2 2 0 2 2 u u 0 2 0 0 0 0	0.072143

Compound 1-CH_3 UB3LYP Singlet

Atomic Number	Atomic Type	Coordinates (Angstroms)		
		X	Y	Z
6	C	-2.433186	-0.293582	0.000056
6	C	-1.510700	-1.408414	0.000009
6	C	-0.126188	-1.416027	-0.000036
6	C	-2.095168	1.117205	0.000048
6	C	0.756376	-0.329362	-0.000049
6	C	-0.858776	1.733653	0.000011
6	C	0.410454	1.138053	-0.000026
1	H	-1.986844	-2.384858	0.000012
1	H	0.334884	-2.400602	-0.000059
1	H	-2.953199	1.783795	0.000081
1	H	-0.868001	2.821081	0.000023
6	C	2.168873	-0.422835	-0.000078
6	C	2.678402	0.905269	-0.000070
6	C	1.630100	1.838857	-0.000043
1	H	1.736465	2.916108	-0.000046
1	H	3.733996	1.153740	-0.000100
6	C	-3.774410	-0.598642	0.000100
1	H	-4.123201	-1.626791	0.000075
1	H	-4.534057	0.176940	0.000100
6	C	2.992371	-1.669432	0.000018
1	H	2.787336	-2.285905	0.883520
1	H	2.785173	-2.287594	-0.881773
1	H	4.058555	-1.434363	-0.001477

Uncorrected Sum of Electronic and Thermal Free Energies = -463.456218
Corrected Sum of Electronic and Thermal Free Energies = -463.456100

Compound 1-CH_3 CBS-QB3 Singlet

Atomic Number	Atomic Type	Coordinates (Angstroms)		
		X	Y	Z
6	C	2.415423	-0.301616	-0.000027
6	C	1.500554	-1.420260	0.000015
6	C	0.129130	-1.433531	0.000046
6	C	2.097108	1.105756	-0.000049
6	C	-0.766868	-0.352070	0.000039
6	C	0.870418	1.731018	-0.000060
6	C	-0.398868	1.147640	-0.000041
1	H	1.984395	-2.390572	0.000018
1	H	-0.327347	-2.417603	0.000061
1	H	2.961058	1.761495	-0.000061
1	H	0.885979	2.816640	-0.000077
6	C	-2.148485	-0.428980	0.000040
6	C	-2.642049	0.916545	0.000032
6	C	-1.596944	1.850078	-0.000015
1	H	-1.701611	2.924596	-0.000024
1	H	-3.695403	1.172107	0.000053
6	C	3.753605	-0.602692	-0.000055
1	H	4.103884	-1.628947	-0.000045
1	H	4.510336	0.173600	-0.000084
6	C	-3.029383	-1.637931	0.000060
1	H	-3.679413	-1.642852	-0.879981
1	H	-2.464390	-2.569340	0.000070
1	H	-3.679336	-1.642866	0.880166

CBS-QB3 Free Energy = -462.693825
Thermal Correction to Gibbs Free Energy = 0.151501
CASPT2 Energy = -462.530620

Occupation		
A1	A2	Coefficient
	2 2 2 2 2 2 2 0 0 0 0 0 0	0.874805
	2 2 2 2 2 2 0 2 0 0 0 0 0	-0.187509
	2 2 2 2 2 2 0 0 2 0 0 0 0	-0.060883
	2 2 2 2 u d u d 0 0 0 0	0.093893
	2 2 2 2 u d u 0 0 d 0 0	0.054707
	2 2 2 u 2 d 0 u d 0 0 0	-0.053578
	2 u 2 2 2 d u d 0 0 0 0	0.094069
	2 u 2 2 2 d u 0 d 0 0 0	0.054539
	u 2 2 2 2 d u 0 0 0 0 d	0.052463
	2 2 2 2 0 2 2 2 0 0 0 0 0	-0.103818

	2 2 2 2 0 2 0 2 0 0 0 0	-0.060057
	2 u 2 2 d 2 u 0 d 0 0 0	-0.055687
	u 2 2 2 d 2 u 0 0 d 0 0	-0.056943
	2 2 2 2 u u d d 0 0 0 0	0.090541
	2 2 2 2 u u 0 d d 0 0 0	0.052589

Compound 1-CH_3 UB3LYP Triplet

Atomic Number	Atomic Type	Coordinates (Angstroms)		
		X	Y	Z
6	C	2.458067	-0.273941	-0.000068
6	C	2.088810	1.120002	-0.000045
6	C	0.838242	1.716690	-0.000005
6	C	1.533443	-1.378382	-0.000055
6	C	-0.421434	1.100830	0.000023
6	C	0.144895	-1.393026	-0.000019
6	C	-0.738885	-0.308212	0.000016
1	H	2.930552	1.807698	-0.000061
1	H	0.833689	2.804203	0.000007
1	H	2.005153	-2.357684	-0.000077
6	C	-1.679731	1.808970	0.000064
1	H	-1.776942	2.888329	0.000078
6	C	-2.725656	0.888452	0.000082
6	C	-2.196624	-0.418715	0.000054
1	H	-3.781017	1.130438	0.000113
1	H	-0.308243	-2.381210	-0.000017
6	C	3.817877	-0.577603	-0.000106
1	H	4.570624	0.203262	-0.000117
1	H	4.167205	-1.604443	-0.000125
6	C	-2.973214	-1.685945	0.000060
1	H	-2.734150	-2.296021	-0.881165
1	H	-2.734099	-2.296043	0.881257
1	H	-4.047500	-1.493248	0.000093

Sum of Electronic and Thermal Free Energies = -463.460927

Compound 1-CH_3 CBS-QB3 Triplet

Atomic Number	Atomic Type	Coordinates (Angstroms)		
		X	Y	Z
6	C	2.451969	-0.273782	-0.000068
6	C	2.083866	1.117679	-0.000045
6	C	0.837455	1.712855	-0.000005
6	C	1.529398	-1.376127	-0.000055
6	C	-0.419311	1.098404	0.000023
6	C	0.144969	-1.390569	-0.000019
6	C	-0.736165	-0.307943	0.000016
1	H	2.925204	1.803009	-0.000060
1	H	0.832680	2.798607	0.000006
1	H	2.001254	-2.353321	-0.000078
6	C	-1.675286	1.805331	0.000064
1	H	-1.772045	2.882633	0.000078
6	C	-2.718876	0.887366	0.000082
6	C	-2.191472	-0.417294	0.000054
1	H	-3.772179	1.128795	0.000113
1	H	-0.307996	-2.376914	-0.000018
6	C	3.807487	-0.576569	-0.000105
1	H	4.558359	0.203234	-0.000116
1	H	4.155399	-1.601711	-0.000124
6	C	-2.968123	-1.681682	0.000061
1	H	-2.727951	-2.290323	-0.879441
1	H	-2.727900	-2.290345	0.879533
1	H	-4.040282	-1.489674	0.000094

CBS-QB3 Free Energy = -462.698488
Thermal Correction to Gibbs Free Energy = 0.151453
CASPT2 Energy = -462.530593

Occupation		
A1	A2	Coefficient
	2 2 2 2 2 u u 0 0 0 0 0	-0.884847
	2 2 2 2 u d u 0 u 0 0 0	0.061823
	2 2 2 2 u d u 0 0 u 0 0	-0.056354
	2 2 2 u 2 d u u 0 0 0 0	-0.098291
	2 u 2 2 2 d u 0 0 u 0 0	0.055498
	2 2 2 2 u u d 0 u 0 0 0	-0.154361
	2 2 2 2 u u u 0 d 0 0 0	-0.092366
	2 2 2 u 2 u u d 0 0 0 0	0.070877
	2 u 2 2 2 u d 0 0 u 0 0	0.063320
	2 2 2 2 0 u u 0 2 0 0 0	0.095163

	2 2 2 0 2 u u 2 0 0 0 0	0.082063

Compound 1-F UB3LYP Singlet

Atomic Number	Atomic Type	Coordinates (Angstroms)		
		X	Y	Z
6	C	-2.431629	-0.208403	0.000191
6	C	-1.575054	-1.362542	-0.000133
6	C	-0.196328	-1.438100	-0.000321
6	C	-2.001045	1.166429	0.000378
6	C	0.729699	-0.380930	-0.000258
6	C	-0.728949	1.718340	0.000344
6	C	0.499184	1.045918	0.000110
1	H	-2.096762	-2.315375	-0.000167
1	H	0.230145	-2.438359	-0.000422
1	H	-2.813345	1.889063	0.000556
1	H	-0.680370	2.804438	0.000360
6	C	2.147844	-0.513346	-0.000314
6	C	2.796334	0.755322	0.000143
6	C	1.798527	1.707779	-0.000435
1	H	1.935857	2.781732	-0.000519
1	H	3.866547	0.912800	0.000357
6	C	-3.814237	-0.418394	0.000320
1	H	-4.231225	-1.419980	0.000132
1	H	-4.512752	0.411414	0.000509
9	F	2.772869	-1.673129	-0.000107

Uncorrected Sum of Electronic and Thermal Free Energies = -523.395458
Corrected Sum of Electronic and Thermal Free Energies = -523.392090

Compound 1-F CBS-QB3 Singlet

Atomic Number	Atomic Type	Coordinates (Angstroms)		
		X	Y	Z
6	C	-2.391531	-0.228502	-0.000007
6	C	-1.538397	-1.394661	-0.000088
6	C	-0.168796	-1.465274	-0.000129
6	C	-2.009874	1.162574	0.000141
6	C	0.750007	-0.408039	-0.000075
6	C	-0.755569	1.743091	0.000150
6	C	0.481780	1.106046	0.000037
1	H	-2.067268	-2.340785	-0.000142
1	H	0.268274	-2.459137	-0.000250
1	H	-2.844737	1.854905	0.000290
1	H	-0.728412	2.828433	0.000299
6	C	2.118350	-0.517305	-0.000150
6	C	2.724282	0.770769	-0.000104
6	C	1.722953	1.747431	0.000164
1	H	1.879662	2.815405	0.000382
1	H	3.792841	0.942833	-0.000071
6	C	-3.746285	-0.457978	0.000006
1	H	-4.149731	-1.464729	0.000711
1	H	-4.461485	0.356891	0.000858
9	F	2.798815	-1.653637	-0.000194

CBS-QB3 Free Energy = -522.613103
Thermal Correction to Gibbs Free Energy = 0.118968
CASPT2 Energy = -522.418805

Occupation		
A1	A2	Coefficient
	2 2 2 2 2 2 0 0 0 0 0 0	0.874681
	2 2 2 2 2 0 2 0 0 0 0 0	-0.202963
	2 2 2 2 2 0 0 2 0 0 0 0	-0.052802
	2 2 2 2 2 0 u 0 d 0 0 0	0.052576
	2 2 2 2 u d u d 0 0 0 0	0.092657
	2 2 2 u 2 d u 0 d 0 0 0	-0.054441
	2 2 u 2 2 d u d 0 0 0 0	0.103563
	U 2 2 2 2 d u 0 0 d 0 0	0.063415
	2 2 2 2 0 2 2 0 0 0 0 0	-0.093955
	2 2 2 2 0 2 u 0 d 0 0 0	-0.070930
	2 2 2 2 0 2 0 0 2 0 0 0	-0.061981
	2 2 u 2 d 2 u 0 d 0 0 0	0.066358
	2 2 2 2 u u d d 0 0 0 0	0.056701

	2 2 2 2 u u d 0 d 0 0 0	-0.065360

Compound 1-F UB3LYP Triplet

Atomic Number	Atomic Type	Coordinates (Angstroms)		
		X	Y	Z
6	C	-2.438669	-0.204593	0.000160
6	C	-1.570081	-1.353445	0.000020
6	C	-0.183003	-1.428840	-0.000068
6	C	-2.010344	1.174271	0.000227
6	C	0.725435	-0.368758	-0.000037
6	C	-0.737510	1.724056	0.000180
6	C	0.493639	1.054901	0.000067
1	H	-2.085425	-2.310248	-0.000035
1	H	0.245007	-2.428303	-0.000207
1	H	-2.824248	1.894486	0.000342
1	H	-0.689191	2.810490	0.000289
6	C	2.165507	-0.505495	-0.000187
6	C	2.803487	0.741243	-0.000158
6	C	1.790781	1.699283	0.000101
1	H	1.938481	2.772672	0.000170
1	H	3.871936	0.908096	-0.000285
6	C	-3.811601	-0.446973	0.000233
1	H	-4.206177	-1.457339	0.000196
1	H	-4.529055	0.366502	0.000346
9	F	2.768092	-1.674473	-0.000448

Sum of Electronic and Thermal Free Energies = -523.400399

Compound 1-F CBS-QB3 Triplet

Atomic Number	Atomic Type	Coordinates (Angstroms)		
		X	Y	Z
6	C	-2.431842	-0.204507	0.000169
6	C	-1.564430	-1.351069	-0.000019
6	C	-0.181789	-1.424622	-0.000140
6	C	-2.005029	1.172240	0.000270
6	C	0.723722	-0.366536	-0.000111
6	C	-0.736624	1.721291	0.000222
6	C	0.491908	1.054132	0.000054
1	H	-2.079507	-2.305946	-0.000062
1	H	0.248295	-2.421416	-0.000258
1	H	-2.819066	1.889637	0.000399
1	H	-0.688708	2.805964	0.000303
6	C	2.160785	-0.507281	-0.000221
6	C	2.795500	0.738813	-0.000103
6	C	1.787039	1.695978	-0.000048
1	H	1.936447	2.767013	0.000030
1	H	3.861933	0.905570	-0.000110
6	C	-3.799990	-0.446913	0.000260
1	H	-4.192623	-1.455840	0.000190
1	H	-4.516209	0.364988	0.000398
9	F	2.757104	-1.671014	-0.000317

CBS-QB3 Free Energy = -522.620929
Thermal Correction to Gibbs Free Energy = 0.117911
CASPT2 Energy = -522.427732

Occupation		
A1	A2	Coefficient
	2 2 2 2 2 u u 0 0 0 0 0	-0.887288
	2 2 2 2 u d u 0 u 0 0 0	-0.057500
	2 2 2 2 u d u 0 0 u 0 0	0.057058
	2 2 2 u 2 d u u 0 0 0 0	-0.100542
	2 2 u 2 2 d u 0 0 u 0 0	-0.054149
	2 2 2 2 u u d 0 u 0 0 0	0.148084
	2 2 2 2 u u u 0 d 0 0 0	0.087667
	2 2 2 u 2 u u d 0 0 0 0	0.070480
	2 2 u 2 2 u d 0 0 u 0 0	-0.059851
	2 2 2 2 0 u u 0 2 0 0 0	0.091574
	2 2 2 0 2 u u 2 0 0 0 0	0.079796

Compound 1-CHO Singlet

Atomic Number	Atomic Type	Coordinates (Angstroms)		
		X	Y	Z
6	C	2.543123	-0.506524	0.000002
6	C	1.458846	-1.462076	0.000004
6	C	0.088338	-1.263949	0.000002
6	C	2.449551	0.937369	-0.000006
6	C	-0.603114	-0.046574	-0.000003
6	C	1.333202	1.743428	-0.000013
6	C	-0.022370	1.364662	-0.000011
1	H	1.776829	-2.501322	0.000009
1	H	-0.539075	-2.150796	0.000007
1	H	3.406237	1.452069	-0.000006
1	H	1.516671	2.815298	-0.000018
6	C	-2.006849	0.098440	-0.000004
6	C	-2.293249	1.486007	-0.000014
6	C	-1.099884	2.244180	-0.000009
1	H	-1.035677	3.324378	-0.000009
1	H	-3.294294	1.905898	-0.000013
6	C	3.813077	-1.041513	0.000010
1	H	3.978070	-2.115116	0.000018
1	H	4.696807	-0.409648	0.000013
6	C	-3.031081	-0.964490	0.000002
8	O	-2.775583	-2.156030	0.000027
1	H	-4.078442	-0.606271	0.000026

Uncorrected Sum of Electronic and Thermal Free Energies = -537.469497
Corrected Sum of Electronic and Thermal Free Energies = -537.470400

Compound 1-CHO Triplet

C	-2.590595	-0.469144	-0.000003
C	-1.512951	-1.424446	-0.000006
C	-0.135972	-1.243527	-0.000005
C	-2.429649	0.960988	0.000009
C	0.576421	-0.035313	-0.000002
C	-1.277839	1.732594	0.000014
C	0.058102	1.303533	0.000008
H	-1.835674	-2.462590	-0.000010
H	0.469476	-2.144410	-0.000008
H	-3.361546	1.520725	0.000014
H	-1.430482	2.809268	0.000024
C	2.038821	0.089627	-0.000006
C	2.373166	1.455656	0.000002
C	1.197125	2.204751	0.000012
H	1.124692	3.286135	0.000021
H	3.380095	1.854826	0.000002
C	-3.894709	-0.967324	-0.000011
H	-4.090479	-2.034144	-0.000020
H	-4.752581	-0.303581	-0.000009
C	3.031913	-1.002994	-0.000014
O	2.725452	-2.183614	0.000002
H	4.089890	-0.683712	-0.000005

Sum of Electronic and Thermal Free Energies = -537.470046

Compound 1-CF_3 Singlet

Atomic Number	Atomic Type	Coordinates (Angstroms)		
		X	Y	Z
6	C	3.080009	-0.685492	0.000120
6	C	3.151223	0.758747	0.000188
6	C	2.133059	1.689054	0.000200
6	C	1.894576	-1.513526	0.000047
6	C	0.745971	1.469515	0.000144
6	C	0.558974	-1.158997	0.000014
6	C	0.005294	0.129279	0.000048
1	H	4.160166	1.161290	0.000237
1	H	2.438792	2.732634	0.000257
1	H	2.092986	-2.581716	0.000011
6	C	-0.224785	2.465736	0.000159
1	H	-0.040284	3.531793	0.000223
6	C	-1.497106	1.850806	0.000075
6	C	-1.357984	0.443785	0.000005
1	H	-2.447131	2.372788	0.000060
1	H	-0.147177	-1.984024	-0.000034
6	C	4.283396	-1.356436	0.000134
1	H	5.230829	-0.824922	0.000230
1	H	4.330215	-2.441669	0.000132
6	C	-2.496589	-0.536556	-0.000183
9	F	-2.451980	-1.343766	1.087966
9	F	-3.681786	0.097634	0.000229
9	F	-2.452303	-1.342942	-1.088954

Uncorrected Sum of Electronic and Thermal Free Energies = -761.201817
Corrected Sum of Electronic and Thermal Free Energies = -761.202912

Compound 1-CF_3 Triplet

Atomic Number	Atomic Type	Coordinates (Angstroms)		
		X	Y	Z
6	C	3.120483	-0.659202	0.000105
6	C	3.135267	0.780202	0.000218
6	C	2.086070	1.688431	0.000185
6	C	1.934360	-1.477407	-0.000073
6	C	0.709176	1.427444	0.000105
6	C	0.591235	-1.127558	-0.000089
6	C	0.031744	0.157854	0.000007
1	H	4.128526	1.221842	0.000335
1	H	2.370700	2.737964	0.000251
1	H	2.127925	-2.547030	-0.000193
6	C	-0.315010	2.453819	0.000128
1	H	-0.119645	3.519711	0.000178
6	C	-1.578045	1.848238	0.000061
6	C	-1.394713	0.464379	-0.000016
1	H	-2.532963	2.356547	0.000068
1	H	-0.105514	-1.960226	-0.000190
6	C	4.352737	-1.312901	0.000165
1	H	5.285382	-0.759086	0.000315
1	H	4.417165	-2.395668	0.000067
6	C	-2.497583	-0.561730	-0.000134
9	F	-2.403863	-1.356499	1.090743
9	F	-3.706186	0.016699	0.000004
9	F	-2.403940	-1.356141	-1.091280

Sum of Electronic and Thermal Free Energies = -761.201871

Compound 1-CN UB3LYP Singlet

Atomic Number	Atomic Type	Coordinates (Angstroms)		
		X	Y	Z
6	C	-2.539672	-0.532487	0.000114
6	C	-1.472611	-1.508031	0.000237
6	C	-0.103634	-1.319503	0.000154
6	C	-2.415312	0.909398	0.000125
6	C	0.603519	-0.110233	0.000008
6	C	-1.283733	1.704032	0.000136
6	C	0.058661	1.301147	0.000073
1	H	-1.805234	-2.542169	0.000357
1	H	0.505070	-2.221092	0.000187
1	H	-3.362076	1.442568	0.000119
1	H	-1.453172	2.778008	0.000172
6	C	2.003465	0.014306	-0.000128
6	C	2.334393	1.405545	-0.000130
6	C	1.162410	2.170504	0.000008
1	H	1.112021	3.251394	0.000038
1	H	3.346800	1.792572	-0.000215
6	C	-3.826322	-1.027317	-0.000064
1	H	-4.024130	-2.095335	-0.000094
1	H	-4.689938	-0.368682	-0.000211
6	C	2.927057	-1.061082	-0.000218
7	N	3.668762	-1.959277	-0.000321

Uncorrected Sum of Electronic and Thermal Free Energies = -516.390104
Corrected Sum of Electronic and Thermal Free Energies = -516.391363

Compound 1-CN CBS-QB3 Singlet

Atomic Number	Atomic Type	Coordinates (Angstroms)		
		X	Y	Z
6	C	-2.522782	-0.535842	0.000073
6	C	-1.459131	-1.511271	0.000057
6	C	-0.097251	-1.324302	0.000012
6	C	-2.412751	0.902882	0.000073
6	C	0.608640	-0.115889	-0.000017
6	C	-1.292062	1.702521	0.000059
6	C	0.050701	1.309984	0.000019
1	H	-1.794553	-2.542514	0.000078
1	H	0.513727	-2.222378	-0.000002
1	H	-3.363420	1.424950	0.000090
1	H	-1.466859	2.773899	0.000072
6	C	1.993748	0.012211	-0.000068
6	C	2.313521	1.405392	-0.000063
6	C	1.141377	2.172767	-0.000017
1	H	1.093616	3.251301	-0.000009
1	H	3.323200	1.795754	-0.000100
6	C	-3.802937	-1.036029	0.000088
1	H	-3.994596	-2.103525	0.000095
1	H	-4.668400	-0.382516	0.000095
6	C	2.925152	-1.053757	-0.000114
7	N	3.668563	-1.938138	-0.000135

CBS-QB3 Free Energy = -515.569797
Thermal Correction to Gibbs Free Energy = 0.124428
CASPT2 Energy = -515.362332

Occupation		
A1	A2	Coefficient
	2 2 2 2 2 2 2 0 0 0 0 0 0 0	-0.860843
	2 2 2 2 2 2 0 2 0 0 0 0 0 0	0.149221
	2 2 2 2 2 2 0 0 2 0 0 0 0 0	0.069954
	2 2 2 2 u d u d 0 0 0 0 0	0.108214
	2 2 u 2 2 d u d 0 0 0 0 0	0.061330
	2 2 2 2 0 2 2 0 0 0 0 0 0	0.118791
	2 2 2 2 0 2 0 2 0 0 0 0 0	0.053186
	2 2 u 2 d 2 u 0 d 0 0 0 0	-0.051464
	2 2 2 2 u u d d 0 0 0 0 0	0.104497
	2 2 2 2 u u 0 d d 0 0 0 0	-0.053136

Compound 1-CN UB3LYP Triplet

Atomic Number	Atomic Type	Coordinates (Angstroms)		
		X	Y	Z
6	C	-2.574734	-0.504345	0.000057
6	C	-1.507372	-1.470512	0.000019
6	C	-0.130749	-1.290974	0.000007
6	C	-2.403284	0.927346	0.000002
6	C	0.579210	-0.085764	0.000015
6	C	-1.250360	1.695914	-0.000032
6	C	0.084548	1.261103	-0.000019
1	H	-1.836289	-2.506498	-0.000012
1	H	0.466380	-2.200208	-0.000034
1	H	-3.333089	1.490391	0.000002
1	H	-1.399126	2.773035	-0.000034
6	C	2.039406	0.020003	-0.000025
6	C	2.403380	1.384341	-0.000098
6	C	1.236526	2.141353	0.000062
1	H	1.182234	3.223515	0.000083
1	H	3.417853	1.760218	-0.000167
6	C	-3.881352	-0.993374	0.000145
1	H	-4.085015	-2.058761	0.000193
1	H	-4.734772	-0.323867	0.000188
6	C	2.913027	-1.085471	-0.000064
7	N	3.610335	-2.022219	-0.000089

Sum of Electronic and Thermal Free Energies = -516.392620

Compound 1-CN CBS-QB3 Triplet

Atomic Number	Atomic Type	Coordinates (Angstroms)		
		X	Y	Z
6	C	-2.568380	-0.502512	0.000071
6	C	-1.503557	-1.467519	0.000038
6	C	-0.131119	-1.289037	-0.000001
6	C	-2.397044	0.926549	0.000074
6	C	0.577698	-0.086741	-0.000016
6	C	-1.247566	1.692435	0.000045
6	C	0.084092	1.257727	0.000005
1	H	-1.833772	-2.501226	0.000043
1	H	0.465636	-2.196529	-0.000023
1	H	-3.325987	1.487601	0.000103
1	H	-1.395268	2.767917	0.000055
6	C	2.035322	0.017731	-0.000059
6	C	2.397967	1.379363	-0.000063
6	C	1.234829	2.135680	-0.000024
1	H	1.181369	3.215812	-0.000017
1	H	3.410569	1.754366	-0.000091
6	C	-3.870862	-0.989597	0.000105
1	H	-4.073820	-2.053051	0.000105
1	H	-4.722123	-0.320600	0.000131
6	C	2.906247	-1.083619	-0.000090
7	N	3.598234	-2.013864	-0.000116

CBS-QB3 Free Energy = -515.571430
Thermal Correction to Gibbs Free Energy = 0.122542
CASPT2 Energy = -515.355907

Occupation		
A1	A2	Coefficient
	2 2 2 2 2 u u 0 0 0 0 0 0	-0.869734
	2 2 2 2 u d u 0 0 u 0 0 0	0.056563
	2 2 2 u 2 d u u 0 0 0 0 0	-0.079887
	2 2 u 2 2 d u 0 0 u 0 0 0	-0.071797
	2 2 2 2 u u d 0 u 0 0 0 0	0.142706
	2 2 2 2 u u u 0 d 0 0 0 0	0.069403
	2 2 2 u 2 u u d 0 0 0 0 0	0.053712
	2 2 2 2 0 u u 0 2 0 0 0 0	0.082115
	2 2 2 0 2 u u 2 0 0 0 0 0	0.064919

Compound 1-NO_2 Singlet

Atomic Number	Atomic Type	Coordinates (Angstroms)		
		X	Y	Z
6	C	2.858157	-0.551330	0.000076
6	C	2.798399	0.891798	0.000581
6	C	1.698453	1.722267	0.000155
6	C	1.753867	-1.482666	-0.000714
6	C	0.336418	1.378360	-0.000260
6	C	0.390824	-1.259684	-0.000617
6	C	-0.284094	-0.028457	-0.000149
1	H	3.765294	1.387208	0.001251
1	H	1.904489	2.789977	0.000270
1	H	2.049607	-2.528295	-0.001356
6	C	-0.710640	2.297148	-0.000443
1	H	-0.606598	3.373946	-0.000539
6	C	-1.924275	1.590283	-0.000249
6	C	-1.660736	0.194956	0.000030
1	H	-2.921535	2.013680	-0.000238
1	H	-0.248172	-2.135977	-0.000943
6	C	4.119227	-1.108937	0.000361
1	H	5.014354	-0.492959	0.001076
1	H	4.265218	-2.185624	-0.000214
7	N	-2.728864	-0.785530	0.000285
8	O	-3.875405	-0.343204	0.000419
8	O	-2.421371	-1.980007	0.000338

Uncorrected Sum of Electronic and Thermal Free Energies = -628.644325
Corrected Sum of Electronic and Thermal Free Energies = -628.644325

Compound 1-NO_2 Triplet

Atomic Number	Atomic Type	Coordinates (Angstroms)		
		X	Y	Z
6	C	2.894173	-0.538274	-0.043420
6	C	2.786916	0.895872	0.008087
6	C	1.663317	1.708253	0.041565
6	C	1.781049	-1.451580	-0.068203
6	C	0.312567	1.331358	0.038306
6	C	0.411685	-1.221960	-0.040526
6	C	-0.253992	0.010605	0.010005
1	H	3.738324	1.421543	0.019582
1	H	1.854499	2.778209	0.075434
1	H	2.063429	-2.500416	-0.111952
6	C	-0.787479	2.278961	0.068030
1	H	-0.669839	3.355969	0.094797
6	C	-2.001533	1.588076	0.050252
6	C	-1.698482	0.227002	0.020158
1	H	-2.998057	2.007963	0.056181
1	H	-0.220138	-2.103551	-0.049022
6	C	4.177668	-1.086189	-0.072205
1	H	5.060801	-0.456579	-0.055301
1	H	4.332097	-2.159125	-0.111769
7	N	-2.722281	-0.805235	0.000977
8	O	-3.819677	-0.480745	-0.437858
8	O	-2.407884	-1.914269	0.438223

Sum of Electronic and Thermal Free Energies = -628.642691

Compound 2 Singlet

Atomic Number	Atomic Type	Coordinates (Angstroms)		
		X	Y	Z
6	C	2.708558	-0.543078	-0.000077
6	C	2.477476	0.840239	-0.000055
6	C	1.269594	1.523267	-0.000016
6	C	1.786167	-1.580530	-0.000065
6	C	-0.026579	0.993408	0.000012
6	C	0.385228	-1.512759	-0.000028
6	C	-0.419349	-0.389029	0.000006
1	H	3.751807	-0.841516	-0.000108
1	H	3.367371	1.461351	-0.000071
1	H	1.335169	2.607170	-0.000005
1	H	2.203471	-2.582076	-0.000087
1	H	-0.122089	-2.472278	-0.000026
6	C	-1.228100	1.800649	0.000053
1	H	-1.228462	2.881467	0.000066
6	C	-2.319389	0.993028	0.000068
6	C	-1.899126	-0.400762	0.000045
1	H	-3.352474	1.312061	0.000097
6	C	-2.707509	-1.476548	0.000052
1	H	-3.785087	-1.360831	0.000184
1	H	-2.331548	-2.492654	-0.000020

CBS-QB3 Free Energy = -423.505591

Compound 2 Triplet

Atomic Number	Atomic Type	Coordinates (Angstroms)		
		X	Y	Z
6	C	2.700006	-0.597156	-0.000078
6	C	2.533498	0.776769	-0.000054
6	C	1.319803	1.504538	-0.000013
6	C	1.731553	-1.621918	-0.000069
6	C	0.030279	1.011582	0.000012
6	C	0.331613	-1.520266	-0.000032
6	C	-0.439748	-0.376085	0.000004
1	H	3.729125	-0.942373	-0.000109
1	H	3.439353	1.372229	-0.000068
1	H	1.417531	2.586475	0.000000
1	H	2.125990	-2.632095	-0.000093
1	H	-0.198920	-2.467298	-0.000032
6	C	-1.130756	1.833379	0.000050
1	H	-1.128624	2.915560	0.000065
6	C	-2.293722	1.005238	0.000074
6	C	-1.914435	-0.336586	0.000042
1	H	-3.311936	1.368362	0.000109
6	C	-2.818676	-1.429119	0.000054
1	H	-3.884464	-1.245894	0.000130
1	H	-2.484557	-2.457224	0.000049

CBS-QB3 Free Energy = -423.432496

Compound 2a Singlet

Atomic Number	Atomic Type	Coordinates (Angstroms)		
		X	Y	Z
6	C	2.715720	-0.544839	-0.000082
6	C	2.483882	0.842106	-0.000062
6	C	1.272319	1.526687	-0.000021
6	C	1.790149	-1.584949	-0.000067
6	C	-0.027058	0.995664	0.000009
6	C	0.385686	-1.516629	-0.000026
6	C	-0.421048	-0.389655	0.000008
1	H	3.760637	-0.844076	-0.000114
1	H	3.374800	1.464827	-0.000079
1	H	1.337955	2.612330	-0.000009
1	H	2.207368	-2.588464	-0.000090
1	H	-0.122296	-2.477658	-0.000021
6	C	-1.229598	1.804919	0.000059
1	H	-1.229485	2.887800	0.000072
6	C	-2.324172	0.995146	0.000078
6	C	-1.903083	-0.400697	0.000049
1	H	-3.359264	1.314672	0.000112
6	C	-2.715248	-1.479376	0.000055
1	H	-3.794565	-1.361732	0.000099
1	H	-2.340453	-2.497957	0.000032

Uncorrected Sum of Electronic and Thermal Free Energies = -424.199338
Corrected Sum of Electronic and Thermal Free Energies = -424.199338

Compound 2a Triplet

Atomic Number	Atomic Type	Coordinates (Angstroms)		
		X	Y	Z
6	C	2.706609	-0.599124	-0.000084
6	C	2.539162	0.779170	-0.000066
6	C	1.322690	1.508480	-0.000022
6	C	1.735669	-1.625820	-0.000063
6	C	0.029373	1.014212	0.000011
6	C	0.331871	-1.523025	-0.000023
6	C	-0.441266	-0.375722	0.000009
1	H	3.737539	-0.944688	-0.000117
1	H	3.446232	1.376092	-0.000087
1	H	1.420970	2.592159	-0.000013
1	H	2.129484	-2.638180	-0.000082
1	H	-0.199336	-2.471581	-0.000015
6	C	-1.134187	1.837520	0.000056
1	H	-1.132620	2.921721	0.000071
6	C	-2.299607	1.007071	0.000080
6	C	-1.918133	-0.337281	0.000050
1	H	-3.319943	1.370270	0.000113
6	C	-2.822813	-1.433831	0.000055
1	H	-3.890949	-1.252008	0.000111
1	H	-2.487589	-2.463688	0.000001

Sum of Electronic and Thermal Free Energies = -424.134599

Compound 2b Singlet

Atomic Number	Atomic Type	Coordinates (Angstroms)		
		X	Y	Z
6	C	2.708486	-0.599871	0.000002
6	C	1.765641	-1.633378	-0.000017
6	C	0.336511	-1.511185	0.000003
6	C	2.544286	0.791798	0.000015
6	C	-0.456821	-0.399650	0.000028
6	C	1.312532	1.516579	-0.000012
6	C	0.021552	1.046642	-0.000012
1	H	3.749438	-0.933380	0.000007
1	H	2.155929	-2.649192	-0.000039
1	H	-0.196650	-2.463964	-0.000004
1	H	3.453389	1.390335	0.000031
1	H	1.411429	2.605235	-0.000029
6	C	-1.925260	-0.383656	0.000042
6	C	-2.280994	1.023900	-0.000011
1	H	-3.308928	1.377291	-0.000037
6	C	-1.160143	1.835989	-0.000011
1	H	-1.165122	2.923022	-0.000028
6	C	-2.818449	-1.439832	0.000055
1	H	-3.889628	-1.257529	-0.000092
1	H	-2.493898	-2.475836	-0.000301

Uncorrected Sum of Electronic and Thermal Free Energies = -424.451277
Corrected Sum of Electronic and Thermal Free Energies = -424.451277

Compound 2b Triplet

Atomic Number	Atomic Type	Coordinates (Angstroms)		
		X	Y	Z
6	C	2.731778	-0.577157	0.000017
6	C	1.764086	-1.611495	-0.000019
6	C	0.373409	-1.532157	-0.000034
6	C	2.510322	0.825486	0.000018
6	C	-0.449467	-0.367864	-0.000007
6	C	1.315074	1.531006	0.000001
6	C	-0.021914	1.012694	-0.000001
1	H	3.773724	-0.896034	0.000037
1	H	2.172059	-2.624121	-0.000037
1	H	-0.153219	-2.485645	-0.000063
1	H	3.415293	1.436353	0.000028
1	H	1.393326	2.619168	-0.000007
6	C	-1.907522	-0.377666	0.000026
6	C	-2.328549	1.010730	-0.000015
1	H	-3.363016	1.339651	-0.000026
6	C	-1.197567	1.819739	-0.000010
1	H	-1.189881	2.906729	-0.000020
6	C	-2.759529	-1.476298	0.000031
1	H	-3.838062	-1.340850	-0.000053
1	H	-2.390950	-2.497345	0.000098

Sum of Electronic and Thermal Free Energies = -424.451005

Compound 3 Singlet

Atomic Number	Atomic Type	Coordinates (Angstroms)		
		X	Y	Z
6	C	-2.878204	-0.000114	-0.000015
6	C	-2.300593	-1.279752	-0.000002
6	C	-0.958526	-1.611160	0.000001
6	C	-2.300459	1.279528	0.000002
6	C	0.161871	-0.748437	-0.000001
6	C	-0.958558	1.611259	0.000028
6	C	0.162074	0.748817	0.000017
1	H	-3.964930	-0.000037	-0.000037
1	H	-2.999691	-2.108141	0.000006
1	H	-0.725671	-2.672642	0.000028
1	H	-2.999629	2.107889	-0.000004
1	H	-0.726310	2.672881	0.000046
6	C	1.471500	-1.173985	0.000039
1	H	1.817348	-2.197645	0.000082
6	C	2.326209	0.000249	-0.000107
6	C	1.471861	1.174235	0.000007
1	H	1.817552	2.197991	0.000010
6	C	3.683426	-0.000426	-0.000157
1	H	4.248269	-0.926384	0.000502
1	H	3.078077	0.306213	0.000083

CBS-QB3 Free Energy = -423.472143

Compound 3 Triplet

Atomic Number	Atomic Type	Coordinates (Angstroms)		
		X	Y	Z
6	C	-2.925756	0.000000	-0.000089
6	C	-2.320622	-1.259705	-0.000067
6	C	-0.962692	-1.592254	-0.000025
6	C	-2.320623	1.259705	-0.000074
6	C	0.129560	-0.724803	0.000006
6	C	-0.962692	1.592253	-0.000033
6	C	0.129559	0.724803	0.000002
1	H	-4.010167	-0.000001	-0.000122
1	H	-3.003434	-2.103450	-0.000085
1	H	-0.731612	-2.652715	-0.000015
1	H	-3.003434	2.103450	-0.000096
1	H	-0.731612	2.652714	-0.000028
6	C	1.499264	-1.144045	0.000047
1	H	1.826091	-2.175523	0.000060
6	C	2.362307	0.000001	0.000072
6	C	1.499263	1.144046	0.000042
1	H	1.826088	2.175525	0.000050
6	C	3.742930	0.000000	0.000115
1	H	4.302547	-0.926452	0.000134
1	H	4.302550	0.926449	0.000130

CBS-QB3 Free Energy = -423.453016

Compound 3a Singlet

Atomic Number	Atomic Type	Coordinates (Angstroms)		
		X	Y	Z
6	C	2.886947	0.000000	-0.000087
6	C	2.306611	1.282430	-0.000066
6	C	0.960241	1.613951	-0.000025
6	C	2.306611	-1.282430	-0.000073
6	C	-0.162409	0.749207	0.000006
6	C	0.960241	-1.613951	-0.000033
6	C	-0.162409	-0.749207	0.000002
1	H	3.975285	0.000000	-0.000119
1	H	3.005541	2.113232	-0.000085
1	H	0.727519	2.677162	-0.000015
1	H	3.005541	-2.113232	-0.000096
1	H	0.727519	-2.677162	-0.000029
6	C	-1.475867	1.176202	0.000044
1	H	-1.822179	2.202043	0.000056
6	C	-2.331284	0.000000	0.000072
6	C	-1.475867	-1.176202	0.000039
1	H	-1.822179	-2.202043	0.000047
6	C	-3.692706	0.000000	0.000117
1	H	-4.258845	0.927459	0.000135
1	H	-4.258845	-0.927459	0.000131

Uncorrected Sum of Electronic and Thermal Free Energies = -424.170961
Corrected Sum of Electronic and Thermal Free Energies = -424.170961

Compound 3a Triplet

Atomic Number	Atomic Type	Coordinates (Angstroms)		
		X	Y	Z
6	C	2.933323	0.000000	-0.000089
6	C	2.326197	1.263097	-0.000067
6	C	0.964531	1.596132	-0.000025
6	C	2.326198	-1.263097	-0.000074
6	C	-0.130041	0.726156	0.000006
6	C	0.964531	-1.596131	-0.000033
6	C	-0.130041	-0.726156	0.000002
1	H	4.019605	0.000000	-0.000122
1	H	3.009504	2.108696	-0.000086
1	H	0.732793	2.658311	-0.000015
1	H	3.009504	-2.108696	-0.000097
1	H	0.732793	-2.658311	-0.000028
6	C	-1.502446	1.146006	0.000048
1	H	-1.830307	2.179382	0.000061
6	C	-2.367340	0.000000	0.000072
6	C	-1.502446	-1.146006	0.000042
1	H	-1.830306	-2.179383	0.000050
6	C	-3.752083	0.000000	0.000115
1	H	-4.312939	0.928085	0.000134
1	H	-4.312940	-0.928084	0.000130

Sum of Electronic and Thermal Free Energies = -424.157187

Compound 3b Singlet

Atomic Number	Atomic Type	Coordinates (Angstroms)		
		X	Y	Z
6	C	2.949079	0.000008	-0.000012
6	C	2.326499	1.263360	-0.000028
6	C	0.975646	1.601718	-0.000022
6	C	2.326513	-1.263327	0.000011
6	C	-0.165584	0.751964	-0.000003
6	C	0.975651	-1.601718	0.000022
6	C	-0.165587	-0.752000	0.000016
1	H	4.038491	0.000023	-0.000017
1	H	3.011494	2.113776	-0.000040
1	H	0.751030	2.669357	-0.000025
1	H	3.011502	-2.113749	0.000019
1	H	0.751084	-2.669367	0.000031
6	C	-1.488022	1.152858	0.000024
6	C	-2.375657	-0.000026	-0.000024
6	C	-1.488038	-1.152898	0.000013
1	H	-1.827342	-2.185111	0.000024
6	C	-3.749990	0.000036	-0.000015
1	H	-1.827351	2.185063	0.000048
1	H	-4.316058	-0.927883	0.000035
1	H	-4.315915	0.928046	0.000029

Uncorrected Sum of Electronic and Thermal Free Energies = -424.476716
Corrected Sum of Electronic and Thermal Free Energies = -424.476716

Compound 3b Triplet

Atomic Number	Atomic Type	Coordinates (Angstroms)		
		X	Y	Z
6	C	-2.943282	-0.000001	-0.000020
6	C	-2.327864	-1.285151	-0.000027
6	C	-0.985176	-1.613415	-0.000019
6	C	-2.327865	1.285151	-0.000003
6	C	0.149991	-0.721971	-0.000002
6	C	-0.985177	1.613414	0.000011
6	C	0.149990	0.721972	0.000012
1	H	-4.033082	-0.000001	-0.000029
1	H	-3.022758	-2.126840	-0.000039
1	H	-0.743321	-2.677049	-0.000023
1	H	-3.022759	2.126840	-0.000001
1	H	-0.743322	2.677048	0.000023
6	C	1.498504	-1.144540	0.000016
6	C	2.358618	0.000002	0.000009
6	C	1.498504	1.144542	0.000026
1	H	1.832782	2.178678	0.000039
6	C	3.781813	-0.000001	0.000000
1	H	1.832785	-2.178674	0.000017
1	H	4.345670	0.928832	0.000000
1	H	4.345662	-0.928839	-0.000005

Sum of Electronic and Thermal Free Energies = -424.435846

Compound 4 Singlet

Atomic Number	Atomic Type	Coordinates (Angstroms)		
		X	Y	Z
6	C	2.380310	-0.767021	0.000104
6	C	1.526505	-1.875582	-0.000007
6	C	0.120570	-1.948389	-0.000093
6	C	2.062704	0.576373	0.000105
6	C	-0.779682	-0.910465	-0.000060
6	C	0.786655	1.244029	-0.000008
6	C	-0.515381	0.590466	0.000027
1	H	3.439882	-0.997502	0.000174
1	H	2.031131	-2.837406	-0.000020
1	H	-0.299601	-2.949010	-0.000166
1	H	2.913784	1.248842	0.000158
1	C	-2.193119	-1.087495	-0.000087
1	H	-2.696033	-2.042544	-0.000175
6	C	-2.772644	0.152977	0.000097
1	H	-3.834401	0.359791	0.000166
6	C	-1.739974	1.180845	0.000136
1	H	-1.957540	2.238621	0.000276
6	C	0.881395	2.613519	-0.000234
1	H	1.846600	3.104635	-0.000021
1	H	0.012148	3.259037	-0.000276

CBS-QB3 Free Energy = -423.452562

Compound 4 Triplet

Atomic Number	Atomic Type	Coordinates (Angstroms)		
		X	Y	Z
6	C	2.380310	-0.767021	0.000104
6	C	1.526505	-1.875582	-0.000007
6	C	0.120570	-1.948389	-0.000093
6	C	2.062704	0.576373	0.000105
6	C	-0.779682	-0.910465	-0.000060
6	C	0.786655	1.244029	-0.000008
6	C	-0.515381	0.590466	0.000027
1	H	3.439882	-0.997502	0.000174
1	H	2.031131	-2.837406	-0.000020
1	H	-0.299601	-2.949010	-0.000166
1	H	2.913784	1.248842	0.000158
6	C	-2.193119	-1.087495	-0.000087
1	H	-2.696033	-2.042544	-0.000175
6	C	-2.772644	0.152977	0.000097
1	H	-3.834401	0.359791	0.000166
6	C	-1.739974	1.180845	0.000136
1	H	-1.957540	2.238621	0.000276
6	C	0.881395	2.613519	-0.000234
1	H	1.846600	3.104635	-0.000021
1	H	0.012148	3.259037	-0.000276

CBS-QB3 Free Energy = -423.450713

Compound 4a Singlet

Atomic Number	Atomic Type	Coordinates (Angstroms)		
		X	Y	Z
6	C	2.403721	-0.716930	0.000068
6	C	1.578818	-1.854907	0.000028
6	C	0.173887	-1.949642	-0.000012
6	C	2.048328	0.620001	0.000028
6	C	-0.744515	-0.917823	0.000002
6	C	0.752440	1.257440	-0.000081
6	C	-0.516485	0.563957	-0.000015
1	H	3.471498	-0.916788	0.000125
1	H	2.105687	-2.806302	0.000055
1	H	-0.233708	-2.957447	-0.000057
1	H	2.883737	1.315021	0.000072
6	C	-2.166910	-1.127686	-0.000111
1	H	-2.645538	-2.098210	-0.000217
6	C	-2.784698	0.101611	0.000098
1	H	-3.852761	0.281028	0.000147
6	C	-1.780422	1.138330	0.000119
1	H	-2.011458	2.195496	0.000226
6	C	0.804489	2.645195	-0.000178
1	H	1.755858	3.166037	0.000072
1	H	-0.085225	3.263890	-0.000092

Uncorrected Sum of Electronic and Thermal Free Energies = -424.149039
Corrected Sum of Electronic and Thermal Free Energies = -424.151683

Compound 4a Triplet

Atomic Number	Atomic Type	Coordinates (Angstroms)		
		X	Y	Z
6	C	2.452286	-0.550454	0.000029
6	C	1.729788	-1.760734	-0.000020
6	C	0.344686	-1.934754	-0.000042
6	C	1.992795	0.753949	0.000041
6	C	-0.642140	-0.947327	-0.000019
6	C	0.654277	1.297536	0.000007
6	C	-0.529873	0.491278	0.000008
1	H	3.533763	-0.657294	0.000056
1	H	2.325486	-2.669249	-0.000037
1	H	-0.010624	-2.962567	-0.000075
1	H	2.772528	1.511317	0.000075
6	C	-2.060161	-1.253406	-0.000024
1	H	-2.468791	-2.257299	-0.000049
6	C	-2.793107	-0.056562	0.000012
1	H	-3.871875	0.027353	0.000027
6	C	-1.893786	1.004259	0.000030
1	H	-2.171277	2.049525	0.000065
6	C	0.552333	2.694905	-0.000022
1	H	1.444465	3.311078	-0.000013
1	H	-0.396259	3.214997	-0.000063

Sum of Electronic and Thermal Free Energies = -424.149785

Compound 4b Singlet

Atomic Number	Atomic Type	Coordinates (Angstroms)		
		X	Y	Z
6	C	2.412579	-0.626333	0.000022
6	C	1.655188	-1.841441	0.000057
6	C	0.282503	-1.949119	0.000048
6	C	2.014631	0.685142	-0.000023
6	C	-0.693781	-0.921282	0.000014
6	C	0.697072	1.317267	-0.000050
6	C	-0.540218	0.524985	-0.000023
1	H	3.495272	-0.770679	0.000032
1	H	2.231903	-2.765626	0.000076
1	H	-0.122716	-2.963516	0.000039
1	H	2.831266	1.408538	-0.000034
6	C	-2.097374	-1.201366	-0.000090
6	C	-2.791235	0.009291	0.000074
6	C	-1.837820	1.064426	0.000019
1	H	-2.082508	2.120595	0.000048
1	H	-2.525464	-2.199752	-0.000158
6	C	0.678453	2.691523	-0.000060
1	H	-3.870553	0.132378	0.000137
1	H	1.606032	3.258070	-0.000014
1	H	-0.243216	3.261440	-0.000066

Uncorrected Sum of Electronic and Thermal Free Energies = -424.480438
Corrected Sum of Electronic and Thermal Free Energies = -424.480438

Compound 4b Triplet

Atomic Number	Atomic Type	Coordinates (Angstroms)		
		X	Y	Z
6	C	-2.472184	-0.569065	0.000013
6	C	-1.745826	-1.754251	0.000014
6	C	-0.351591	-1.958586	0.000002
6	C	-1.981076	0.783688	0.000001
6	C	0.681514	-0.970771	-0.000007
6	C	-0.672850	1.294447	-0.000009
6	C	0.558412	0.499056	-0.000009
1	H	-3.557424	-0.666570	0.000021
1	H	-2.346018	-2.667090	0.000019
1	H	-0.011850	-2.994110	-0.000007
1	H	-2.762682	1.544618	0.000005
6	C	2.058667	-1.267896	-0.000044
6	C	2.791722	-0.055110	0.000000
6	C	1.876800	1.015401	0.000014
1	H	2.152725	2.063282	0.000038
1	H	2.474036	-2.271271	-0.000071
6	C	-0.544703	2.715756	0.000003
1	H	3.873986	0.038039	0.000008
1	H	-1.431953	3.343097	-0.000123
1	H	0.415863	3.213996	0.000225

Sum of Electronic and Thermal Free Energies = -424.425880

Compound 5 Singlet

Atomic Number	Atomic Type	Coordinates (Angstroms)		
		X	Y	Z
6	C	-2.067343	0.901349	-0.000025
6	C	-1.151832	1.911564	-0.000005
6	C	0.269397	1.828293	0.000018
6	C	-1.894587	-0.542936	-0.000033
6	C	1.053965	0.701084	0.000019
6	C	-0.623828	-1.241939	-0.000019
6	C	0.631372	-0.718631	0.000002
1	H	-3.107054	1.213459	-0.000036
1	H	-1.555397	2.917843	-0.000006
1	H	0.794579	2.779486	0.000030
1	H	-0.698866	-2.325589	-0.000025
6	C	2.468968	0.694167	0.000010
1	H	3.089984	1.580199	0.000018
6	C	2.933664	-0.633301	0.000058
6	C	1.839175	-1.490325	-0.000007
1	H	1.878076	-2.571342	-0.000020
1	H	3.971224	-0.934175	0.000091
6	C	-3.023347	-1.309780	-0.000047
1	H	-4.011322	-0.864856	-0.000133
1	H	-2.974853	-2.392285	0.000254

CBS-QB3 Free Energy = -423.479357

Compound 5 Triplet

Atomic Number	Atomic Type	Coordinates (Angstroms)		
		X	Y	Z
6	C	2.065599	0.894637	0.000023
6	C	1.110232	1.91163	0.000021
6	C	-0.286224	1.836617	0.000008
6	C	1.910684	-0.514305	0.000011
6	C	-1.063484	0.684747	-0.000004
6	C	0.656664	-1.201756	-0.000003
6	C	-0.621012	-0.692925	-0.000009
1	H	3.094666	1.238827	0.000035
1	H	1.517749	2.917698	0.00003
1	H	-0.81758	2.78278	0.000008
1	H	0.729734	-2.285493	-0.00001
6	C	-2.521425	0.657758	-0.000018
1	H	-3.146767	1.539461	-0.000018
6	C	-2.950334	-0.663598	-0.000024
6	C	-1.83439	-1.507981	-0.000024
1	H	-1.845252	-2.589005	-0.000029
1	H	-3.980482	-0.989306	-0.000031
6	C	3.090091	-1.303064	0.000016
1	H	4.068599	-0.841583	0.000036
1	H	3.040919	-2.383937	-0.000001

CBS-QB3 Free Energy = -423.438674

Compound 5a Singlet

Atomic Number	Atomic Type	Coordinates (Angstroms)		
		X	Y	Z
6	C	-2.072158	0.903658	-0.000028
6	C	-1.152843	1.916722	-0.000011
6	C	0.271437	1.833078	0.000005
6	C	-1.899588	-0.543303	-0.000027
6	C	1.057616	0.702184	0.000008
6	C	-0.626543	-1.243559	-0.000002
6	C	0.632772	-0.719214	0.000004
1	H	-3.113107	1.217948	-0.000044
1	H	-1.556820	2.924743	-0.000016
1	H	0.797591	2.785774	0.000010
1	H	-0.701176	-2.329092	0.000011
6	C	2.475919	0.693973	0.000003
1	H	3.099196	1.580996	0.000008
6	C	2.940281	-0.636605	0.000049
6	C	1.841834	-1.494100	0.000012
1	H	1.879494	-2.577301	0.000019
1	H	3.979478	-0.939397	0.000079
6	C	-3.031906	-1.312650	-0.000032
1	H	-4.022034	-0.867587	0.000006
1	H	-2.983550	-2.397185	0.000036

Uncorrected Sum of Electronic and Thermal Free Energies = -424.175115
Corrected Sum of Electronic and Thermal Free Energies = -424.175115

Compound 5a Triplet

Atomic Number	Atomic Type	Coordinates (Angstroms)		
		X	Y	Z
6	C	-2.070517	0.897181	-0.000023
6	C	-1.112057	1.916628	-0.000021
6	C	0.288115	1.841106	-0.000008
6	C	-1.915117	-0.515013	-0.000011
6	C	1.066875	0.685974	0.000004
6	C	-0.658869	-1.204171	0.000003
6	C	0.622722	-0.693985	0.000009
1	H	-3.101023	1.242762	-0.000035
1	H	-1.519708	2.924485	-0.000031
1	H	0.820362	2.788829	-0.000008
1	H	-0.731703	-2.289661	0.000009
6	C	2.527159	0.658399	0.000018
1	H	3.154033	1.541595	0.000018
6	C	2.956866	-0.666430	0.000023
6	C	1.837183	-1.511296	0.000025
1	H	1.846844	-2.594442	0.000030
1	H	3.988704	-0.993557	0.000029
6	C	-3.097507	-1.306107	-0.000016
1	H	-4.078317	-0.844712	-0.000038
1	H	-3.048319	-2.389022	0.000002

Sum of Electronic and Thermal Free Energies = -424.141146

Compound 5b Singlet

Atomic Number	Atomic Type	Coordinates (Angstroms)		
		X	Y	Z
6	C	2.065246	0.924597	-0.000022
6	C	1.098540	1.915876	-0.000030
6	C	-0.312507	1.861424	-0.000015
6	C	1.956203	-0.520317	0.000001
6	C	-1.120364	0.719392	0.000000
6	C	0.659223	-1.174957	0.000015
6	C	-0.627810	-0.698681	0.000014
1	H	3.094644	1.285432	-0.000032
1	H	1.503972	2.930084	-0.000036
1	H	-0.830819	2.820791	0.000001
6	C	-2.529432	0.655271	0.000077
6	C	-2.921568	-0.712689	-0.000056
6	C	-1.806199	-1.539501	0.000010
1	H	-1.792000	-2.623474	0.000012
1	H	-3.195099	1.511846	0.000142
1	H	-3.953209	-1.057844	-0.000089
6	C	3.093697	-1.317087	-0.000003
1	H	4.088692	-0.881039	0.000017
1	H	3.022956	-2.400583	0.000028
1	H	0.730684	-2.265181	0.000017

Uncorrected Sum of Electronic and Thermal Free Energies = -424.461628
Corrected Sum of Electronic and Thermal Free Energies = -424.461628

Compound 5b Triplet

Atomic Number	Atomic Type	Coordinates (Angstroms)		
		X	Y	Z
6	C	2.080705	0.907749	0.000003
6	C	1.112220	1.924643	0.000019
6	C	-0.281313	1.848627	0.000015
6	C	1.922014	-0.537322	-0.000018
6	C	-1.093356	0.675990	0.000001
6	C	0.671953	-1.226958	-0.000019
6	C	-0.654445	-0.703535	-0.000010
1	H	3.116825	1.244525	0.000005
1	H	1.520671	2.937283	0.000026
1	H	-0.816800	2.799169	0.000007
6	C	-2.516514	0.667545	-0.000055
6	C	-2.970577	-0.666350	0.000057
6	C	-1.830301	-1.504266	0.000021
1	H	-1.835391	-2.591049	0.000045
1	H	-3.139611	1.558092	-0.000095
1	H	-4.005787	-0.992657	0.000103
6	C	3.102924	-1.293485	-0.000024
1	H	4.078832	-0.816430	0.000081
1	H	3.076227	-2.379674	-0.000088
1	H	0.745165	-2.315076	-0.000018

Sum of Electronic and Thermal Free Energies = -424.445603

Compound 6 Singlet

Atomic Number	Atomic Type	Coordinates (Angstroms)		
		X	Y	Z
6	C	-2.132871	0.000000	-0.000014
6	C	-1.501555	1.299499	-0.000017
6	C	-0.168224	1.630100	-0.000010
6	C	-1.501552	-1.299495	-0.000004
6	C	0.939426	0.772211	0.000001
6	C	-0.168217	-1.630097	0.000005
6	C	0.939425	-0.772205	0.000008
1	H	-2.195651	2.132783	-0.000027
1	H	0.062957	2.690979	-0.000012
1	H	-2.195644	-2.132783	-0.000003
1	H	0.062963	-2.690976	0.000011
6	C	2.265023	1.163742	0.000013
1	H	2.626269	2.181161	0.000016
6	C	3.069082	-0.000004	0.000015
6	C	2.265030	-1.163741	0.000017
1	H	2.626267	-2.181163	0.000023
1	H	4.151946	0.000000	0.000023
6	C	-3.504645	-0.000007	-0.000023
1	H	-4.072322	0.923826	0.000047
1	H	-4.072313	-0.923847	-0.000027

CBS-QB3 Free Energy = -423.454678
Thermal Correction to Gibbs Free Energy = 0.128658
CASPT2 Energy = -423.300182

Occupation		
A1	A2	Coefficient
	2 2 2 2 2 0 0 0 0 0 0	0.879420
	2 2 2 2 0 2 0 0 0 0 0	-0.092853
	2 2 2 2 0 0 2 0 0 0 0	-0.118827
	2 2 2 2 0 u 0 0 d 0 0	0.060214
	2 2 u 2 d u 0 0 0 d 0	-0.052551
	2 2 u 2 d 0 u 0 0 0 d	0.079283
	2 u 2 2 d u d 0 0 0 0	-0.100616
	2 u 2 2 d 0 u 0 d 0 0	0.058615
	u 2 2 2 d 0 u 0 0 d 0	0.052894
	u 2 2 2 d u 0 0 0 0 d	-0.056459
	2 2 2 0 2 2 0 0 0 0 0	-0.173856
	2 2 2 0 2 0 0 2 0 0 0	-0.062997
	2 2 2 0 2 u 0 0 d 0 0	-0.054537

	2 u 2 d 2 u 0 d 0 0 0	0.085780
	2 u 2 d 2 0 0 u d 0 0	0.059883
	2 2 2 u u d d 0 0 0 0	0.115754
	2 2 2 u u 0 d d 0 0 0	0.066425
	2 u 2 u 2 d 0 d 0 0 0	-0.053162

Compound 6 Triplet

Atomic Number	Atomic Type	Coordinates (Angstroms)		
		X	Y	Z
6	C	2.175037	-0.000052	0.000061
6	C	1.514087	-1.278196	0.000034
6	C	0.166882	-1.592512	-0.000006
6	C	1.514233	1.278188	0.000049
6	C	-0.924243	-0.719715	-0.000031
6	C	0.166944	1.592625	0.000012
6	C	-0.924281	0.719778	-0.000023
1	H	2.187625	-2.129117	0.000048
1	H	-0.070729	-2.651947	-0.000019
1	H	2.187792	2.12907	0.000073
6	C	-2.311191	-1.132362	-0.000071
1	H	-2.644847	-2.161437	-0.000085
6	C	-3.128549	-0.000029	-0.000087
6	C	-2.311228	1.132345	-0.000058
1	H	-4.208486	0.000016	-0.000116
1	H	-2.644939	2.161387	-0.000062
1	H	-0.070608	2.652034	0.00001
6	C	3.563984	-0.000052	0.000103
1	H	4.127068	-0.92466	0.000154
1	H	4.127084	0.924557	0.000087

CBS-QB3 Free Energy = -423.455460
Thermal Correction to Gibbs Free Energy = 0.126720
CASPT2 Energy = -423.292772

Occupation		
A1	A2	Coefficient
	2 2 2 2 u u 0 0 0 0 0	0.885032
	2 2 2 u d u 0 u 0 0 0	-0.054227
	2 2 2 u d u 0 0 u 0 0	0.063812
	2 2 u 2 d u u 0 0 0 0	-0.099883
	2 u 2 2 d u 0 0 u 0 0	-0.067807
	2 2 2 u u d 0 u 0 0 0	0.154182
	2 u 2 2 u d 0 0 u 0 0	-0.063133
	2 2 2 u u u 0 d 0 0 0	0.087731
	2 2 u 2 u u d 0 0 0 0	0.070670
	2 2 2 0 u u 0 2 0 0 0	-0.092839
	2 2 0 2 u u 2 0 0 0 0	-0.083208

Compound 6a Singlet

Atomic Number	Atomic Type	Coordinates (Angstroms)		
		X	Y	Z
6	C	2.147001	0.000000	-0.000005
6	C	1.507928	1.299611	0.000001
6	C	0.166609	1.626661	0.000002
6	C	1.507928	-1.299610	-0.000002
6	C	-0.939722	0.764285	-0.000002
6	C	0.166608	-1.626660	-0.000003
6	C	-0.939722	-0.764284	-0.000004
1	H	2.197755	2.138916	0.000005
1	H	-0.066259	2.688968	0.000006
1	H	2.197754	-2.138916	0.000000
1	H	-0.066259	-2.688967	-0.000005
6	C	-2.277357	1.160647	0.000007
1	H	-2.634986	2.182079	0.000015
6	C	-3.085933	0.000000	-0.000007
6	C	-2.277358	-1.160647	-0.000004
1	H	-2.634985	-2.182080	-0.000005
1	H	-4.170227	0.000000	-0.000006
6	C	3.522910	-0.000001	-0.000004
1	H	4.091931	0.925053	0.000059
1	H	4.091930	-0.925055	0.000055

Uncorrected Sum of Electronic and Thermal Free Energies = -424.153559
Corrected Sum of Electronic and Thermal Free Energies = -424.154926

Compound 6a Triplet

Atomic Number	Atomic Type	Coordinates (Angstroms)		
		X	Y	Z
6	C	-2.180431	0.000003	0.000062
6	C	-1.518065	1.280588	0.000037
6	C	-0.166690	1.595832	-0.000003
6	C	-1.518065	-1.280582	0.000047
6	C	0.927186	0.721131	-0.000030
6	C	-0.166691	-1.595835	0.000011
6	C	0.927191	-0.721137	-0.000023
1	H	-2.191635	2.133744	0.000054
1	H	0.070895	2.657068	-0.000015
1	H	-2.191630	-2.133741	0.000068
6	C	2.316534	1.134744	-0.000070
1	H	2.650568	2.165901	-0.000084
6	C	3.136158	0.000004	-0.000088
6	C	2.316534	-1.134744	-0.000060
1	H	4.218195	-0.000005	-0.000118
1	H	2.650580	-2.165896	-0.000064
1	H	0.070885	-2.657072	0.000008
6	C	-3.573796	-0.000003	0.000103
1	H	-4.138527	0.926031	0.000119
1	H	-4.138518	-0.926041	0.000120

Sum of Electronic and Thermal Free Energies = -423.155488

Compound 6b Singlet

Atomic Number	Atomic Type	Coordinates (Angstroms)		
		X	Y	Z
6	C	2.217728	-0.000007	0.000023
6	C	1.517288	1.283594	-0.000060
6	C	0.179612	1.576069	-0.000058
6	C	1.517286	-1.283594	0.000101
6	C	-0.965516	0.721996	-0.000004
6	C	0.179608	-1.576065	0.000099
6	C	-0.965523	-0.721991	0.000047
1	H	2.188462	2.143106	-0.000088
1	H	-0.057348	2.643126	-0.000030
6	C	-2.318378	1.143723	0.000191
6	C	-3.146298	-0.000001	-0.000196
6	C	-2.318389	-1.143722	-0.000079
1	H	-2.650719	-2.178335	-0.000165
1	H	-2.650697	2.178340	0.000336
1	H	-4.232347	0.000002	-0.000354
1	H	-0.057355	-2.643121	0.000095
6	C	3.594325	-0.000003	0.000021
1	H	4.160543	-0.927375	-0.000060
1	H	4.160542	0.927369	-0.000390
1	H	2.188448	-2.143118	0.000142

Uncorrected Sum of Electronic and Thermal Free Energies = -424.484664
Corrected Sum of Electronic and Thermal Free Energies = -424.484664

Compound 6b Triplet

Atomic Number	Atomic Type	Coordinates (Angstroms)		
		X	Y	Z
6	C	-2.168267	0.000019	-0.000012
6	C	-1.517688	1.267864	0.000068
6	C	-0.172391	1.609771	0.000094
6	C	-1.517678	-1.267868	-0.000081
6	C	0.967272	0.733428	0.000053
6	C	-0.172407	-1.609746	-0.000086
6	C	0.967265	-0.733411	-0.000047
1	H	-2.202497	2.118529	0.000099
1	H	0.056397	2.675602	0.000110
6	C	2.312523	1.146415	-0.000074
6	C	3.145491	-0.000015	-0.000107
6	C	2.312509	-1.146429	0.000201
1	H	2.645935	-2.180109	0.000359
1	H	2.645961	2.180091	-0.000144
1	H	4.231304	-0.000030	-0.000182
1	H	0.056393	-2.675577	-0.000073
6	C	-3.631135	-0.000015	-0.000019
1	H	-4.191955	-0.929759	-0.000108
1	H	-4.191999	0.929703	0.000111
1	H	-2.202495	-2.118525	-0.000105

Sum of Electronic and Thermal Free Energies = -424.423310

Compound 7-$N(CH_3)_2$ Singlet

Atomic Number	Atomic Type	Coordinates (Angstroms)		
		X	Y	Z
6	C	3.394992	-0.000094	0.000115
6	C	2.736991	-1.298425	0.022112
6	C	1.412974	-1.622620	0.023963
6	C	2.737204	1.298349	-0.021978
6	C	0.279373	-0.757874	0.005694
6	C	1.413248	1.622743	-0.024051
6	C	0.279490	0.758173	-0.005989
1	H	3.425745	-2.139124	0.037116
1	H	1.178025	-2.684217	0.038701
1	H	3.426097	2.138936	-0.036872
1	H	1.178465	2.684377	-0.038837
6	C	-1.023982	-1.175680	0.002699
1	H	-1.350612	-2.204556	0.028061
6	C	-1.878084	0.000317	-0.000316
6	C	-1.023771	1.176207	-0.003207
1	H	-1.350080	2.205185	-0.028414
6	C	4.761675	-0.000221	0.000214
1	H	5.330238	-0.924634	0.016175
1	H	5.330407	0.924090	-0.015665
7	N	-3.201837	0.000121	-0.000449
6	C	-4.000524	-1.232222	-0.056583
1	H	-4.408425	-1.455313	0.934788
1	H	-4.828927	-1.078440	-0.752346
1	H	-3.402471	-2.069887	-0.405619
6	C	-4.001559	1.231604	0.057273
1	H	-3.401353	2.072017	0.395650
1	H	-4.419423	1.448700	-0.931244
1	H	-4.822988	1.080479	0.761974

Uncorrected Sum of Electronic and Thermal Free Energies = -558.119688
Corrected Sum of Electronic and Thermal Free Energies = -558.119688

Compound 7-$N(CH_3)_2$ Triplet

Atomic Number	Atomic Type	Coordinates (Angstroms)		
		X	Y	Z
6	C	3.434201	-0.000018	0.008480
6	C	2.755937	-1.282350	0.004611
6	C	1.413726	-1.594566	-0.003878
6	C	2.755880	1.282358	0.004617
6	C	0.308054	-0.715296	-0.011904
6	C	1.413755	1.594593	-0.003933
6	C	0.307982	0.715307	-0.011950
1	H	3.428170	-2.136946	0.009304
1	H	1.173553	-2.654955	-0.004625
1	H	3.428148	2.136927	0.009363
1	H	1.173575	2.654981	-0.004696
6	C	-1.065788	-1.141994	-0.016750
1	H	-1.381751	-2.176933	-0.003287
6	C	-1.919693	0.000009	-0.030386
6	C	-1.065754	1.141956	-0.016771
1	H	-1.381706	2.176896	-0.003372
6	C	4.812747	-0.000008	0.016885
1	H	5.378730	-0.925135	0.020332
1	H	5.378723	0.925123	0.020365
7	N	-3.267609	-0.000004	-0.053530
6	C	-4.002295	-1.264467	0.033635
1	H	-3.933739	-1.699837	1.038382
1	H	-5.051428	-1.082423	-0.195725
1	H	-3.616554	-1.981683	-0.696267
6	C	-4.002243	1.264471	0.033718
1	H	-3.933599	1.699831	1.038460
1	H	-3.616518	1.981707	-0.696192
1	H	-5.051403	1.082499	-0.195577

Sum of Electronic and Thermal Free Energies = -558.092250

Compound 7-NH_2 Singlet

6	C	-2.595724	0.000005	-0.000086
6	C	-1.942422	1.298867	-0.000067
6	C	-0.616657	1.625503	0.000153
6	C	-1.942418	-1.298842	0.000123
6	C	0.514115	0.763398	0.000189
6	C	-0.616635	-1.625486	0.000185
6	C	0.514099	-0.763378	0.000049
1	H	-2.632114	2.138781	-0.000223
1	H	-0.383198	2.687409	0.000257
1	H	-2.632098	-2.138767	0.000165
1	H	-0.383191	-2.687393	0.000311
6	C	1.820873	1.177684	0.000224
1	H	2.178268	2.199134	0.000556
6	C	2.654347	-0.000061	-0.000122
6	C	1.820884	-1.177708	-0.000081
1	H	2.178152	-2.199202	0.000251
6	C	-3.964124	-0.000019	-0.000252
1	H	-4.532959	0.924547	-0.000420
1	H	-4.532929	-0.924602	-0.000228
7	N	3.978279	0.000025	-0.000313
1	H	4.506977	0.863279	-0.000493
1	H	4.507102	-0.863150	0.000115

Uncorrected Sum of Electronic and Thermal Free Energies = -479.542392
Corrected Sum of Electronic and Thermal Free Energies = -479.542392

Compound 7-NH_2 Triplet

Atomic Number	Atomic Type	Coordinates (Angstroms)		
		X	Y	Z
6	C	-2.639617	-0.000012	-0.000051
6	C	-1.964942	1.280864	0.000133
6	C	-0.619737	1.594311	0.00019
6	C	-1.964971	-1.280857	-0.000011
6	C	0.482792	0.717783	0.000218
6	C	-0.61972	-1.594296	0.000027
6	C	0.482763	-0.71778	0.000057
1	H	-2.636795	2.135656	0.00006
1	H	-0.380945	2.654986	0.000175
1	H	-2.636786	-2.135676	0.000056
1	H	-0.380955	-2.654977	0.000136
6	C	1.859125	1.141084	-0.000119
1	H	2.190859	2.173029	-0.000296
6	C	2.701571	0.000008	-0.000085
6	C	1.859135	-1.141113	0.000154
1	H	2.190935	-2.173038	0.000216
6	C	-4.022382	0.000003	-0.000227
1	H	-4.588046	0.925337	-0.000254
1	H	-4.588057	-0.925325	-0.000358
7	N	4.050349	0.000018	-0.00017
1	H	4.57672	0.860666	-0.000472
1	H	4.576537	-0.860754	0.000203

Sum of Electronic and Thermal Free Energies = -479.518975

Compound 7-OH Singlet

Atomic Number	Atomic Type	Coordinates (Angstroms)		
		X	Y	Z
6	C	2.578024	0.004744	0.000018
6	C	1.927015	1.301121	-0.000127
6	C	0.595629	1.626550	-0.000060
6	C	1.936319	-1.297249	0.000170
6	C	-0.524504	0.763131	0.000014
6	C	0.608525	-1.628993	0.000096
6	C	-0.520436	-0.773181	-0.000005
1	H	2.614434	2.142745	-0.000274
1	H	0.361509	2.688273	-0.000106
1	H	2.629830	-2.133718	0.000331
1	H	0.378565	-2.691623	0.000154
6	C	-1.839790	1.171700	0.000018
1	H	-2.203181	2.191321	0.000006
6	C	-2.645625	-0.010666	-0.000008
6	C	-1.829455	-1.187236	-0.000050
1	H	-2.206005	-2.200743	-0.000070
6	C	3.949355	0.013353	-0.000005
1	H	4.512952	0.941402	-0.000230
1	H	4.523875	-0.908030	0.000087
8	O	-3.954273	-0.088798	-0.000035
1	H	-4.388139	0.781121	0.000015

Uncorrected Sum of Electronic and Thermal Free Energies = -499.395235
Corrected Sum of Electronic and Thermal Free Energies = -499.395235

Compound 7-OH Triplet

Atomic Number	Atomic Type	Coordinates (Angstroms)		
		X	Y	Z
6	C	2.623530	0.003118	-0.000003
6	C	1.950427	1.282423	-0.000097
6	C	0.602530	1.594954	-0.000071
6	C	1.958222	-1.276456	0.000133
6	C	-0.496078	0.719163	-0.000022
6	C	0.607950	-1.593086	0.000105
6	C	-0.490348	-0.721415	0.000030
1	H	2.620972	2.138112	-0.000184
1	H	0.364317	2.655773	-0.000113
1	H	2.630524	-2.130759	0.000251
1	H	0.371973	-2.654391	0.000166
6	C	-1.877988	1.139189	-0.000041
1	H	-2.213894	2.169875	-0.000070
6	C	-2.694867	-0.003865	-0.000006
6	C	-1.869290	-1.148714	0.000034
1	H	-2.220997	-2.173239	0.000043
6	C	4.012089	0.004746	-0.000038
1	H	4.576306	0.931014	-0.000161
1	H	4.578083	-0.920405	0.000055
8	O	-4.025348	-0.097955	-0.000015
1	H	-4.461559	0.767329	-0.000015

Sum of Electronic and Thermal Free Energies = -499.380035

Compound 7-OCH_3 Singlet

Atomic Number	Atomic Type	Coordinates (Angstroms)		
		X	Y	Z
6	C	-3.009788	-0.138178	0.000006
6	C	-2.253820	-1.376718	0.000093
6	C	-0.900992	-1.593891	0.000058
6	C	-2.473487	1.211901	-0.000050
6	C	0.147409	-0.643114	0.000002
6	C	-1.178277	1.647449	0.000003
6	C	0.019427	0.884574	0.000077
1	H	-2.870713	-2.271458	0.000148
1	H	-0.582631	-2.633483	0.000067
1	H	-3.232107	1.989832	-0.000184
1	H	-1.033463	2.725008	-0.000089
6	C	1.490560	-0.952105	-0.000175
1	H	1.918421	-1.944183	-0.000319
6	C	2.206061	0.290589	0.000348
6	C	1.287475	1.400735	-0.000115
1	H	1.582285	2.440964	-0.000332
6	C	-4.374690	-0.261023	-0.000056
1	H	-4.858822	-1.232706	-0.000005
1	H	-5.023622	0.609421	-0.000161
8	O	3.490430	0.491597	0.000203
6	C	4.414171	-0.632605	-0.000187
1	H	4.263412	-1.229629	-0.901992
1	H	5.404590	-0.183424	-0.001210
1	H	4.264914	-1.228802	0.902425

Uncorrected Sum of Electronic and Thermal Free Energies = -538.680116
Corrected Sum of Electronic and Thermal Free Energies = -538.680116

Compound 7-OCH_3 Triplet

Atomic Number	Atomic Type	Coordinates (Angstroms)		
		X	Y	Z
6	C	3.055608	-0.135283	0.000086
6	C	2.281305	-1.358248	-0.000083
6	C	0.915341	-1.563174	-0.000164
6	C	2.494302	1.192228	0.000144
6	C	-0.114058	-0.603041	-0.000147
6	C	1.172626	1.614374	0.000059
6	C	0.009104	0.832273	-0.000064
1	H	2.883028	-2.263682	-0.000132
1	H	0.593797	-2.601784	-0.000251
1	H	3.231953	1.990808	0.000259
1	H	1.020989	2.691001	0.000088
6	C	-1.521923	-0.914855	-0.000248
1	H	-1.928489	-1.917751	-0.000329
6	C	-2.250035	0.289954	-0.000226
6	C	-1.330540	1.368284	-0.000133
1	H	-1.599750	2.417587	-0.000100
6	C	4.438928	-0.246063	0.000203
1	H	4.928526	-1.213802	0.000163
1	H	5.075728	0.631800	0.000338
8	O	-3.557940	0.498999	-0.000398
6	C	-4.439844	-0.644048	0.000649
1	H	-4.279577	-1.243646	0.901803
1	H	-5.447016	-0.232351	0.000949
1	H	-4.280551	-1.244574	-0.900064

Sum of Electronic and Thermal Free Energies = -538.663018

Compound 7-CH_3 Singlet

Atomic Number	Atomic Type	Coordinates (Angstroms)		
		X	Y	Z
6	C	2.604254	0.000000	0.003319
6	C	1.965437	-1.301018	0.001596
6	C	0.631313	-1.630557	-0.002294
6	C	1.965438	1.301018	0.001677
6	C	-0.484638	-0.769989	-0.005345
6	C	0.631313	1.630557	-0.002194
6	C	-0.484638	0.769990	-0.005297
1	H	2.658129	-2.138004	0.003613
1	H	0.399510	-2.692987	-0.002879
1	H	2.658129	2.138004	0.003745
1	H	0.399510	2.692987	-0.002713
6	C	-1.806930	-1.164342	-0.010411
1	H	-2.166821	-2.185231	-0.015000
6	C	-2.635603	0.000001	-0.013075
6	C	-1.806930	1.164343	-0.010339
1	H	-2.166820	2.185232	-0.014865
6	C	3.977988	0.000000	0.007448
1	H	4.547016	-0.924967	0.009115
1	H	4.547016	0.924967	0.009172
6	C	-4.116330	0.000000	0.011701
1	H	-4.532995	-0.895924	-0.455811
1	H	-4.453725	-0.000055	1.060625
1	H	-4.532996	0.895969	-0.455721

Uncorrected Sum of Electronic and Thermal Free Energies = -463.462677
Corrected Sum of Electronic and Thermal Free Energies = -463.462677

Compound 7-CH_3 Triplet

Atomic Number	Atomic Type	Coordinates (Angstroms)		
		X	Y	Z
6	C	-2.650353	0.000000	0.002910
6	C	-1.986032	1.280426	0.000723
6	C	-0.635518	1.595583	-0.002733
6	C	-1.986032	-1.280426	0.000723
6	C	0.459322	0.720734	-0.003872
6	C	-0.635518	-1.595583	-0.002733
6	C	0.459322	-0.720733	-0.003872
1	H	-2.659366	2.133799	0.001763
1	H	-0.397982	2.656772	-0.004228
1	H	-2.659365	-2.133799	0.001763
1	H	-0.397981	-2.656772	-0.004228
6	C	1.846283	1.130188	-0.007723
1	H	2.178923	2.162523	-0.014317
6	C	2.684923	0.000001	-0.006933
6	C	1.846283	-1.130188	-0.007723
1	H	2.178922	-2.162523	-0.014317
6	C	-4.042038	-0.000001	0.007118
1	H	-4.606741	0.925955	0.008834
1	H	-4.606741	-0.925956	0.008834
6	C	4.180867	0.000000	0.012617
1	H	4.585410	0.887609	-0.480340
1	H	4.550461	-0.000100	1.045734
1	H	4.585409	-0.887517	-0.480508

Sum of Electronic and Thermal Free Energies = -463.459439

Compound 7-F Singlet

Atomic Number	Atomic Type	Coordinates (Angstroms)		
		X	Y	Z
6	C	2.563456	0.000013	0.000041
6	C	1.923821	1.299554	0.000034
6	C	0.589860	1.629830	0.000016
6	C	1.923972	-1.299772	0.000022
6	C	-0.527981	0.771702	-0.000003
6	C	0.590300	-1.630076	0.000001
6	C	-0.528107	-0.772126	-0.000011
1	H	2.614905	2.137922	0.000047
1	H	0.358755	2.692210	0.000016
1	H	2.615307	-2.137921	0.000027
1	H	0.359149	-2.692461	-0.000008
6	C	-1.846769	1.182580	-0.000023
1	H	-2.229433	2.193623	-0.000027
6	C	-2.627502	0.000372	-0.000035
6	C	-1.846480	-1.182567	-0.000032
1	H	-2.230125	-2.193247	-0.000043
6	C	3.937897	0.000393	0.000062
1	H	4.506912	0.925515	0.000033
1	H	4.507395	-0.924442	0.000025
9	F	-3.935295	-0.000070	-0.000057

Uncorrected Sum of Electronic and Thermal Free Energies = -523.402044
Corrected Sum of Electronic and Thermal Free Energies = -523.402044

Compound 7-F Triplet

Atomic Number	Atomic Type	Coordinates (Angstroms)		
		X	Y	Z
6	C	2.610638	-0.000068	0.000042
6	C	1.945215	1.279253	0.000039
6	C	0.594100	1.594609	0.000019
6	C	1.945363	-1.279222	0.000023
6	C	-0.501705	0.721593	-0.000004
6	C	0.594085	-1.594569	-0.000001
6	C	-0.501660	-0.721594	-0.000013
1	H	2.616942	2.133885	0.000055
1	H	0.357309	2.655765	0.000022
1	H	2.616896	-2.134003	0.000028
6	C	-1.885760	1.149261	-0.000023
1	H	-2.240526	2.172499	-0.000022
6	C	-2.680077	0.000488	-0.000042
6	C	-1.885480	-1.149001	-0.000038
1	H	-2.242301	-2.171556	-0.000050
1	H	0.357265	-2.655718	-0.000011
6	C	4.002909	-0.000125	0.000064
1	H	4.567762	0.925796	0.000080
1	H	4.567623	-0.926126	0.000068
9	F	-4.002971	-0.000477	-0.000063

Sum of Electronic and Thermal Free Energies = -523.394129

Compound 7-CHO Singlet

Atomic Number	Atomic Type	Coordinates (Angstroms)		
		X	Y	Z
6	C	-2.932529	-0.108900	0.000016
6	C	-2.379231	1.228884	0.000007
6	C	-1.059943	1.644622	-0.000010
6	C	-2.211401	-1.363677	0.000006
6	C	0.096840	0.854133	-0.000023
6	C	-0.851361	-1.599902	-0.000011
6	C	0.198145	-0.665530	-0.000023
1	H	-3.121299	2.022490	0.000015
1	H	-0.898038	2.719924	-0.000014
1	H	-2.844433	-2.246438	0.000014
1	H	-0.547595	-2.644112	-0.000016
6	C	1.409724	1.342709	-0.000043
1	H	1.695918	2.387314	-0.000051
6	C	2.292590	0.240698	-0.000051
6	C	1.553347	-0.976640	-0.000043
1	H	1.993834	-1.965197	-0.000052
6	C	-4.307567	-0.190953	0.000034
1	H	-4.930979	0.698310	0.000032
1	H	-4.819886	-1.148619	0.000032
6	C	3.780298	0.316765	-0.000088
8	O	4.464330	-0.685764	0.000156
1	H	4.224357	1.329183	0.000170

Uncorrected Sum of Electronic and Thermal Free Energies = -537.465568
Corrected Sum of Electronic and Thermal Free Energies = -537.466819

Compound 7-CHO Triplet

Atomic Number	Atomic Type	Coordinates (Angstroms)		
		X	Y	Z
6	C	2.970689	-0.107905	0.000013
6	C	2.394919	1.214873	0.000000
6	C	1.068574	1.619971	-0.000016
6	C	2.227003	-1.342277	0.000009
6	C	-0.082321	0.818838	-0.000024
6	C	0.857686	-1.568650	-0.000004
6	C	-0.176767	-0.622407	-0.000019
1	H	3.123917	2.021175	0.000006
1	H	0.902669	2.694611	-0.000022
1	H	2.842849	-2.238014	0.000020
1	H	0.550090	-2.611577	-0.000004
6	C	-1.438108	1.323490	-0.000042
1	H	-1.704008	2.375101	-0.000053
6	C	-2.333172	0.244884	-0.000046
6	C	-1.582564	-0.948607	-0.000029
1	H	-2.004121	-1.947357	-0.000029
6	C	4.360880	-0.201170	0.000030
1	H	4.986299	0.685089	0.000034
1	H	4.862280	-1.162995	0.000042
6	C	-3.817560	0.308188	-0.000077
8	O	-4.498617	-0.696309	0.000139
1	H	-4.266594	1.319076	0.000128

Sum of Electronic and Thermal Free Energies = -537.468008

Compound 7-CF_3 Singlet

Atomic Number	Atomic Type	Coordinates (Angstroms)		
		X	Y	Z
6	C	-3.639452	-0.000001	0.007271
6	C	-3.002688	-1.299420	0.002206
6	C	-1.660016	-1.627033	-0.008497
6	C	-3.002689	1.299418	0.002206
6	C	-0.556454	-0.763008	-0.015657
6	C	-1.660017	1.627034	-0.008497
6	C	-0.556455	0.763009	-0.015657
1	H	-3.692279	-2.138797	0.007255
1	H	-1.426951	-2.689166	-0.010932
1	H	-3.692280	2.138795	0.007255
1	H	-1.426953	2.689167	-0.010932
6	C	0.780481	-1.163358	-0.026470
1	H	1.144530	-2.182119	-0.034808
6	C	1.582250	0.000003	-0.032049
6	C	0.780481	1.163361	-0.026470
1	H	1.144528	2.182123	-0.034809
6	C	-5.017083	0.000000	0.019392
1	H	-5.586090	-0.925176	0.024428
1	H	-5.586089	0.925176	0.024428
6	C	3.092411	0.000001	0.001719
9	F	3.518348	-0.000073	1.282858
9	F	3.589544	-1.092213	-0.606428
9	F	3.589548	1.092281	-0.606304

Uncorrected Sum of Electronic and Thermal Free Energies = -761.202540
Corrected Sum of Electronic and Thermal Free Energies = -761.204249

Compound 7-CF_3 Triplet

Atomic Number	Atomic Type	Coordinates (Angstroms)		
		X	Y	Z
6	C	-3.688381	0.000000	0.006545
6	C	-3.027429	-1.280891	0.001929
6	C	-1.675923	-1.597045	-0.006428
6	C	-3.027430	1.280891	0.001929
6	C	-0.583011	-0.720699	-0.010641
6	C	-1.675924	1.597046	-0.006428
6	C	-0.583011	0.720700	-0.010641
1	H	-3.700940	-2.134013	0.005155
1	H	-1.438273	-2.658139	-0.009172
1	H	-3.700942	2.134012	0.005155
1	H	-1.438274	2.658139	-0.009172
6	C	0.803181	-1.138578	-0.019353
1	H	1.145862	-2.166667	-0.028204
6	C	1.617454	0.000002	-0.023528
6	C	0.803180	1.138580	-0.019353
1	H	1.145861	2.166670	-0.028204
6	C	-5.082781	-0.000001	0.015933
1	H	-5.647388	-0.926142	0.019762
1	H	-5.647389	0.926139	0.019762
6	C	3.123458	0.000000	0.002320
9	F	3.579575	-0.000061	1.273596
9	F	3.613611	-1.093963	-0.612893
9	F	3.613612	1.094021	-0.612790

Sum of Electronic and Thermal Free Energies = -761.203789

Compound 7-CN Singlet

Atomic Number	Atomic Type	Coordinates (Angstroms)		
		X	Y	Z
6	C	-2.859035	0.000000	0.000103
6	C	-2.223801	1.300010	0.000003
6	C	-0.884582	1.628879	-0.000128
6	C	-2.223801	-1.300010	0.000003
6	C	0.224705	0.766145	-0.000122
6	C	-0.884581	-1.628879	-0.000128
6	C	0.224705	-0.766144	-0.000122
1	H	-2.914595	2.138415	0.000025
1	H	-0.652211	2.691148	-0.000229
1	H	-2.914594	-2.138416	0.000025
1	H	-0.652210	-2.691148	-0.000229
6	C	1.551348	1.170242	-0.000135
1	H	1.919342	2.187304	-0.000070
6	C	2.362326	0.000001	-0.000142
6	C	1.551349	-1.170240	-0.000135
1	H	1.919343	-2.187303	-0.000070
6	C	-4.236739	-0.000001	0.000264
1	H	-4.805917	0.925186	0.000347
1	H	-4.805917	-0.925188	0.000347
6	C	3.779797	0.000001	-0.000039
7	N	4.945230	-0.000002	0.000474

Uncorrected Sum of Electronic and Thermal Free Energies = -516.388890
Corrected Sum of Electronic and Thermal Free Energies = -516.389525

Compound 7-CN Triplet

Atomic Number	Atomic Type	Coordinates (Angstroms)		
		X	Y	Z
6	C	-2.904689	0.000000	0.000052
6	C	-2.242481	1.281334	-0.000034
6	C	-0.891965	1.597668	-0.000137
6	C	-2.242481	-1.281334	-0.000034
6	C	0.202501	0.720697	-0.000157
6	C	-0.891965	-1.597668	-0.000137
6	C	0.202501	-0.720697	-0.000157
1	H	-2.915882	2.134610	0.000007
1	H	-0.654160	2.658656	-0.000167
1	H	-2.915882	-2.134610	0.000007
1	H	-0.654160	-2.658656	-0.000167
6	C	1.583728	1.141330	0.000144
1	H	1.927822	2.168889	0.000115
6	C	2.408692	0.000000	-0.000116
6	C	1.583728	-1.141330	0.000144
1	H	1.927822	-2.168889	0.000115
6	C	-4.297495	0.000000	0.000207
1	H	-4.862401	0.926018	0.000278
1	H	-4.862401	-0.926018	0.000278
6	C	3.831246	0.000000	-0.000134
7	N	4.994476	0.000000	0.000239

Sum of Electronic and Thermal Free Energies = -516.388170

Compound 7-NO_2 Singlet

Atomic Number	Atomic Type	Coordinates (Angstroms)		
		X	Y	Z
6	C	3.262489	0.000000	0.000057
6	C	2.626334	1.299075	0.000120
6	C	1.282039	1.626919	0.000114
6	C	2.626334	-1.299075	-0.000029
6	C	0.179365	0.762934	0.000046
6	C	1.282039	-1.626919	-0.000074
6	C	0.179365	-0.762934	-0.000045
1	H	3.315178	2.139079	0.000183
1	H	1.048695	2.688976	0.000178
1	H	3.315177	-2.139079	-0.000067
1	H	1.048695	-2.688976	-0.000144
6	C	-1.157979	1.170493	0.000066
1	H	-1.539772	2.182067	0.000120
6	C	-1.938268	0.000000	-0.000044
6	C	-1.157979	-1.170493	-0.000086
1	H	-1.539772	-2.182067	-0.000146
6	C	4.641369	0.000000	0.000084
1	H	5.210500	0.925228	0.000152
1	H	5.210500	-0.925228	0.000043
7	N	-3.402718	0.000000	-0.000077
8	O	-3.950051	-1.099192	0.000256
8	O	-3.950051	1.099192	-0.000387

Uncorrected Sum of Electronic and Thermal Free Energies = -628.642654
Corrected Sum of Electronic and Thermal Free Energies = -628.644094

Compound 7-NO_2 Triplet

Atomic Number	Atomic Type	Coordinates (Angstroms)		
		X	Y	Z
6	C	-3.305048	0.000000	0.000060
6	C	-2.644633	-1.281252	0.000112
6	C	-1.293140	-1.598337	0.000103
6	C	-2.644633	1.281252	-0.000014
6	C	-0.199036	-0.722416	0.000041
6	C	-1.293140	1.598337	-0.000054
6	C	-0.199035	0.722416	-0.000030
1	H	-3.318068	-2.134472	0.000167
1	H	-1.055592	-2.659379	0.000153
1	H	-3.318068	2.134472	-0.000045
1	H	-1.055592	2.659379	-0.000111
6	C	1.183723	-1.147975	0.000039
1	H	1.546648	-2.168425	0.000075
6	C	1.978307	0.000000	-0.000039
6	C	1.183723	1.147975	-0.000073
1	H	1.546648	2.168425	-0.000125
6	C	-4.699683	0.000000	0.000084
1	H	-5.264429	-0.926166	0.000141
1	H	-5.264429	0.926166	0.000048
7	N	3.435119	0.000000	-0.000077
8	O	3.983289	1.099024	0.000021
8	O	3.983289	-1.099024	-0.000162

Sum of Electronic and Thermal Free Energies = -628.644349

Compound 8-$N(CH_3)_2$ Singlet

Atomic Number	Atomic Type	Coordinates (Angstroms)		
		X	Y	Z
6	C	-1.663255	-1.654045	-0.030382
6	C	-2.599158	-0.567608	0.214337
6	C	-2.344180	0.779219	0.302544
6	C	-0.239578	-1.583059	-0.113165
6	C	-1.113971	1.434310	0.123223
6	C	0.663718	-0.488890	-0.038929
6	C	0.247418	0.890448	-0.086113
1	H	-3.635036	-0.874772	0.324048
1	H	-3.202625	1.425574	0.467082
1	H	0.221640	-2.559428	-0.178783
6	C	-1.031803	2.864971	-0.005612
1	H	-1.872511	3.537492	0.104017
6	C	0.255575	3.196338	-0.350243
6	C	1.045364	1.990084	-0.400583
1	H	2.087887	1.968826	-0.682259
1	H	0.630635	4.188115	-0.569007
6	C	-2.233445	-2.912775	-0.135197
1	H	-3.306235	-3.055137	-0.059263
1	H	-1.630673	-3.799448	-0.303869
7	N	1.994483	-0.792451	0.038140
6	C	2.527582	-2.103509	-0.354092
1	H	3.581656	-1.974621	-0.606657
1	H	2.458408	-2.835537	0.460651
1	H	2.018195	-2.483809	-1.240271
6	C	2.980166	0.039126	0.743346
1	H	2.486768	0.803849	1.339468
1	H	3.549772	-0.610843	1.415025
1	H	3.684148	0.509244	0.048025

Uncorrected Sum of Electronic and Thermal Free Energies = -558.070733
Corrected Sum of Electronic and Thermal Free Energies = -558.069447

Compound 8-$N(CH_3)_2$ Triplet

Atomic Number	Atomic Type	Coordinates (Angstroms)		
		X	Y	Z
6	C	2.359293	-0.158077	-0.043617
6	C	2.140498	-1.554011	0.252601
6	C	0.950677	-2.246307	0.342942
6	C	1.356387	0.850589	-0.144539
6	C	-0.346475	-1.756962	0.130718
6	C	-0.069414	0.808924	-0.050589
6	C	-0.820777	-0.414378	-0.098168
1	H	3.046164	-2.136337	0.397615
1	H	1.029302	-3.312840	0.538877
1	H	1.776160	1.842617	-0.250059
6	C	-1.492558	-2.650059	-0.003453
1	H	-1.441182	-3.723960	0.127660
6	C	-2.594267	-1.911141	-0.368273
6	C	-2.202663	-0.545168	-0.434134
1	H	-2.854999	0.257618	-0.748240
1	H	-3.583915	-2.288145	-0.588770
6	C	3.689737	0.258841	-0.177769
1	H	4.509865	-0.443856	-0.083086
1	H	3.942486	1.292648	-0.385899
7	N	-0.713030	2.007707	0.052841
6	C	-0.077998	3.285481	-0.303411
1	H	-0.870380	4.004269	-0.517983
1	H	0.532593	3.681922	0.517134
1	H	0.528350	3.185906	-1.204465
6	C	-2.001355	2.178012	0.739186
1	H	-2.253301	1.287358	1.312921
1	H	-1.906621	3.021298	1.429988
1	H	-2.809830	2.403106	0.035463

Sum of Electronic and Thermal Free Energies = -558.074169

Compound 8-NH_2 Singlet

Atomic Number	Atomic Type	Coordinates (Angstroms)		
		X	Y	Z
6	C	2.163937	-0.279189	0.000179
6	C	1.530555	-1.587640	-0.000441
6	C	0.190376	-1.901197	-0.000859
6	C	1.531101	0.995781	0.000142
6	C	-0.902963	-1.020889	-0.000490
6	C	0.164043	1.363926	-0.000224
6	C	-0.942926	0.458273	0.000103
1	H	2.221199	-2.425782	-0.000478
1	H	-0.057617	-2.959549	-0.001219
1	H	2.223391	1.833788	0.000400
6	C	-2.264253	-1.483046	0.000074
1	H	-2.557480	-2.524675	-0.000059
6	C	-3.096739	-0.388906	0.000789
6	C	-2.291233	0.807745	0.000514
1	H	-2.711682	1.805850	0.001099
1	H	-4.179319	-0.403026	0.001365
6	C	3.552180	-0.298019	0.000871
1	H	4.104644	-1.231679	0.000994
1	H	4.133715	0.618542	0.001451
7	N	-0.088326	2.693572	-0.000734
1	H	-1.025462	3.062750	-0.002010
1	H	0.662424	3.367734	-0.000357

Uncorrected Sum of Electronic and Thermal Free Energies = -479.513012
Corrected Sum of Electronic and Thermal Free Energies = -479.514073

Compound 8-NH_2 Triplet

Atomic Number	Atomic Type	Coordinates (Angstroms)		
		X	Y	Z
6	C	-2.187064	-0.283091	-0.000359
6	C	-1.543970	-1.575706	0.000934
6	C	-0.199084	-1.883625	0.001403
6	C	-1.530234	0.978072	-0.000365
6	C	0.893241	-1.001606	0.000566
6	C	-0.152563	1.344957	0.000396
6	C	0.938662	0.431916	-0.000192
1	H	-2.225841	-2.421497	0.001412
1	H	0.049223	-2.942035	0.002193
1	H	-2.210347	1.826993	-0.000928
6	C	2.275282	-1.470375	-0.000099
1	H	2.560518	-2.515511	0.000247
6	C	3.125323	-0.379691	-0.001137
6	C	2.331639	0.788796	-0.001142
1	H	2.738404	1.793427	-0.002289
1	H	4.206632	-0.404128	-0.001939
6	C	-3.589641	-0.271808	-0.001556
1	H	-4.157462	-1.195293	-0.001555
1	H	-4.148895	0.657218	-0.002625
7	N	0.093170	2.676310	0.001468
1	H	1.027265	3.053186	0.003110
1	H	-0.661241	3.346434	0.001402

Sum of Electronic and Thermal Free Energies = -479.515605

Compound 8-OH Singlet

Atomic Number	Atomic Type	Coordinates (Angstroms)		
		X	Y	Z
6	C	-2.160784	-0.263707	0.000050
6	C	-1.529742	-1.569833	-0.000143
6	C	-0.189224	-1.902455	-0.000107
6	C	-1.521293	1.018321	0.000102
6	C	0.908728	-1.033658	0.000014
6	C	-0.165337	1.348893	-0.000006
6	C	0.935328	0.457116	0.000034
1	H	-2.225104	-2.404322	-0.000326
1	H	0.043878	-2.964186	-0.000243
1	H	-2.207132	1.862604	0.000151
6	C	2.268548	-1.470128	-0.000085
1	H	2.584965	-2.504987	-0.000212
6	C	3.086013	-0.353575	0.000168
6	C	2.273369	0.834983	0.000182
1	H	2.641021	1.851892	0.000267
1	H	4.169273	-0.359368	0.000244
6	C	-3.545363	-0.283133	0.000180
1	H	-4.098389	-1.216655	0.000051
1	H	-4.127775	0.633122	0.000374
8	O	0.201779	2.644684	-0.000331
1	H	-0.556424	3.247493	0.000009

Uncorrected Sum of Electronic and Thermal Free Energies = -499.379191
Corrected Sum of Electronic and Thermal Free Energies = -499.380644

Compound 8-OH Triplet

Atomic Number	Atomic Type	Coordinates (Angstroms)		
		X	Y	Z
6	C	2.187913	-0.261501	0.000109
6	C	1.551378	-1.555831	-0.000110
6	C	0.208088	-1.882569	-0.000379
6	C	1.518887	1.002492	-0.000446
6	C	-0.892524	-1.012175	-0.000152
6	C	0.152374	1.324913	-0.000293
6	C	-0.929016	0.420857	0.000053
1	H	2.240296	-2.395926	-0.000129
1	H	-0.025248	-2.944431	-0.000760
1	H	2.187999	1.861333	-0.000817
6	C	-2.277891	-1.457083	-0.000233
1	H	-2.583022	-2.496950	-0.000543
6	C	-3.118794	-0.346388	0.000399
6	C	-2.321622	0.809668	0.000665
1	H	-2.670982	1.833979	0.001049
1	H	-4.200463	-0.366059	0.000645
6	C	3.587297	-0.242400	0.000687
1	H	4.160655	-1.162572	0.001088
1	H	4.141875	0.689574	0.000799
8	O	-0.222237	2.619006	-0.000435
1	H	0.530246	3.229109	0.000345

Sum of Electronic and Thermal Free Energies = -499.382081

Compound 8-OCH_3 Singlet

Atomic Number	Atomic Type	Coordinates (Angstroms)		
		X	Y	Z
6	C	-1.764766	-1.270785	0.000026
6	C	-0.735439	-2.292123	-0.000159
6	C	0.638330	-2.156261	-0.000187
6	C	-1.598703	0.153351	0.000129
6	C	1.378990	-0.968572	-0.000120
6	C	-0.434459	0.926781	0.000041
6	C	0.905961	0.444167	0.000011
1	H	-1.111876	-3.311050	-0.000244
1	H	1.214914	-3.077752	-0.000185
1	H	-2.532365	0.701973	0.000205
6	C	2.808946	-0.928876	0.000243
1	H	3.450149	-1.800548	0.000437
6	C	3.207027	0.393871	0.000088
6	C	2.041745	1.243642	-0.000108
1	H	2.050430	2.324530	-0.000170
1	H	4.229831	0.750671	0.000195
6	C	-3.062266	-1.755706	0.000120
1	H	-3.268949	-2.820829	0.000033
1	H	-3.919064	-1.088978	0.000331
8	O	-0.490078	2.265381	-0.000185
6	C	-1.737082	2.984567	0.000026
1	H	-2.314611	2.759636	0.901574
1	H	-1.452625	4.035323	-0.000028
1	H	-2.314923	2.759638	-0.901324

Uncorrected Sum of Electronic and Thermal Free Energies = -538.659076
Corrected Sum of Electronic and Thermal Free Energies = -538.660584

Compound 8-OCH_3 Triplet

Atomic Number	Atomic Type	Coordinates (Angstroms)		
		X	Y	Z
6	C	1.864464	-1.173047	-0.000093
6	C	0.894658	-2.239216	0.000441
6	C	-0.485338	-2.175703	0.000369
6	C	1.582990	0.230771	-0.000714
6	C	-1.295528	-1.030127	0.000042
6	C	0.363924	0.930987	-0.000512
6	C	-0.930137	0.354291	-0.000163
1	H	1.321311	-3.238363	0.000837
1	H	-1.008306	-3.128897	0.000631
1	H	2.471433	0.851394	-0.001511
6	C	-2.751934	-1.072722	0.000156
1	H	-3.333408	-1.987218	0.000346
6	C	-3.249566	0.227269	0.000033
6	C	-2.160330	1.114420	-0.000136
1	H	-2.211051	2.195008	-0.000233
1	H	-4.293483	0.511149	0.000094
6	C	3.213071	-1.548253	-0.000181
1	H	3.505287	-2.592358	0.000226
1	H	4.007061	-0.809604	-0.000661
8	O	0.332705	2.271620	-0.001197
6	C	1.530603	3.073014	0.001387
1	H	2.121848	2.888588	-0.900232
1	H	1.176055	4.102183	0.003056
1	H	2.120347	2.885060	0.903252

Sum of Electronic and Thermal Free Energies = -538.662010

Compound 8-CH_3 Singlet

Atomic Number	Atomic Type	Coordinates (Angstroms)		
		X	Y	Z
6	C	2.160492	-0.267992	0.000022
6	C	1.573019	-1.587912	0.000011
6	C	0.241627	-1.944649	-0.000016
6	C	1.485863	1.012958	0.000004
6	C	-0.877547	-1.101541	-0.000030
6	C	0.141386	1.355106	0.000018
6	C	-0.933955	0.431213	0.000026
1	H	2.287902	-2.405801	0.000037
1	H	0.027280	-3.010686	0.000026
1	H	2.172679	1.854563	-0.000028
6	C	-2.200415	-1.554626	0.000112
1	H	-2.510167	-2.591391	0.000219
6	C	-3.050880	-0.434529	0.000004
6	C	-2.285567	0.761709	-0.000132
1	H	-2.701008	1.759287	-0.000230
1	H	-4.134424	-0.474096	0.000022
6	C	3.536478	-0.225629	0.000060
1	H	4.132698	-1.133269	0.000087
1	H	4.078175	0.715612	0.000083
6	C	-0.201720	2.830615	-0.000073
1	H	-0.791879	3.094498	-0.883699
1	H	-0.791395	3.094681	0.883826
1	H	0.697453	3.448266	-0.000381

Uncorrected Sum of Electronic and Thermal Free Energies = -463.450077
Corrected Sum of Electronic and Thermal Free Energies = -463.451338

Compound 8-CH_3 Triplet

Atomic Number	Atomic Type	Coordinates (Angstroms)		
		X	Y	Z
6	C	2.196061	-0.267131	0.000063
6	C	1.583364	-1.568311	-0.000002
6	C	0.241801	-1.908687	-0.000047
6	C	1.498870	0.992302	-0.000068
6	C	-0.865441	-1.050958	-0.000010
6	C	0.141465	1.325474	-0.000135
6	C	-0.918983	0.390393	-0.000032
1	H	2.280749	-2.401829	-0.000012
1	H	0.019409	-2.973091	-0.000106
1	H	2.168871	1.848179	-0.000103
6	C	-2.237877	-1.524162	-0.000007
1	H	-2.522989	-2.569846	-0.000055
6	C	-3.103253	-0.428222	0.000087
6	C	-2.329738	0.738050	0.000126
1	H	-2.720686	1.747170	0.000193
1	H	-4.184443	-0.469693	0.000144
6	C	3.591176	-0.220236	0.000220
1	H	4.184924	-1.127791	0.000300
1	H	4.125620	0.723461	0.000259
6	C	-0.206015	2.800071	-0.000197
1	H	-0.795889	3.065637	-0.883913
1	H	-0.795165	3.065867	0.883943
1	H	0.691026	3.420429	-0.000639

Sum of Electronic and Thermal Free Energies = -463.452067

Compound 8-F Singlet

Atomic Number	Atomic Type	Coordinates (Angstroms)		
		X	Y	Z
6	C	-2.164521	-0.248514	0.000025
6	C	-1.540003	-1.555376	0.000288
6	C	-0.200161	-1.899260	0.000370
6	C	-1.515556	1.037319	-0.000211
6	C	0.911900	-1.048701	0.000217
6	C	-0.167241	1.323602	-0.000232
6	C	0.943767	0.465895	-0.000085
1	H	-2.239465	-2.386438	0.000437
1	H	0.020828	-2.963733	0.000559
1	H	-2.164481	1.907896	-0.000388
6	C	2.255014	-1.471898	0.000243
1	H	2.586107	-2.502082	0.000404
6	C	3.075757	-0.338052	0.000062
6	C	2.277702	0.847444	-0.000139
1	H	2.649212	1.862820	-0.000331
1	H	4.159651	-0.349532	0.000048
6	C	-3.543253	-0.244500	0.000004
1	H	-4.113113	-1.168653	0.000197
1	H	-4.109355	0.682288	-0.000171
9	F	0.134466	2.634409	-0.000445

Uncorrected Sum of Electronic and Thermal Free Energies = -523.397265
Corrected Sum of Electronic and Thermal Free Energies = -523.398802

Compound 8-F Triplet

Atomic Number	Atomic Type	Coordinates (Angstroms)		
		X	Y	Z
6	C	-2.197977	-0.244726	0.000021
6	C	-1.556239	-1.536399	0.000005
6	C	-0.210811	-1.870400	-0.000030
6	C	-1.521724	1.019743	0.000011
6	C	0.897444	-1.011223	-0.000029
6	C	-0.161189	1.294756	-0.000023
6	C	0.933235	0.425187	0.000011
1	H	-2.243616	-2.378053	0.000019
1	H	0.013215	-2.934302	-0.000062
1	H	-2.151302	1.905586	0.000021
6	C	2.281672	-1.448925	-0.000032
1	H	2.593803	-2.486791	-0.000078
6	C	3.120645	-0.330736	0.000035
6	C	2.325565	0.822669	0.000070
1	H	2.672788	1.848042	0.000127
1	H	4.202492	-0.349243	0.000048
6	C	-3.594395	-0.220928	0.000030
1	H	-4.171779	-1.138882	0.000030
1	H	-4.143333	0.714360	0.000030
9	F	0.147820	2.602797	-0.000061

Sum of Electronic and Thermal Free Energies = -523.399510

Compound 8-CHO Singlet

Atomic Number	Atomic Type	Coordinates (Angstroms)		
		X	Y	Z
6	C	-2.308366	0.046640	-0.000004
6	C	-2.035222	-1.371967	0.000017
6	C	-0.821476	-2.030894	0.000029
6	C	-1.343238	1.125729	-0.000007
6	C	0.465779	-1.480760	0.000015
6	C	0.896521	-0.005005	-0.000013
1	H	-2.918507	-2.004694	0.000025
1	H	-0.866568	-3.117260	0.000047
1	H	-1.802954	2.112039	-0.000004
6	C	1.639905	-2.244276	0.000026
1	H	1.687028	-3.325289	0.000046
6	C	2.734541	-1.364726	-0.000007
6	C	2.286576	-0.011096	-0.000014
1	H	2.919649	0.862351	-0.000028
1	H	3.776470	-1.665442	-0.000003
6	C	-3.635030	0.409812	-0.000008
1	H	-4.427420	-0.332768	0.000071
1	H	-3.941046	1.451884	0.000059
6	C	0.038273	1.129690	-0.000017
6	C	0.625638	2.526980	-0.000029
8	O	1.805632	2.788190	-0.000018
1	H	-0.135106	3.332898	0.000012

Uncorrected Sum of Electronic and Thermal Free Energies = -537.461778
Corrected Sum of Electronic and Thermal Free Energies = -537.463306

Compound 8-CHO Triplet

Atomic Number	Atomic Type	Coordinates (Angstroms)		
		X	Y	Z
6	C	-2.342988	-0.011811	0.000023
6	C	-2.005400	-1.411026	0.000019
6	C	-0.760532	-2.017269	0.000010
6	C	-1.391144	1.070564	0.000018
6	C	0.504833	-1.414480	0.000002
6	C	0.871725	-0.013063	0.000001
1	H	-2.857176	-2.085999	0.000024
1	H	-0.763972	-3.104636	0.000008
1	H	-1.861879	2.052476	0.000024
6	C	1.739437	-2.168375	-0.000012
1	H	1.794588	-3.250906	-0.000018
6	C	2.822544	-1.281643	-0.000008
6	C	2.327240	0.025977	0.000000
1	H	2.910335	0.935068	0.000001
1	H	3.869019	-1.557325	-0.000011
6	C	-3.691229	0.329319	0.000031
1	H	-4.465004	-0.430906	0.000035
1	H	-4.014165	1.364906	0.000033
6	C	-0.001822	1.105493	0.000005
6	C	0.555027	2.515529	0.000002
8	O	1.730329	2.799487	-0.000077
1	H	-0.220536	3.306142	-0.000037

Sum of Electronic and Thermal Free Energies = -537.464240

Compound 8-CF_3 Singlet

Atomic Number	Atomic Type	Coordinates (Angstroms)		
		X	Y	Z
6	C	1.619664	-1.844811	-0.000563
6	C	2.703294	-0.891605	-0.000039
6	C	2.653443	0.488523	0.001020
6	C	0.196745	-1.575418	-0.000517
6	C	1.524516	1.315289	0.000505
6	C	-0.497231	-0.384839	-0.000068
6	C	0.036957	0.928378	0.000012
1	H	3.696613	-1.331465	-0.000430
1	H	3.610822	1.003846	0.001820
1	H	-0.415429	-2.470150	-0.000808
6	C	1.576163	2.712710	0.000026
1	H	2.479511	3.308288	0.000184
6	C	0.258645	3.202529	-0.000642
6	C	-0.669985	2.125873	-0.000611
1	H	-1.743710	2.239674	-0.000572
1	H	-0.017769	4.251044	-0.001859
6	C	1.981283	-3.172933	-0.000476
1	H	3.023772	-3.477520	0.000005
1	H	1.239012	-3.965798	-0.000085
6	C	-2.029465	-0.494066	0.000234
9	F	-2.551567	0.106825	-1.090509
9	F	-2.452823	-1.770788	0.000155
9	F	-2.550834	0.106663	1.091295

Uncorrected Sum of Electronic and Thermal Free Energies = -761.195218
Corrected Sum of Electronic and Thermal Free Energies = -761.196698

Compound 8-CF_3 Triplet

Atomic Number	Atomic Type	Coordinates (Angstroms)		
		X	Y	Z
6	C	1.931735	-1.570749	0.000048
6	C	2.804062	-0.426971	-0.000293
6	C	2.491022	0.922295	-0.000026
6	C	0.489951	-1.527079	0.000430
6	C	1.224672	1.521536	0.000108
6	C	-0.398513	-0.463912	0.000108
6	C	-0.088267	0.915398	-0.000016
1	H	3.864894	-0.662892	-0.000683
1	H	3.336645	1.605798	-0.000018
1	H	0.025427	-2.507387	0.000840
6	C	1.021983	2.954290	0.000168
1	H	1.822643	3.684888	0.000297
6	C	-0.351089	3.227888	0.000045
6	C	-1.042924	2.015156	-0.000088
1	H	-2.118501	1.906378	-0.000243
1	H	-0.803551	4.210797	0.000053
6	C	2.521648	-2.831341	0.000011
1	H	3.600067	-2.948218	-0.000456
1	H	1.924049	-3.736564	0.000426
6	C	-1.890319	-0.826782	-0.000017
9	F	-2.500905	-0.313257	-1.091142
9	F	-2.101781	-2.152225	-0.000203
9	F	-2.501251	-0.313538	1.091003

Sum of Electronic and Thermal Free Energies = -761.196628

Compound 8-CN Singlet

Atomic Number	Atomic Type	Coordinates (Angstroms)		
		X	Y	Z
6	C	-2.231419	-0.221517	0.000054
6	C	-1.771458	-1.592568	0.000211
6	C	-0.482925	-2.093002	0.000262
6	C	-1.434660	0.983585	-0.000179
6	C	0.721750	-1.380959	0.000177
6	C	-0.057123	1.143025	-0.000283
6	C	0.938455	0.131560	-0.000137
1	H	-2.565725	-2.333753	0.000293
1	H	-0.389432	-3.176205	0.000272
1	H	-2.009006	1.904870	-0.000221
6	C	1.995892	-1.965343	-0.000110
1	H	2.199961	-3.028012	-0.000235
6	C	2.952860	-0.937192	-0.000023
6	C	2.310026	0.335637	0.000228
1	H	2.810952	1.294784	0.000357
1	H	4.027148	-1.083962	-0.000043
6	C	-3.597599	-0.043624	0.000090
1	H	-4.280453	-0.888132	-0.000041
1	H	-4.043075	0.946984	-0.000339
6	C	0.419029	2.503492	-0.000177
7	N	0.810380	3.597837	-0.000103

Uncorrected Sum of Electronic and Thermal Free Energies = -516.382821
Corrected Sum of Electronic and Thermal Free Energies = -516.384340

Compound 8-CN Triplet

Atomic Number	Atomic Type	Coordinates (Angstroms)		
		X	Y	Z
6	C	-2.265580	-0.237601	-0.000017
6	C	-1.768933	-1.591028	0.000119
6	C	-0.466433	-2.064467	0.000167
6	C	-1.457554	0.952451	-0.000061
6	C	0.725017	-1.326753	0.000085
6	C	-0.070705	1.114501	-0.000067
6	C	0.916980	0.102003	-0.000029
1	H	-2.542463	-2.354397	0.000184
1	H	-0.355352	-3.146073	0.000261
1	H	-2.022390	1.880554	-0.000093
6	C	2.048346	-1.917008	0.000078
1	H	2.242642	-2.983410	0.000137
6	C	3.010486	-0.898450	-0.000007
6	C	2.348886	0.333056	-0.000032
1	H	2.813301	1.312093	-0.000091
1	H	4.083477	-1.038353	-0.000040
6	C	-3.647272	-0.058222	-0.000098
1	H	-4.325094	-0.905062	-0.000076
1	H	-4.089492	0.932201	-0.000202
6	C	0.400714	2.476348	-0.000075
7	N	0.793095	3.570497	-0.000065

Sum of Electronic and Thermal Free Energies = -516.384219

Compound 8-NO_2 Singlet

Atomic Number	Atomic Type	Coordinates (Angstroms)		
		X	Y	Z
6	C	2.294301	-0.351190	0.056929
6	C	1.925836	-1.747482	0.054273
6	C	0.670474	-2.322757	0.005271
6	C	1.411072	0.791116	0.020332
6	C	-0.578864	-1.692250	-0.035844
6	C	0.037217	0.844908	0.016177
6	C	-0.915311	-0.194602	-0.005383
1	H	2.763480	-2.438393	0.087457
1	H	0.642933	-3.409613	-0.002547
1	H	1.905675	1.756484	-0.011247
6	C	-1.800700	-2.372801	-0.092572
1	H	-1.919044	-3.447997	-0.121828
6	C	-2.835498	-1.422808	-0.105665
6	C	-2.302414	-0.104601	-0.047161
1	H	-2.886901	0.802540	-0.024435
1	H	-3.894250	-1.652508	-0.150384
6	C	3.642696	-0.073427	0.086658
1	H	4.384560	-0.866238	0.113246
1	H	4.014284	0.947232	0.082671
7	N	-0.492192	2.255289	0.011695
8	O	0.107827	3.060787	-0.688931
8	O	-1.465109	2.488817	0.717319

Uncorrected Sum of Electronic and Thermal Free Energies = -628.637928
Corrected Sum of Electronic and Thermal Free Energies = -628.639426

Compound 8-NO_2 Triplet

Atomic Number	Atomic Type	Coordinates (Angstroms)		
		X	Y	Z
6	C	2.317268	-0.432286	0.047528
6	C	1.866655	-1.799731	0.055477
6	C	0.578563	-2.309117	0.018713
6	C	1.460663	0.722991	0.004818
6	C	-0.638176	-1.614138	-0.026222
6	C	0.081885	0.819661	0.004817
6	C	-0.896011	-0.190555	-0.017577
1	H	2.662077	-2.539481	0.087219
1	H	0.499453	-3.393509	0.021568
1	H	1.975969	1.678376	-0.030044
6	C	-1.928698	-2.264768	-0.077520
1	H	-2.071086	-3.339235	-0.088333
6	C	-2.936773	-1.293259	-0.113244
6	C	-2.341056	-0.030404	-0.078937
1	H	-2.860156	0.917336	-0.092697
1	H	-4.000993	-1.483398	-0.160786
6	C	3.689113	-0.197717	0.076028
1	H	4.399419	-1.017015	0.106459
1	H	4.091200	0.809674	0.067541
7	N	-0.408890	2.241673	0.022097
8	O	0.202271	3.043527	-0.670649
8	O	-1.371552	2.482908	0.742036

Sum of Electronic and Thermal Free Energies = -628.639606

Compound 9-$N(CH_3)_2$ Singlet

Atomic Number	Atomic Type	Coordinates (Angstroms)		
		X	Y	Z
6	C	-1.056534	1.291564	0.350782
6	C	-0.026350	2.144782	-0.247955
6	C	1.255977	1.838132	-0.557917
6	C	-1.078023	-0.185544	0.166257
6	C	1.954017	0.611869	-0.295292
6	C	0.086112	-0.996207	0.474434
6	C	1.385805	-0.643490	0.283093
1	H	-0.342257	3.175414	-0.385050
1	H	1.863206	2.636117	-0.978132
1	H	-0.097976	-2.011294	0.808988
6	C	3.294514	0.392477	-0.445422
1	H	4.009865	1.101722	-0.844391
6	C	3.633545	-0.953969	0.038797
6	C	2.506961	-1.568134	0.480492
1	H	2.413965	-2.567783	0.884035
1	H	4.632566	-1.372248	0.033337
6	C	-2.000131	1.829506	1.160523
1	H	-1.987984	2.891005	1.387554
1	H	-2.761312	1.233194	1.651769
7	N	-2.189957	-0.794937	-0.217447
6	C	-2.289579	-2.273610	-0.260557
1	H	-3.294668	-2.538977	-0.581641
1	H	-1.566480	-2.683898	-0.969716
1	H	-2.121001	-2.699419	0.730980
6	C	-3.390532	-0.109418	-0.743406
1	H	-3.188380	0.941035	-0.928056
1	H	-3.668367	-0.602130	-1.677764
1	H	-4.216161	-0.205923	-0.032760

Uncorrected Sum of Electronic and Thermal Free Energies = -558.090802
Corrected Sum of Electronic and Thermal Free Energies = -558.090802

Compound 9-$N(CH_3)_2$ Triplet

Atomic Number	Atomic Type	Coordinates (Angstroms)		
		X	Y	Z
6	C	1.028300	1.298938	-0.258161
6	C	-0.083305	2.123798	0.155672
6	C	-1.418696	1.815390	0.372557
6	C	1.084267	-0.179200	-0.088506
6	C	-2.034749	0.580347	0.173655
6	C	-0.047398	-1.014790	-0.214804
6	C	-1.401325	-0.682882	-0.154157
1	H	0.162807	3.181141	0.216575
1	H	-2.063013	2.646647	0.647923
1	H	0.144102	-2.077814	-0.299841
6	C	-3.463251	0.332557	0.252763
1	H	-4.204175	1.087121	0.487359
6	C	-3.704091	-1.003919	-0.025365
6	C	-2.462202	-1.642606	-0.264739
1	H	-2.323737	-2.693969	-0.489151
1	H	-4.672533	-1.486056	-0.050205
6	C	2.060384	1.939773	-0.899671
1	H	2.037913	3.015137	-1.040946
1	H	2.897070	1.406765	-1.333238
7	N	2.303569	-0.753950	0.102650
6	C	2.515227	-2.197547	-0.072638
1	H	3.588704	-2.374226	-0.147104
1	H	2.127617	-2.769352	0.779360
1	H	2.050619	-2.548331	-0.995562
6	C	3.425109	-0.087312	0.788122
1	H	3.128313	0.882643	1.178108
1	H	3.732339	-0.722758	1.624689
1	H	4.279378	0.035423	0.115112

Sum of Electronic and Thermal Free Energies = -558.080462

Compound 9-NH_2 Singlet

Atomic Number	Atomic Type	Coordinates (Angstroms)		
		X	Y	Z
6	C	1.874132	0.571867	0.085667
6	C	1.022595	1.730917	-0.174395
6	C	-0.327010	1.817809	-0.266344
6	C	1.451140	-0.832942	-0.110458
6	C	-1.303885	0.783582	-0.102993
6	C	0.131767	-1.361482	0.087796
6	C	-1.048402	-0.678807	0.112328
1	H	1.579094	2.661447	-0.236865
1	H	-0.735649	2.810779	-0.436801
1	H	0.073607	-2.446650	0.154262
6	C	-2.660011	0.938296	-0.099771
1	H	-3.192370	1.871206	-0.239347
6	C	-3.302286	-0.366604	0.125861
6	C	-2.347589	-1.326641	0.249559
1	H	-2.493660	-2.385906	0.413119
1	H	-4.373466	-0.518915	0.179102
6	C	3.121967	0.774497	0.591749
1	H	3.480457	1.782312	0.773511
1	H	3.776172	-0.030969	0.907503
7	N	2.386908	-1.705991	-0.464223
1	H	2.181931	-2.695445	-0.542823
1	H	3.321025	-1.408880	-0.716091

Uncorrected Sum of Electronic and Thermal Free Energies = -479.522593
Corrected Sum of Electronic and Thermal Free Energies = -479.522593

Compound 9-NH_2 Triplet

Atomic Number	Atomic Type	Coordinates (Angstroms)		
		X	Y	Z
6	C	1.885920	0.583506	0.041729
6	C	0.984804	1.696058	-0.093460
6	C	-0.403084	1.783482	-0.144034
6	C	1.479704	-0.834599	-0.052334
6	C	-1.326123	0.746931	-0.049754
6	C	0.165521	-1.342074	0.019830
6	C	-1.051421	-0.674337	0.049662
1	H	1.496343	2.654355	-0.122613
1	H	-0.807157	2.788801	-0.233434
1	H	0.096629	-2.428465	0.030173
6	C	-2.767339	0.899712	-0.035711
1	H	-3.285260	1.849004	-0.099573
6	C	-3.353919	-0.356690	0.073485
6	C	-2.331145	-1.328063	0.121428
1	H	-2.474796	-2.399566	0.197140
1	H	-4.415930	-0.561021	0.111897
6	C	3.211166	0.902264	0.277935
1	H	3.525232	1.939077	0.313536
1	H	3.980000	0.171839	0.497736
7	N	2.465066	-1.749947	-0.186156
1	H	2.248045	-2.734177	-0.255162
1	H	3.416930	-1.487358	-0.389267

Sum of Electronic and Thermal Free Energies = -479.519629

Compound 9-OH Singlet

Atomic Number	Atomic Type	Coordinates (Angstroms)		
		X	Y	Z
6	C	-1.885765	0.565994	0.000158
6	C	-1.008609	1.718047	-0.000109
6	C	0.360279	1.821397	-0.000293
6	C	-1.453007	-0.836897	0.000039
6	C	1.322032	0.790199	-0.000198
6	C	-0.145677	-1.355929	0.000119
6	C	1.062074	-0.684084	0.000047
1	H	-1.547451	2.660741	-0.000135
1	H	0.759579	2.832681	-0.000493
1	H	-0.078821	-2.443368	0.000193
6	C	2.702740	0.944731	-0.000279
1	H	3.233553	1.888357	-0.000461
6	C	3.318522	-0.359598	-0.000095
6	C	2.340133	-1.335211	0.000107
1	H	2.493034	-2.406452	0.000266
1	H	4.387231	-0.537537	-0.000106
6	C	-3.240260	0.802908	0.000585
1	H	-3.614391	1.821313	0.000707
1	H	-3.970114	0.003490	0.000851
8	O	-2.465639	-1.702603	-0.000148
1	H	-2.172289	-2.627745	-0.000117

Uncorrected Sum of Electronic and Thermal Free Energies = -499.384845
Corrected Sum of Electronic and Thermal Free Energies = -499.387290

Compound 9-OH Triplet

Atomic Number	Atomic Type	Coordinates (Angstroms)		
		X	Y	Z
6	C	-1.896863	0.565951	0.000166
6	C	-1.008560	1.694109	-0.000049
6	C	0.378834	1.791182	-0.000222
6	C	-1.461979	-0.832794	0.000084
6	C	1.310357	0.753907	-0.000177
6	C	-0.162667	-1.347405	0.000089
6	C	1.054988	-0.669320	-0.000008
1	H	-1.528846	2.648090	-0.000062
1	H	0.779478	2.801795	-0.000381
1	H	-0.092793	-2.434505	0.000151
6	C	2.749322	0.918858	-0.000279
1	H	3.258332	1.875370	-0.000419
6	C	3.354089	-0.340661	-0.000169
6	C	2.346515	-1.317625	-0.000008
1	H	2.497849	-2.390722	0.000101
1	H	4.419003	-0.532753	-0.000206
6	C	-3.258394	0.829385	0.000436
1	H	-3.614171	1.853668	0.000491
1	H	-3.996779	0.039158	0.000611
8	O	-2.493124	-1.689563	0.000063
1	H	-2.210937	-2.617126	0.000035

Sum of Electronic and Thermal Free Energies = -499.385026

Compound 9-OCH_3 Singlet

Atomic Number	Atomic Type	Coordinates (Angstroms)		
		X	Y	Z
6	C	-1.403713	1.252301	-0.000525
6	C	-0.260338	2.140872	0.001064
6	C	1.089853	1.897379	0.001806
6	C	-1.351345	-0.223267	0.000202
6	C	1.758171	0.655861	0.000762
6	C	-0.208465	-1.050554	-0.000811
6	C	1.128070	-0.698701	-0.000808
1	H	-0.542257	3.189504	0.001464
1	H	1.731291	2.775322	0.003031
1	H	-0.398785	-2.117583	-0.001480
6	C	3.132548	0.451986	0.000840
1	H	3.886750	1.228832	0.001867
6	C	3.394695	-0.967486	-0.000762
6	C	2.200097	-1.657624	-0.001769
1	H	2.071459	-2.732097	-0.002975
1	H	4.382390	-1.412590	-0.001123
6	C	-2.646237	1.840379	-0.003115
1	H	-2.734984	2.921494	-0.003734
1	H	-3.561948	1.264392	-0.004827
8	O	-2.555241	-0.770979	0.001501
6	C	-2.793437	-2.199392	0.001596
1	H	-3.877356	-2.294324	0.002372
1	H	-2.377642	-2.652739	-0.901372
1	H	-2.376386	-2.652897	0.903900

Uncorrected Sum of Electronic and Thermal Free Energies = -538.666422
Corrected Sum of Electronic and Thermal Free Energies = -538.669342

Compound 9-OCH_3 Triplet

Atomic Number	Atomic Type	Coordinates (Angstroms)		
		X	Y	Z
6	C	1.420546	1.240545	0.000198
6	C	0.281853	2.114085	-0.000379
6	C	-1.087438	1.870544	-0.000590
6	C	1.354208	-0.231338	-0.000413
6	C	-1.734476	0.636870	-0.000136
6	C	0.214125	-1.043515	-0.000197
6	C	-1.132505	-0.677649	0.000148
1	H	0.552155	3.166550	-0.000469
1	H	-1.722948	2.752637	-0.000988
1	H	0.394087	-2.112649	-0.000474
6	C	-3.169711	0.440801	0.000006
1	H	-3.899837	1.241338	-0.000156
6	C	-3.442967	-0.928658	0.000382
6	C	-2.223415	-1.624766	0.000429
1	H	-2.103985	-2.701886	0.000646
1	H	-4.426887	-1.379023	0.000591
6	C	2.668209	1.843902	0.001252
1	H	2.748364	2.925199	0.001523
1	H	3.584566	1.270443	0.001883
8	O	2.570957	-0.774447	-0.001330
6	C	2.796885	-2.199440	0.000306
1	H	3.879815	-2.308068	0.000783
1	H	2.377198	-2.652626	0.902552
1	H	2.377922	-2.654617	-0.901285

Sum of Electronic and Thermal Free Energies = -538.665847

Compound 9-CH_3 Singlet

Atomic Number	Atomic Type	Coordinates (Angstroms)		
		X	Y	Z
6	C	1.819528	0.636802	-0.000174
6	C	0.897924	1.762706	-0.000259
6	C	-0.479217	1.826965	-0.000207
6	C	1.490824	-0.796062	-0.000082
6	C	-1.387797	0.761254	-0.000030
6	C	0.204101	-1.331917	0.000067
6	C	-1.050667	-0.713745	0.000116
1	H	1.398433	2.726636	-0.000352
1	H	-0.911606	2.824631	-0.000317
1	H	0.157934	-2.418116	0.000241
6	C	-2.780264	0.856771	0.000021
1	H	-3.351810	1.775829	-0.000068
6	C	-3.317700	-0.456854	0.000239
6	C	-2.276720	-1.402940	0.000306
1	H	-2.394139	-2.478781	0.000462
1	H	-4.375339	-0.694501	0.000350
6	C	3.143663	1.005013	-0.000175
1	H	3.428805	2.052218	0.000095
1	H	3.957001	0.290587	0.000229
6	C	2.624168	-1.796288	0.000044
1	H	3.261906	-1.685912	-0.884040
1	H	3.261640	-1.686186	0.884345
1	H	2.240125	-2.816642	-0.000141

Uncorrected Sum of Electronic and Thermal Free Energies = -463.447850
Corrected Sum of Electronic and Thermal Free Energies = -463.449158

Compound 9-CH_3 Triplet

Atomic Number	Atomic Type	Coordinates (Angstroms)		
		X	Y	Z
6	C	1.850165	0.623183	-0.000060
6	C	0.930358	1.734415	-0.000044
6	C	-0.456240	1.801354	0.000008
6	C	1.486691	-0.793909	0.000135
6	C	-1.359046	0.737435	0.000023
6	C	0.187361	-1.312660	0.000121
6	C	-1.054349	-0.671368	0.000045
1	H	1.423587	2.702846	-0.000118
1	H	-0.882376	2.801680	0.000005
1	H	0.130148	-2.398437	0.000185
6	C	-2.801664	0.855022	0.000015
1	H	-3.343316	1.793665	0.000000
6	C	-3.365386	-0.426592	0.000014
6	C	-2.326844	-1.365708	0.000037
1	H	-2.439151	-2.443684	0.000050
1	H	-4.423514	-0.653102	-0.000001
6	C	3.196545	0.981154	-0.000401
1	H	3.487172	2.025904	-0.000627
1	H	4.000410	0.258233	-0.000571
6	C	2.603258	-1.807981	0.000190
1	H	3.243782	-1.693188	-0.882025
1	H	3.243589	-1.693326	0.882549
1	H	2.214583	-2.826658	0.000062

Sum of Electronic and Thermal Free Energies = -463.451241

Compound 9-F Singlet

Atomic Number	Atomic Type	Coordinates (Angstroms)		
		X	Y	Z
6	C	-1.880022	0.550578	0.000127
6	C	-1.017366	1.714547	0.000217
6	C	0.356538	1.815105	0.000184
6	C	-1.422944	-0.831039	-0.000032
6	C	1.312903	0.784577	0.000044
6	C	-0.156228	-1.385017	-0.000135
6	C	1.064148	-0.711491	-0.000106
1	H	-1.559276	2.655570	0.000328
1	H	0.757846	2.825644	0.000279
1	H	-0.133842	-2.471758	-0.000251
6	C	2.695249	0.951113	0.000016
1	H	3.218071	1.898914	0.000099
6	C	3.309204	-0.336333	-0.000144
6	C	2.328263	-1.335617	-0.000219
1	H	2.502448	-2.403625	-0.000341
1	H	4.378817	-0.512106	-0.000200
6	C	-3.236043	0.765416	0.000176
1	H	-3.630885	1.776350	0.000201
1	H	-3.949989	-0.049698	0.000030
9	F	-2.411711	-1.734481	-0.000101

Uncorrected Sum of Electronic and Thermal Free Energies = -523.398503
Corrected Sum of Electronic and Thermal Free Energies = -523.399962

Compound 9-F Triplet

Atomic Number	Atomic Type	Coordinates (Angstroms)		
		X	Y	Z
6	C	-1.907814	0.542853	0.000104
6	C	-1.035325	1.686362	0.000206
6	C	0.350781	1.786177	0.000182
6	C	-1.429701	-0.823299	-0.000047
6	C	1.292954	0.753325	0.000045
6	C	-0.152969	-1.362085	-0.000137
6	C	1.060323	-0.672904	-0.000100
1	H	-1.563823	2.635893	0.000321
1	H	0.747916	2.798244	0.000281
1	H	-0.121425	-2.448676	-0.000248
6	C	2.729622	0.936599	0.000022
1	H	3.226327	1.899743	0.000110
6	C	3.353889	-0.316060	-0.000128
6	C	2.362421	-1.305680	-0.000203
1	H	2.525813	-2.376976	-0.000320
1	H	4.421765	-0.491332	-0.000177
6	C	-3.279503	0.763517	0.000156
1	H	-3.669232	1.775337	0.000270
1	H	-3.991154	-0.051810	0.000087
9	F	-2.404916	-1.741472	-0.000103

Sum of Electronic and Thermal Free Energies = -523.401426

Compound 9-CHO Singlet

Atomic Number	Atomic Type	Coordinates (Angstroms)		
		X	Y	Z
6	C	1.473379	0.874929	0.075077
6	C	0.477240	1.890144	-0.110354
6	C	-0.915329	1.810390	-0.177450
6	C	1.279802	-0.571485	0.055243
6	C	-1.705437	0.675530	-0.071669
6	C	0.079425	-1.254077	0.106334
6	C	-1.242205	-0.769191	0.091615
1	H	0.873944	2.901394	-0.144068
1	H	-1.438749	2.755111	-0.302579
1	H	0.159192	-2.340298	0.134504
6	C	-3.117656	0.652678	-0.104600
1	H	-3.757083	1.518796	-0.211808
6	C	-3.529164	-0.668332	0.028712
6	C	-2.383941	-1.537451	0.137323
1	H	-2.424980	-2.615218	0.231444
1	H	-4.559226	-1.006064	0.045014
6	C	2.714435	1.394631	0.413004
1	H	2.896702	2.463456	0.339559
1	H	3.518851	0.784924	0.801683
6	C	2.480676	-1.440870	-0.101784
8	O	3.596657	-1.034948	-0.370236
1	H	2.290743	-2.523883	0.019451

Uncorrected Sum of Electronic and Thermal Free Energies = -537.462643
Corrected Sum of Electronic and Thermal Free Energies = -537.462724

Compound 9-CHO Triplet

Atomic Number	Atomic Type	Coordinates (Angstroms)		
		X	Y	Z
6	C	1.513898	0.932590	0.000017
6	C	0.460547	1.922931	-0.000002
6	C	-0.922827	1.827591	-0.000016
6	C	1.296389	-0.513260	0.000015
6	C	-1.701458	0.665241	-0.000012
6	C	0.067741	-1.182724	0.000014
6	C	-1.241691	-0.697920	0.000004
1	H	0.835004	2.943196	-0.000005
1	H	-1.461800	2.771891	-0.000032
1	H	0.142802	-2.269204	0.000020
6	C	-3.143945	0.617407	-0.000026
1	H	-3.789897	1.487962	-0.000041
6	C	-3.563084	-0.725488	-0.000012
6	C	-2.431069	-1.538063	0.000004
1	H	-2.418960	-2.621867	0.000015
1	H	-4.590051	-1.066434	-0.000016
6	C	2.802188	1.465536	0.000038
1	H	2.932036	2.542836	0.000039
1	H	3.685575	0.844374	0.000056
6	C	2.448768	-1.493705	0.000010
8	O	3.628692	-1.220212	-0.000029
1	H	2.123014	-2.551875	0.000008

Sum of Electronic and Thermal Free Energies = -537.461817

Compound 9-CF_3 Singlet

Atomic Number	Atomic Type	Coordinates (Angstroms)		
		X	Y	Z
6	C	0.773666	1.386734	-0.000665
6	C	-0.434876	2.184170	-0.000577
6	C	-1.781082	1.845509	0.000578
6	C	0.853597	-0.072495	-0.000335
6	C	-2.333296	0.568710	0.000742
6	C	-0.192605	-0.970526	-0.000917
6	C	-1.583799	-0.749880	-0.000882
1	H	-0.244107	3.253459	-0.001256
1	H	-2.480814	2.677542	0.001020
1	H	0.086905	-2.019107	-0.001169
6	C	-3.710688	0.262968	0.001058
1	H	-4.515876	0.985807	0.001868
6	C	-3.843245	-1.125718	-0.000071
6	C	-2.547100	-1.744473	-0.000847
1	H	-2.361766	-2.811063	-0.001761
1	H	-4.783342	-1.665656	0.000065
6	C	1.918991	2.160946	-0.000769
1	H	1.844170	3.243676	-0.001277
1	H	2.920114	1.751040	-0.000046
6	C	2.254036	-0.691398	0.000319
9	F	2.958912	-0.303116	-1.091005
9	F	2.957697	-0.302421	1.092209
9	F	2.227073	-2.032572	0.000657

Uncorrected Sum of Electronic and Thermal Free Energies = -761.192447
Corrected Sum of Electronic and Thermal Free Energies = -761.192357

Compound 9-CF_3 Triplet

Atomic Number	Atomic Type	Coordinates (Angstroms)		
		X	Y	Z
6	C	0.822318	1.406268	-0.000042
6	C	-0.386015	2.198854	-0.000142
6	C	-1.730484	1.860508	0.000072
6	C	0.852449	-0.051458	-0.000072
6	C	-2.290467	0.579484	0.000081
6	C	-0.220595	-0.936873	-0.000109
6	C	-1.596647	-0.680341	-0.000099
1	H	-0.195370	3.268434	-0.000293
1	H	-2.427359	2.695094	0.000151
1	H	0.044877	-1.988765	-0.000118
6	C	-3.703977	0.276887	0.000103
1	H	-4.493547	1.019597	0.000236
6	C	-3.877158	-1.116950	0.000044
6	C	-2.616567	-1.716255	-0.000102
1	H	-2.411992	-2.780669	-0.000232
1	H	-4.826639	-1.635967	0.000060
6	C	2.000343	2.153028	0.000001
1	H	1.948552	3.236167	-0.000062
1	H	2.988092	1.716820	0.000106
6	C	2.237214	-0.720925	0.000013
9	F	2.943630	-0.359973	-1.093420
9	F	2.943569	-0.359849	1.093485
9	F	2.160680	-2.062853	0.000120

Sum of Electronic and Thermal Free Energies = -761.192963

Compound 9-CN Singlet

Atomic Number	Atomic Type	Coordinates (Angstroms)		
		X	Y	Z
6	C	-1.527642	0.993305	0.000100
6	C	-0.463028	1.972678	0.000197
6	C	0.915304	1.832225	0.000175
6	C	-1.376732	-0.464559	-0.000036
6	C	1.656400	0.649246	0.000052
6	C	-0.200579	-1.211674	-0.000117
6	C	1.127734	-0.773992	-0.000084
1	H	-0.818160	2.999256	0.000305
1	H	1.487693	2.756548	0.000265
1	H	-0.333911	-2.290594	-0.000224
6	C	3.059043	0.560262	0.000037
1	H	3.742765	1.399241	0.000117
6	C	3.415072	-0.797345	-0.000101
6	C	2.244648	-1.608456	-0.000173
1	H	2.227039	-2.690892	-0.000279
1	H	4.430509	-1.176847	-0.000145
6	C	-2.798650	1.523276	0.000162
1	H	-2.953112	2.597655	0.000350
1	H	-3.686738	0.898708	0.000187
6	C	-2.582033	-1.239000	-0.000110
7	N	-3.559044	-1.872698	-0.000170

Uncorrected Sum of Electronic and Thermal Free Energies = -516.384068
Corrected Sum of Electronic and Thermal Free Energies = -516.385674

Compound 9-CN Triplet

Atomic Number	Atomic Type	Coordinates (Angstroms)		
		X	Y	Z
6	C	-1.572340	0.990187	0.000117
6	C	-0.509829	1.963897	0.000209
6	C	0.871211	1.827348	0.000181
6	C	-1.371765	-0.457280	-0.000029
6	C	1.620089	0.645773	0.000048
6	C	-0.172674	-1.186205	-0.000118
6	C	1.138564	-0.715748	-0.000087
1	H	-0.864142	2.991133	0.000320
1	H	1.436614	2.756030	0.000274
1	H	-0.289817	-2.266935	-0.000226
6	C	3.061826	0.573045	0.000024
1	H	3.721447	1.433246	0.000106
6	C	3.458983	-0.773619	-0.000122
6	C	2.312557	-1.570737	-0.000191
1	H	2.284319	-2.654281	-0.000302
1	H	4.480179	-1.131632	-0.000171
6	C	-2.869438	1.491312	0.000175
1	H	-3.045728	2.561226	0.000285
1	H	-3.739603	0.845203	0.000115
6	C	-2.549844	-1.271307	-0.000096
7	N	-3.498186	-1.947712	-0.000152

Sum of Electronic and Thermal Free Energies = -516.385460

Compound 9-NO_2 Singlet

Atomic Number	Atomic Type	Coordinates (Angstroms)		
		X	Y	Z
6	C	-1.161377	1.225258	-0.095412
6	C	-0.024101	2.107098	-0.000058
6	C	1.343274	1.858954	0.073014
6	C	-1.108705	-0.225882	-0.030675
6	C	1.986833	0.626727	0.046890
6	C	-0.022392	-1.068260	-0.050260
6	C	1.345716	-0.746833	-0.034833
1	H	-0.290204	3.160090	-0.022219
1	H	1.980841	2.737295	0.135247
1	H	-0.261801	-2.128081	-0.061974
6	C	3.385737	0.426711	0.084864
1	H	4.130582	1.209406	0.143927
6	C	3.627512	-0.943317	0.029321
6	C	2.382519	-1.661917	-0.036878
1	H	2.281393	-2.739130	-0.076998
1	H	4.606488	-1.408874	0.036921
6	C	-2.344478	1.888269	-0.369390
1	H	-2.362574	2.973652	-0.394302
1	H	-3.274621	1.380862	-0.590416
7	N	-2.412245	-0.944969	0.092615
8	O	-2.500847	-2.046938	-0.434392
8	O	-3.297606	-0.389473	0.744645

Uncorrected Sum of Electronic and Thermal Free Energies = -628.636705
Corrected Sum of Electronic and Thermal Free Energies = -628.638338

Compound 9-NO_2 Triplet

Atomic Number	Atomic Type	Coordinates (Angstroms)		
		X	Y	Z
6	C	-1.209327	1.236879	-0.064276
6	C	-0.065346	2.114960	-0.067894
6	C	1.298347	1.859301	-0.026908
6	C	-1.095013	-0.204179	-0.005107
6	C	1.946958	0.620023	0.009110
6	C	0.005614	-1.048352	-0.000584
6	C	1.356245	-0.696389	0.013552
1	H	-0.329841	3.168041	-0.107647
1	H	1.939999	2.737037	-0.030424
1	H	-0.219072	-2.112061	-0.003160
6	C	3.379218	0.427983	0.036245
1	H	4.107994	1.230368	0.039958
6	C	3.661778	-0.946680	0.055150
6	C	2.452603	-1.646249	0.041493
1	H	2.333953	-2.723596	0.050052
1	H	4.649186	-1.388929	0.076200
6	C	-2.459122	1.845536	-0.143893
1	H	-2.530399	2.925739	-0.203838
1	H	-3.389769	1.293096	-0.146572
7	N	-2.380404	-0.955818	0.040200
8	O	-2.628431	-1.665003	-0.926448
8	O	-3.062937	-0.811993	1.046786

Sum of Electronic and Thermal Free Energies = -628.637916

Compound 10-$N(CH_3)_2$ Singlet

Atomic Number	Atomic Type	Coordinates (Angstroms)		
		X	Y	Z
6	C	-0.873885	0.381882	-0.209236
6	C	-0.098161	1.558148	-0.048229
6	C	1.280993	1.705036	0.117134
6	C	-0.415930	-0.950822	-0.369952
6	C	2.268799	0.727490	0.106160
6	C	0.888221	-1.440524	-0.352072
6	C	2.084924	-0.761500	-0.126299
1	H	-0.658067	2.489539	-0.017078
1	H	1.627302	2.725776	0.264621
1	H	-1.169977	-1.694930	-0.594964
1	H	0.986363	-2.508869	-0.534356
6	C	3.644592	0.938976	0.267856
1	H	4.117394	1.896163	0.445646
6	C	4.298902	-0.300241	0.154095
6	C	3.359626	-1.328182	-0.088994
1	H	3.587670	-2.377650	-0.224125
1	H	5.369584	-0.447085	0.237067
6	C	-2.292562	0.680968	-0.258656
1	H	-2.552899	1.680517	-0.599727
7	N	-3.330936	-0.046801	0.068232
6	C	-4.696336	0.465874	-0.165313
1	H	-5.243136	-0.250537	-0.784152
1	H	-5.209501	0.576436	0.793533
1	H	-4.655370	1.426758	-0.677153
6	C	-3.285096	-1.364785	0.727841
1	H	-3.392035	-2.164652	-0.010560
1	H	-2.350961	-1.480558	1.274124
1	H	-4.124344	-1.417224	1.423501

Uncorrected Sum of Electronic and Thermal Free Energies = -558.122263
Corrected Sum of Electronic and Thermal Free Energies = -558.122263

Compound 10-$N(CH_3)_2$ Triplet

Atomic Number	Atomic Type	Coordinates (Angstroms)		
		X	Y	Z
6	C	-0.912724	0.419890	-0.134050
6	C	-0.070563	1.583881	-0.029908
6	C	1.301261	1.708855	0.063641
6	C	-0.419114	-0.919662	-0.330178
6	C	2.273891	0.685098	0.068923
6	C	0.872125	-1.410280	-0.319096
6	C	2.080598	-0.714140	-0.102381
1	H	-0.609437	2.528335	0.003786
1	H	1.677229	2.724656	0.156637
1	H	-1.169584	-1.664442	-0.563718
1	H	0.969462	-2.476935	-0.507646
6	C	3.703189	0.894969	0.216572
1	H	4.170145	1.862832	0.359337
6	C	4.363647	-0.339543	0.141773
6	C	3.399842	-1.330090	-0.057962
1	H	3.584973	-2.392791	-0.164911
1	H	5.430951	-0.495333	0.221201
6	C	-2.300641	0.719390	-0.119614
1	H	-2.561033	1.762356	-0.277308
7	N	-3.371444	-0.060724	0.068417
6	C	-4.715739	0.481829	-0.173433
1	H	-5.228906	-0.128644	-0.923325
1	H	-5.297278	0.466081	0.753595
1	H	-4.648227	1.505043	-0.542666
6	C	-3.348815	-1.434014	0.581723
1	H	-3.364446	-2.168523	-0.231182
1	H	-2.472445	-1.591670	1.210179
1	H	-4.243039	-1.583002	1.191056

Sum of Electronic and Thermal Free Energies = -558.096785

Compound 10-NH_2 Singlet

Atomic Number	Atomic Type	Coordinates (Angstroms)		
		X	Y	Z
6	C	-1.681854	0.225804	0.000098
6	C	-0.955858	1.445350	0.000079
6	C	0.423144	1.667611	0.000013
6	C	-1.173571	-1.103711	0.000099
6	C	1.449671	0.732686	-0.000047
6	C	0.141878	-1.539777	0.000037
6	C	1.321348	-0.785431	-0.000044
1	H	-1.559951	2.348903	0.000108
1	H	0.730017	2.711051	0.000007
1	H	-1.904336	-1.907531	0.000178
1	H	0.280715	-2.618717	0.000050
6	C	2.824213	1.018251	-0.000126
1	H	3.260800	2.008351	-0.000150
6	C	3.524003	-0.195653	-0.000155
6	C	2.614945	-1.288832	-0.000122
1	H	2.886671	-2.336563	-0.000143
1	H	4.603802	-0.291134	-0.000215
6	C	-3.098338	0.433680	0.000111
1	H	-3.462555	1.457889	0.000205
7	N	-4.043471	-0.478419	0.000041
1	H	-5.020356	-0.210512	0.000076
1	H	-3.847997	-1.472682	-0.000059

Uncorrected Sum of Electronic and Thermal Free Energies = -479.547774
Corrected Sum of Electronic and Thermal Free Energies = -479.547774

Compound 10-NH_2 Triplet

Atomic Number	Atomic Type	Coordinates (Angstroms)		
		X	Y	Z
6	C	-1.726015	0.244295	0.000097
6	C	-0.956746	1.457063	0.000094
6	C	0.410577	1.660522	0.000035
6	C	-1.173973	-1.085573	0.000115
6	C	1.435234	0.692619	-0.000045
6	C	0.140771	-1.510346	0.000054
6	C	1.313965	-0.730087	-0.000041
1	H	-1.549986	2.368812	0.000136
1	H	0.733891	2.698361	0.000042
1	H	-1.893825	-1.899359	0.000212
1	H	0.288236	-2.587636	0.000084
6	C	2.854570	0.988193	-0.000135
1	H	3.273420	1.987959	-0.000156
6	C	3.580481	-0.213768	-0.000189
6	C	2.668082	-1.268107	-0.000129
1	H	2.908578	-2.324912	-0.000146
1	H	4.658406	-0.303799	-0.000263
6	C	-3.124977	0.429077	0.000097
1	H	-3.508790	1.444383	0.000154
7	N	-4.073779	-0.515299	0.000038
1	H	-5.049518	-0.254662	0.000068
1	H	-3.875766	-1.505386	-0.000109

Sum of Electronic and Thermal Free Energies = -479.528661

Compound 10-OH Singlet

Atomic Number	Atomic Type	Coordinates (Angstroms)		
		X	Y	Z
6	C	1.685884	0.232946	0.000022
6	C	1.190469	-1.108208	0.000031
6	C	-0.121045	-1.546406	0.000029
6	C	0.961654	1.465822	0.000010
6	C	-1.301193	-0.789615	0.000007
6	C	-0.409191	1.684129	-0.000009
6	C	-1.435109	0.738531	-0.000014
1	H	1.950354	-1.882458	0.000043
1	H	-0.259635	-2.625209	0.000043
1	H	1.572367	2.365247	0.000013
1	H	-0.724312	2.724932	-0.000019
6	C	-2.592381	-1.295464	-0.000005
1	H	-2.862619	-2.343330	0.000002
6	C	-3.501245	-0.203836	-0.000027
6	C	-2.803244	1.018018	-0.000034
1	H	-3.246672	2.004943	-0.000050
1	H	-4.581263	-0.300369	-0.000039
6	C	3.078754	0.416799	0.000022
1	H	3.504561	1.419918	0.000008
8	O	3.910374	-0.589951	-0.000019
1	H	4.844112	-0.320368	-0.000036

Uncorrected Sum of Electronic and Thermal Free Energies = -499.399273
Corrected Sum of Electronic and Thermal Free Energies = -499.399273

Compound 10-OH Triplet

Atomic Number	Atomic Type	Coordinates (Angstroms)		
		X	Y	Z
6	C	1.727653	0.262262	0.000012
6	C	1.198744	-1.076524	0.000029
6	C	-0.116771	-1.508443	0.000026
6	C	0.963675	1.476598	0.000014
6	C	-1.288926	-0.734260	0.000003
6	C	-0.407645	1.669121	0.000003
6	C	-1.419982	0.695117	-0.000008
1	H	1.950105	-1.859941	0.000046
1	H	-0.258244	-2.586616	0.000042
1	H	1.555077	2.389653	0.000021
1	H	-0.739928	2.704285	0.000002
6	C	-2.637637	-1.276194	-0.000009
1	H	-2.873678	-2.333990	-0.000004
6	C	-3.556907	-0.224311	-0.000030
6	C	-2.841404	0.980982	-0.000028
1	H	-3.267325	1.977687	-0.000039
1	H	-4.634346	-0.321868	-0.000044
6	C	3.119397	0.422433	-0.000002
1	H	3.576556	1.408526	-0.000009
8	O	3.923754	-0.638412	-0.000008
1	H	4.860564	-0.391133	-0.000011

Sum of Electronic and Thermal Free Energies = -499.390789

Compound 10-OCH_3 Singlet

Atomic Number	Atomic Type	Coordinates (Angstroms)		
		X	Y	Z
6	C	-1.190889	0.281734	0.000032
6	C	-0.444237	1.495445	0.000114
6	C	0.934032	1.685929	0.000114
6	C	-0.723767	-1.066222	-0.000071
6	C	1.939140	0.721688	0.000034
6	C	0.580107	-1.530810	-0.000118
6	C	1.775276	-0.799484	-0.000076
1	H	-1.034687	2.408385	0.000186
1	H	1.269242	2.720510	0.000187
1	H	-1.499192	-1.824862	-0.000119
1	H	0.696508	-2.612362	-0.000198
6	C	3.315518	0.974072	0.000039
1	H	3.776926	1.952776	0.000106
6	C	3.988413	-0.259293	-0.000058
6	C	3.057058	-1.331506	-0.000128
1	H	3.305416	-2.384819	-0.000208
1	H	5.066035	-0.378268	-0.000078
6	C	-2.589821	0.498495	0.000062
1	H	-2.986706	1.516060	0.000140
8	O	-3.442477	-0.467526	-0.000001
6	C	-4.878793	-0.201143	0.000034
1	H	-5.281005	-0.672670	-0.896350
1	H	-5.280986	-0.672792	0.896363
1	H	-5.063951	0.874812	0.000109

Uncorrected Sum of Electronic and Thermal Free Energies = -538.687840
Corrected Sum of Electronic and Thermal Free Energies = -538.687840

Compound 10-OCH_3 Triplet

Atomic Number	Atomic Type	Coordinates (Angstroms)		
		X	Y	Z
6	C	-1.231906	0.319258	0.000000
6	C	-0.440498	1.514915	0.000000
6	C	0.934400	1.676565	0.000000
6	C	-0.733227	-1.031074	0.000000
6	C	1.925044	0.678996	0.000000
6	C	0.571163	-1.492756	0.000000
6	C	1.761759	-0.744997	0.000000
1	H	-1.011179	2.441103	0.000000
1	H	1.290680	2.703720	-0.000001
1	H	-1.503713	-1.795690	-0.000001
1	H	0.688748	-2.573851	-0.000001
6	C	3.352768	0.932599	0.000000
1	H	3.800821	1.919646	0.000000
6	C	4.041544	-0.288684	0.000001
6	C	3.098394	-1.318387	0.000000
1	H	3.309213	-2.381555	0.000000
1	H	5.116415	-0.410481	0.000001
6	C	-2.623214	0.512952	0.000000
1	H	-3.051609	1.514422	0.000001
8	O	-3.455349	-0.509476	0.000000
6	C	-4.879431	-0.227133	0.000000
1	H	-5.143614	0.331571	-0.901235
1	H	-5.370129	-1.198165	-0.000002
1	H	-5.143614	0.331569	0.901237

Sum of Electronic and Thermal Free Energies = -538.675884

Compound 10-CH_3 Singlet

Atomic Number	Atomic Type	Coordinates (Angstroms)		
		X	Y	Z
6	C	-1.675784	0.259133	0.000128
6	C	-0.912596	1.481766	0.000095
6	C	0.456793	1.686882	0.000116
6	C	-1.167683	-1.086347	0.000322
6	C	1.478453	0.733075	0.000050
6	C	0.137025	-1.537891	0.000223
6	C	1.328506	-0.796215	-0.000009
1	H	-1.511711	2.388113	0.000019
1	H	0.781150	2.724930	0.000146
1	H	-1.919122	-1.866421	0.000552
1	H	0.264405	-2.618107	0.000320
6	C	2.846118	0.999235	-0.000039
1	H	3.300787	1.980896	-0.000022
6	C	3.531603	-0.232543	-0.000189
6	C	2.612152	-1.316533	-0.000185
1	H	2.873011	-2.366611	-0.000265
1	H	4.610774	-0.340159	-0.000314
6	C	-3.059202	0.459283	-0.000103
1	H	-3.388136	1.498406	-0.000207
6	C	-4.162787	-0.516654	-0.000271
1	H	-3.873358	-1.565643	-0.000042
1	H	-4.806963	-0.317110	0.869490
1	H	-4.806421	-0.317437	-0.870506

Uncorrected Sum of Electronic and Thermal Free Energies = -463.459968
Corrected Sum of Electronic and Thermal Free Energies = -463.459968

Compound 10-CH_3 Triplet

Atomic Number	Atomic Type	Coordinates (Angstroms)		
		X	Y	Z
6	C	1.714788	0.273874	-0.000002
6	C	0.920539	1.477939	-0.000012
6	C	-0.453259	1.663933	-0.000019
6	C	1.171998	-1.060160	-0.000053
6	C	-1.462026	0.690234	-0.000012
6	C	-0.143421	-1.500853	-0.000056
6	C	-1.321136	-0.740443	-0.000023
1	H	1.502477	2.396299	-0.000010
1	H	-0.788209	2.698490	-0.000024
1	H	1.908809	-1.855409	-0.000088
1	H	-0.276195	-2.580272	-0.000087
6	C	-2.884834	0.967135	0.000006
1	H	-3.317812	1.960746	0.000013
6	C	-3.590735	-0.243022	0.000005
6	C	-2.664483	-1.290628	-0.000015
1	H	-2.895102	-2.349624	-0.000019
1	H	-4.667553	-0.348369	0.000018
6	C	3.105800	0.463397	0.000050
1	H	3.444885	1.497882	0.000084
6	C	4.195932	-0.545027	0.000092
1	H	3.870165	-1.585169	-0.000075
1	H	4.841910	-0.386325	-0.874397
1	H	4.841644	-0.386531	0.874819

Sum of Electronic and Thermal Free Energies = -463.457253

Compound 10-F Singlet

Atomic Number	Atomic Type	Coordinates (Angstroms)		
		X	Y	Z
6	C	1.697693	0.239076	-0.000001
6	C	0.968543	1.480038	-0.000001
6	C	-0.398065	1.694098	-0.000001
6	C	1.204333	-1.111435	0.000000
6	C	-1.421575	0.740250	-0.000001
6	C	-0.103354	-1.550501	0.000000
6	C	-1.284599	-0.790584	0.000000
1	H	1.582420	2.377108	0.000000
1	H	-0.718471	2.733092	0.000001
1	H	1.968266	-1.881806	0.000000
1	H	-0.242654	-2.629032	0.000000
6	C	-2.788632	1.017252	0.000003
1	H	-3.234489	2.003071	0.000007
6	C	-3.485583	-0.207419	-0.000002
6	C	-2.574068	-1.298823	0.000000
1	H	-2.843923	-2.346740	0.000000
1	H	-4.565562	-0.305967	-0.000001
6	C	3.069455	0.425793	-0.000001
1	H	3.565106	1.393940	0.000000
9	F	3.909381	-0.574458	0.000001

Uncorrected Sum of Electronic and Thermal Free Energies = -523.402523
Corrected Sum of Electronic and Thermal Free Energies = -523.403161

Compound 10-F Triplet

Atomic Number	Atomic Type	Coordinates (Angstroms)		
		X	Y	Z
6	C	0.639800	-1.634643	0.000000
6	C	-0.796411	-1.585460	0.000000
6	C	-1.647860	-0.491855	0.000000
6	C	1.538714	-0.510828	0.000000
6	C	-1.308612	0.868809	0.000000
6	C	1.254704	0.846226	0.000000
6	C	0.000000	1.471860	0.000000
1	H	-1.288999	-2.555123	0.000000
1	H	-2.710826	-0.720396	0.000000
1	H	2.592716	-0.770792	0.000000
1	H	2.117945	1.507479	0.000000
6	C	-2.265139	1.957159	0.000000
1	H	-3.341501	1.828989	0.000000
6	C	-1.577873	3.178034	0.000000
6	C	-0.205868	2.908481	0.000000
1	H	0.591428	3.642770	0.000000
1	H	-2.031474	4.160329	0.000000
6	C	1.189074	-2.915062	0.000000
1	H	0.628289	-3.843757	0.000000
9	F	2.502139	-3.089537	0.000000

Sum of Electronic and Thermal Free Energies = -523.400834

Compound 10-CHO Singlet

Atomic Number	Atomic Type	Coordinates (Angstroms)		
		X	Y	Z
6	C	-1.392582	0.534782	0.000495
6	C	-0.506614	1.654744	0.000301
6	C	0.883956	1.716209	0.000010
6	C	-0.991425	-0.837899	0.000857
6	C	1.797597	0.660639	-0.000130
6	C	0.283481	-1.397153	0.000770
6	C	1.523801	-0.754527	0.000219
1	H	-0.999274	2.623734	0.000327
1	H	1.309131	2.717131	-0.000168
1	H	-1.815349	-1.544131	0.001185
1	H	0.312064	-2.484455	0.001130
6	C	3.242708	0.806463	-0.000591
1	H	3.765117	1.755785	-0.000922
6	C	3.830449	-0.465671	-0.000506
6	C	2.810574	-1.427485	-0.000017
1	H	2.941187	-2.503178	0.000154
1	H	4.892890	-0.671104	-0.000789
6	C	-2.786895	0.863028	0.000416
1	H	-3.042305	1.920040	0.000912
6	C	-3.939633	-0.042093	-0.000557
8	O	-3.871931	-1.267920	-0.001078
1	H	-4.920528	0.463314	-0.000809

Uncorrected Sum of Electronic and Thermal Free Energies = -537.465495
Corrected Sum of Electronic and Thermal Free Energies = -537.460306

Compound 10-CHO Triplet

Atomic Number	Atomic Type	Coordinates (Angstroms)		
		X	Y	Z
6	C	-1.394991	0.537407	0.000002
6	C	-0.504387	1.660614	-0.000004
6	C	0.888526	1.719489	-0.000003
6	C	-0.993254	-0.840536	0.000032
6	C	1.797609	0.662407	0.000002
6	C	0.284301	-1.400952	0.000039
6	C	1.521581	-0.757736	0.000019
1	H	-0.996824	2.629559	-0.000019
1	H	1.315739	2.719612	-0.000013
1	H	-1.817983	-1.545630	0.000047
1	H	0.312738	-2.488327	0.000067
6	C	3.239734	0.803418	-0.000013
1	H	3.765274	1.751275	-0.000027
6	C	3.825938	-0.467748	0.000001
6	C	2.806701	-1.427529	0.000023
1	H	2.938965	-2.503244	0.000037
1	H	4.888350	-0.673901	-0.000005
6	C	-2.779118	0.863427	-0.000011
1	H	-3.037081	1.919910	0.000002
6	C	-3.936627	-0.042452	-0.000050
8	O	-3.873675	-1.266849	-0.000048
1	H	-4.915864	0.466692	0.000069

Sum of Electronic and Thermal Free Energies = -537.470489

Compound 10-CF_3 Singlet

Atomic Number	Atomic Type	Coordinates (Angstroms)		
		X	Y	Z
6	C	-0.640510	0.544747	-0.000023
6	C	0.251993	1.694174	0.000017
6	C	1.631683	1.759528	0.000071
6	C	-0.253369	-0.852884	0.000233
6	C	2.551155	0.701811	0.000025
6	C	1.007420	-1.417191	0.000205
6	C	2.258792	-0.782613	-0.000002
1	H	-0.256285	2.654216	-0.000019
1	H	2.059516	2.759187	0.000122
1	H	-1.071971	-1.561875	0.000400
1	H	1.035467	-2.504336	0.000334
6	C	3.950047	0.837963	-0.000044
1	H	4.492100	1.774872	-0.000022
6	C	4.521883	-0.449200	-0.000147
6	C	3.498944	-1.431813	-0.000134
1	H	3.650950	-2.503632	-0.000197
1	H	5.585272	-0.659416	-0.000238
6	C	-1.977324	0.878033	-0.000280
1	H	-2.276505	1.922360	-0.000356
6	C	-3.152469	-0.081413	-0.000126
9	F	-4.300338	0.608792	-0.001635
9	F	-3.132991	-0.877298	-1.091811
9	F	-3.134227	-0.874632	1.093580

Uncorrected Sum of Electronic and Thermal Free Energies = -761.194914
Corrected Sum of Electronic and Thermal Free Energies = -761.195819

Compound 10-CF_3 Triplet

Atomic Number	Atomic Type	Coordinates (Angstroms)		
		X	Y	Z
6	C	-0.655420	0.568367	-0.000325
6	C	0.244920	1.691475	-0.000378
6	C	1.634295	1.741322	-0.000227
6	C	-0.255875	-0.815150	-0.000629
6	C	2.538975	0.676467	0.000018
6	C	1.014854	-1.381083	-0.000514
6	C	2.256607	-0.740702	-0.000100
1	H	-0.244791	2.661668	-0.000500
1	H	2.067934	2.738570	-0.000254
1	H	-1.066357	-1.534427	-0.000994
1	H	1.039638	-2.468299	-0.000744
6	C	3.981034	0.810669	0.000347
1	H	4.511226	1.756021	0.000487
6	C	4.562294	-0.463666	0.000456
6	C	3.538044	-1.417557	0.000163
1	H	3.664854	-2.493997	0.000138
1	H	5.623679	-0.674789	0.000718
6	C	-2.019049	0.897904	-0.000076
1	H	-2.324961	1.938871	0.000003
6	C	-3.163478	-0.082111	0.000187
9	F	-4.333268	0.571334	0.000414
9	F	-3.129971	-0.887891	-1.090785
9	F	-3.129476	-0.887800	1.091217

Sum of Electronic and Thermal Free Energies = -761.200604

Compound 10-CN Singlet

Atomic Number	Atomic Type	Coordinates (Angstroms)		
		X	Y	Z
6	C	1.421156	0.482283	-0.000109
6	C	0.555142	1.641897	-0.000069
6	C	-0.825322	1.721573	-0.000002
6	C	1.027032	-0.906891	-0.000072
6	C	-1.755161	0.673567	0.000052
6	C	-0.238118	-1.463216	-0.000004
6	C	-1.481123	-0.813645	0.000051
1	H	1.076566	2.595044	-0.000098
1	H	-1.242965	2.725521	0.000012
1	H	1.850177	-1.615326	-0.000106
1	H	-0.276176	-2.550044	0.000007
6	C	-3.153242	0.826083	0.000121
1	H	-3.683590	1.769649	0.000140
6	C	-3.740824	-0.453686	0.000159
6	C	-2.729879	-1.447978	0.000118
1	H	-2.893928	-2.518006	0.000136
1	H	-4.806594	-0.650889	0.000214
6	C	2.781014	0.778979	-0.000172
1	H	3.110186	1.815789	-0.000095
6	C	3.815530	-0.182493	-0.000088
7	N	4.687012	-0.958653	-0.000018

Uncorrected Sum of Electronic and Thermal Free Energies = -516.389469
Corrected Sum of Electronic and Thermal Free Energies = -516.389986

Compound 10-NO_2 Singlet

Atomic Number	Atomic Type	Coordinates (Angstroms)		
		X	Y	Z
6	C	-1.015848	0.502456	-0.068428
6	C	-0.142583	1.660657	-0.104028
6	C	1.236839	1.734293	-0.085520
6	C	-0.632780	-0.891099	-0.005786
6	C	2.161167	0.683370	-0.018920
6	C	0.629324	-1.447727	0.046608
6	C	1.877041	-0.804807	0.045016
1	H	-0.658453	2.615461	-0.154202
1	H	1.658958	2.735512	-0.125865
1	H	-1.451873	-1.601604	-0.008232
1	H	0.662889	-2.533691	0.092471
6	C	3.557550	0.828379	0.001188
1	H	4.094710	1.767450	-0.032277
6	C	4.135916	-0.453856	0.071828
6	C	3.118857	-1.443555	0.098136
1	H	3.278323	-2.512989	0.149936
1	H	5.200357	-0.656826	0.101743
6	C	-2.352610	0.832664	-0.082634
1	H	-2.728891	1.850111	-0.103498
7	N	-3.432532	-0.145085	0.000011
8	O	-3.616834	-0.880500	-0.959862
8	O	-4.066357	-0.101060	1.046748

Uncorrected Sum of Electronic and Thermal Free Energies = -628.637603
Corrected Sum of Electronic and Thermal Free Energies = -628.639291

Compound 10-NO_2 Triplet

Atomic Number	Atomic Type	Coordinates (Angstroms)		
		X	Y	Z
6	C	-1.025685	0.441463	-0.058224
6	C	-0.175409	1.600235	-0.096627
6	C	1.212211	1.707860	-0.085028
6	C	-0.577539	-0.922017	0.011585
6	C	2.158706	0.684377	-0.021266
6	C	0.718247	-1.430942	0.066808
6	C	1.932466	-0.743319	0.050201
1	H	-0.701329	2.550017	-0.146965
1	H	1.602856	2.721660	-0.130741
1	H	-1.368469	-1.662292	0.013629
1	H	0.785479	-2.514907	0.124796
6	C	3.593591	0.876628	-0.010812
1	H	4.086204	1.841133	-0.053852
6	C	4.224323	-0.371820	0.063016
6	C	3.239413	-1.366148	0.101017
1	H	3.409772	-2.434996	0.158783
1	H	5.293274	-0.539450	0.086867
6	C	-2.396735	0.761949	-0.073311
1	H	-2.746076	1.786794	-0.068220
7	N	-3.506022	-0.163305	-0.008455
8	O	-3.381106	-1.306879	-0.454854
8	O	-4.524031	0.302578	0.503696

Sum of Electronic and Thermal Free Energies = -628.643815

Compound 11 Singlet

Atomic Number	Atomic Type	Coordinates (Angstroms)		
		X	Y	Z
6	C	2.749629	-0.423541	0.000169
6	C	2.396368	0.973145	0.000249
6	C	1.156568	1.576399	0.000203
6	C	1.825444	-1.521079	0.000016
6	C	-0.108692	0.957240	0.000065
6	C	0.439154	-1.532080	-0.000078
6	C	-0.429021	-0.433008	-0.000059
1	H	3.244177	1.652935	0.000369
1	H	1.147826	2.663288	0.000297
1	H	2.291133	-2.503280	-0.000031
1	H	-0.031065	-2.512759	-0.000208
6	C	-1.369426	1.664663	0.000062
6	C	-2.446574	0.743167	-0.000067
6	C	-1.894834	-0.535523	-0.000253
1	H	-3.502358	0.982064	-0.000073
6	C	4.113716	-0.745862	0.000243
1	H	4.874901	0.026830	0.000352
1	H	4.448793	-1.777380	0.000187
8	O	-1.401515	2.988935	0.000207
1	H	-2.304133	3.346995	0.000206
6	C	-2.595617	-1.762310	-0.000441
7	N	-3.139634	-2.793920	-0.000487

Uncorrected Sum of Electronic and Thermal Free Energies = -591.622704
Corrected Sum of Electronic and Thermal Free Energies = -591.617624

Compound 11 Triplet

Atomic Number	Atomic Type	Coordinates (Angstroms)		
		X	Y	Z
6	C	2.754318	-0.425007	0.000155
6	C	2.397136	0.969908	0.000267
6	C	1.148371	1.575291	0.000236
6	C	1.826733	-1.530051	-0.000022
6	C	-0.104719	0.955143	0.000083
6	C	0.441749	-1.542067	-0.000123
6	C	-0.429578	-0.444496	-0.000076
1	H	3.242274	1.653126	0.000393
1	H	1.140039	2.662330	0.000339
1	H	2.295993	-2.510312	-0.000084
1	H	-0.027459	-2.523296	-0.000251
6	C	-1.372475	1.665372	0.000058
6	C	-2.440216	0.746317	-0.000114
6	C	-1.882222	-0.535880	-0.000175
1	H	-3.497220	0.979505	-0.000175
6	C	4.114778	-0.733033	0.000223
1	H	4.869698	0.045819	0.000353
1	H	4.460795	-1.761080	0.000144
8	O	-1.402544	2.987096	0.000178
1	H	-2.304748	3.346989	0.000140
6	C	-2.596972	-1.757194	-0.000335
7	N	-3.157206	-2.779380	-0.000477

Sum of Electronic and Thermal Free Energies = -591.628197

Compound 12 Singlet

Atomic Number	Atomic Type	Coordinates (Angstroms)		
		X	Y	Z
6	C	-1.760035	0.511247	0.000140
6	C	-1.195339	-0.803610	0.000094
6	C	0.132236	-1.194787	0.000036
6	C	-1.036816	1.739288	0.000120
6	C	1.275983	-0.384253	0.000009
6	C	0.334132	1.980705	0.000072
6	C	1.375618	1.051229	0.000026
1	H	-1.917290	-1.615355	0.000116
1	H	0.313498	-2.266807	0.000021
1	H	-1.655470	2.633051	0.000142
1	H	0.627512	3.027789	0.000068
6	C	2.633402	-0.868561	-0.000031
6	C	3.542028	0.235062	-0.000074
6	C	2.791059	1.391892	-0.000024
1	H	3.176737	2.403739	-0.000032
1	H	4.622719	0.165248	-0.000121
6	C	-3.182226	0.645031	0.000172
1	H	-3.629550	1.634777	-0.000033
8	O	2.895770	-2.164806	-0.000047
1	H	3.847146	-2.359860	-0.000102
6	C	-4.065698	-0.442501	-0.000100
7	N	-4.802504	-1.352655	-0.000334

Uncorrected Sum of Electronic and Thermal Free Energies = -591.627044
Corrected Sum of Electronic and Thermal Free Energies = -591.622212

Compound 12 Triplet

Atomic Number	Atomic Type	Coordinates (Angstroms)		
		X	Y	Z
6	C	1.763506	0.518339	-0.000035
6	C	1.197178	-0.796272	-0.000061
6	C	-0.139391	-1.191173	-0.000032
6	C	1.036145	1.754620	-0.000025
6	C	-1.271168	-0.381957	0.000011
6	C	-0.332726	1.993688	0.000006
6	C	-1.375528	1.062549	0.000029
1	H	1.917715	-1.609433	-0.000099
1	H	-0.318956	-2.263560	-0.000045
1	H	1.657125	2.646440	-0.000039
1	H	-0.628224	3.040272	0.000013
6	C	-2.638157	-0.871860	0.000049
6	C	-3.537119	0.222487	0.000087
6	C	-2.780716	1.386111	0.000067
1	H	-3.176433	2.394639	0.000084
1	H	-4.618096	0.158089	0.000120
6	C	3.177427	0.639235	-0.000029
1	H	3.633009	1.625469	-0.000027
8	O	-2.890229	-2.168276	0.000037
1	H	-3.840130	-2.371219	0.000108
6	C	4.058675	-0.454768	-0.000052
7	N	4.792438	-1.365783	-0.000072

Sum of Electronic and Thermal Free Energies = -591.632725

Compound 13a Singlet

Atomic Number	Atomic Type	Coordinates (Angstroms)		
		X	Y	Z
6	C	-2.691485	-0.409738	0.000000
6	C	-1.722445	0.585213	0.000001
6	C	-0.376861	0.183209	0.000001
6	C	0.049442	-1.166539	0.000000
6	C	-0.978400	-2.176172	0.000002
6	C	-2.300090	-1.787651	0.000002
1	H	-3.750350	-0.166047	-0.000001
6	C	0.474377	1.341390	0.000002
6	C	1.424472	-1.363099	-0.000006
1	H	-0.708502	-3.227399	0.000000
1	H	-3.078437	-2.543344	0.000001
6	C	2.329071	-0.228431	-0.000002
6	C	1.823670	1.144401	0.000004
1	H	1.849528	-2.363666	-0.000008
1	H	2.540222	1.960490	0.000004
6	C	-1.715207	2.062195	-0.000002
6	C	-0.427106	2.504221	0.000000
1	H	-2.600662	2.685260	-0.000002
1	H	-0.105340	3.537377	0.000001
6	C	3.674935	-0.476466	-0.000019
1	H	4.401023	0.332006	0.000029
1	H	4.066282	-1.489871	0.000083

Uncorrected Sum of Electronic and Thermal Free Energies = -500.400310
Corrected Sum of Electronic and Thermal Free Energies = -500.400310

Compound 13a Triplet

Atomic Number	Atomic Type	Coordinates (Angstroms)		
		X	Y	Z
6	C	-2.768435	-0.378114	-0.000007
6	C	-1.755873	0.604087	-0.000005
6	C	-0.385266	0.174886	0.000003
6	C	0.041661	-1.153419	0.000007
6	C	-1.015904	-2.118006	0.000005
6	C	-2.368528	-1.721301	-0.000001
1	H	-3.820683	-0.114019	-0.000013
6	C	0.494693	1.310284	0.000006
6	C	1.442869	-1.365669	0.000008
1	H	-0.779501	-3.178343	0.000007
1	H	-3.129548	-2.494710	-0.000003
6	C	2.362077	-0.262853	0.000011
6	C	1.856402	1.095704	0.000013
1	H	1.842886	-2.375006	0.000011
1	H	2.565063	1.919130	0.000016
6	C	-1.724226	2.019390	-0.000010
6	C	-0.365957	2.457678	-0.000001
1	H	-2.582872	2.680817	-0.000015
1	H	-0.053588	3.495901	-0.000001
6	C	3.745705	-0.468217	0.000026
1	H	4.436795	0.367216	-0.000095
1	H	4.166134	-1.467687	-0.000231

Sum of Electronic and Thermal Free Energies = -500.369596

Compound 13b Singlet

Atomic Number	Atomic Type	Coordinates (Angstroms)		
		X	Y	Z
6	C	-2.762377	-0.380526	-0.000008
6	C	-1.747289	0.585996	-0.000006
6	C	-0.410495	0.168505	-0.000001
6	C	0.054267	-1.168632	0.000001
6	C	-1.011748	-2.136606	-0.000001
6	C	-2.348697	-1.739718	-0.000005
1	H	-3.819124	-0.123929	-0.000011
6	C	0.468907	1.292664	0.000000
6	C	1.443777	-1.376474	0.000005
1	H	-0.771814	-3.199268	0.000000
1	H	-3.115928	-2.513841	-0.000007
6	C	2.383214	-0.277566	0.000007
6	C	1.822114	1.077643	0.000004
1	H	1.841189	-2.390434	0.000007
1	H	2.525008	1.911508	0.000005
6	C	-1.706367	2.061639	-0.000007
6	C	-0.401481	2.480763	-0.000002
1	H	-2.578417	2.709284	-0.000010
1	H	-0.061443	3.511465	-0.000002
6	C	3.769348	-0.415414	0.000009
1	H	4.418920	0.455056	0.000020
1	H	4.242561	-1.393484	0.000017

Uncorrected Sum of Electronic and Thermal Free Energies = -500.694159
Corrected Sum of Electronic and Thermal Free Energies = -500.694159

Compound 13b Triplet

C	-2.741475	-0.437639	-0.000008
C	-1.769030	0.591595	-0.000006
C	-0.397466	0.191269	-0.000001
C	0.049234	-1.146547	0.000001
C	-0.962899	-2.153488	-0.000001
C	-2.320532	-1.779217	-0.000006
H	-3.806724	-0.209288	-0.000012
C	0.453561	1.338154	0.000000
C	1.457434	-1.333426	0.000006
H	-0.686139	-3.206250	0.000001
H	-3.077000	-2.563400	-0.000007
C	2.364237	-0.211850	0.000008
C	1.849063	1.124441	0.000005
H	1.876174	-2.338732	0.000008
H	2.554705	1.954116	0.000006
C	-1.762585	2.027778	-0.000008
C	-0.425897	2.474913	-0.000003
H	-2.641628	2.665467	-0.000011
H	-0.110975	3.514509	-0.000003
C	3.752869	-0.466069	0.000012
H	4.474949	0.345903	0.000012
H	4.137554	-1.481817	0.000015

Sum of Electronic and Thermal Free Energies = -500.682927

Compound 14a Singlet

Atomic Number	Atomic Type	Coordinates (Angstroms)		
		X	Y	Z
6	C	1.475621	-2.077685	-0.000001
6	C	0.213216	-1.435684	-0.000001
6	C	0.170763	0.000506	-0.000004
6	C	1.313469	0.818631	0.000000
6	C	2.551749	0.133270	0.000003
6	C	2.618963	-1.277431	0.000002
1	H	1.561021	-3.159983	0.000002
6	C	-1.145394	0.476153	-0.000007
6	C	1.085672	2.227026	0.000001
1	H	3.480207	0.698902	0.000005
1	H	3.595857	-1.749075	0.000005
6	C	-0.214564	2.709644	0.000000
6	C	-1.347669	1.848621	-0.000004
1	H	1.923970	2.916870	0.000006
1	H	-2.342467	2.284354	-0.000006
6	C	-1.111758	-1.881999	0.000006
6	C	-2.007336	-0.733883	-0.000007
1	H	-1.448139	-2.913439	0.000012
6	C	-3.359245	-0.827222	-0.000015
1	H	-0.380850	3.781687	0.000001
1	H	-3.994237	0.053723	0.000162
1	H	-3.856294	-1.792720	-0.000028

Uncorrected Sum of Electronic and Thermal Free Energies = -500.419995
Corrected Sum of Electronic and Thermal Free Energies = -500.419995

Compound 14a Triplet

Atomic Number	Atomic Type	Coordinates (Angstroms)		
		X	Y	Z
6	C	1.611343	-1.977482	0.000002
6	C	0.317540	-1.423489	-0.000001
6	C	0.203799	-0.022061	-0.000001
6	C	1.293808	0.866857	-0.000001
6	C	2.597086	0.283545	0.000002
6	C	2.722563	-1.108630	0.000004
1	H	1.765024	-3.051622	-0.000003
6	C	-1.175313	0.379663	0.000001
6	C	0.952247	2.234920	-0.000003
1	H	3.480624	0.913496	-0.000002
1	H	3.717498	-1.542052	-0.000003
6	C	-0.418367	2.654775	-0.000002
6	C	-1.479937	1.758606	0.000002
1	H	1.728507	2.995496	-0.000002
1	H	-2.502758	2.119633	0.000011
6	C	-1.033065	-1.950638	-0.000008
6	C	-1.981036	-0.841129	0.000008
1	H	-1.311620	-2.997083	-0.000014
6	C	-3.348561	-0.904039	0.000003
1	H	-0.623302	3.720767	0.000001
1	H	-3.958884	-0.007585	-0.000029
1	H	-3.867734	-1.856430	0.000009

Sum of Electronic and Thermal Free Energies = -500.375466

Compound 14b Singlet

Atomic Number	Atomic Type	Coordinates (Angstroms)		
		X	Y	Z
6	C	1.549367	-2.023314	-0.000007
6	C	0.249695	-1.463793	-0.000001
6	C	0.195238	-0.015584	0.000000
6	C	1.308368	0.845523	-0.000005
6	C	2.604338	0.228689	-0.000011
6	C	2.674431	-1.165712	-0.000012
1	H	1.699731	-3.101313	-0.000006
6	C	-1.145628	0.412166	0.000005
6	C	1.003472	2.235887	-0.000002
1	H	3.507612	0.834131	-0.000015
1	H	3.662128	-1.628974	-0.000016
6	C	-0.319921	2.676260	0.000004
6	C	-1.414048	1.775812	0.000008
1	H	1.813178	2.964756	-0.000005
1	H	-2.432255	2.159108	0.000011
6	C	-1.080616	-1.939871	0.000007
6	C	-1.999126	-0.827761	0.000005
1	H	-1.386584	-2.980612	0.000012
6	C	-3.370343	-0.805143	0.000008
1	H	-0.519106	3.746937	0.000005
1	H	-3.922937	0.129986	0.000007
1	H	-3.953117	-1.722966	0.000011

Uncorrected Sum of Electronic and Thermal Free Energies = -500.711934
Corrected Sum of Electronic and Thermal Free Energies = -500.711934

Compound 14b Triplet

C	-1.554949	-2.036712	-0.000003
C	-0.270359	-1.436736	0.000007
C	-0.191345	-0.012092	0.000004
C	-1.309132	0.848005	-0.000004
C	-2.582508	0.205485	-0.000012
C	-2.682901	-1.205134	-0.000013
H	-1.674669	-3.119052	0.000002
C	1.166680	0.413877	0.000009
C	-1.024951	2.249767	-0.000002
H	-3.491000	0.806264	-0.000018
H	-3.673506	-1.657482	-0.000017
C	0.316596	2.688949	0.000006
C	1.412059	1.811760	0.000011
H	-1.837334	2.974065	-0.000008
H	2.424467	2.211088	0.000010
C	1.080439	-1.902809	0.000030
C	1.986931	-0.781558	-0.000003
H	1.397837	-2.942196	0.000043
C	3.392503	-0.876177	-0.000023
H	0.509432	3.761664	0.000004
H	4.021883	0.008696	-0.000037
H	3.888510	-1.842798	-0.000022

Sum of Electronic and Thermal Free Energies = -500.683595

Compound 15a Singlet

Atomic Number	Atomic Type	Coordinates (Angstroms)		
		X	Y	Z
6	C	0.000037	1.220240	-0.000004
6	C	-0.719043	0.038176	0.000018
6	C	0.719059	0.038182	0.000226
6	C	1.953819	-0.570508	-0.000077
1	H	2.856960	0.032210	-0.000805
1	H	0.000059	2.305763	0.000225
6	C	-1.953867	-0.570483	-0.000076
1	H	2.044038	-1.652014	0.000261
1	H	-2.044166	-1.651974	0.000361
1	H	-2.856922	0.032366	-0.000565

Uncorrected Sum of Electronic and Thermal Free Energies = -193.073354
Corrected Sum of Electronic and Thermal Free Energies = -193.079024

Compound 15a Triplet

Atomic Number	Atomic Type	Coordinates (Angstroms)		
		X	Y	Z
6	C	0.000018	1.283055	0.000012
6	C	0.700243	0.091357	-0.002935
6	C	-0.700261	0.091390	0.002914
6	C	-1.918725	-0.628361	0.007894
1	H	-2.858620	-0.106455	-0.137834
1	H	-0.000019	2.365017	-0.000025
6	C	1.918721	-0.628355	-0.007886
1	H	-1.917183	-1.703321	0.151751
1	H	1.917246	-1.703315	-0.151756
1	H	2.858604	-0.106434	0.137868

Sum of Electronic and Thermal Free Energies = -193.058262

Compound 15b Singlet

Atomic Number	Atomic Type	Coordinates (Angstroms)		
		X	Y	Z
6	C	0.000001	1.292042	0.000123
6	C	0.731833	0.106815	0.000010
6	C	-0.731833	0.106816	0.000038
6	C	-1.878304	-0.636003	0.000005
1	H	-2.860352	-0.165741	-0.000010
1	H	0.000001	2.373508	-0.000066
6	C	1.878303	-0.636003	-0.000104
1	H	-1.840118	-1.722013	-0.000078
1	H	1.840119	-1.722014	-0.000150
1	H	2.860353	-0.165744	-0.000132

Uncorrected Sum of Electronic and Thermal Free Energies = -193.409159
Corrected Sum of Electronic and Thermal Free Energies = -193.409159

Compound 15b Triplet

Atomic Number	Atomic Type	Coordinates (Angstroms)		
		X	Y	Z
6	C	-0.000208	1.322834	-0.083144
6	C	0.692279	0.033453	-0.055857
6	C	-0.692250	0.033342	-0.055150
6	C	-1.927544	-0.589330	0.033039
1	H	-2.842064	-0.027850	-0.143736
1	H	0.000278	2.117670	0.704786
6	C	1.927647	-0.589196	0.033271
1	H	-2.016923	-1.664230	0.174626
1	H	2.016912	-1.663926	0.176322
1	H	2.842250	-0.028276	-0.144951

Sum of Electronic and Thermal Free Energies = -193.330440

Compound 16a Singlet

Atomic Number	Atomic Type	Coordinates (Angstroms)		
		X	Y	Z
6	C	-1.694593	-0.483765	-0.000845
6	C	-1.326149	0.816708	0.000448
6	C	-0.345602	-0.165146	0.000287
6	C	1.019026	-0.575625	0.000771
1	H	1.217647	-1.642729	0.001769
6	C	2.014324	0.337172	-0.000551
1	H	3.054444	0.025436	-0.001012
1	H	-2.482408	-1.226598	-0.001396
1	H	-1.610257	1.861675	0.001452
1	H	1.818541	1.406157	-0.001473

Uncorrected Sum of Electronic and Thermal Free Energies = -193.124244
Corrected Sum of Electronic and Thermal Free Energies = -193.124244

Compound 16a Triplet

Atomic Number	Atomic Type	Coordinates (Angstroms)		
		X	Y	Z
6	C	-1.736966	-0.445256	0.000072
6	C	-1.326218	0.845122	-0.000064
6	C	-0.372617	-0.168476	0.000147
6	C	0.975827	-0.596530	-0.000168
1	H	1.140667	-1.677142	-0.000628
6	C	2.096083	0.321603	0.000093
1	H	2.583953	0.600327	-0.933461
1	H	-2.550267	-1.160534	0.000110
1	H	-1.575186	1.899014	-0.000262
1	H	2.584181	0.599553	0.933756

Sum of Electronic and Thermal Free Energies = -193.038196

Compound 16b Singlet

Atomic Number	Atomic Type	Coordinates (Angstroms)		
		X	Y	Z
6	C	1.653979	-0.510369	0.026909
6	C	1.446320	0.885903	-0.167038
6	C	0.343287	-0.205889	-0.010035
6	C	-1.003477	-0.542073	-0.012596
1	H	-1.233688	-1.613132	-0.032850
6	C	-2.077119	0.350805	0.018986
1	H	-3.103841	-0.005292	0.039624
1	H	2.390451	-1.295110	0.190346
1	H	1.679855	1.619115	0.630268
1	H	-1.910725	1.424153	0.035256

Uncorrected Sum of Electronic and Thermal Free Energies = -193.342444
Corrected Sum of Electronic and Thermal Free Energies = -193.344963

Compound 16b Triplet

Atomic Number	Atomic Type	Coordinates (Angstroms)		
		X	Y	Z
6	C	1.750347	-0.529579	0.061116
6	C	1.337403	0.879905	0.104064
6	C	0.368554	-0.157965	0.098024
6	C	-0.965712	-0.543318	0.007326
1	H	-1.153996	-1.621112	-0.018333
6	C	-2.081912	0.302922	-0.040573
1	H	-3.090600	-0.099899	-0.088204
1	H	2.284755	-1.105921	-0.705273
1	H	1.481028	1.731274	-0.567730
1	H	-1.973268	1.383863	-0.000206

Sum of Electronic and Thermal Free Energies = 193.343381

Compound 17a Singlet

Atomic Number	Atomic Type	Coordinates (Angstroms)		
		X	Y	Z
6	C	1.584360	-0.854550	0.000005
6	C	0.720407	-1.905186	0.000036
6	C	-0.720409	-1.905185	0.000017
6	C	1.294530	0.571116	-0.000052
6	C	-1.584361	-0.854548	-0.000036
6	C	0.000001	1.155491	-0.000086
6	C	-1.294530	0.571117	-0.000088
1	H	2.643539	-1.097385	0.000027
1	H	1.175959	-2.891727	0.000081
1	H	-1.175963	-2.891725	0.000048
1	H	-2.643540	-1.097382	-0.000041
6	C	-2.358800	1.475335	-0.000125
1	H	-2.193253	2.547481	0.000873
1	H	-3.388864	1.133224	0.000760
6	C	2.358802	1.475333	-0.000085
1	H	3.388866	1.133222	0.000890
1	H	2.193256	2.547480	-0.000034
1	H	0.000001	2.243270	-0.000120

Uncorrected Sum of Electronic and Thermal Free Energies = -347.935579
Corrected Sum of Electronic and Thermal Free Energies = -347.939268

Compound 17a Triplet

Atomic Number	Atomic Type	Coordinates (Angstroms)		
		X	Y	Z
6	C	1.576738	-0.823070	0.000000
6	C	0.705622	-1.901104	-0.000001
6	C	-0.705623	-1.901103	-0.000001
6	C	1.297358	0.584526	0.000001
6	C	-1.576738	-0.823070	0.000001
6	C	0.000000	1.171418	0.000005
6	C	-1.297358	0.584526	0.000005
1	H	2.633142	-1.080173	-0.000001
1	H	1.172314	-2.882787	-0.000002
1	H	-1.172314	-2.882787	-0.000004
1	H	-2.633142	-1.080173	0.000000
6	C	-2.423609	1.435043	0.000009
1	H	-2.314046	2.514056	-0.000032
1	H	-3.430322	1.033098	-0.000049
6	C	2.423609	1.435043	0.000000
1	H	3.430322	1.033098	-0.000013
1	H	2.314046	2.514056	-0.000015
1	H	0.000000	2.258357	0.000007

Sum of Electronic and Thermal Free Energies = -347.928304

Compound 17b Singlet

Atomic Number	Atomic Type	Coordinates (Angstroms)		
		X	Y	Z
6	C	1.577527	-0.830248	-0.000048
6	C	0.725829	-1.884912	-0.000071
6	C	-0.725829	-1.884912	-0.000064
6	C	1.309297	0.618165	0.000004
6	C	-1.577527	-0.830248	-0.000024
6	C	0.000000	1.179808	0.000113
6	C	-1.309297	0.618165	0.000050
1	H	2.638852	-1.079480	-0.000073
1	H	1.183087	-2.875677	-0.000108
1	H	-1.183087	-2.875677	-0.000113
1	H	-2.638852	-1.079480	-0.000048
6	C	-2.462654	1.403254	0.000069
1	H	-2.409228	2.488772	0.000145
1	H	-3.450928	0.953733	0.000149
6	C	2.462654	1.403254	-0.000051
1	H	3.450928	0.953733	-0.000022
1	H	2.409228	2.488772	-0.000011
1	H	0.000000	2.271343	0.000217

Uncorrected Sum of Electronic and Thermal Free Energies = -348.231508
Corrected Sum of Electronic and Thermal Free Energies = -348.231508

Compound 17b Triplet

Atomic Number	Atomic Type	Coordinates (Angstroms)		
		X	Y	Z
6	C	1.601229	-0.842046	-0.000072
6	C	0.702894	-1.921554	-0.000112
6	C	-0.702894	-1.921554	-0.000080
6	C	1.322128	0.567756	0.000003
6	C	-1.601229	-0.842046	-0.000007
6	C	0.000000	1.157326	0.000041
6	C	-1.322128	0.567756	0.000036
1	H	2.661609	-1.095548	-0.000098
1	H	1.167452	-2.909471	-0.000170
1	H	-1.167452	-2.909471	-0.000118
1	H	-2.661609	-1.095548	0.000014
6	C	-2.383006	1.481228	0.000123
1	H	-2.206404	2.552691	0.000143
1	H	-3.416778	1.144908	0.000111
6	C	2.383006	1.481228	0.000054
1	H	3.416778	1.144908	0.000004
1	H	2.206404	2.552691	0.000105
1	H	0.000000	2.246275	0.000090

Sum of Electronic and Thermal Free Energies = -348.213721

Compound 18a Singlet

Atomic Number	Atomic Type	Coordinates (Angstroms)		
		X	Y	Z
6	C	1.599389	1.226730	-0.363841
6	C	2.188543	0.000284	-0.000275
6	C	1.599882	-1.226226	0.363856
6	C	0.263162	1.568162	-0.274888
6	C	0.263729	-1.568025	0.275369
6	C	-0.858026	0.741386	0.089285
6	C	-0.857669	-0.741619	-0.089083
1	H	2.286377	2.021155	-0.638744
1	H	3.277094	0.000519	-0.000328
1	H	2.287176	-2.020400	0.638786
1	H	0.026924	2.618579	-0.436535
1	H	0.027748	-2.618424	0.437466
6	C	-1.956128	-1.357505	-0.606827
1	H	-2.771567	-0.789250	-1.043070
6	C	-1.956992	1.356852	0.606483
1	H	-2.038994	2.439490	0.631745
1	H	-2.772398	0.788278	1.042380
1	H	-2.037698	-2.440182	-0.632179

Uncorrected Sum of Electronic and Thermal Free Energies = -347.948484
Corrected Sum of Electronic and Thermal Free Energies = -347.948484

Compound 18a Triplet

Atomic Number	Atomic Type	Coordinates (Angstroms)		
		X	Y	Z
6	C	-1.665793	-1.247958	0.000000
6	C	-2.298550	0.000185	0.000001
6	C	-1.665640	1.248097	0.000001
6	C	-0.302386	-1.555625	-0.000001
6	C	-0.302164	1.555698	0.000001
6	C	0.860115	-0.735000	-0.000001
6	C	0.860294	0.734881	0.000001
1	H	-2.326170	-2.111765	0.000000
1	H	-3.384139	0.000175	0.000001
1	H	-2.325683	2.112177	0.000001
1	H	-0.089184	-2.620923	-0.000002
1	H	-0.088996	2.620964	0.000001
6	C	2.085234	1.435107	0.000004
1	H	3.054581	0.958917	0.000016
6	C	2.084810	-1.435380	-0.000002
1	H	2.084568	-2.519698	-0.000001
1	H	3.054196	-0.959300	0.000013
1	H	2.085307	2.519418	-0.000042

Sum of Electronic and Thermal Free Energies = -347.922895

Compound 18b Singlet

Atomic Number	Atomic Type	Coordinates (Angstroms)		
		X	Y	Z
6	C	1.669785	-1.243406	-0.000115
6	C	2.323634	0.000000	0.000017
6	C	1.669785	1.243406	0.000127
6	C	0.316568	-1.552390	-0.000114
6	C	0.316568	1.552390	0.000080
6	C	-0.891906	-0.757580	0.000005
6	C	-0.891906	0.757580	-0.000057
1	H	2.327011	-2.116582	-0.000205
1	H	3.412410	0.000000	0.000034
1	H	2.327011	2.116582	0.000229
1	H	0.105673	-2.623014	-0.000167
1	H	0.105673	2.623014	0.000127
6	C	-2.087949	1.434376	-0.000173
1	H	-3.054059	0.949681	-0.000303
6	C	-2.087949	-1.434376	0.000202
1	H	-2.094718	-2.521946	0.000262
1	H	-3.054059	-0.949680	0.000377
1	H	-2.094717	2.521946	-0.000192

Uncorrected Sum of Electronic and Thermal Free Energies = -348.248821
Corrected Sum of Electronic and Thermal Free Energies = -348.248821

Compound 18b Triplet

Atomic Number	Atomic Type	Coordinates (Angstroms)		
		X	Y	Z
6	C	1.618614	-1.186344	0.454993
6	C	2.219743	-0.000003	0.000002
6	C	1.618615	1.186339	-0.454977
6	C	0.250891	-1.558085	0.288055
6	C	0.250893	1.558087	-0.288081
6	C	-0.849907	-0.752665	-0.098405
6	C	-0.849908	0.752668	0.098409
1	H	2.292344	-1.975813	0.786696
1	H	3.314557	-0.000003	-0.000005
1	H	2.292350	1.975801	-0.786691
1	H	0.045879	-2.633182	0.322737
1	H	0.045881	2.633181	-0.322799
6	C	-1.984074	1.319007	0.663876
1	H	-2.811838	0.708743	1.006841
6	C	-1.984089	-1.319003	-0.663865
1	H	-2.056007	-2.396057	-0.802972
1	H	-2.811848	-0.708734	-1.006834
1	H	-2.055988	2.396061	0.802987

Sum of Electronic and Thermal Free Energies = -348.194576

Compound 19a Singlet

Atomic Number	Atomic Type	Coordinates (Angstroms)		
		X	Y	Z
6	C	-1.989990	-1.140721	0.000000
6	C	-3.075666	-0.331389	-0.000001
6	C	-2.616243	1.105963	0.000002
6	C	-1.270793	1.130681	-0.000002
1	H	-1.967750	-2.221872	-0.000001
1	H	-4.114595	-0.638846	-0.000001
1	H	-3.284282	1.958126	0.000003
1	H	-0.637065	2.005565	-0.000003
6	C	-0.808683	-0.282747	0.000000
6	C	0.498829	-0.757399	0.000001
1	H	0.656876	-1.833283	0.000001
6	C	1.614984	0.082037	0.000000
1	H	1.465494	1.159867	0.000000
6	C	2.954654	-0.390557	0.000000
6	C	4.003958	0.476850	0.000000
1	H	5.030670	0.123785	0.000000
1	H	3.131317	-1.463292	0.000001
1	H	3.853043	1.553640	0.000000

Uncorrected Sum of Electronic and Thermal Free Energies = -347.939831
Corrected Sum of Electronic and Thermal Free Energies = -347.939831

Compound 19a Triplet

Atomic Number	Atomic Type	Coordinates (Angstroms)		
		X	Y	Z
6	C	-1.945528	-1.123313	0.000001
6	C	-3.134713	-0.324995	-0.000001
6	C	-2.757704	1.014455	0.000000
6	C	-1.328478	1.084434	0.000000
1	H	-1.910946	-2.206821	0.000001
1	H	-4.146837	-0.708747	-0.000001
1	H	-3.423161	1.868299	0.000001
1	H	-0.752499	2.001710	0.000001
6	C	-0.799496	-0.255575	0.000000
6	C	0.537734	-0.689388	0.000000
1	H	0.702138	-1.765391	0.000000
6	C	1.659930	0.134410	0.000000
1	H	1.533507	1.215422	0.000000
6	C	2.986276	-0.370910	0.000000
6	C	4.082121	0.444442	0.000000
1	H	5.087768	0.037588	0.000000
1	H	3.124073	-1.450273	0.000000
1	H	3.985113	1.526855	0.000000

Sum of Electronic and Thermal Free Energies = -347.933760

Compound 19b Singlet

Atomic Number	Atomic Type	Coordinates (Angstroms)		
		X	Y	Z
6	C	1.932364	-1.135321	0.000001
6	C	3.127762	-0.415080	-0.000003
6	C	2.800017	0.980495	0.000006
6	C	1.409572	1.108644	-0.000004
1	H	1.827540	-2.217190	0.000000
1	H	4.130778	-0.832132	-0.000007
1	H	3.515516	1.798521	0.000011
1	H	0.853917	2.040847	-0.000006
6	C	0.834290	-0.207053	0.000000
6	C	-0.537302	-0.586432	0.000001
1	H	-0.712022	-1.667597	0.000002
6	C	-1.668499	0.200910	-0.000001
1	H	-1.563706	1.286227	-0.000003
6	C	-2.997805	-0.335009	0.000001
6	C	-4.176926	0.347807	0.000000
1	H	-5.131683	-0.170265	0.000001
1	H	-3.055996	-1.427900	0.000003
1	H	-4.205180	1.435724	-0.000002

Uncorrected Sum of Electronic and Thermal Free Energies = -348.276982
Corrected Sum of Electronic and Thermal Free Energies = -348.276982

Compound 19b Triplet

Atomic Number	Atomic Type	Coordinates (Angstroms)		
		X	Y	Z
6	C	2.031303	-1.111961	0.000001
6	C	3.144328	-0.274317	-0.000002
6	C	2.682938	1.091818	0.000000
6	C	1.291240	1.084440	0.000001
1	H	2.035688	-2.197406	0.000003
1	H	4.184361	-0.587907	-0.000003
1	H	3.319217	1.971944	0.000001
1	H	0.648670	1.957299	0.000002
6	C	0.834519	-0.297610	0.000000
6	C	-0.494668	-0.783257	0.000000
1	H	-0.642346	-1.863448	0.000000
6	C	-1.663350	0.050737	0.000000
1	H	-1.490741	1.128740	-0.000001
6	C	-2.992067	-0.360646	0.000000
6	C	-4.122526	0.493515	0.000000
1	H	-5.132750	0.096062	0.000000
1	H	-3.181073	-1.436818	0.000000
1	H	-4.011329	1.575221	-0.000001

Sum of Electronic and Thermal Free Energies = -348.213750

Compound 20a Singlet

Atomic Number	Atomic Type	Coordinates (Angstroms)		
		X	Y	Z
6	C	1.324513	0.742097	-0.000008
6	C	2.429984	-0.191413	0.000000
6	C	1.939654	-1.489643	0.000011
6	C	0.528217	-1.437334	-0.000021
1	H	3.474941	0.097135	0.000006
1	H	2.532430	-2.395150	0.000023
1	H	-0.116428	-2.308498	-0.000024
6	C	0.088625	-0.087608	-0.000007
6	C	-1.212787	0.377170	0.000001
1	H	-1.361320	1.456355	0.000005
6	C	-2.403402	-0.414645	0.000005
1	H	-2.324196	-1.497919	0.000001
6	C	-3.627430	0.166195	0.000014
1	H	-3.745719	1.246717	0.000017
1	H	-4.535441	-0.428021	0.000018
6	C	1.444153	2.088036	-0.000007
1	H	2.421854	2.560526	0.000283
1	H	0.584717	2.751719	-0.000259

Uncorrected Sum of Electronic and Thermal Free Energies = -347.960727
Corrected Sum of Electronic and Thermal Free Energies = -347.960727

Compound 20a Triplet

Atomic Number	Atomic Type	Coordinates (Angstroms)		
		X	Y	Z
6	C	1.326719	0.754725	0.000017
6	C	2.450950	-0.179449	0.000019
6	C	1.935637	-1.497914	0.000009
6	C	0.544786	-1.463124	-0.000007
1	H	3.496324	0.102512	0.000031
1	H	2.536794	-2.399182	0.000001
1	H	-0.103092	-2.328909	-0.000017
6	C	0.111449	-0.085684	-0.000003
6	C	-1.221617	0.415244	-0.000002
1	H	-1.360747	1.492492	0.000010
6	C	-2.356824	-0.396766	-0.000012
1	H	-2.239121	-1.477571	-0.000019
6	C	-3.654444	0.114987	-0.000011
1	H	-3.837353	1.185780	-0.000003
1	H	-4.515927	-0.543771	-0.000019
6	C	1.391349	2.107253	0.000027
1	H	2.348769	2.618568	-0.000697
1	H	0.506318	2.734442	0.000492

Sum of Electronic and Thermal Free Energies = -347.911240

Compound 20b Singlet

Atomic Number	Atomic Type	Coordinates (Angstroms)		
		X	Y	Z
6	C	1.353658	0.740669	0.000012
6	C	2.478710	-0.176573	0.000006
6	C	1.962481	-1.466539	0.000021
6	C	0.541698	-1.467368	-0.000010
1	H	3.522459	0.115276	-0.000008
1	H	2.568455	-2.371618	0.000026
1	H	-0.086461	-2.349870	-0.000020
6	C	0.092821	-0.120528	0.000001
6	C	-1.200059	0.405905	0.000000
1	H	-1.312322	1.489986	0.000011
6	C	-2.400694	-0.357089	-0.000013
1	H	-2.274971	-1.442290	-0.000022
6	C	-3.689193	0.108763	-0.000016
1	H	-3.909753	1.175065	-0.000008
1	H	-4.537012	-0.570363	-0.000025
6	C	1.392816	2.101593	0.000006
1	H	2.343067	2.631411	0.000006
1	H	0.493120	2.709413	-0.000001

Uncorrected Sum of Electronic and Thermal Free Energies = -348.244214
Corrected Sum of Electronic and Thermal Free Energies = -348.244214

Compound 20b Triplet

Atomic Number	Atomic Type	Coordinates (Angstroms)		
		X	Y	Z
6	C	1.330217	0.726481	0.000005
6	C	2.437959	-0.183296	0.000007
6	C	1.956649	-1.512729	0.000012
6	C	0.550715	-1.460197	-0.000001
1	H	3.481516	0.119591	0.000007
1	H	2.561907	-2.413653	0.000019
1	H	-0.109436	-2.321754	0.000000
6	C	0.124389	-0.091251	0.000001
6	C	-1.210646	0.397958	0.000000
1	H	-1.334584	1.481946	0.000013
6	C	-2.395335	-0.364962	-0.000015
1	H	-2.278316	-1.450812	-0.000031
6	C	-3.693524	0.117645	-0.000011
1	H	-3.901771	1.185849	0.000005
1	H	-4.547170	-0.553786	-0.000024
6	C	1.428693	2.130715	0.000003
1	H	2.400092	2.618278	0.000024
1	H	0.553058	2.772168	-0.000022

Sum of Electronic and Thermal Free Energies = -348.228164

Compound 21a Singlet

Atomic Number	Atomic Type	Coordinates (Angstroms)		
		X	Y	Z
6	C	0.825120	-1.200548	-0.000222
6	C	1.996898	-0.491454	-0.000137
6	C	1.681897	0.978305	0.000067
6	C	0.338381	1.124687	0.000183
1	H	0.706875	-2.275798	-0.000477
1	H	2.999795	-0.903509	-0.000322
1	H	2.427227	1.763445	0.000299
1	H	-0.215323	2.052618	0.000394
6	C	-0.254392	-0.244073	-0.000011
6	C	-1.624362	-0.589836	0.000597
1	H	-1.882162	-1.645588	0.001064
6	C	-2.622046	0.347393	-0.000373
1	H	-2.419025	1.413992	-0.001203
1	H	-3.666361	0.047993	-0.000379

Uncorrected Sum of Electronic and Thermal Free Energies = -270.533532
Corrected Sum of Electronic and Thermal Free Energies = -270.532601

Compound 21a Triplet

Atomic Number	Atomic Type	Coordinates (Angstroms)		
		X	Y	Z
6	C	0.809589	-1.169442	-0.000030
6	C	2.045243	-0.458231	-0.000161
6	C	1.759366	0.914017	-0.000199
6	C	0.342581	1.081900	0.000328
1	H	0.697664	-2.247906	0.000184
1	H	3.030406	-0.907613	-0.000464
1	H	2.484141	1.718789	-0.000297
1	H	-0.170810	2.035593	0.000707
6	C	-0.275203	-0.220853	0.000333
6	C	-1.652065	-0.559955	0.000134
1	H	-1.893296	-1.620373	0.000263
6	C	-2.682217	0.345164	-0.000302
1	H	-2.518698	1.418630	-0.000478
1	H	-3.713174	0.007271	-0.000538

Sum of Electronic and Thermal Free Energies = -270.532601

Compound 21b Singlet

Atomic Number	Atomic Type	Coordinates (Angstroms)		
		X	Y	Z
6	C	-0.802721	-1.162214	0.000016
6	C	-2.037581	-0.496726	-0.000115
6	C	-1.774242	0.906783	-0.000060
6	C	-0.383541	1.095833	0.000034
1	H	-0.649357	-2.239156	0.000049
1	H	-3.019694	-0.962062	-0.000186
1	H	-2.524369	1.694019	-0.000082
1	H	0.129739	2.053046	0.000087
6	C	0.246564	-0.187465	-0.000002
6	C	1.649461	-0.509326	0.000012
1	H	1.857571	-1.584506	-0.000038
6	C	2.737973	0.310016	0.000098
1	H	2.644205	1.393715	0.000168
1	H	3.746417	-0.096468	0.000102

Uncorrected Sum of Electronic and Thermal Free Energies = -270.884069
Corrected Sum of Electronic and Thermal Free Energies = -270.884069

Compound 21b Triplet

Atomic Number	Atomic Type	Coordinates (Angstroms)		
		X	Y	Z
6	C	-0.877968	-1.181139	-0.000055
6	C	-2.047706	-0.424555	0.000018
6	C	-1.696903	0.975300	0.000043
6	C	-0.307200	1.078714	-0.000010
1	H	-0.804081	-2.263887	-0.000113
1	H	-3.062158	-0.815260	0.000025
1	H	-2.404085	1.799648	0.000088
1	H	0.271431	1.994373	-0.000049
6	C	0.262397	-0.274732	-0.000041
6	C	1.622572	-0.622583	-0.000137
1	H	1.890142	-1.678184	-0.000061
6	C	2.692676	0.361812	0.000115
1	H	2.484788	1.426327	0.000162
1	H	3.736753	0.060072	0.000353

Sum of Electronic and Thermal Free Energies = -270.801740

Compound 22a Singlet

Atomic Number	Atomic Type	Coordinates (Angstroms)		
		X	Y	Z
6	C	0.674970	1.281850	0.000242
6	C	-0.675053	1.281883	-0.000204
6	C	-1.154918	-0.117383	0.000045
6	C	-0.000021	-0.958144	0.000192
1	H	1.326320	2.146396	0.000564
1	H	-1.326314	2.146515	-0.000560
6	C	1.154895	-0.117415	0.000284
6	C	2.432098	-0.603688	-0.000436
1	H	3.294301	0.058127	-0.000285
1	H	2.626039	-1.672774	0.000066
6	C	-2.432026	-0.603761	-0.000086
1	H	0.000019	-2.043444	0.000249
1	H	-3.294272	0.057996	-0.000378
1	H	-2.625769	-1.672872	0.000120

Uncorrected Sum of Electronic and Thermal Free Energies = -270.547323
Corrected Sum of Electronic and Thermal Free Energies = -270.547323

Compound 22a Triplet

Atomic Number	Atomic Type	Coordinates (Angstroms)		
		X	Y	Z
6	C	0.707923	1.200141	0.000020
6	C	-0.707894	1.200193	-0.000113
6	C	-1.181034	-0.150784	0.000023
6	C	0.000011	-0.999233	-0.000087
1	H	1.335602	2.085017	0.000111
1	H	-1.335543	2.085092	-0.000186
6	C	1.180970	-0.150770	-0.000025
6	C	2.517058	-0.507491	0.000049
1	H	3.294076	0.249767	0.000300
1	H	2.826745	-1.547464	0.000142
6	C	-2.517040	-0.507485	0.000110
1	H	0.000017	-2.082133	-0.000581
1	H	-3.294092	0.249743	0.000154
1	H	-2.826767	-1.547450	0.000206

Sum of Electronic and Thermal Free Energies = -270.516373

Compound 22b Singlet

Atomic Number	Atomic Type	Coordinates (Angstroms)		
		X	Y	Z
6	C	-0.675705	1.214120	0.000045
6	C	0.675705	1.214120	0.000058
6	C	1.167547	-0.192165	0.000019
6	C	0.000000	-1.008992	-0.000047
1	H	-1.331696	2.080802	0.000077
1	H	1.331696	2.080802	0.000108
6	C	-1.167547	-0.192166	-0.000038
6	C	-2.518206	-0.488231	-0.000075
1	H	-3.262736	0.303805	-0.000070
1	H	-2.883293	-1.512776	-0.000127
6	C	2.518206	-0.488231	0.000039
1	H	0.000002	-2.094383	-0.000094
1	H	3.262736	0.303804	0.000090
1	H	2.883292	-1.512776	0.000007

Uncorrected Sum of Electronic and Thermal Free Energies = -270.853555
Corrected Sum of Electronic and Thermal Free Energies = -270.853555

Compound 22b Triplet

Atomic Number	Atomic Type	Coordinates (Angstroms)		
		X	Y	Z
6	C	-0.704160	1.249861	-0.000012
6	C	0.704158	1.249862	0.000050
6	C	1.177800	-0.107401	0.000020
6	C	0.000001	-0.932385	-0.000054
1	H	-1.346218	2.126648	-0.000057
1	H	1.346212	2.126652	0.000094
6	C	-1.177799	-0.107405	-0.000027
6	C	-2.503615	-0.597003	0.000023
1	H	-3.360353	0.072591	-0.000048
1	H	-2.705766	-1.664959	-0.000225
6	C	2.503615	-0.597003	0.000040
1	H	0.000004	-2.019719	-0.000095
1	H	3.360357	0.072588	0.000097
1	H	2.705760	-1.664960	-0.000005

Sum of Electronic and Thermal Free Energies = -270.825341

Compound 23a Singlet

Atomic Number	Atomic Type	Coordinates (Angstroms)		
		X	Y	Z
6	C	-1.017627	1.134515	-0.000054
6	C	-1.836608	0.000240	-0.000111
6	C	-1.017892	-1.134186	0.000167
6	C	0.368078	-0.744961	0.000111
1	H	-1.364458	2.162728	-0.000113
1	H	-2.919032	0.000482	-0.000306
1	H	-1.364576	-2.162449	0.000348
6	C	0.368239	0.745090	0.000108
6	C	1.419729	1.597394	-0.000050
1	H	1.267884	2.672883	0.000012
6	C	1.419019	-1.597926	-0.000116
1	H	2.448375	1.248734	0.000369
1	H	1.266234	-2.673281	-0.000411
1	H	2.447941	-1.250100	-0.000235

Uncorrected Sum of Electronic and Thermal Free Energies = -270.565083
Corrected Sum of Electronic and Thermal Free Energies = -270.565083

Compound 23a Triplet

Atomic Number	Atomic Type	Coordinates (Angstroms)		
		X	Y	Z
6	C	-1.167799	1.044915	-0.031783
6	C	-1.843016	-0.200734	0.016478
6	C	-0.911401	-1.242576	0.000399
6	C	0.406531	-0.683638	-0.021351
1	H	-1.627364	2.022454	-0.114551
1	H	-2.919272	-0.324142	0.046835
1	H	-1.134902	-2.301677	0.010879
6	C	0.273972	0.786029	-0.009480
6	C	1.275750	1.691846	0.055015
1	H	1.066945	2.757178	0.073799
6	C	1.643235	-1.417224	-0.020106
1	H	2.318763	1.394796	0.100010
1	H	1.726525	-2.351453	0.527746
1	H	2.505679	-1.068869	-0.579756

Sum of Electronic and Thermal Free Energies = -270.497364

Compound 23b Singlet

Atomic Number	Atomic Type	Coordinates (Angstroms)		
		X	Y	Z
6	C	-1.046290	-1.165881	-0.000033
6	C	-1.828801	-0.000013	0.000093
6	C	-1.046312	1.165862	-0.000032
6	C	0.340391	0.767651	0.000025
1	H	-1.403934	-2.189547	-0.000112
1	H	-2.919099	-0.000026	0.000118
1	H	-1.403964	2.189525	-0.000088
6	C	0.340402	-0.767652	-0.000007
6	C	1.457968	-1.556814	-0.000053
1	H	1.376655	-2.642000	-0.000093
6	C	1.457933	1.556841	0.000034
1	H	2.461016	-1.139756	-0.000049
1	H	1.376590	2.642025	0.000014
1	H	2.460995	1.139814	0.000047

Uncorrected Sum of Electronic and Thermal Free Energies = -270.849289
Corrected Sum of Electronic and Thermal Free Energies = -270.849289

Compound 23b Triplet

Atomic Number	Atomic Type	Coordinates (Angstroms)		
		X	Y	Z
6	C	-1.031761	-1.137813	-0.000064
6	C	-1.870312	0.000001	-0.000004
6	C	-1.031760	1.137812	0.000103
6	C	0.342422	0.728586	-0.000004
1	H	-1.363020	-2.173518	-0.000122
1	H	-2.955633	-0.000001	-0.000010
1	H	-1.363015	2.173518	0.000177
6	C	0.342421	-0.728589	0.000000
6	C	1.461983	-1.586236	0.000015
1	H	1.338509	-2.666595	-0.000025
6	C	1.461980	1.586237	-0.000038
1	H	2.477406	-1.201894	0.000007
1	H	1.338498	2.666595	-0.000004
1	H	2.477407	1.201904	-0.000081

Sum of Electronic and Thermal Free Energies = -270.827837

Compound 24a Singlet

Atomic Number	Atomic Type	Coordinates (Angstroms)		
		X	Y	Z
6	C	-0.338787	1.784757	0.000232
6	C	-1.585743	1.121765	0.000068
6	C	-1.435401	-0.317406	-0.000140
6	C	0.018410	-0.527169	0.000253
6	C	0.648380	0.802287	0.000247
6	C	1.006162	-1.453977	0.000667
6	C	2.319709	-0.703616	-0.000522
6	C	2.093140	0.642420	-0.000091
1	H	-2.549191	1.623366	-0.000088
1	H	3.287024	-1.191886	-0.000984
1	H	2.831469	1.432228	-0.000384
1	H	0.926503	-2.534602	0.000503
1	H	-0.201522	2.859048	-0.000753
6	C	-2.472538	-1.194983	-0.000297
1	H	-3.499734	-0.843841	-0.000442
1	H	-2.314548	-2.268770	-0.000360

Uncorrected Sum of Electronic and Thermal Free Energies = -346.737900
Corrected Sum of Electronic and Thermal Free Energies = -346.740259

Compound 24a Triplet

Atomic Number	Atomic Type	Coordinates (Angstroms)		
		X	Y	Z
6	C	-0.428650	1.804755	-0.000157
6	C	-1.639390	1.111229	-0.000138
6	C	-1.404842	-0.342674	0.000056
6	C	0.034211	-0.483194	0.000011
6	C	0.613273	0.847810	-0.000120
6	C	1.089950	-1.430599	-0.000026
6	C	2.299384	-0.700911	0.000232
6	C	2.022423	0.706030	-0.000045
1	H	-2.624199	1.564071	-0.000191
1	H	3.291446	-1.135909	0.000425
1	H	2.765463	1.494223	-0.000004
1	H	1.007806	-2.510304	0.000003
1	H	-0.321220	2.882452	0.000059
6	C	-2.355824	-1.320240	0.000114
1	H	-3.413534	-1.075459	-0.000059
1	H	-2.088975	-2.372317	0.000198

Sum of Electronic and Thermal Free Energies = -346.736132

Compound 24b Singlet

Atomic Number	Atomic Type	Coordinates (Angstroms)		
		X	Y	Z
6	C	0.481627	1.779730	-0.000133
6	C	1.672226	1.102016	-0.000047
6	C	1.399465	-0.367570	0.000084
6	C	-0.059590	-0.482267	-0.000042
6	C	-0.617008	0.828974	0.000018
6	C	-1.123901	-1.411908	-0.000329
6	C	-2.333865	-0.665476	0.000029
6	C	-2.024229	0.725571	0.000275
1	H	2.669324	1.531264	-0.000043
1	H	-3.335033	-1.088725	0.000120
1	H	-2.747552	1.536611	0.000548
1	H	-1.053839	-2.496436	-0.000538
1	H	0.385436	2.864072	-0.000114
6	C	2.362959	-1.325588	0.000114
1	H	3.418936	-1.063093	0.000103
1	H	2.116623	-2.384580	0.000109

Uncorrected Sum of Electronic and Thermal Free Energies = -347.069323
Corrected Sum of Electronic and Thermal Free Energies = -347.069323

Compound 24b Triplet

Atomic Number	Atomic Type	Coordinates (Angstroms)		
		X	Y	Z
6	C	0.330353	1.827096	0.000057
6	C	1.603818	1.127397	-0.000103
6	C	1.430215	-0.293071	-0.000059
6	C	-0.013816	-0.479044	-0.000015
6	C	-0.660337	0.837165	0.000090
6	C	-1.010775	-1.449930	0.000129
6	C	-2.288314	-0.751647	-0.000286
6	C	-2.092351	0.627940	0.000256
1	H	2.577037	1.614414	-0.000165
1	H	-3.257192	-1.246408	-0.000502
1	H	-2.865189	1.388718	0.000407
1	H	-0.891151	-2.528511	0.000227
1	H	0.202940	2.904173	0.000206
6	C	2.451967	-1.254933	-0.000074
1	H	3.498351	-0.960331	-0.000101
1	H	2.230650	-2.317884	-0.000036

Sum of Electronic and Thermal Free Energies = -347.022941

Compound 25a Singlet

Atomic Number	Atomic Type	Coordinates (Angstroms)		
		X	Y	Z
6	C	0.880819	-1.182503	-0.000106
6	C	1.767183	-0.000025	-0.000022
6	C	0.880857	1.182475	-0.000013
6	C	-0.414083	0.733218	0.000029
6	C	-0.414143	-0.733206	-0.000033
6	C	-1.774280	1.156326	0.000114
6	C	-2.583910	0.000028	0.000052
6	C	-1.774348	-1.156318	0.000015
1	H	1.233471	2.208234	0.000017
1	H	-3.666596	0.000130	0.000061
1	H	-2.140180	-2.176877	-0.000016
1	H	1.233266	-2.208329	-0.000065
1	H	-2.140009	2.176922	0.000072
6	C	3.117523	-0.000012	-0.000029
1	H	3.683131	0.926468	-0.000029
1	H	3.683207	-0.926438	-0.000080

Uncorrected Sum of Electronic and Thermal Free Energies = -346.757189
Corrected Sum of Electronic and Thermal Free Energies = -346.757189

Compound 25a Triplet

Atomic Number	Atomic Type	Coordinates (Angstroms)		
		X	Y	Z
6	C	0.889968	-1.197414	0.000042
6	C	1.722920	0.000071	0.000023
6	C	0.890095	1.197017	0.000066
6	C	-0.428664	0.763966	0.000029
6	C	-0.428799	-0.764119	-0.000012
6	C	-1.758002	1.193100	-0.000044
6	C	-2.557643	-0.000023	0.000035
6	C	-1.758383	-1.192688	-0.000050
1	H	1.262973	2.213150	0.000083
1	H	-3.643752	0.000163	0.000008
1	H	-2.141061	-2.204076	-0.000077
1	H	1.262848	-2.213442	0.000091
1	H	-2.140077	2.204620	-0.000139
6	C	3.105616	0.000016	0.000019
1	H	3.668218	0.929033	-0.000439
1	H	3.668210	-0.929004	-0.000173

Sum of Electronic and Thermal Free Energies = -346.712730

Compound 25b Singlet

Atomic Number	Atomic Type	Coordinates (Angstroms)		
		X	Y	Z
6	C	0.889150	1.197205	0.000033
6	C	1.754941	0.000007	0.000040
6	C	0.889147	-1.197199	-0.000038
6	C	-0.418389	-0.759827	-0.000032
6	C	-0.418390	0.759829	0.000010
6	C	-1.783047	-1.182654	-0.000059
6	C	-2.560337	-0.000003	-0.000046
6	C	-1.783042	1.182652	0.000011
1	H	1.265409	-2.214531	-0.000065
1	H	-3.651221	-0.000001	-0.000066
1	H	-2.162344	2.197612	0.000034
1	H	1.265404	2.214539	0.000042
1	H	-2.162347	-2.197613	-0.000065
6	C	3.111093	-0.000007	0.000074
1	H	3.679157	-0.927978	0.000054
1	H	3.679178	0.927951	0.000112

Uncorrected Sum of Electronic and Thermal Free Energies = -347.048834
Corrected Sum of Electronic and Thermal Free Energies = -347.048834

Compound 25b Triplet

Atomic Number	Atomic Type	Coordinates (Angstroms)		
		X	Y	Z
6	C	0.906164	1.164853	0.000050
6	C	1.775077	0.000001	0.000036
6	C	0.906165	-1.164852	-0.000009
6	C	-0.441685	-0.723013	-0.000031
6	C	-0.441686	0.723012	0.000005
6	C	-1.794566	-1.159023	-0.000083
6	C	-2.620241	0.000000	-0.000016
6	C	-1.794566	1.159023	-0.000023
1	H	1.266746	-2.189898	-0.000027
1	H	-3.706335	0.000000	-0.000005
1	H	-2.151691	2.185196	-0.000008
1	H	1.266743	2.189900	0.000079
1	H	-2.151692	-2.185196	-0.000120
6	C	3.172204	0.000000	0.000061
1	H	3.737514	-0.928333	0.000048
1	H	3.737516	0.928331	0.000096

Sum of Electronic and Thermal Free Energies = -347.040031

Compound 26a Singlet

Atomic Number	Atomic Type	Coordinates (Angstroms)		
		X	Y	Z
6	C	-1.327618	-1.018109	0.000003
6	C	-2.730562	-1.161805	0.000004
6	C	-3.537657	-0.040565	0.000000
6	C	-2.944531	1.241999	-0.000006
6	C	-1.572887	1.386235	-0.000007
6	C	0.629075	0.181804	-0.000003
6	C	0.934461	-1.212863	0.000001
6	C	-0.289961	-1.953135	0.000005
7	N	-0.765729	0.285163	-0.000002
1	H	-0.389698	-3.029219	0.000009
1	H	-1.097549	2.353786	-0.000013
1	H	-3.556939	2.136341	-0.000010
1	H	-4.617341	-0.139138	0.000002
1	H	-3.146578	-2.163242	0.000009
6	C	1.682999	1.136278	-0.000005
6	C	2.994205	0.537493	-0.000006
1	H	3.859419	1.192808	-0.000008
6	C	3.181221	-0.837348	-0.000002
1	H	4.182414	-1.256592	-0.000004
7	N	2.164729	-1.723449	0.000001
6	C	1.608377	2.533587	-0.000005
1	H	0.686351	3.097547	0.000094
1	H	2.524189	3.114275	0.000055

Uncorrected Sum of Electronic and Thermal Free Energies = -571.783356
Corrected Sum of Electronic and Thermal Free Energies = -571.783856

Compound 26a Triplet

Atomic Number	Atomic Type	Coordinates (Angstroms)		
		X	Y	Z
6	C	-1.321173	-1.015973	0.000003
6	C	-2.702632	-1.187846	0.000004
6	C	-3.534995	-0.065377	0.000000
6	C	-2.971604	1.216655	-0.000006
6	C	-1.592806	1.368166	-0.000007
6	C	0.639020	0.178080	0.000000
6	C	0.956797	-1.210817	0.000000
6	C	-0.244029	-1.948558	0.000004
7	N	-0.785935	0.278386	-0.000001
1	H	-0.339097	-3.025781	0.000006
1	H	-1.123257	2.338539	-0.000012
1	H	-3.592664	2.104479	-0.000010
1	H	-4.612802	-0.187791	0.000000
1	H	-3.108857	-2.193158	0.000008
6	C	1.671946	1.156626	0.000001
6	C	2.983684	0.567886	-0.000005
1	H	3.842631	1.231052	-0.000007
6	C	3.190456	-0.815868	-0.000005
1	H	4.204184	-1.206464	-0.000008
7	N	2.208775	-1.721082	-0.000001
6	C	1.545538	2.554428	0.000004
1	H	0.607287	3.088855	0.000016
1	H	2.441487	3.164729	0.000045

Sum of Electronic and Thermal Free Energies = -571.785921

Compound 26b Singlet

Atomic Number	Atomic Type	Coordinates (Angstroms)		
		X	Y	Z
6	C	-1.309203	-1.053126	0.000035
6	C	-2.721169	-1.172993	-0.000072
6	C	-3.529057	-0.053728	-0.000094
6	C	-2.934122	1.237080	-0.000016
6	C	-1.563084	1.368766	0.000098
6	C	0.638976	0.158726	-0.000027
6	C	0.958986	-1.235377	-0.000016
6	C	-0.265487	-1.964474	0.000086
7	N	-0.745711	0.265912	0.000130
1	H	-0.363088	-3.041505	0.000371
1	H	-1.072248	2.327099	0.000217
1	H	-3.542056	2.135801	-0.000092
1	H	-4.610946	-0.156123	-0.000201
1	H	-3.144273	-2.173585	-0.000066
6	C	1.658569	1.186682	-0.000040
6	C	2.975166	0.577775	0.000000
1	H	3.841021	1.236787	0.000092
6	C	3.171988	-0.791564	-0.000048
1	H	4.198709	-1.165392	-0.000034
7	N	2.213668	-1.746608	-0.000065
6	C	1.498636	2.563425	0.000017
1	H	0.551750	3.081347	-0.000135
1	H	2.384234	3.193290	-0.000135

Uncorrected Sum of Electronic and Thermal Free Energies = -572.104615
Corrected Sum of Electronic and Thermal Free Energies = -572.104615

Compound 26b Triplet

Atomic Number	Atomic Type	Coordinates (Angstroms)		
		X	Y	Z
6	C	-1.317437	-1.052113	0.049936
6	C	-2.714128	-1.187963	0.095796
6	C	-3.547895	-0.038712	0.044909
6	C	-2.955023	1.228794	-0.120149
6	C	-1.582790	1.394942	-0.171493
6	C	0.626716	0.168856	-0.006060
6	C	0.953572	-1.238691	-0.001992
6	C	-0.254644	-1.970014	0.048396
7	N	-0.749261	0.258846	0.000450
1	H	-0.348015	-3.046572	0.079381
1	H	-1.093153	2.326441	-0.404031
1	H	-3.576610	2.113306	-0.242111
1	H	-4.628805	-0.137980	0.083601
1	H	-3.130835	-2.188858	0.153320
6	C	1.656870	1.141412	0.024660
6	C	2.980374	0.588156	-0.053404
1	H	3.829507	1.267997	-0.055234
6	C	3.199047	-0.783065	-0.082099
1	H	4.218468	-1.166068	-0.131634
7	N	2.221493	-1.715396	-0.041192
6	C	1.525721	2.551599	0.204236
1	H	0.585955	3.035310	0.431683
1	H	2.415585	3.173060	0.173801

Sum of Electronic and Thermal Free Energies = -572.048391

Compound 27a Singlet

Atomic Number	Atomic Type	Coordinates (Angstroms)		
		X	Y	Z
6	C	-0.978096	-0.638166	-0.000005
6	C	-2.440183	-0.673890	-0.000004
6	C	-3.128964	0.598089	0.000013
6	C	-2.454512	1.791468	0.000023
6	C	-1.040021	1.792138	0.000020
6	C	1.054714	0.481642	0.000002
6	C	1.286780	-0.908052	-0.000010
6	C	0.001977	-1.583140	-0.000013
7	N	-0.350233	0.664565	0.000007
1	H	-0.129856	-2.656193	-0.000021
6	C	2.109117	1.385416	0.000008
6	C	3.382463	0.811654	0.000001
6	C	3.526616	-0.588644	-0.000011
1	H	4.520179	-1.027930	-0.000016
7	N	2.503254	-1.453846	-0.000017
1	H	1.979215	2.463111	0.000018
1	H	-0.472996	2.717990	0.000028
1	H	4.265269	1.441200	0.000006
1	H	-2.973686	2.741923	0.000034
6	C	-3.167800	-1.825730	-0.000018
1	H	-4.214924	0.588411	0.000016
1	H	-2.704772	-2.806729	-0.000030
1	H	-4.252123	-1.793539	-0.000011

Uncorrected Sum of Electronic and Thermal Free Energies = -571.806198
Corrected Sum of Electronic and Thermal Free Energies = -571.806198

Compound 27a Triplet

Atomic Number	Atomic Type	Coordinates (Angstroms)		
		X	Y	Z
6	C	-0.986830	-0.572821	-0.000005
6	C	-2.405258	-0.671201	-0.000007
6	C	-3.130568	0.555242	0.000006
6	C	-2.471582	1.786663	0.000018
6	C	-1.088103	1.845075	0.000018
6	C	1.021821	0.488917	0.000005
6	C	1.276993	-0.912009	-0.000009
6	C	0.039409	-1.580974	-0.000015
7	N	-0.384095	0.669352	0.000007
1	H	-0.113429	-2.650605	-0.000026
6	C	2.063814	1.400371	0.000013
6	C	3.344473	0.836145	0.000008
6	C	3.517949	-0.575000	-0.000006
1	H	4.522705	-0.988518	-0.000010
7	N	2.519927	-1.446455	-0.000014
1	H	1.923547	2.476057	0.000024
1	H	-0.517764	2.763909	0.000027
1	H	4.219953	1.476851	0.000014
1	H	-3.033701	2.713321	0.000027
6	C	-3.038910	-1.935311	-0.000019
1	H	-4.214779	0.522049	0.000005
1	H	-2.476048	-2.860040	-0.000025
1	H	-4.120562	-2.003891	-0.000021

Sum of Electronic and Thermal Free Energies = -571.783195

Compound 27b Singlet

Atomic Number	Atomic Type	Coordinates (Angstroms)		
		X	Y	Z
6	C	-0.978654	-0.586989	-0.000004
6	C	-2.444055	-0.680480	-0.000002
6	C	-3.123666	0.584097	0.000011
6	C	-2.432327	1.802569	0.000022
6	C	-1.052683	1.880710	0.000020
6	C	1.009891	0.476172	0.000003
6	C	1.260895	-0.943753	-0.000012
6	C	0.001670	-1.586055	-0.000016
7	N	-0.356110	0.666177	0.000007
1	H	-0.179691	-2.649894	-0.000025
6	C	2.067478	1.378012	0.000010
6	C	3.354941	0.830350	0.000002
6	C	3.514335	-0.572060	-0.000012
1	H	4.521125	-0.991580	-0.000018
7	N	2.514681	-1.456889	-0.000019
1	H	1.910290	2.453397	0.000021
1	H	-0.465125	2.786848	0.000028
1	H	4.231634	1.472278	0.000007
1	H	-2.993857	2.734850	0.000032
6	C	-3.058993	-1.918746	-0.000014
1	H	-4.210856	0.584646	0.000013
1	H	-2.493773	-2.843232	-0.000024
1	H	-4.142739	-1.995299	-0.000013

Uncorrected Sum of Electronic and Thermal Free Energies = -572.100700
Corrected Sum of Electronic and Thermal Free Energies = -572.100700

Compound 27b Triplet

Atomic Number	Atomic Type	Coordinates (Angstroms)		
		X	Y	Z
6	C	-1.001015	-0.628226	-0.000006
6	C	-2.422677	-0.665790	-0.000007
6	C	-3.124256	0.599420	0.000006
6	C	-2.432409	1.811992	0.000017
6	C	-1.041160	1.838576	0.000018
6	C	1.026509	0.470698	0.000005
6	C	1.274214	-0.941963	-0.000009
6	C	0.012480	-1.596044	-0.000015
7	N	-0.340187	0.651457	0.000006
1	H	-0.137397	-2.666854	-0.000026
6	C	2.075346	1.393605	0.000014
6	C	3.380836	0.819471	0.000008
6	C	3.534875	-0.563475	-0.000005
1	H	4.541122	-0.985209	-0.000009
7	N	2.525461	-1.474298	-0.000014
1	H	1.920221	2.468197	0.000024
1	H	-0.455043	2.748787	0.000027
1	H	4.261236	1.457897	0.000015
1	H	-2.968129	2.757207	0.000027
6	C	-3.166995	-1.861898	-0.000019
1	H	-4.210752	0.588000	0.000005
1	H	-2.690682	-2.836131	-0.000028
1	H	-4.251992	-1.830206	-0.000018

Sum of Electronic and Thermal Free Energies = -572.062861

Compound 28a Singlet

Atomic Number	Atomic Type	Coordinates (Angstroms)		
		X	Y	Z
6	C	1.539518	1.093675	0.000225
6	C	2.782056	0.387709	0.000383
6	C	2.509595	-0.971938	-0.000044
6	C	1.082578	-1.152079	-0.000499
1	H	1.422789	2.171284	0.000287
1	H	3.761585	0.847682	0.000851
6	C	0.467517	0.131394	-0.000095
6	C	-2.256209	-0.004413	0.000023
6	C	-1.931140	1.331159	-0.000370
6	C	-0.895911	0.410566	-0.000243
1	H	-2.250874	2.364283	-0.000509
1	H	0.569295	-2.106359	-0.000953
6	C	-3.219901	-1.008955	0.000460
1	H	3.236535	-1.774013	-0.000040
1	H	-2.934753	-2.054529	0.000672
1	H	-4.273198	-0.751051	0.000659

Uncorrected Sum of Electronic and Thermal Free Energies = -346.672474
Corrected Sum of Electronic and Thermal Free Energies = -346.670559

Compound 28a Triplet

Atomic Number	Atomic Type	Coordinates (Angstroms)		
		X	Y	Z
6	C	1.531859	1.095716	-0.000182
6	C	2.781092	0.387895	-0.000038
6	C	2.514507	-0.971674	-0.000021
6	C	1.091893	-1.153389	0.000093
1	H	1.415233	2.173224	-0.000188
1	H	3.758562	0.852624	-0.000080
6	C	0.469526	0.136951	0.000183
6	C	-2.258380	-0.010188	-0.000010
6	C	-1.929673	1.326197	0.000128
6	C	-0.895637	0.408810	-0.000018
1	H	-2.248935	2.359839	0.000241
1	H	0.576876	-2.106892	0.000284
6	C	-3.225312	-1.005807	-0.000134
1	H	3.243906	-1.771157	0.000022
1	H	-2.947919	-2.053501	-0.000161
1	H	-4.276974	-0.741211	-0.000127

Sum of Electronic and Thermal Free Energies = -346.674465

Compound 28b Singlet

Atomic Number	Atomic Type	Coordinates (Angstroms)		
		X	Y	Z
6	C	1.567669	1.101941	0.000214
6	C	2.758976	0.378164	-0.000281
6	C	2.439181	-1.020590	0.000144
6	C	1.051555	-1.151704	-0.000063
1	H	1.464082	2.182543	0.000443
1	H	3.761307	0.797754	-0.000490
6	C	0.477254	0.165393	0.000128
6	C	-2.263660	-0.013939	-0.000033
6	C	-1.926341	1.357151	0.000132
6	C	-0.894013	0.472340	-0.000095
1	H	-2.251096	2.387905	-0.000873
1	H	0.482748	-2.075446	-0.000083
6	C	-3.143791	-1.042407	-0.000053
1	H	3.158897	-1.834259	0.000226
1	H	-2.800287	-2.072174	-0.000282
1	H	-4.216639	-0.864411	0.000496

Uncorrected Sum of Electronic and Thermal Free Energies = -347.023923
Corrected Sum of Electronic and Thermal Free Energies = -347.023923

Compound 28b Triplet

Atomic Number	Atomic Type	Coordinates (Angstroms)		
		X	Y	Z
6	C	1.520277	1.099625	-0.072885
6	C	2.749007	0.447869	-0.047864
6	C	2.505845	-0.970458	0.038300
6	C	1.129735	-1.181855	0.071151
1	H	1.344166	2.167836	-0.134036
1	H	3.727109	0.918758	-0.080833
6	C	0.475483	0.102417	0.001607
6	C	-2.224047	-0.032764	-0.036109
6	C	-1.952943	1.399283	-0.000477
6	C	-0.902235	0.361292	-0.002216
1	H	-2.220393	2.115418	0.797865
1	H	0.618441	-2.136045	0.136886
6	C	-3.217212	-0.988385	-0.054564
1	H	3.272192	-1.739600	0.075219
1	H	-2.988027	-2.048988	0.006807
1	H	-4.256957	-0.699518	-0.183569

Sum of Electronic and Thermal Free Energies = -346.948066

Compound 29a Singlet

Atomic Number	Atomic Type	Coordinates (Angstroms)		
		X	Y	Z
6	C	0.322244	1.435207	0.000214
6	C	1.657176	1.216577	0.000094
6	C	1.896136	-0.236302	-0.000069
6	C	0.594058	-0.880012	0.000038
1	H	-0.183177	2.392339	0.000399
1	H	2.439233	1.964459	0.000139
6	C	-0.361624	0.124893	0.000287
6	C	-2.855955	-0.943675	0.000279
6	C	-3.055104	0.386689	-0.000627
6	C	-1.746377	-0.098107	0.000202
1	H	-3.270515	-1.943098	0.000783
1	H	-3.743335	1.221370	-0.001398
6	C	3.080523	-0.895737	-0.000278
1	H	4.026544	-0.361918	-0.000306
1	H	3.124323	-1.980988	-0.000384
1	H	0.420461	-1.949366	-0.000074

Uncorrected Sum of Electronic and Thermal Free Energies = -346.711966
Corrected Sum of Electronic and Thermal Free Energies = -346.711966

Compound 29a Triplet

Atomic Number	Atomic Type	Coordinates (Angstroms)		
		X	Y	Z
6	C	0.224636	1.367423	-0.000258
6	C	1.640978	1.168185	-0.000100
6	C	1.935461	-0.230831	0.000045
6	C	0.662702	-0.902600	0.000179
1	H	-0.271065	2.331202	-0.000575
1	H	2.378184	1.963042	-0.000048
6	C	-0.402025	0.115781	-0.000072
6	C	-2.892658	-0.946121	-0.000476
6	C	-3.100100	0.378303	0.000760
6	C	-1.778011	-0.091573	-0.000431
1	H	-3.301937	-1.946973	-0.000976
1	H	-3.796093	1.206181	0.001580
6	C	3.213566	-0.786340	0.000134
1	H	4.092801	-0.152532	0.000057
1	H	3.362571	-1.859961	0.000156
1	H	0.508237	-1.974324	0.001117

Sum of Electronic and Thermal Free Energies = -346.665216

Compound 29b Singlet

Atomic Number	Atomic Type	Coordinates (Angstroms)		
		X	Y	Z
6	C	-0.287319	1.411925	-0.003454
6	C	-1.644748	1.192039	0.017565
6	C	-1.899799	-0.249144	0.005430
6	C	-0.616099	-0.875056	-0.026349
1	H	0.215400	2.374295	-0.002060
1	H	-2.423181	1.949671	0.036180
6	C	0.392535	0.127191	-0.030202
6	C	2.911857	-1.023506	-0.102907
6	C	3.045319	0.399414	0.026071
6	C	1.778017	-0.066620	-0.035386
1	H	3.175743	-1.808720	0.620966
1	H	3.701274	1.252150	0.135964
6	C	-3.161572	-0.854277	0.018111
1	H	-4.071174	-0.259493	0.035321
1	H	-3.272198	-1.935394	0.005288
1	H	-0.435011	-1.944303	-0.044934

Uncorrected Sum of Electronic and Thermal Free Energies = -346.951820
Corrected Sum of Electronic and Thermal Free Energies = -346.665216

Compound 29b Triplet

Atomic Number	Atomic Type	Coordinates (Angstroms)		
		X	Y	Z
6	C	0.256642	1.387766	-0.017725
6	C	1.618125	1.203533	-0.014680
6	C	1.904915	-0.232525	0.004154
6	C	0.637008	-0.889590	0.006475
1	H	-0.273286	2.334739	-0.035094
1	H	2.378918	1.978621	-0.025798
6	C	-0.394676	0.088017	-0.005613
6	C	-2.857724	-0.988754	0.068839
6	C	-3.086155	0.448997	-0.090974
6	C	-1.781372	-0.122214	-0.000098
1	H	-3.201952	-1.935096	-0.334760
1	H	-3.615487	1.178112	0.528885
6	C	3.176924	-0.812127	0.016296
1	H	4.075177	-0.200184	0.011710
1	H	3.308067	-1.890961	0.030477
1	H	0.486449	-1.963845	0.024540

Sum of Electronic and Thermal Free Energies = -346.952597

Compound 30a Singlet

Atomic Number	Atomic Type	Coordinates (Angstroms)		
		X	Y	Z
6	C	1.202793	-0.966838	0.000047
6	C	1.229620	0.473780	-0.000041
6	C	0.000040	1.251024	0.000050
6	C	-1.229942	0.473919	-0.000023
6	C	-1.203005	-0.967122	0.000099
6	C	0.000034	-1.681428	0.000314
6	C	2.556603	-1.405546	-0.000480
6	C	3.404347	-0.248761	0.000287
6	C	2.612300	0.886612	-0.000021
1	H	0.000125	-2.768576	0.000163
1	H	2.893468	-2.435630	-0.000556
6	C	-2.612359	0.886677	-0.000009
6	C	-3.404296	-0.248653	0.000109
6	C	-2.556233	-1.405807	-0.000232
1	H	-2.975237	1.906337	0.000037
1	H	-4.486200	-0.270018	0.000224
1	H	-2.893353	-2.435805	-0.000267
1	H	2.975061	1.906322	0.000088
6	C	0.000058	2.617372	0.000086
1	H	-0.922466	3.187929	-0.000683
1	H	0.922584	3.187934	-0.000683
1	H	4.486251	-0.269874	0.000572

Uncorrected Sum of Electronic and Thermal Free Energies = -500.338746
Corrected Sum of Electronic and Thermal Free Energies = -500.332911

Compound 30a Triplet

Atomic Number	Atomic Type	Coordinates (Angstroms)		
		X	Y	Z
6	C	-1.196823	-0.987413	0.000009
6	C	-1.216744	0.495211	0.000006
6	C	-0.000069	1.269703	0.000002
6	C	1.216793	0.495378	0.000003
6	C	1.196901	-0.987274	-0.000004
6	C	0.000167	-1.706781	0.000006
6	C	-2.528937	-1.418625	-0.000024
6	C	-3.369365	-0.261041	0.000017
6	C	-2.572032	0.893246	-0.000010
1	H	0.000237	-2.793724	-0.000005
1	H	-2.870577	-2.446506	-0.000029
6	C	2.571793	0.893519	0.000027
6	C	3.369484	-0.261046	0.000013
6	C	2.529144	-1.418389	-0.000018
1	H	2.947548	1.908184	0.000046
1	H	4.452261	-0.277671	0.000017
1	H	2.870599	-2.446354	-0.000032
1	H	-2.947538	1.907998	-0.000006
6	C	-0.000252	2.656624	0.000004
1	H	0.923178	3.223910	-0.000422
1	H	-0.923913	3.223538	0.000226
1	H	-4.452165	-0.278047	0.000023

Sum of Electronic and Thermal Free Energy = -500.345948

Compound 30b Singlet

Atomic Number	Atomic Type	Coordinates (Angstroms)		
		X	Y	Z
6	C	-1.211629	-0.951484	-0.000109
6	C	-1.241984	0.497677	0.000074
6	C	0.000033	1.291766	0.000193
6	C	1.241990	0.497665	0.000101
6	C	1.211621	-0.951445	-0.000078
6	C	-0.000009	-1.648597	-0.000203
6	C	-2.568779	-1.422682	0.000013
6	C	-3.400556	-0.307268	-0.000110
6	C	-2.585454	0.875332	-0.000066
1	H	0.000028	-2.740498	-0.000202
1	H	-2.875751	-2.463932	0.000045
6	C	2.585503	0.875316	-0.000031
6	C	3.400555	-0.307275	-0.000135
6	C	2.568752	-1.422694	0.000153
1	H	2.968121	1.891300	-0.000074
1	H	4.486357	-0.318142	-0.000248
1	H	2.875648	-2.463963	0.000255
1	H	-2.968124	1.891296	-0.000090
6	C	-0.000016	2.653571	0.000211
1	H	0.925557	3.221420	0.000213
1	H	-0.925647	3.221323	0.000189
1	H	-4.486357	-0.318105	-0.000169

Uncorrected Sum of Electronic and Thermal Free Energies = -500.689740
Corrected Sum of Electronic and Thermal Free Energies = -500.689740

Compound 30b Triplet

Atomic Number	Atomic Type	Coordinates (Angstroms)		
		X	Y	Z
6	C	-1.212163	-0.977247	-0.000032
6	C	-1.214490	0.470258	-0.000002
6	C	0.000006	1.211832	0.000013
6	C	1.214485	0.470244	0.000012
6	C	1.212153	-0.977259	-0.000018
6	C	-0.000014	-1.697857	-0.000044
6	C	-2.575967	-1.399864	-0.000062
6	C	-3.400870	-0.244729	-0.000024
6	C	-2.590030	0.898949	0.000036
1	H	-0.000019	-2.787708	-0.000077
1	H	-2.919707	-2.429812	-0.000102
6	C	2.590050	0.898931	0.000081
6	C	3.400866	-0.244743	0.000012
6	C	2.575940	-1.399885	-0.000039
1	H	2.941587	1.924867	0.000138
1	H	4.486858	-0.247821	0.000012
1	H	2.919681	-2.429832	-0.000083
1	H	-2.941569	1.924885	0.000078
6	C	0.000027	2.639918	0.000055
1	H	0.928233	3.200948	0.000078
1	H	-0.928164	3.200976	0.000067
1	H	-4.486861	-0.247784	-0.000032

Sum of Electronic and Thermal Free Energies = -500.654682

Compound 31a Singlet

Atomic Number	Atomic Type	Coordinates (Angstroms)		
		X	Y	Z
6	C	-0.816043	-0.740499	0.000020
6	C	-0.816043	0.740499	-0.000022
6	C	0.411562	1.466600	-0.000032
6	C	1.581210	0.739714	-0.000011
6	C	1.581210	-0.739714	0.000010
6	C	0.411562	-1.466600	0.000031
6	C	-2.120586	-1.174419	0.000004
6	C	-2.996380	0.000000	0.000001
6	C	-2.120586	1.174419	-0.000005
1	H	0.414543	2.553554	-0.000015
1	H	0.414543	-2.553554	0.000014
1	H	-2.465120	-2.201889	0.000004
6	C	2.945906	1.146618	0.000022
6	C	3.759176	0.000000	0.000001
6	C	2.945906	-1.146618	-0.000021
1	H	3.297981	2.171597	0.000038
1	H	4.841586	0.000000	0.000002
1	H	3.297981	-2.171597	-0.000037
6	C	-4.350878	0.000000	0.000003
1	H	-2.465120	2.201889	-0.000005
1	H	-4.916241	0.926932	0.000287
1	H	-4.916241	-0.926932	-0.000291

Uncorrected Sum of Electronic and Thermal Free Energies = -500.382558
Corrected Sum of Electronic and Thermal Free Energies = -500.382558

Compound 31a Triplet

Atomic Number	Atomic Type	Coordinates (Angstroms)		
		X	Y	Z
6	C	-0.805970	-0.730271	0.000013
6	C	-0.805970	0.730271	-0.000013
6	C	0.404015	1.460180	-0.000006
6	C	1.582402	0.726859	0.000000
6	C	1.582402	-0.726859	-0.000001
6	C	0.404015	-1.460180	0.000006
6	C	-2.145275	-1.168171	0.000024
6	C	-3.016520	0.000000	0.000002
6	C	-2.145275	1.168171	-0.000023
1	H	0.405871	2.545539	0.000005
1	H	0.405871	-2.545539	-0.000005
1	H	-2.483973	-2.197591	0.000041
6	C	2.978015	1.148319	0.000021
6	C	3.784126	0.000000	0.000000
6	C	2.978015	-1.148319	-0.000021
1	H	3.320425	2.175315	0.000038
1	H	4.866702	0.000000	0.000001
1	H	3.320425	-2.175315	-0.000038
6	C	-4.373186	0.000000	0.000004
1	H	-2.483973	2.197591	-0.000041
1	H	-4.938055	0.927222	0.000115
1	H	-4.938055	-0.927222	-0.000141

Sum of Electronic and Thermal Free Energies = -500.350140

Compound 31b Singlet

Atomic Number	Atomic Type	Coordinates (Angstroms)		
		X	Y	Z
6	C	-0.814723	0.732619	0.000008
6	C	-0.816496	-0.748930	-0.000008
6	C	0.396783	-1.451371	-0.000016
6	C	1.613181	-0.741311	-0.000008
6	C	1.614003	0.733704	0.000008
6	C	0.417649	1.446272	0.000015
6	C	-2.136055	1.165108	0.000013
6	C	-3.019214	-0.011361	0.000000
6	C	-2.166009	-1.168578	-0.000012
1	H	0.398236	-2.541475	-0.000027
1	H	0.415935	2.536141	0.000027
1	H	-2.483556	2.193288	0.000024
6	C	2.967669	-1.156451	-0.000013
6	C	3.786630	0.005279	-0.000001
6	C	2.985595	1.156450	0.000012
1	H	3.319152	-2.183160	-0.000024
1	H	4.873419	-0.000448	-0.000001
1	H	3.331954	2.184550	0.000023
6	C	-4.397609	0.025772	0.000001
1	H	-2.517615	-2.195155	-0.000023
1	H	-4.989073	-0.886035	-0.000009
1	H	-4.936882	0.969088	0.000011

Uncorrected Sum of Electronic and Thermal Free Energies = -500.668302
Corrected Sum of Electronic and Thermal Free Energies = -500.664036

Compound 31b Triplet

Atomic Number	Atomic Type	Coordinates (Angstroms)		
		X	Y	Z
6	C	-0.809625	0.735178	0.000008
6	C	-0.809625	-0.735178	-0.000008
6	C	0.405634	-1.442266	-0.000015
6	C	1.618339	-0.729720	-0.000008
6	C	1.618339	0.729720	0.000007
6	C	0.405634	1.442266	0.000015
6	C	-2.157720	1.159245	0.000013
6	C	-3.020680	0.000000	0.000001
6	C	-2.157720	-1.159245	-0.000012
1	H	0.404994	-2.532802	-0.000027
1	H	0.404994	2.532802	0.000027
1	H	-2.507237	2.187334	0.000025
6	C	2.980944	-1.151819	-0.000014
6	C	3.797838	0.000000	-0.000001
6	C	2.980944	1.151819	0.000013
1	H	3.326234	-2.181109	-0.000026
1	H	4.883900	0.000000	-0.000002
1	H	3.326234	2.181109	0.000025
6	C	-4.414448	0.000000	0.000001
1	H	-2.507237	-2.187334	-0.000024
1	H	-4.979500	-0.928105	-0.000010
1	H	-4.979500	0.928105	0.000011

Sum of Electronic and Thermal Free Energies = -500.674573

Compound 32a Singlet

Atomic Number	Atomic Type	Coordinates (Angstroms)		
		X	Y	Z
6	C	-2.301557	0.962281	-0.000255
6	C	-0.926109	1.207783	-0.000154
6	C	-0.402395	2.534690	-0.000191
6	C	-2.833086	-0.371202	-0.000217
6	C	-0.025821	0.085915	-0.000008
6	C	-0.546847	-1.251684	0.000031
6	C	-1.928604	-1.463995	-0.000073
6	C	0.411701	-2.312162	0.000178
6	C	1.747548	-2.035469	0.000274
6	C	1.373596	0.323952	0.000096
6	C	1.854635	1.655279	0.000055
6	C	0.967769	2.737908	-0.000088
7	N	2.241406	-0.757304	0.000237
1	H	2.491003	-2.822729	0.000386
1	H	0.087920	-3.346336	0.000215
1	H	-2.315881	-2.477714	-0.000042
1	H	-2.991162	1.801827	-0.000365
1	H	-1.087328	3.376434	-0.000301
1	H	1.368369	3.745983	-0.000116
6	C	-4.221886	-0.541424	-0.000324
1	H	2.918894	1.851997	0.000133
1	H	-4.667397	-1.530734	-0.000299
1	H	-4.892229	0.311418	-0.000433
6	C	3.697491	-0.547458	0.000349
1	H	4.004833	0.001431	-0.893776
1	H	4.004680	0.001542	0.894458
1	H	4.189848	-1.518481	0.000451

Uncorrected Sum of Electronic and Thermal Free Energies = -595.066345
Corrected Sum of Electronic and Thermal Free Energies = -595.065145

Compound 32a Triplet

Atomic Number	Atomic Type	Coordinates (Angstroms)		
		X	Y	Z
6	C	-2.307135	0.985954	-0.000257
6	C	-0.914772	1.221341	-0.000155
6	C	-0.366821	2.527883	-0.000190
6	C	-2.857378	-0.331391	-0.000220
6	C	-0.019466	0.102987	-0.000009
6	C	-0.549915	-1.224040	0.000030
6	C	-1.946567	-1.424306	-0.000075
6	C	0.374492	-2.299106	0.000175
6	C	1.726324	-2.044028	0.000274
6	C	1.384659	0.322564	0.000096
6	C	1.896412	1.641461	0.000056
6	C	1.014547	2.720033	-0.000086
7	N	2.229273	-0.781193	0.000237
1	H	2.456092	-2.843945	0.000386
1	H	0.033985	-3.328313	0.000211
1	H	-2.332418	-2.439588	-0.000042
1	H	-2.981534	1.837131	-0.000367
1	H	-1.034118	3.383910	-0.000300
1	H	1.415793	3.728079	-0.000116
6	C	-4.245279	-0.556255	-0.000321
1	H	2.962180	1.826925	0.000135
1	H	-4.648190	-1.562703	-0.000290
1	H	-4.947439	0.269837	-0.000431
6	C	3.693868	-0.597246	0.000351
1	H	4.001786	-0.051009	-0.893846
1	H	4.001634	-0.050896	0.894532
1	H	4.169502	-1.576188	0.000453

Sum of Electronic and Thermal Free Energies = -595.069888

Compound 32b Singlet

Atomic Number	Atomic Type	Coordinates (Angstroms)		
		X	Y	Z
6	C	-2.308055	0.988553	-0.000256
6	C	-0.933306	1.224637	-0.000156
6	C	-0.356759	2.546456	-0.000189
6	C	-2.895255	-0.327869	-0.000223
6	C	-0.006561	0.112322	-0.000009
6	C	-0.568825	-1.220275	0.000028
6	C	-1.931113	-1.414882	-0.000073
6	C	0.389983	-2.313530	0.000177
6	C	1.720556	-2.068367	0.000274
6	C	1.391720	0.336151	0.000095
6	C	1.916377	1.640402	0.000058
6	C	1.007357	2.728419	-0.000086
7	N	2.251027	-0.787218	0.000238
1	H	2.454717	-2.868197	0.000385
1	H	0.041084	-3.342312	0.000212
1	H	-2.311963	-2.436754	-0.000040
1	H	-2.977738	1.848454	-0.000366
1	H	-1.025036	3.405461	-0.000299
1	H	1.411186	3.740492	-0.000116
6	C	-4.261799	-0.572378	-0.000323
1	H	2.982930	1.827946	0.000134
1	H	-4.651014	-1.587057	-0.000290
1	H	-4.981297	0.242183	-0.000436
6	C	3.684212	-0.601245	0.000350
1	H	4.018529	-0.045552	-0.887657
1	H	4.018376	-0.045444	0.888348
1	H	4.171845	-1.579057	0.000452

Uncorrected Sum of Electronic and Thermal Free Energies = -595.334589
Corrected Sum of Electronic and Thermal Free Energies = -595.334589

Compound 32b Triplet

Atomic Number	Atomic Type	Coordinates (Angstroms)		
		X	Y	Z
6	C	-2.323370	0.968094	0.004383
6	C	-0.921614	1.207415	0.004713
6	C	-0.391339	2.532651	-0.014246
6	C	-2.848495	-0.368260	0.003361
6	C	-0.019135	0.097001	0.025309
6	C	-0.537155	-1.271794	0.015431
6	C	-1.951041	-1.455352	0.003193
6	C	0.410641	-2.313698	-0.048722
6	C	1.775221	-2.075781	-0.059793
6	C	1.389906	0.335397	0.047134
6	C	1.873280	1.654687	-0.011419
6	C	0.972897	2.740385	-0.030809
7	N	2.265749	-0.753075	0.157067
1	H	2.518299	-2.857794	0.028775
1	H	0.068798	-3.344421	-0.118740
1	H	-2.348924	-2.469253	-0.009909
1	H	-3.008275	1.812729	-0.005702
1	H	-1.081784	3.373510	-0.025127
1	H	1.370938	3.753106	-0.063147
6	C	-4.256662	-0.537501	-0.002318
1	H	2.937512	1.855140	-0.031208
1	H	-4.706961	-1.526566	-0.005791
1	H	-4.921665	0.321148	-0.006374
6	C	3.679459	-0.540294	-0.037246
1	H	3.923305	-0.206149	-1.066212
1	H	4.069104	0.204054	0.666430
1	H	4.203852	-1.481674	0.143719

Sum of Electronic and Thermal Free Energies = -595.286903

Compound 33a Singlet

Atomic Number	Atomic Type	Coordinates (Angstroms)		
		X	Y	Z
6	C	2.869761	0.338823	-0.000284
6	C	1.794161	-0.584587	-0.000186
6	C	2.014704	-1.990252	-0.000225
6	C	2.628674	1.698735	-0.000245
6	C	0.454512	-0.084369	-0.000046
6	C	0.222423	1.321910	-0.000006
6	C	1.309745	2.188058	-0.000106
6	C	-1.179311	1.781113	0.000143
6	C	-2.193479	0.761317	0.000228
6	C	-0.613891	-1.031280	0.000049
6	C	-0.370312	-2.395658	0.000007
6	C	0.957271	-2.872825	-0.000130
1	H	1.133121	-3.942839	-0.000160
1	H	-1.179461	-3.114584	0.000078
1	H	3.456700	2.399609	-0.000321
1	H	1.153539	3.261434	-0.000079
7	N	-1.949234	-0.526455	0.000186
1	H	3.887385	-0.039518	-0.000390
1	H	3.035699	-2.359428	-0.000331
1	H	-3.237794	1.055853	0.000334
6	C	-1.624203	3.067867	0.000211
1	H	-2.685376	3.293268	0.000322
1	H	-0.951659	3.917214	0.000162
6	C	-3.083633	-1.477312	0.000284
1	H	-3.035684	-2.100581	-0.894134
1	H	-3.035515	-2.100599	0.894679
1	H	-4.014846	-0.913875	0.000377

Uncorrected Sum of Electronic and Thermal Free Energies = -595.089183

Compound 33a Triplet

Atomic Number	Atomic Type	Coordinates (Angstroms)		
		X	Y	Z
6	C	2.866705	0.107740	-0.000280
6	C	1.727864	-0.734623	-0.000187
6	C	1.847247	-2.143218	-0.000231
6	C	2.731425	1.498214	-0.000234
6	C	0.427055	-0.132932	-0.000044
6	C	0.296668	1.284282	0.000001
6	C	1.472591	2.084150	-0.000096
6	C	-1.032786	1.850743	0.000143
6	C	-2.108774	0.965571	0.000230
6	C	-0.723260	-0.979725	0.000050
6	C	-0.564982	-2.395097	0.000003
6	C	0.703913	-2.952250	-0.000135
1	H	0.808941	-4.032050	-0.000170
1	H	-1.429785	-3.044950	0.000075
1	H	3.616705	2.125417	-0.000307
1	H	1.392300	3.163389	-0.000064
7	N	-1.966433	-0.399191	0.000186
1	H	3.854649	-0.342607	-0.000389
1	H	2.834456	-2.593909	-0.000339
1	H	-3.128143	1.327866	0.000337
6	C	-1.279573	3.256403	0.000194
1	H	-2.297218	3.628998	0.000298
1	H	-0.484589	3.987657	0.000133
6	C	-3.184539	-1.231368	0.000286
1	H	-3.212988	-1.857958	-0.894156
1	H	-3.212813	-1.857999	0.894704
1	H	-4.053799	-0.576860	0.000386

Sum of Electronic and Thermal Free Energies = -595.056188

Compound 33b Singlet

Atomic Number	Atomic Type	Coordinates (Angstroms)		
		X	Y	Z
6	C	2.825843	0.512549	-0.000276
6	C	1.820704	-0.492177	-0.000186
6	C	2.140142	-1.876906	-0.000230
6	C	2.482073	1.855043	-0.000229
6	C	0.438835	-0.072271	-0.000045
6	C	0.101872	1.312610	0.000000
6	C	1.140356	2.260730	-0.000093
6	C	-1.320947	1.735096	0.000143
6	C	-2.242443	0.674260	0.000230
6	C	-0.585123	-1.095114	0.000048
6	C	-0.223311	-2.466398	-0.000002
6	C	1.126848	-2.831190	-0.000139
1	H	1.382941	-3.889583	-0.000176
1	H	-0.982307	-3.239973	0.000065
1	H	3.266783	2.610607	-0.000300
1	H	0.899885	3.316079	-0.000058
7	N	-1.885331	-0.677431	0.000184
1	H	3.870651	0.207498	-0.000382
1	H	3.185590	-2.176738	-0.000337
1	H	-3.309628	0.857784	0.000338
6	C	-1.705904	3.078990	0.000190
1	H	-2.760267	3.343544	0.000296
1	H	-0.997075	3.894885	0.000124
6	C	-2.944870	-1.664163	0.000289
1	H	-2.887933	-2.307959	-0.888894
1	H	-2.887741	-2.307974	0.889449
1	H	-3.908039	-1.152505	0.000398

Uncorrected Sum of Electronic and Thermal Free Energies = -595.325676
Corrected Sum of Electronic and Thermal Free Energies = -595.325676

Compound 33b Triplet

Atomic Number	Atomic Type	Coordinates (Angstroms)		
		X	Y	Z
6	C	2.872847	0.344668	-0.000328
6	C	1.800093	-0.586768	0.011885
6	C	2.024400	-1.993735	0.050060
6	C	2.603181	1.731203	-0.036127
6	C	0.441545	-0.103574	-0.018304
6	C	0.181544	1.328728	-0.035061
6	C	1.302691	2.210485	-0.049700
6	C	-1.188496	1.780420	0.027574
6	C	-2.215029	0.786985	0.050854
6	C	-0.617492	-1.050086	-0.040188
6	C	-0.360088	-2.431756	0.007719
6	C	0.966830	-2.890241	0.053370
1	H	1.157765	-3.961192	0.092589
1	H	-1.169348	-3.151826	0.016223
1	H	3.431996	2.438035	-0.051605
1	H	1.135632	3.283610	-0.072328
7	N	-1.947831	-0.569398	-0.158547
1	H	3.895604	-0.024812	0.019475
1	H	3.050875	-2.354974	0.077461
1	H	-3.260485	1.067349	0.001803
6	C	-1.590152	3.117561	0.090939
1	H	-2.645567	3.371185	0.137897
1	H	-0.884240	3.938759	0.114741
6	C	-3.054623	-1.485552	-0.001154
1	H	-2.963250	-2.331858	-0.688775
1	H	-3.135278	-1.880372	1.030048
1	H	-3.982402	-0.958149	-0.236928

Sum of Electronic and Thermal Free Energies = -595.296120

Compound 34a Singlet

Atomic Number	Atomic Type	Coordinates (Angstroms)		
		X	Y	Z
6	C	0.080289	0.727812	-0.000275
6	C	1.169969	-0.305405	-0.000269
6	C	0.090881	-1.296623	-0.000065
7	N	-0.864513	-0.391909	-0.000122
1	H	-0.015497	-2.376518	0.000016
6	C	-2.318389	-0.458024	0.000176
1	H	-2.628032	-1.503483	0.001089
1	H	-2.699286	0.045605	0.892718
1	H	-2.699553	0.044083	-0.893118
6	C	-0.130946	2.035611	0.000074
1	H	-1.131169	2.456124	0.000212
1	H	0.707216	2.723824	0.000038
6	C	2.505363	-0.352962	0.000206
1	H	3.102589	0.553286	0.000345
1	H	3.032328	-1.302007	0.000463

Uncorrected Sum of Electronic and Thermal Free Energies = -287.797830
Corrected Sum of Electronic and Thermal Free Energies = -287.797830

Compound 34a Triplet

Atomic Number	Atomic Type	Coordinates (Angstroms)		
		X	Y	Z
6	C	0.025669	0.631217	0.009203
6	C	1.176284	-0.347900	-0.014039
6	C	0.174695	-1.396830	-0.028458
7	N	-0.885003	-0.349846	0.008800
1	H	0.061849	-2.431897	0.263759
6	C	-2.329758	-0.425536	-0.015976
1	H	-2.645928	-0.957483	-0.920059
1	H	-2.678521	-0.981466	0.860775
1	H	-2.756319	0.577673	-0.008136
6	C	-0.147269	2.030238	0.015301
1	H	-1.134306	2.479989	0.005291
1	H	0.720596	2.679676	0.015291
6	C	2.513486	-0.243068	-0.009364
1	H	3.008480	0.721966	0.007060
1	H	3.140525	-1.128258	-0.025592

Sum of Electronic and Thermal Free Energies = -287.719327

Compound 34b Singlet

Atomic Number	Atomic Type	Coordinates (Angstroms)		
		X	Y	Z
6	C	-0.013381	0.626193	0.142199
6	C	-1.113234	-0.374812	-0.053656
6	C	-0.116102	-1.398007	-0.012044
7	N	0.934357	-0.398281	0.433357
1	H	-0.186157	-2.344419	0.527180
6	C	2.203675	-0.366105	-0.278436
1	H	2.779493	-1.264256	-0.026867
1	H	2.062904	-0.344923	-1.378351
1	H	2.793934	0.508831	0.027722
6	C	0.132735	1.958975	0.031068
1	H	1.090522	2.446980	0.194365
1	H	-0.721023	2.584415	-0.210254
6	C	-2.475876	-0.203154	-0.128235
1	H	-2.912544	0.791368	-0.158382
1	H	-3.154536	-1.048563	-0.214286

Uncorrected Sum of Electronic and Thermal Free Energies = -288.034865

Compound 34b Triplet

Atomic Number	Atomic Type	Coordinates (Angstroms)		
		X	Y	Z
6	C	-0.065465	0.682042	-0.071065
6	C	-1.097956	-0.313174	0.012939
6	C	-0.083883	-1.342666	-0.275635
7	N	0.928930	-0.347716	-0.390293
1	H	0.106545	-2.319999	0.176682
6	C	2.193515	-0.452258	0.306411
1	H	2.726584	-1.348868	-0.037553
1	H	2.817441	0.420169	0.083119
1	H	2.070947	-0.512902	1.406111
6	C	0.115105	2.056076	-0.046095
1	H	1.088857	2.501432	-0.225910
1	H	-0.713445	2.718361	0.181389
6	C	-2.481643	-0.346067	0.179398
1	H	-3.051447	0.557436	0.376341
1	H	-3.026028	-1.285334	0.136153

Sum of Electronic and Thermal Free Energies = -287.996985

Compound 35a Singlet

Atomic Number	Atomic Type	Coordinates (Angstroms)		
		X	Y	Z
6	C	0.399393	0.237835	-0.000011
6	C	0.068311	1.557669	-0.000005
6	C	-1.359046	1.166680	0.000038
7	N	-1.142882	-0.117979	-0.000012
1	H	0.597428	2.496520	-0.000018
1	H	-2.319796	1.677593	0.000075
6	C	-1.946349	-1.321900	-0.000009
1	H	-3.000781	-1.036541	0.000020
1	H	-1.729370	-1.915742	-0.893432
1	H	-1.729327	-1.915764	0.893391
6	C	1.463987	-0.701723	-0.000026
6	C	2.767094	-0.331534	0.000018
1	H	3.553833	-1.078513	0.000008
1	H	3.073599	0.710541	0.000081
1	H	1.194243	-1.754400	-0.000073

Uncorrected Sum of Electronic and Thermal Free Energies = -287.753764
Corrected Sum of Electronic and Thermal Free Energies = -287.753764

Compound 35a Triplet

Atomic Number	Atomic Type	Coordinates (Angstroms)		
		X	Y	Z
6	C	-0.335117	0.160231	0.000040
6	C	0.025912	1.573882	-0.000002
6	C	1.401912	1.189289	-0.000029
7	N	1.066268	-0.139812	-0.000002
1	H	-0.509139	2.512738	-0.000034
1	H	2.383446	1.641813	-0.000003
6	C	1.829803	-1.372599	0.000019
1	H	2.892578	-1.130198	0.000189
1	H	1.580742	-1.956059	0.892419
1	H	1.580992	-1.955913	-0.892542
6	C	-1.444566	-0.686460	-0.000039
6	C	-2.742424	-0.239703	0.000018
1	H	-3.567444	-0.942471	-0.000046
1	H	-2.989154	0.817533	0.000129
1	H	-1.249016	-1.756596	-0.000139

Sum of Electronic and Thermal Free Energies = -287.743208

Compound 35b Singlet

C	0.286323	0.228352	0.128047
C	-0.253442	1.517187	-0.181996
C	-1.549442	1.049136	0.025761
N	-1.076463	-0.257626	0.443182
H	0.180612	2.499001	-0.339984
H	-2.544597	1.449751	0.182085
C	-1.572217	-1.417184	-0.285985
H	-2.641397	-1.555931	-0.070894
H	-1.442574	-1.323855	-1.381233
H	-1.049414	-2.322905	0.049856
C	1.478972	-0.495667	0.169703
C	2.784847	-0.124147	-0.118240
H	3.597451	-0.837635	-0.018338
H	3.042629	0.881488	-0.442856
H	1.342289	-1.532586	0.495352

Uncorrected Sum of Electronic and Thermal Free Energies = -288.036432
Corrected Sum of Electronic and Thermal Free Energies = -288.036432

Compound 35b Triplet

Atomic Number	Atomic Type	Coordinates (Angstroms)		
		X	Y	Z
6	C	-0.280937	0.163459	-0.120637
6	C	0.113963	1.492166	0.217293
6	C	1.487642	1.127542	-0.020705
7	N	1.088449	-0.237445	-0.444471
1	H	-0.421429	2.424576	0.337455
1	H	2.299135	1.656927	-0.527736
6	C	1.653341	-1.350161	0.317476
1	H	2.716282	-1.458300	0.066339
1	H	1.568843	-1.204129	1.411315
1	H	1.140979	-2.282888	0.048994
6	C	-1.438428	-0.617812	-0.216042
6	C	-2.724409	-0.136714	0.120616
1	H	-3.603849	-0.770214	0.046216
1	H	-2.858602	0.855686	0.541943
1	H	-1.327538	-1.630422	-0.601229

Sum of Electronic and Thermal Free Energies = -287.984075

Compound 36a Singlet

Atomic Number	Atomic Type	Coordinates (Angstroms)		
		X	Y	Z
6	C	1.273969	-1.324593	-0.000316
6	C	1.659818	0.080317	-0.000345
6	C	0.581856	1.034225	-0.000408
6	C	-1.102803	-0.676713	0.000145
6	C	-0.029443	-1.661857	0.000180
1	H	0.807176	2.096613	-0.000655
7	N	-0.685762	0.703074	-0.000091
6	C	-1.718265	1.763238	0.000164
1	H	-2.335520	1.665204	0.894821
1	H	-1.227742	2.734999	0.000202
1	H	-2.335767	1.665490	-0.894356
6	C	-2.410734	-1.005401	0.000024
1	H	-2.686036	-2.053410	0.000012
1	H	-3.221835	-0.290405	-0.000064
1	H	-0.336006	-2.702024	0.000573
6	C	2.936788	0.550385	0.000420
1	H	3.784571	-0.126867	0.000966
1	H	3.154477	1.613714	0.000517
1	H	2.049902	-2.082435	-0.000566

Uncorrected Sum of Electronic and Thermal Free Energies = -365.236183
Corrected Sum of Electronic and Thermal Free Energies = -365.236183

Compound 36a Triplet

Atomic Number	Atomic Type	Coordinates (Angstroms)		
		X	Y	Z
6	C	1.261249	-1.274216	-0.000099
6	C	1.649289	0.091472	-0.000024
6	C	0.615217	1.038954	-0.000052
6	C	-1.093857	-0.635323	0.000017
6	C	-0.076368	-1.620405	-0.000043
1	H	0.819752	2.101654	-0.000144
7	N	-0.703977	0.685431	-0.000038
6	C	-1.722606	1.757332	-0.000054
1	H	-2.343137	1.679765	0.895264
1	H	-1.217375	2.720932	-0.000239
1	H	-2.343416	1.679477	-0.895132
6	C	-2.461631	-1.015519	0.000101
1	H	-2.706363	-2.070189	0.000023
1	H	-3.278458	-0.307595	0.000453
1	H	-0.376828	-2.662164	-0.000028
6	C	3.031518	0.471279	0.000141
1	H	3.805934	-0.285898	0.000152
1	H	3.328820	1.513298	0.000227
1	H	2.022042	-2.048743	-0.000233

Sum of Electronic and Thermal Free Energies = -365.198253

Compound 36b Singlet

Atomic Number	Atomic Type	Coordinates (Angstroms)		
		X	Y	Z
6	C	-1.233495	1.249073	0.000039
6	C	-1.700366	-0.135514	0.000031
6	C	-0.647383	-1.065013	-0.000037
6	C	1.152520	0.620851	-0.000020
6	C	0.077172	1.597483	0.000014
1	H	-0.810782	-2.135274	-0.000038
7	N	0.718530	-0.683405	0.000000
6	C	1.718287	-1.721514	-0.000034
1	H	2.365195	-1.643343	0.887951
1	H	1.231205	-2.698373	0.000052
1	H	2.365062	-1.643433	-0.888127
6	C	2.492744	0.999965	-0.000070
1	H	2.734947	2.056515	-0.000055
1	H	3.316946	0.299478	-0.000121
1	H	0.370745	2.644481	0.000015
6	C	-3.082947	-0.400351	0.000103
1	H	-3.797237	0.416927	0.000025
1	H	-3.473075	-1.414257	0.000071
1	H	-1.991913	2.031239	0.000072

Uncorrected Sum of Electronic and Thermal Free Energies = -365.468524
Corrected Sum of Electronic and Thermal Free Energies = -365.468524

Compound 36b Triplet

Atomic Number	Atomic Type	Coordinates (Angstroms)		
		X	Y	Z
6	C	-1.264713	-1.313706	-0.021919
6	C	-1.685936	0.045020	0.002956
6	C	-0.642690	1.029871	0.026752
6	C	1.142583	-0.661183	-0.019759
6	C	0.111811	-1.635213	0.012763
1	H	-0.870277	2.086718	-0.060862
7	N	0.707750	0.695615	-0.134628
6	C	1.657342	1.771093	0.037463
1	H	2.549544	1.597920	-0.569729
1	H	1.196022	2.706094	-0.296070
1	H	1.967556	1.897999	1.092692
6	C	2.505029	-0.953818	0.020476
1	H	2.810111	-1.992456	0.090423
1	H	3.286835	-0.208162	0.052335
1	H	0.427828	-2.674815	0.043134
6	C	-3.022345	0.473411	0.026350
1	H	-3.840520	-0.241383	0.030335
1	H	-3.275706	1.529722	0.052454
1	H	-2.01213	-2.103792	-0.002808

Sum of Electronic and Thermal Free Energies = -365.444262

Compound 37a Singlet

Atomic Number	Atomic Type	Coordinates (Angstroms)		
		X	Y	Z
6	C	-0.000164	-2.122641	-0.071882
6	C	1.190913	-1.410957	-0.031182
6	C	1.213198	0.009857	0.021544
6	C	-1.213213	0.010044	0.021523
6	C	-1.191134	-1.410777	-0.031257
7	N	0.000038	0.692099	-0.048880
6	C	0.000213	2.155868	-0.181812
1	H	0.000932	2.647982	0.796389
1	H	0.878226	2.465296	-0.747829
1	H	-0.878402	2.465634	-0.746683
6	C	-2.441935	0.645078	0.160505
1	H	-3.343892	0.046103	0.147496
1	H	-2.557120	1.710739	0.303188
1	H	-2.144229	-1.928553	-0.019583
1	H	-0.000247	-3.206348	-0.109308
6	C	2.442008	0.644741	0.160464
1	H	3.343874	0.045623	0.147702
1	H	2.557347	1.710430	0.302797
1	H	2.143929	-1.928876	-0.019429

Uncorrected Sum of Electronic and Thermal Free Energies = -365.210661
Corrected Sum of Electronic and Thermal Free Energies = -365.213918

Compound 37a Triplet

Atomic Number	Atomic Type	Coordinates (Angstroms)		
		X	Y	Z
6	C	-0.000694	-2.129800	-0.048516
6	C	1.196977	-1.429408	-0.019251
6	C	1.210330	-0.006603	0.010161
6	C	-1.210395	-0.005811	0.010054
6	C	-1.197913	-1.428633	-0.019365
7	N	0.000199	0.675479	-0.032424
6	C	0.000898	2.154151	-0.122476
1	H	0.001843	2.595680	0.877759
1	H	0.875798	2.481415	-0.676695
1	H	-0.874362	2.482424	-0.675476
6	C	-2.434569	0.682692	0.106362
1	H	-3.347242	0.099972	0.132757
1	H	-2.530644	1.754643	0.195316
1	H	-2.151444	-1.942581	-0.008705
1	H	-0.001046	-3.214323	-0.077047
6	C	2.434845	0.681265	0.106295
1	H	3.347258	0.098116	0.132223
1	H	2.531392	1.753133	0.195804
1	H	2.150184	-1.943956	-0.00855

Sum of Electronic and Thermal Free Energies = -365.204612

Compound 37b Singlet

Atomic Number	Atomic Type	Coordinates (Angstroms)		
		X	Y	Z
6	C	-0.000082	2.104176	0.064827
6	C	1.217737	1.434250	0.029729
6	C	1.254461	0.002453	-0.026218
6	C	-1.254473	0.002356	-0.026203
6	C	-1.217851	1.434153	0.029772
7	N	0.000022	-0.655066	0.061288
6	C	0.000109	-2.097172	0.170953
1	H	0.000287	-2.599413	-0.810775
1	H	0.887670	-2.414994	0.723496
1	H	-0.887536	-2.415126	0.723268
6	C	-2.438520	-0.710854	-0.140618
1	H	-3.368784	-0.153748	-0.178225
1	H	-2.501170	-1.781249	-0.275366
1	H	-2.161385	1.968704	-0.009471
1	H	-0.000126	3.194957	0.091234
6	C	2.438553	-0.710684	-0.140602
1	H	3.368784	-0.153524	-0.178221
1	H	2.501268	-1.781085	-0.275270
1	H	2.161232	1.968869	-0.009527

Uncorrected Sum of Electronic and Thermal Free Energies = -365.496510
Corrected Sum of Electronic and Thermal Free Energies = -365.496510

Compound 37b Triplet

Atomic Number	Atomic Type	Coordinates (Angstroms)		
		X	Y	Z
6	C	-0.000079	2.146590	0.253547
6	C	1.200765	1.417877	0.009748
6	C	1.225621	0.007779	-0.057990
6	C	-1.225627	0.007690	-0.057984
6	C	-1.200871	1.417789	0.009760
7	N	0.000024	-0.658520	0.260128
6	C	0.000094	-2.099433	0.349794
1	H	0.000108	-2.611308	-0.635928
1	H	0.885625	-2.425636	0.899867
1	H	-0.885386	-2.425726	0.899892
6	C	-2.393049	-0.710557	-0.335221
1	H	-3.312681	-0.163628	-0.511738
1	H	-2.422798	-1.783857	-0.463696
1	H	-2.146755	1.936054	-0.144524
1	H	-0.000118	3.230622	0.236583
6	C	2.393086	-0.710397	-0.335221
1	H	3.312685	-0.163414	-0.511742
1	H	2.422905	-1.783701	-0.463660
1	H	2.146611	1.936209	-0.144544

Sum of Electronic and Thermal Free Energies = -365.427190

Compound 38a Singlet

Atomic Number	Atomic Type	Coordinates (Angstroms)		
		X	Y	Z
6	C	0.658504	0.542366	-0.000042
6	C	-0.261273	-0.536544	0.000033
6	C	-2.059137	0.977889	-0.000198
6	C	-1.195659	2.055406	-0.000278
6	C	0.196015	1.845823	-0.000206
6	C	2.004801	-0.063799	0.000156
6	C	1.785650	-1.512675	0.000100
6	C	0.452105	-1.797349	0.000198
1	H	-3.135366	1.095083	-0.000248
1	H	-1.610342	3.056377	-0.000387
1	H	0.874412	2.693248	-0.000242
7	N	-1.596396	-0.306599	-0.000045
1	H	0.006287	-2.782455	0.000283
6	C	3.193598	0.568804	0.000233
1	H	2.586695	-2.241745	0.000144
1	H	4.124577	0.010524	0.000275
1	H	3.271787	1.651670	0.000137
6	C	-2.543272	-1.443571	0.000034
1	H	-2.385208	-2.049042	-0.894653
1	H	-2.385287	-2.048851	0.894865
1	H	-3.560770	-1.056727	-0.000052

Uncorrected Sum of Electronic and Thermal Free Energies = -441.474182
Corrected Sum of Electronic and Thermal Free Energies = -441.474182

Compound 38a Triplet

Atomic Number	Atomic Type	Coordinates (Angstroms)		
		X	Y	Z
6	C	0.683121	0.492043	-0.000012
6	C	-0.299473	-0.563354	0.000039
6	C	-2.040051	1.018113	-0.000195
6	C	-1.115779	2.070714	-0.000244
6	C	0.264864	1.812449	-0.000152
6	C	2.028943	-0.136342	0.000111
6	C	1.807735	-1.513435	0.000208
6	C	0.347802	-1.793949	0.000191
1	H	-3.108297	1.198097	-0.000263
1	H	-1.495394	3.085816	-0.000355
1	H	0.972497	2.635228	-0.000194
7	N	-1.649023	-0.266873	-0.000057
1	H	-0.096498	-2.780703	0.000260
6	C	3.261538	0.531765	0.000119
1	H	2.568697	-2.282468	0.000303
1	H	4.188349	-0.029232	0.000211
1	H	3.328580	1.613203	0.000035
6	C	-2.635817	-1.364405	-0.000003
1	H	-2.496342	-1.975707	-0.894450
1	H	-2.496424	-1.975546	0.894567
1	H	-3.639296	-0.942170	-0.000087

Sum of Electronic and Thermal Free Energies = -441.399338

Compound 38b Singlet

Atomic Number	Atomic Type	Coordinates (Angstroms)		
		X	Y	Z
6	C	0.693230	0.500489	0.012863
6	C	-0.282006	-0.584276	0.027253
6	C	-2.071707	1.022822	0.005836
6	C	-1.152754	2.074958	-0.011875
6	C	0.234922	1.827779	-0.006313
6	C	2.015730	-0.092097	-0.000690
6	C	1.783561	-1.518237	-0.001979
6	C	0.391066	-1.813149	0.012675
1	H	-3.145754	1.161866	0.027345
1	H	-1.537380	3.091184	-0.028623
1	H	0.935774	2.658517	-0.027447
7	N	-1.631636	-0.300399	0.055438
1	H	-0.050811	-2.802824	-0.000328
6	C	3.252917	0.560887	-0.013577
1	H	2.574824	-2.262117	-0.018260
1	H	4.181750	-0.002785	-0.027539
1	H	3.326897	1.644261	-0.010903
6	C	-2.588146	-1.378182	-0.043488
1	H	-2.683853	-1.748840	-1.078078
1	H	-2.272947	-2.212350	0.593261
1	H	-3.567923	-1.030092	0.298288

Uncorrected Sum of Electronic and Thermal Free Energies = -441.663675
Corrected Sum of Electronic and Thermal Free Energies = -441.656961

Compound 38b Triplet

Atomic Number	Atomic Type	Coordinates (Angstroms)		
		X	Y	Z
6	C	0.678900	0.499184	0.022381
6	C	-0.261687	-0.574838	0.062545
6	C	-2.079915	1.006825	0.016228
6	C	-1.172157	2.059891	-0.016091
6	C	0.217156	1.847389	-0.001286
6	C	2.003385	-0.086353	-0.008697
6	C	1.807211	-1.516926	0.009361
6	C	0.428492	-1.812040	0.048815
1	H	-3.154026	1.146750	0.059042
1	H	-1.575047	3.070138	-0.054568
1	H	0.908025	2.684422	-0.043333
7	N	-1.636910	-0.325019	0.145940
1	H	-0.011415	-2.802825	0.059412
6	C	3.230283	0.589792	-0.054617
1	H	2.609922	-2.247590	-0.018235
1	H	4.169795	0.043496	-0.079357
1	H	3.284439	1.674552	-0.067423
6	C	-2.568870	-1.390613	-0.127806
1	H	-2.652748	-1.611615	-1.210720
1	H	-2.255710	-2.304589	0.385844
1	H	-3.561652	-1.111474	0.242761

Sum of Electronic and Thermal Free Energies = -441.674668

Compound 39a Singlet

Atomic Number	Atomic Type	Coordinates (Angstroms)		
		X	Y	Z
6	C	-0.247915	-1.061543	-0.000028
6	C	0.043344	0.385473	0.000031
6	C	2.350259	-0.100522	-0.000232
6	C	2.116644	-1.496950	-0.000293
6	C	0.828732	-1.986036	-0.000195
6	C	-1.616547	-1.214838	0.000117
6	C	-2.199068	0.123605	0.000187
6	C	-1.113344	1.109234	0.000198
1	H	3.363171	0.288022	-0.000304
1	H	2.976879	-2.154981	-0.000415
1	H	0.640146	-3.055477	-0.000235
1	H	-2.175059	-2.141591	0.000126
7	N	1.376469	0.806194	-0.000078
6	C	-3.529054	0.393493	0.000281
1	H	-1.248493	2.181248	0.000281
1	H	-3.903453	1.413204	0.000415
1	H	-4.265199	-0.405143	0.000349
6	C	1.663826	2.251243	-0.000013
1	H	1.226699	2.703707	-0.893257
1	H	1.226902	2.703587	0.893391
1	H	2.741869	2.405109	-0.000126

Uncorrected Sum of Electronic and Thermal Free Energies = -441.444774
Corrected Sum of Electronic and Thermal Free Energies = -441.444774

Compound 39a Triplet

Atomic Number	Atomic Type	Coordinates (Angstroms)		
		X	Y	Z
6	C	0.177083	-1.066991	0.000009
6	C	-0.078532	0.339830	-0.000019
6	C	-2.398343	-0.061944	-0.000078
6	C	-2.192791	-1.425597	-0.000014
6	C	-0.885917	-1.960016	-0.000003
6	C	1.611988	-1.224450	0.000078
6	C	2.230102	0.078898	-0.000028
6	C	1.168965	1.047709	0.000033
1	H	-3.384745	0.382301	-0.000123
1	H	-3.058052	-2.078411	0.000007
1	H	-0.727972	-3.033157	0.000129
1	H	2.139078	-2.170312	0.000146
7	N	-1.340828	0.818373	-0.000041
6	C	3.587436	0.362591	-0.000052
1	H	1.302451	2.121853	-0.000039
1	H	3.946919	1.385380	-0.000121
1	H	4.325082	-0.431820	-0.000035
6	C	-1.586824	2.276377	0.000071
1	H	-1.146936	2.721800	0.894824
1	H	-1.148281	2.721805	-0.895352
1	H	-2.660751	2.453505	0.000863

Sum of Electronic and Thermal Free Energies = -441.425390

Compound 39b Singlet

Atomic Number	Atomic Type	Coordinates (Angstroms)		
		X	Y	Z
6	C	-0.211995	-1.094486	-0.000039
6	C	0.050466	0.367171	0.000020
6	C	2.430254	-0.053284	-0.000235
6	C	2.204491	-1.413007	-0.000292
6	C	0.897074	-1.962786	-0.000196
6	C	-1.599309	-1.240154	0.000094
6	C	-2.232919	0.064119	0.000206
6	C	-1.144237	1.050844	0.000173
1	H	3.412716	0.401634	-0.000303
1	H	3.071392	-2.070257	-0.000414
1	H	0.760913	-3.041694	-0.000241
1	H	-2.137755	-2.182523	0.000098
7	N	1.343965	0.833557	-0.000077
6	C	-3.580619	0.360630	0.000339
1	H	-1.291700	2.124880	0.000249
1	H	-3.933996	1.388884	0.000423
1	H	-4.332913	-0.424296	0.000361
6	C	1.556155	2.261551	-0.000011
1	H	1.099484	2.724917	-0.887746
1	H	1.099687	2.724807	0.887887
1	H	2.628256	2.475168	-0.000121

Uncorrected Sum of Electronic and Thermal Free Energies = -441.703661
Corrected Sum of Electronic and Thermal Free Energies = -441.703661

Compound 39b Triplet

Atomic Number	Atomic Type	Coordinates (Angstroms)		
		X	Y	Z
6	C	0.208931	-1.042424	0.005659
6	C	-0.047719	0.364284	-0.076179
6	C	-2.411321	-0.076189	-0.036168
6	C	-2.176438	-1.454101	0.023752
6	C	-0.880777	-1.978432	0.030785
6	C	1.616152	-1.200657	0.041026
6	C	2.228485	0.095482	-0.007920
6	C	1.163113	1.067867	-0.086143
1	H	-3.414067	0.336709	-0.072787
1	H	-3.043507	-2.109663	0.070122
1	H	-0.707131	-3.049891	0.087844
1	H	2.151883	-2.142856	0.109074
7	N	-1.376827	0.851506	-0.200747
6	C	3.620839	0.356026	0.030153
1	H	1.305514	2.141965	-0.121968
1	H	4.010665	1.369793	-0.010981
1	H	4.341174	-0.455296	0.092782
6	C	-1.635411	2.227964	0.158967
1	H	-1.563618	2.398033	1.253861
1	H	-0.915593	2.883598	-0.338550
1	H	-2.642662	2.508148	-0.167764

Sum of Electronic and Thermal Free Energies = -441.662049

Compound 40a Singlet

Atomic Number	Atomic Type	Coordinates (Angstroms)		
		X	Y	Z
6	C	-1.597722	-1.222434	-0.002930
6	C	-1.920941	1.113677	-0.002140
6	C	-0.563958	1.333806	-0.000447
6	C	0.332444	0.236618	-0.000203
6	C	1.748281	0.417992	0.000525
6	C	2.789729	1.258141	0.000715
6	C	3.095070	-0.134411	0.000917
1	H	3.148637	2.279446	0.000306
1	H	-0.195479	2.353165	-0.000069
1	H	-2.643552	1.921236	-0.002482
6	C	-0.228218	-1.061006	-0.001440
7	N	-2.432359	-0.150867	-0.003854
1	H	-2.065812	-2.199127	-0.003824
6	C	3.985466	-1.131385	0.001253
1	H	0.407815	-1.938193	-0.001930
1	H	3.674449	-2.169666	0.001156
1	H	5.048189	-0.917664	0.001621
6	C	-3.901608	-0.333155	0.005588
1	H	-4.311087	0.060193	0.938553
1	H	-4.337248	0.194318	-0.845019
1	H	-4.130666	-1.394690	-0.072369

Uncorrected Sum of Electronic and Thermal Free Energies = -441.388425
Corrected Sum of Electronic and Thermal Free Energies = -441.388425

Compound 40a Triplet

Atomic Number	Atomic Type	Coordinates (Angstroms)		
		X	Y	Z
6	C	-1.602766	-1.223129	-0.015718
6	C	-1.900934	1.134662	-0.012925
6	C	-0.548860	1.336376	-0.005512
6	C	0.354343	0.220622	-0.003045
6	C	1.733347	0.408577	0.002743
6	C	2.865645	1.267541	0.007725
6	C	3.058179	-0.085516	0.005566
1	H	3.278666	2.264376	0.010883
1	H	-0.170438	2.352025	-0.004050
1	H	-2.609903	1.953396	-0.014076
6	C	-0.244073	-1.083769	-0.008176
7	N	-2.438703	-0.129436	-0.023700
1	H	-2.088217	-2.191255	-0.019276
6	C	3.933909	-1.184130	0.005766
1	H	0.371212	-1.975975	-0.008578
1	H	3.553173	-2.198731	0.003014
1	H	5.006645	-1.025417	0.008733
6	C	-3.896750	-0.319958	0.033649
1	H	-4.220577	-0.453072	1.070508
1	H	-4.391435	0.553264	-0.391947
1	H	-4.170445	-1.200215	-0.549743

Sum of Electronic and Thermal Free Energies = -441.353571

Compound 40b Singlet

Atomic Number	Atomic Type	Coordinates (Angstroms)		
		X	Y	Z
6	C	-1.581898	-1.239007	-0.295498
6	C	-1.941382	1.140207	-0.229466
6	C	-0.608543	1.349363	-0.117959
6	C	0.357318	0.263421	-0.068561
6	C	1.718927	0.472091	0.030156
6	C	2.835712	1.280857	0.138268
6	C	3.055199	-0.103324	0.096565
1	H	3.246675	2.275358	0.228268
1	H	-0.248380	2.376635	-0.114275
1	H	-2.649233	1.961048	-0.319658
6	C	-0.245181	-1.052287	-0.184277
7	N	-2.534778	-0.161527	-0.316852
1	H	-2.018903	-2.223376	-0.443028
6	C	3.838348	-1.215465	0.107462
1	H	0.403639	-1.924221	-0.227944
1	H	3.404173	-2.208199	0.035134
1	H	4.920611	-1.141396	0.187644
6	C	-3.662754	-0.355787	0.590461
1	H	-3.363617	-0.346644	1.658784
1	H	-4.402157	0.438679	0.432942
1	H	-4.143841	-1.317610	0.377181

Uncorrected Sum of Electronic and Thermal Free Energies = -441.621382
Corrected Sum of Electronic and Thermal Free Energies = -441.621382

Compound 40b Triplet

Atomic Number	Atomic Type	Coordinates (Angstroms)		
		X	Y	Z
6	C	-1.585794	-1.225416	-0.075268
6	C	-1.942839	1.143467	0.004541
6	C	-0.553241	1.372423	-0.034582
6	C	0.357577	0.269005	-0.009986
6	C	1.750703	0.452073	0.010471
6	C	2.848212	1.268916	0.015234
6	C	3.077721	-0.129491	0.017973
1	H	3.253330	2.269649	0.010306
1	H	-0.183130	2.391513	-0.012780
1	H	-2.677455	1.935167	-0.057959
6	C	-0.208081	-1.052934	-0.009367
7	N	-2.444541	-0.144148	-0.083886
1	H	-2.061913	-2.198059	-0.063221
6	C	3.893177	-1.208061	0.021902
1	H	0.439285	-1.922404	-0.006011
1	H	3.490713	-2.216255	0.021752
1	H	4.974109	-1.093368	0.027687
6	C	-3.868684	-0.369684	0.099604
1	H	-4.146608	-0.348292	1.166272
1	H	-4.440676	0.409552	-0.410997
1	H	-4.148371	-1.340260	-0.330975

Sum of Electronic and Thermal Free Energies = -441.573251

Compound 41a Singlet

Atomic Number	Atomic Type	Coordinates (Angstroms)		
		X	Y	Z
6	C	-1.287819	0.861955	-0.000015
6	C	-0.996484	-1.548992	0.000016
6	C	0.360826	-1.472659	0.000013
6	C	0.958633	-0.155380	-0.000005
6	C	2.356341	0.035630	-0.000004
6	C	3.653954	-0.486507	0.000005
6	C	3.491286	0.848252	-0.000012
1	H	3.937691	1.833738	-0.000024
1	H	4.321562	-1.337223	0.000018
1	H	0.962566	-2.371328	0.000026
1	H	-1.507569	-2.504407	0.000031
6	C	-2.061921	1.992462	-0.000006
1	H	-3.142416	1.977797	0.000019
1	H	-1.580685	2.962050	0.000011
6	C	0.139996	0.963571	-0.000019
1	H	0.566147	1.961405	-0.000018
7	N	-1.816511	-0.450140	0.000002
6	C	-3.268521	-0.608307	0.000009
1	H	-3.701597	-0.141013	0.891252
1	H	-3.701601	-0.141038	-0.891244
1	H	-3.516267	-1.669150	0.000024

Uncorrected Sum of Electronic and Thermal Free Energies = -441.358208
Corrected Sum of Electronic and Thermal Free Energies = -441.359346

Compound 41a Triplet

Atomic Number	Atomic Type	Coordinates (Angstroms)		
		X	Y	Z
6	C	-1.294847	0.855678	-0.000003
6	C	-0.984060	-1.522699	0.000015
6	C	0.386871	-1.399646	0.000013
6	C	0.994930	-0.112608	0.000000
6	C	2.376442	0.029742	-0.000005
6	C	3.692239	-0.496795	-0.000004
6	C	3.549690	0.829249	-0.000015
1	H	4.014534	1.804477	-0.000025
1	H	4.354319	-1.350898	0.000003
1	H	0.987380	-2.302258	0.000021
1	H	-1.461768	-2.494319	0.000025
6	C	-2.167555	1.958180	-0.000010
1	H	-3.244230	1.869436	-0.000020
1	H	-1.747531	2.956055	-0.000011
6	C	0.099609	1.012439	-0.000008
1	H	0.493501	2.021551	-0.000018
7	N	-1.820445	-0.447568	0.000006
6	C	-3.276390	-0.676852	0.000009
1	H	-3.727116	-0.240692	0.894078
1	H	-3.727119	-0.240694	-0.894060
1	H	-3.460431	-1.749813	0.000010

Sum of Electronic and Thermal Free Energies = -441.343721

Compound 41b Singlet

Atomic Number	Atomic Type	Coordinates (Angstroms)		
		X	Y	Z
6	C	-1.309291	0.879613	-0.002658
6	C	-0.969518	-1.546628	-0.015113
6	C	0.380230	-1.429661	-0.028366
6	C	1.000102	-0.115200	-0.024089
6	C	2.382382	0.012615	-0.010138
6	C	3.635500	-0.499835	0.045738
6	C	3.613879	0.904760	-0.128040
1	H	3.963034	1.651742	0.602924
1	H	4.249458	-1.378052	0.215659
1	H	0.993882	-2.325189	-0.046588
1	H	-1.467624	-2.511372	-0.023100
6	C	-2.182297	1.957684	0.005581
1	H	-3.259331	1.861032	0.004830
1	H	-1.772330	2.960928	-0.006248
6	C	0.112178	0.998203	-0.025708
1	H	0.525126	2.001257	-0.039975
7	N	-1.826629	-0.445370	0.013110
6	C	-3.252215	-0.658766	0.031165
1	H	-3.716912	-0.193913	0.913678
1	H	-3.737989	-0.233001	-0.860651
1	H	-3.456607	-1.732553	0.057470

Uncorrected Sum of Electronic and Thermal Free Energies = -441.573544
Corrected Sum of Electronic and Thermal Free Energies = -441.576673

Compound 41b Triplet

Atomic Number	Atomic Type	Coordinates (Angstroms)		
		X	Y	Z
6	C	-1.318622	0.871282	-0.007435
6	C	-0.934222	-1.548051	-0.015805
6	C	0.412446	-1.404844	-0.052394
6	C	1.006735	-0.081166	-0.060259
6	C	2.397153	0.063161	-0.091713
6	C	3.674881	-0.561587	-0.015848
6	C	3.524173	0.904291	-0.039510
1	H	3.794838	1.728473	0.622177
1	H	4.063860	-1.256613	0.735953
1	H	1.047928	-2.283880	-0.083592
1	H	-1.417210	-2.520081	-0.012149
6	C	-2.200830	1.939335	0.009461
1	H	-3.276545	1.832092	0.035696
1	H	-1.800844	2.946373	-0.009721
6	C	0.103179	1.014075	-0.047481
1	H	0.494958	2.025842	-0.072226
7	N	-1.809189	-0.459401	0.018878
6	C	-3.231435	-0.693199	0.060332
1	H	-3.686444	-0.241714	0.954916
1	H	-3.736194	-0.264909	-0.819261
1	H	-3.420781	-1.769565	0.079979

Sum of Electronic and Thermal Free Energies = -441.573620

Compound 42a Singlet

Atomic Number	Atomic Type	Coordinates (Angstroms)		
		X	Y	Z
6	C	2.981855	-1.581075	-0.048256
6	C	1.685863	-2.074396	-0.176046
6	C	0.588388	-1.204858	-0.156230
6	C	0.814535	0.201670	-0.063918
6	C	3.151656	-0.229874	0.163146
6	C	-0.767185	-1.740260	-0.171018
6	C	-0.330817	1.126968	-0.253421
6	C	-1.640723	0.543630	0.032398
6	C	-1.828034	-0.916047	-0.019424
6	C	-3.254691	-1.182671	0.147446
1	H	-3.710743	-2.163346	0.160758
6	C	-3.866957	0.020191	0.318339
6	C	-2.869712	1.099936	0.240282
1	H	-0.880031	-2.818846	-0.233467
1	H	3.846977	-2.233220	-0.064649
1	H	1.514834	-3.141598	-0.278832
1	H	-4.922395	0.182403	0.498312
1	H	-3.100351	2.153191	0.348156
7	N	2.094251	0.630572	0.173101
6	C	2.424172	2.032771	0.552128
1	H	2.737042	2.599766	-0.326144
1	H	1.558074	2.500587	1.012788
1	H	3.242320	1.997989	1.270511
6	C	-0.206272	2.340206	-0.847862
1	H	4.121646	0.212591	0.350331
1	H	-1.095776	2.903220	-1.107723
1	H	0.736189	2.776113	-1.147134

Uncorrected Sum of Electronic and Thermal Free Energies = -595.035912
Corrected Sum of Electronic and Thermal Free Energies = -595.035912

Compound 42a Triplet

Atomic Number	Atomic Type	Coordinates (Angstroms)		
		X	Y	Z
6	C	3.021750	-1.538510	-0.131631
6	C	1.745915	-2.068695	-0.148710
6	C	0.612369	-1.231680	-0.078038
6	C	0.794526	0.200907	-0.048222
6	C	3.159322	-0.167160	0.041003
6	C	-0.688872	-1.800997	-0.026022
6	C	-0.361079	1.090032	-0.168718
6	C	-1.624696	0.461597	-0.000410
6	C	-1.784857	-0.963196	0.068486
6	C	-3.194192	-1.226429	0.163411
1	H	-3.651485	-2.206318	0.225105
6	C	-3.881949	0.021982	0.157903
6	C	-2.965267	1.049510	0.045913
1	H	-0.792387	-2.880846	-0.048827
1	H	3.906780	-2.160080	-0.191293
1	H	1.598535	-3.142878	-0.208045
1	H	-4.955322	0.140059	0.231375
1	H	-3.200017	2.104886	0.022449
7	N	2.095838	0.662847	0.104221
6	C	2.414973	2.068941	0.474067
1	H	2.572182	2.675640	-0.418779
1	H	1.609008	2.481088	1.076274
1	H	3.328760	2.058402	1.066088
6	C	-0.332719	2.442747	-0.543599
1	H	4.129432	0.296980	0.166146
1	H	-1.259096	2.998306	-0.603632
1	H	0.551406	2.960534	-0.879010

Sum of Electronic and Thermal Free Energies = -595.015763

Compound 42b Singlet

Atomic Number	Atomic Type	Coordinates (Angstroms)		
		X	Y	Z
6	C	-3.033311	-1.520236	0.187959
6	C	-1.763932	-2.075129	0.180437
6	C	-0.594309	-1.252169	0.063882
6	C	-0.774721	0.205643	0.053085
6	C	-3.185418	-0.143184	0.010296
6	C	0.691914	-1.783937	-0.011292
6	C	0.354439	1.094849	0.242999
6	C	1.659839	0.492023	0.028310
6	C	1.818821	-0.941075	-0.092732
6	C	3.211396	-1.218538	-0.216441
1	H	3.657253	-2.202520	-0.324497
6	C	3.889578	0.012450	-0.177176
6	C	2.946759	1.065437	-0.014974
1	H	0.807122	-2.867840	-0.004409
1	H	-3.921208	-2.137112	0.294100
1	H	-1.630751	-3.150721	0.269734
1	H	4.965284	0.144939	-0.261681
1	H	3.186868	2.121631	0.047431
7	N	-2.079219	0.676795	-0.113250
6	C	-2.365687	2.001447	-0.673861
1	H	-2.777962	2.680298	0.083287
1	H	-1.452263	2.440701	-1.069332
1	H	-3.102519	1.883918	-1.477321
6	C	0.234712	2.420377	0.668926
1	H	-4.146836	0.340869	-0.105866
1	H	1.129841	3.004164	0.850650
1	H	-0.700765	2.856368	0.994147

Uncorrected Sum of Electronic and Thermal Free Energies = -595.276083
Corrected Sum of Electronic and Thermal Free Energies = -595.275626

Compound 42b Triplet

Atomic Number	Atomic Type	Coordinates (Angstroms)		
		X	Y	Z
6	C	3.031743	-1.474161	-0.272242
6	C	1.762749	-2.051935	-0.103971
6	C	0.606902	-1.212657	-0.011520
6	C	0.771300	0.210652	-0.069955
6	C	3.185265	-0.106174	-0.217409
6	C	-0.699200	-1.758886	0.081623
6	C	-0.359454	1.096247	-0.263183
6	C	-1.673740	0.502607	-0.059382
6	C	-1.823006	-0.931166	0.105816
6	C	-3.224177	-1.214996	0.225432
1	H	-3.663559	-2.197858	0.362441
6	C	-3.903577	0.003185	0.139620
6	C	-2.960076	1.062403	-0.043021
1	H	-0.801765	-2.842583	0.134472
1	H	3.909378	-2.093454	-0.443046
1	H	1.636404	-3.130992	-0.099137
1	H	-4.980450	0.134033	0.206296
1	H	-3.209576	2.114076	-0.137714
7	N	2.093447	0.746444	-0.009952
6	C	2.377508	1.830848	0.939181
1	H	1.506631	2.473442	1.050611
1	H	2.654321	1.422613	1.925435
1	H	3.216930	2.432677	0.567763
6	C	-0.214247	2.414536	-0.680876
1	H	4.150994	0.385721	-0.288331
1	H	-1.090053	3.023744	-0.875995
1	H	0.748679	2.830451	-0.953815

Sum of Electronic and Thermal Free Energies = -595.281084

Compound 43a Singlet

Atomic Number	Atomic Type	Coordinates (Angstroms)		
		X	Y	Z
6	C	-3.384423	-1.331810	-0.000307
6	C	-2.124992	-1.900575	-0.000305
6	C	-0.964888	-1.092664	-0.000136
6	C	-1.116343	0.360435	0.000069
6	C	-3.494027	0.061628	-0.000119
6	C	0.319611	-1.662667	-0.000125
6	C	-0.012058	1.201453	0.000268
6	C	1.284811	0.632697	0.000290
6	C	1.451381	-0.836450	0.000094
6	C	2.815839	-1.127528	0.000373
6	C	3.530734	0.146579	-0.000293
6	C	2.541959	1.219311	0.000382
1	H	-2.011590	-2.980737	-0.000443
1	H	2.772889	2.276615	0.000493
7	N	-2.426440	0.869453	0.000063
6	C	-2.633664	2.330689	0.000256
1	H	-2.183373	2.767506	0.894212
1	H	-2.183260	2.767757	-0.893520
1	H	-3.702728	2.535021	0.000217
1	H	-4.461359	0.549937	-0.000111
1	H	-0.115633	2.278992	0.000397
1	H	3.278503	-2.105815	0.000494
1	H	-4.286624	-1.930576	-0.000446
6	C	4.881594	0.296101	-0.000482
1	H	5.545429	-0.563176	-0.000731
1	H	5.342167	1.279441	-0.000582
1	H	0.417453	-2.744331	-0.000194

Uncorrected Sum of Electronic and Thermal Free Energies = -595.045394
Corrected Sum of Electronic and Thermal Free Energies = -595.046785

Compound 43a Triplet

Atomic Number	Atomic Type	Coordinates (Angstroms)		
		X	Y	Z
6	C	-3.409501	-1.305430	0.000025
6	C	-2.146567	-1.888090	0.000021
6	C	-0.989981	-1.088926	0.000020
6	C	-1.136708	0.345432	0.000035
6	C	-3.513646	0.075745	0.000032
6	C	0.319340	-1.663763	0.000012
6	C	0.003328	1.188475	0.000040
6	C	1.262747	0.606041	0.000036
6	C	1.428173	-0.843546	0.000023
6	C	2.828882	-1.115873	0.000094
6	C	3.547454	0.136568	-0.000147
6	C	2.561234	1.189158	0.000007
1	H	-2.043065	-2.968791	0.000014
1	H	2.781917	2.249954	-0.000020
7	N	-2.415978	0.871139	0.000036
6	C	-2.608706	2.336589	0.000044
1	H	-2.157953	2.771314	0.894559
1	H	-2.157941	2.771326	-0.894459
1	H	-3.675982	2.548756	0.000039
1	H	-4.470003	0.582593	0.000035
1	H	-0.093033	2.266352	0.000035
1	H	3.285261	-2.098021	0.000123
1	H	-4.312500	-1.903770	0.000023
6	C	4.918890	0.306761	-0.000234
1	H	5.591193	-0.544170	-0.000253
1	H	5.361308	1.296871	-0.000423
1	H	0.413013	-2.745238	0.000029

Sum of Electronic and Thermal Free Energies =

Compound 43b Singlet

Atomic	Atomic	Coordinates (Angstroms)		
Number	Type	X	Y	Z
6	C	-3.437372	-1.287583	-0.000086
6	C	-2.162531	-1.886156	-0.000162
6	C	-0.982991	-1.112981	-0.000100
6	C	-1.128182	0.362152	0.000053
6	C	-3.560398	0.081930	0.000050
6	C	0.310072	-1.664273	-0.000181
6	C	-0.011198	1.182457	0.000112
6	C	1.302915	0.620145	0.000034
6	C	1.468805	-0.860740	-0.000117
6	C	2.832212	-1.131366	-0.000177
6	C	3.562899	0.125111	0.000000
6	C	2.553006	1.192004	0.000055
1	H	-2.073498	-2.969851	-0.000272
1	H	2.783679	2.253119	0.000142
7	N	-2.423469	0.881718	0.000118
6	C	-2.592799	2.320837	0.000261
1	H	-2.127962	2.771138	0.887835
1	H	-2.127977	2.771312	-0.887233
1	H	-3.658339	2.557801	0.000293
1	H	-4.507454	0.605800	0.000113
1	H	-0.106980	2.263151	0.000213
1	H	3.295048	-2.112846	-0.000298
1	H	-4.338107	-1.894854	-0.000137
6	C	4.922126	0.315519	0.000043
1	H	5.614020	-0.523223	-0.000041
1	H	5.352102	1.314027	0.000151
1	H	0.400375	-2.749933	-0.000296

Uncorrected Sum of Electronic and Thermal Free Energies = -595.307457
Corrected Sum of Electronic and Thermal Free Energies = -595.307457

Compound 43b Triplet

Atomic Number	Atomic Type	Coordinates (Angstroms)		
		X	Y	Z
6	C	-3.431250	-1.320419	0.000074
6	C	-2.164234	-1.914061	0.000055
6	C	-0.985800	-1.086362	0.000028
6	C	-1.124820	0.354012	0.000022
6	C	-3.561539	0.058990	0.000069
6	C	0.309095	-1.633999	0.000012
6	C	-0.009314	1.183740	0.000003
6	C	1.302782	0.633107	-0.000014
6	C	1.455799	-0.821617	-0.000009
6	C	2.841716	-1.107590	-0.000015
6	C	3.557607	0.139414	-0.000097
6	C	2.585306	1.203865	-0.000026
1	H	-2.052002	-2.994634	0.000060
1	H	2.822905	2.263225	-0.000025
7	N	-2.446912	0.892243	0.000043
6	C	-2.630289	2.321555	0.000038
1	H	-2.176498	2.788233	0.887980
1	H	-2.176529	2.788224	-0.887923
1	H	-3.699501	2.547290	0.000056
1	H	-4.522507	0.559608	0.000085
1	H	-0.121418	2.263884	0.000004
1	H	3.299371	-2.091962	-0.000007
1	H	-4.335233	-1.923657	0.000095
6	C	4.960905	0.264215	-0.000176
1	H	5.602402	-0.612967	-0.000201
1	H	5.444027	1.237816	-0.000212
1	H	0.407578	-2.719864	0.000021

Sum of Electronic and Thermal Free Energies = -595.287445
